# Supplementary material for: Conversion of anilines to chiral benzylic amines via formal one-carbon insertion into aromatic C–N bonds
Source: Nat Commun. 2020 Sep 23;11:4805. doi: 10.1038/s41467-020-18593-4 (PMC7511326; doi:10.1038/s41467-020-18593-4)
Supplement: Supplementary file 1 — Supplementary Information [file 41467_2020_18593_MOESM1_ESM.pdf]

Conversion of Anilines to Chiral Benzylic Amines via Formal One-Carbon Insertion into Aromatic C-N Bonds

Li, et al.

Conversion of Anilines to Chiral Benzylic Amines via Formal One-Carbon Insertion into Aromatic C-N Bonds

Lei Li, Min Yang, Qiuqin He,* Renhua Fan*

*Corresponding author. Email: rhfan@fudan.edu.cn

Supplementary Information

Supplementary Tables……………………………………………….S2

Supplementary Methods…………………………………….........S4

Supplementary Figures…………………………………………….S21

Supplementary References……………………………………….S172

Supplementary Tables

Supplementary Table 1：evaluation of oxidative dearomatization

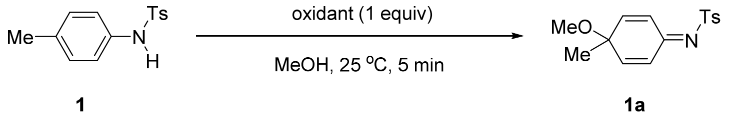

entry	oxidant	yield
1	PhIO	95 %
2	$PhI(OAc)_2$	92 %
3	mCPBA	N.D.
4	AcOOH	N.D.
5	*t*-BuOOH	N.D.
6	H_2O_2	N.D.

Supplementary Table 2：evaluation of nucleophilic attack

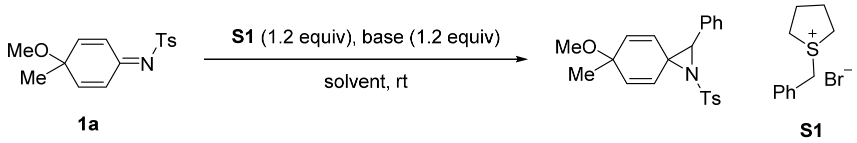

entry	base	solvent	yield
1	NaH	MeCN	92 %
2	K_2CO_3	MeCN	27%
3	Cs_2CO_3	MeCN	81%
4	$NaOCH_3$	MeCN	26%
5	*t*-BuOK	MeCN	65%
6	NaH	THF	42 %
7	NaH	DCM	33 %
8	NaH	DCE	27 %
9	NaH	1,4-dioxane	32 %
10	NaH	EA	24 %

Supplementary Table 3：evaluation of tandem reaction

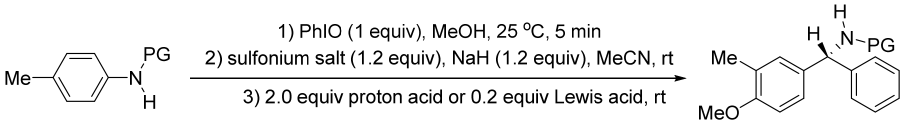

1 **2**

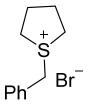
S1
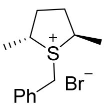
S2
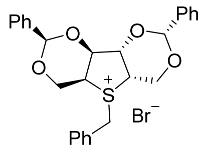
S3
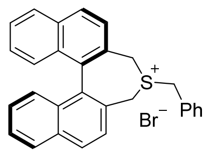
S4
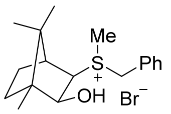
S5

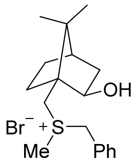
S6
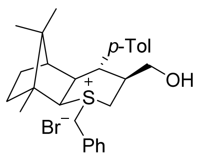
S7
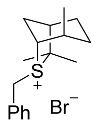
S8
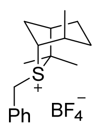
S9
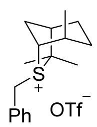
S10

entry	PG	sulfonium salt	acid	yield	e.e.
1	Ts	S1	TFA	71%	/
2	Ts	S2	TFA	51%	17%
3	Ts	S3	TFA	<5%	/
4	Ts	S4	TFA	15%	<5%
5	Ts	S5	TFA	33%	<5%
6	Ts	S6	TFA	37%	<5%
7	Ts	S7	TFA	<5%	/
8	Ts	S8	TFA	64%	95%
9	Ts	S9	TFA	77%	93%
10	Ts	S10	TFA	78%	94%
11	Ns	S10	$Cu(OTf)_2$	53%	94%
12	Ms	S10	$Cu(OTf)_2$	75%	95%
13	Ts	S10	$Cu(OTf)_2$	79%	96%
14	Ts	S10	$Yb(OTf)_3$	81%	93%
15	Ts	S10	$Zn(OTf)_2$	75%	93%
16	Ts	S10	$Bi(OTf)_3$	74%	81%
17	Ts	S10	$Sc(OTf)_3$	80%	94%
18	Ts	S10	AgOTf	N.D.	/
19	Ts	S10	$In(OTf)_3$	80%	92%
20	Ts	S10	$ZrCl_4$	55%	91%
21	Ts	S10	$TsOH\cdot H_2O$	55%	11%
22	Ts	S10	*p*-$NO_2C_6H_4COOH$	34%	93%
23	Ts	S10	AcOH	25%	93%
24	Ts	S10	$Cu(OAc)_2$	44%	93%
25	Ts	S10	$CuBr_2$	51%	94%

Supplementary Methods

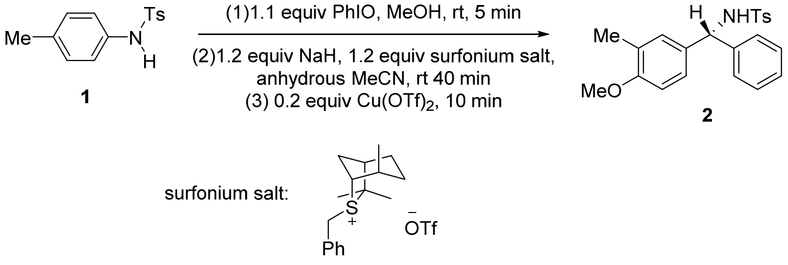

PhIO (0.22 mmol) was added to a solution of N-Ts p-toluidine (**1**) (0.2 mmol) in MeOH (2.0 mL) at 25 °C. After 5 min, the reaction mixture was concentrated in vacuo. The resulting mixture was mixed with the sulfonium salt (0.24 mmol)[1-2] and sodium hydride (0.24 mmol) in MeCN (2 mL). The reaction was stirred at rt for 3 h, then $Cu(OTf)_2$ (0.02 mmol) was added. After the intermediate was completely consumed (monitored by TLC analysis), the reaction was quenched with saturated $NaHCO_3$ (25 mL), and extracted with EtOAc (25 mL×3). The organic layer was dried over Na_2SO_4, and concentrated in vacuo. The residue was purified by flash column chromatography on silica gel (eluent: petroleum ether/EtOAc) to furnish the desired compound **2**.

The procedure[3] for synthesis of compound **48**

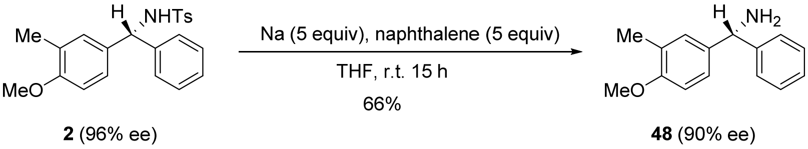

To dry THF (5 mL) under nitrogen was added Na metal (5.0 equiv, 1.5 mmol, 34.5 mg) and naphthalene (5.0 equiv, 1.5 mmol, 192.3 mg). The mixture was stirred for 1 h at room temperature. To this solution was added a solution of compound **2** (1.0 equiv, 0.3 mmol, 114.3 mg) in dry THF (6 mL). The reaction was stirred at room temperature for 15 h. The mixture was quenched by addition of H_2O and extracted with ethylacetate. The combined organic layer was dried over Na_2SO_4 and concentrated under reduced pressure. Purification of the crude residue by flash column chromatography (DCM/MeOH 100:1→50:1) afforded the analytically compound **48** as yellow oil.

The procedure[4] for synthesis of compound **49**

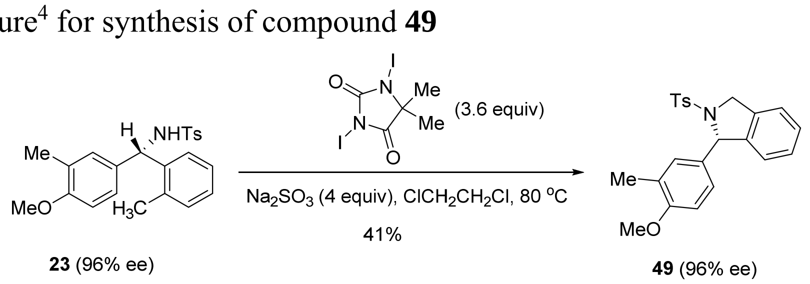

A mixture of Na_2SO_3 (4 equiv) and 1,3-Diiodo-5,5-dimethylhydantoin (DIH, 3.6 equiv) was add to compound **23** (39.5 mg, 0.1 mmol). The system was sealed and flushed with N_2. DCE (1.5 mL) was added, and the resulting yellow mixture was heated at 80 °C for 12 h. The reaction was quenched with saturated solution of Na_2SO_3. The layers were separated and the aqueous layer was extracted with CH_2Cl_2. The combined organic fractions were dried over Na_2SO_4 and concentrated under vacuum. The resulting residue was purified by flash chromatography, providing the product **49**.

The procedure[5] for synthesis of compound **50**

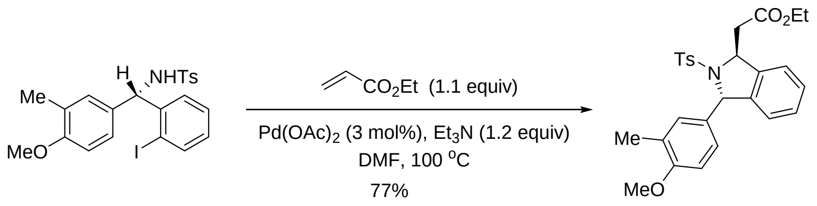

33 (93% ee) **50** (9:1 d.r., 93% ee)

A mixture of $Pd(OAc)_2$ (0.03 equiv), compound **33** (253.5 mg, 0.5 mmol), ethyl acrylate (1.1 equiv), NEt_3 (1.2 equiv) and DMF (1.0 mL) were placed in a screw caped reaction vial. The resulting mixture was heated at 100 °C and stirred for 24 h. After the substrate was consumed completely (monitored by TLC analysis), the mixture was passed through a short silica gel column and then concentrated under reduced pressure. The residue was purified by flash column chromatography on silica gel (petroleum ether/ethyl acetate = 5/1) to furnish compound **50**.

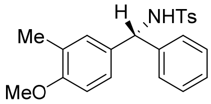

2

(R)-N-((4-methoxy-3-methylphenyl)(phenyl)methyl)-4-methylbenzenesulfonamide 2: 60.2 mg, 79% yield, 96% ee. White solid, mp:105-106 ℃. ^{1}H NMR (400 MHz, Chloroform-*d*) δ 7.54 (d, *J* = 8.2 Hz, 2H), 7.22 – 7.17 (m, 3H), 7.15 – 7.10 (m, 4H), 6.84 (dd, *J* = 8.4, 1.9 Hz, 1H), 6.80 – 6.72 (m, *J* = 2.0 Hz, 1H), 6.62 (d, *J* = 8.4 Hz, 1H), 5.48 (d, *J* = 7.1 Hz, 1H), 5.23 (d, *J* = 7.1 Hz, 1H), 3.75 (s, 3H), 2.37 (s, 3H), 2.05 (s, 3H); ^{13}C NMR (101 MHz, $CDCl_3$) δ 157.1, 143.0, 140.8, 137.4, 132.1, 129.7, 129.2, 128.4, 127.3, 127.2, 126.7, 125.8, 109.6, 60.9, 55.3, 21.4, 16.1; HRMS (m/z): $[M+Na]^+$ calcd. for $C_{22}H_{23}NO_3SNa$, 404.1291; found, 404.1291.

The ee value was determined by HPLC analysis: Chiralcel OD-H Column, hexane/2-propanol= 90/10, 25 ℃, 1.0 mL/min, 220 nm, retention time: 15.8 min (minor) and 21.3 min (major).

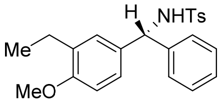

3

(R)-N-((3-ethyl-4-methoxyphenyl)(phenyl)methyl)-4-methylbenzenesulfonamide 3: 55.2 mg, 70% yield, 94% ee. White solid, mp:112-113 ℃. ^{1}H NMR (400 MHz, Chloroform-*d*) δ 7.55 (d, *J* = 8.3 Hz, 2H), 7.23 – 7.18 (m, 3H), 7.16 – 7.14 (m, 2H), 7.12 (d, *J* = 8.6 Hz, 2H), 6.82 (dd, *J* = 8.4, 2.3 Hz, 1H), 6.77 (d, *J* = 2.2 Hz, 1H), 6.63 (d, *J* = 8.4 Hz, 1H), 5.50 (d, *J* = 7.0 Hz, 1H), 5.17 (d, *J* = 7.0 Hz, 1H), 3.75 (s, 3H), 2.47 (q, *J* = 7.5 Hz, 2H), 2.37 (s, 3H), 1.05 (t, *J* = 7.5 Hz, 3H); ^{13}C NMR (101 MHz, $CDCl_3$) δ 156.7, 143.0, 140.8, 137.4, 132.7, 132.3, 129.3, 128.4, 128.1, 127.3, 127.2, 127.2, 125.8, 109.9, 60.9, 55.3, 23.2, 21.4, 13.9; HRMS (m/z): $[M+Na]^+$ calcd. for $C_{23}H_{25}NO_3SNa$, 418.1447; found, 418.1457.

The ee value was determined by HPLC analysis: Chiralcel OD-H Column, hexane/2-propanol= 90/10, 25 ℃, 1.0 mL/min, 214 nm, retention time: 13.0 min (minor) and 16.6 min (major).

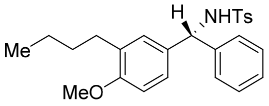

4

(R)-N-((3-butyl-4-methoxyphenyl)(phenyl)methyl)-4-methylbenzenesulfonamide 4: 53.1 mg, 63% yield, 93% ee. White solid, mp:130-131 ℃. ^{1}H NMR (400 MHz, Chloroform-*d*) δ 7.55 (d, *J* = 8.2 Hz, 2H), 7.21 – 7.18 (m, 3H), 7.15 – 7.11 (m, 4H), 6.81 (dd, *J* = 8.4, 2.2 Hz, 1H), 6.76 (d, *J* = 2.1 Hz, 1H), 6.62 (d, *J* = 8.4 Hz, 1H), 5.49 (d, *J* = 7.0 Hz, 1H), 5.14 (d, *J* = 7.0 Hz, 1H), 3.75 (s, 3H), 2.45 – 2.42 (m, 2H), 2.37 (s, 3H), 1.41 (p, *J* = 7.2 Hz, 2H), 1.34 – 1.28 (m, 2H), 0.90 (t, *J* = 7.2 Hz, 3H); ^{13}C NMR (101 MHz, $CDCl_3$) δ 156.8, 143.0, 140.9, 137.4, 132.1, 131.4, 129.3, 128.8, 128.4, 127.3, 127.3, 127.2, 125.8, 109.9, 60.9, 55.3, 31.8, 29.8, 22.6, 21.4, 14.0; HRMS (m/z): $[M+Na]^+$ calcd. for $C_{25}H_{29}NO_3SNa$, 446.1760; found, 446.1778.

The ee value was determined by HPLC analysis: Chiralcel OD-H Column, hexane/2-propanol= 90/10, 25 ℃, 1.0 mL/min, 254 nm, retention time: 9.3 min (minor) and 11.8 min (major).

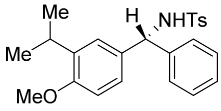

5

(S)-N-((3-isopropyl-4-methoxyphenyl)(phenyl)methyl)-4-methylbenzenesulfonamide 5: 45.2 mg, 55% yield, 89% ee. White oil. ^{1}H NMR (400 MHz, Chloroform-*d*) δ 7.57 (d, *J* = 8.3 Hz, 2H), 7.24 – 7.12 (m, 7H), 6.83 (d, *J* = 2.2 Hz, 1H), 6.79 (dd, *J* = 8.4, 2.2 Hz, 1H), 6.63 (d, *J* = 8.4 Hz, 1H), 5.52 (d, *J* = 7.0 Hz, 1H), 5.10 (d, *J* = 6.9 Hz, 1H), 3.75 (s, 3H), 3.18 (hept, *J* = 6.9 Hz, 1H), 2.37 (s, 3H), 1.06 (t, *J* = 6.5 Hz, 6H); ^{13}C NMR (101 MHz, $CDCl_3$) δ 156.2, 143.0, 140.9, 137.5, 137.1, 132.4, 129.3, 128.4, 127.3, 127.3, 127.2, 125.6, 125.3, 110.2, 61.1, 55.3, 26.7, 22.4, 22.3, 21.4; HRMS (m/z): $[M+Na]^+$ calcd. for $C_{24}H_{27}NO_3SNa$, 432.1604; found, 432.1616.

The ee value was determined by HPLC analysis: Chiralpak IC Column, hexane/2-propanol= 90/10, 25 ℃, 1.0 mL/min, 214 nm, retention time: 19.7 min (major) and 21.6 min (minor).

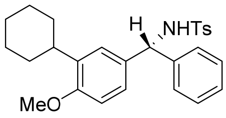

6

(R)-N-((3-cyclohexyl-4-methoxyphenyl)(phenyl)methyl)-4-methylbenzenesulfonamide 6: 53.7 mg, 60% yield, 74% ee. White solid, mp:143-144 ℃. ^{1}H NMR (400 MHz, Chloroform-*d*) δ 7.57 (d, *J* = 8.3 Hz, 2H), 7.24 – 7.12 (m, 7H), 6.82 (d, *J* = 2.3 Hz, 1H), 6.75 (dd, *J* = 8.4, 2.3 Hz, 1H), 6.62 (d, *J* = 8.4 Hz, 1H), 5.51 (d, *J* = 7.0 Hz, 1H), 5.13 (d, *J* = 7.0 Hz, 1H), 3.74 (s, 3H), 2.81 (tt, *J* = 11.9, 3.1 Hz, 1H), 2.37 (s, 3H), 1.79 – 1.64 (m, 5H), 1.42 – 1.32 (m, 2H), 1.21 – 1.12 (m, 3H); ^{13}C NMR (101 MHz, $CDCl_3$) δ 156.1, 143.0, 140.9, 137.5, 136.3, 132.4, 129.3, 128.4, 127.3, 127.2, 125.8, 125.5, 110.1, 61.1, 55.4, 36.8, 33.0, 32.9, 27.0, 27.0, 26.3, 21.4; HRMS (m/z): $[M+Na]^+$ calcd. for $C_{27}H_{31}NO_3SNa$, 472.1917; found, 472.1926.

The ee value was determined by HPLC analysis: Chiralpak IC Column, hexane/2-propanol= 90/10, 25 ℃, 1.0 mL/min, 214 nm, retention time: 19.9 min (major) and 22.3 min (minor).

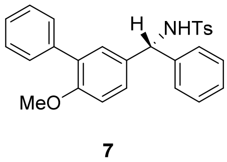

7

(R)-N-((6-methoxy-[1,1'-biphenyl]-3-yl)(phenyl)methyl)-4-methylbenzenesulfonamide 7: 63.5 mg, 72% yield, 96% ee. White solid, mp:122-123 ℃. ^{1}H NMR (400 MHz, Chloroform-*d*) δ 7.56 (d, *J* = 8.2

Hz, 2H), 7.35 – 7.34 (m, 4H), 7.31 – 7.28 (m, 1H), 7.21 – 7.15 (m, 5H), 7.09 (d, *J* = 8.0 Hz, 2H), 7.02 (dd, *J* = 8.5, 2.2 Hz, 1H), 6.96 (d, *J* = 2.2 Hz, 1H), 6.78 (d, *J* = 8.5 Hz, 1H), 5.56 (d, *J* = 7.1 Hz, 1H), 5.34 (d, *J* = 7.1 Hz, 1H), 3.73 (s, 3H), 2.32 (s, 3H); ^{13}C NMR (101 MHz, $CDCl_3$) δ 155.8, 143.1, 140.7, 137.9, 137.4, 132.8, 130.5, 129.9, 129.3, 129.3, 128.5, 127.9, 127.6, 127.5, 127.2, 127.2, 127.0, 111.2, 60.9, 55.6, 21.4; HRMS (m/z): $[M+Na]^+$ calcd. for $C_{27}H_{25}NO_3SNa$, 466.1447; found, 466.1438.
The ee value was determined by HPLC analysis: Chiralpak IC Column, hexane/2-propanol= 90/10, 25 ℃, 1.0 mL/min, 214 nm, retention time: 30.9 min (major) and 33.3 min (minor).

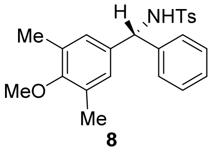

8

(R)-N-((4-methoxy-3,5-dimethylphenyl)(phenyl)methyl)-4-methylbenzenesulfonamide 8: 45.9 mg, 58% yield, 95% ee. White solid, mp:109-110 ℃. ^{1}H NMR (400 MHz, Chloroform-*d*) δ 7.54 (d, *J* = 7.7 Hz, 2H), 7.21 – 7.20 (m, 3H), 7.13 – 7.11 (m, 4H), 6.65 (s, 2H), 5.45 (d, *J* = 6.9 Hz, 1H), 5.15 (d, *J* = 6.8 Hz, 1H), 3.64 (s, 3H), 2.37 (s, 3H), 2.11 (s, 6H); ^{13}C NMR (101 MHz, $CDCl_3$) δ 156.2, 143.0, 140.6, 137.5, 135.5, 130.8, 129.2, 128.4, 127.8, 127.4, 127.3, 127.2, 61.0, 59.6, 21.4, 16.0; HRMS (m/z): $[M+Na]^+$ calcd. for $C_{23}H_{25}NO_3SNa$, 418.1447; found, 418.1458.
The ee value was determined by HPLC analysis: Chiralcel OD-H Column, hexane/2-propanol= 90/10, 25 ℃, 1.0 mL/min, 254 nm, retention time: 10.9 min (major) and 15.2 min (minor).

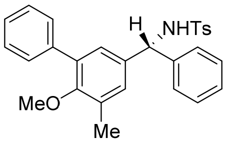

9

(R)-N-((6-methoxy-5-methyl-[1,1'-biphenyl]-3-yl)(phenyl)methyl)-4-methylbenzenesulfonamide 9: 64.8 mg, 71% yield, 98% ee. White solid, mp:121-122 ℃. ^{1}H NMR (400 MHz, Chloroform-*d*) δ 7.56 (d, *J* = 7.5 Hz, 2H), 7.40 – 7.29 (m, 5H), 7.22 – 7.15 (m, 5H), 7.10 (d, *J* = 7.5 Hz, 2H), 6.83 (s, 2H), 5.54 (d, *J* = 6.7 Hz, 1H), 5.26 (d, *J* = 6.6 Hz, 1H), 3.29 (s, 3H), 2.32 (s, 3H), 2.17 (s, 3H); ^{13}C NMR (101 MHz, $CDCl_3$) δ 155.2, 143.1, 140.5, 138.3, 137.4, 135.7, 134.5, 131.7, 129.2, 128.9, 128.5, 128.1, 127.7, 127.5, 127.2, 127.2, 127.1, 61.0, 59.8, 21.4, 16.2; HRMS (m/z): $[M+Na]^+$ calcd. for $C_{28}H_{27}NO_3SNa$, 480.1604; found, 480.1618.
The ee value was determined by HPLC analysis: Chiralcel OD-H Column, hexane/2-propanol= 90/10, 25 ℃, 1.0 mL/min, 254 nm, retention time: 9.6 min (major) and 13.1 min (minor).

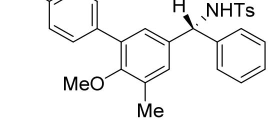

10

(R)-N-((6-methoxy-4',5-dimethyl-[1,1'-biphenyl]-3-yl)(phenyl)methyl)-4-methylbenzenesulfonamide 10: 76.3 mg, 81% yield, 96% ee. White solid, mp:90-91 ℃. ^{1}H NMR (400 MHz, Chloroform-*d*) δ 7.55 (d, *J* = 8.3 Hz, 2H), 7.29 (d, *J* = 8.1 Hz, 2H), 7.24 – 7.15 (m, 7H), 7.10 (d, *J* = 8.0 Hz, 2H), 6.81 (s, 2H), 5.53 (d, *J* = 7.1 Hz, 1H), 5.24 (d, *J* = 7.1 Hz, 1H), 3.30 (s, 3H), 2.38 (s, 3H), 2.32 (s, 3H), 2.17 (s, 3H); ^{13}C NMR (101 MHz, $CDCl_3$) δ 155.2, 143.0, 140.5, 137.4, 136.8, 135.6, 135.3,

134.5, 131.7, 129.2, 128.9, 128.9, 128.7, 128.5, 127.7, 127.5, 127.2, 127.2, 61.0, 59.8, 21.4, 21.2, 16.2; HRMS (m/z): $[M+Na]^+$ calcd. for $C_{29}H_{29}NO_3SNa$, 494.1760; found, 494.1775.

The ee value was determined by HPLC analysis: Chiralcel OD-H Column, hexane/2-propanol= 90/10, 25 ℃, 1.0 mL/min, 214 nm, retention time: 9.9 min (major) and 19.0 min (minor).

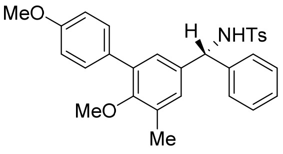

11

(R)-N-((4',6-dimethoxy-5-methyl-[1,1'-biphenyl]-3-yl)(phenyl)methyl)-4-methylbenzenesulfonamide 11: 62.4 mg, 64% yield, 96% ee. White solid, mp:169-171 ℃. ^{1}H NMR (400 MHz, Chloroform-*d*) δ 7.56 (d, *J* = 8.3 Hz, 2H), 7.36 – 7.32 (m, 2H), 7.24 – 7.15 (m, 5H), 7.10 (d, *J* = 8.0 Hz, 2H), 6.92 – 6.88 (m, 2H), 6.81 – 6.79 (m, 2H), 5.53 (d, *J* = 7.1 Hz, 1H), 5.24 (d, *J* = 7.1 Hz, 1H), 3.84 (s, 3H), 3.30 (s, 3H), 2.33 (s, 3H), 2.17 (s, 3H); ^{13}C NMR (101 MHz, $CDCl_3$) δ 158.8, 155.1, 143.1, 140.5, 137.4, 135.7, 134.1, 131.7, 130.6, 130.0, 129.2, 128.7, 128.5, 127.5, 127.2, 127.2, 113.6, 61.0, 59.6, 55.2, 21.4, 16.2; HRMS (m/z): $[M+Na]^+$ calcd. for $C_{29}H_{29}NO_4SNa$, 510.1710; found, 510.1719.

The ee value was determined by HPLC analysis: Chiralcel OD-H Column, hexane/2-propanol= 90/10, 25 ℃, 1.0 mL/min, 254 nm, retention time: 9.9 min (major) and 16.2 min (minor).

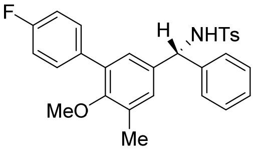

12

(R)-N-((4'-fluoro-6-methoxy-5-methyl-[1,1'-biphenyl]-3-yl)(phenyl)methyl)-4-methylbenzenesulfonamide 12: 79.7 mg, 84% yield, 95% ee. White solid, mp:141-142 ℃. ^{1}H NMR (400 MHz, Chloroform-*d*) δ 7.56 (d, *J* = 8.3 Hz, 2H), 7.40 – 7.35 (m, 2H), 7.24 – 7.19 (m, 3H), 7.18 – 7.14 (m, 2H), 7.10 (d, *J* = 8.0 Hz, 2H), 7.08 – 7.02 (m, 2H), 6.84 (d, *J* = 2.2 Hz, 1H), 6.83 (d, *J* = 2.3 Hz, 1H), 5.53 (d, *J* = 7.1 Hz, 1H), 5.34 (d, *J* = 7.2 Hz, 1H), 3.29 (s, 3H), 2.33 (s, 3H), 2.17 (s, 3H); ^{13}C NMR (101 MHz, $CDCl_3$) δ 162.1 (d, *J* (C, F)= 246.2 Hz), 155.1, 143.1, 140.5, 137.4, 135.9, 134.1 (d, *J* (C, F)= 3.4 Hz), 133.5, 131.8, 130.5 (d, *J* (C, F)= 8.0 Hz), 129.3, 129.2, 128.6, 127.6 ,127.5, 127.2, 127.2, 115.0 (d, *J* (C, F)= 21.1 Hz), 61.0, 59.7, 21.4, 16.2; HRMS (m/z): $[M+Na]^+$ calcd. for $C_{28}H_{26}FNO_3SNa$, 498.1510; found, 498.1504.

The ee value was determined by HPLC analysis: Chiralcel OD-H Column, hexane/2-propanol= 90/10, 25 ℃, 1.0 mL/min, 254 nm, retention time: 7.7 min (major) and 10.5 min (minor).

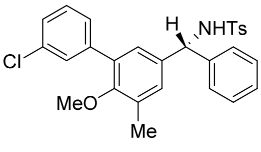

13

(R)-N-((3'-chloro-6-methoxy-5-methyl-[1,1'-biphenyl]-3-yl)(phenyl)methyl)-4-methylbenzenesulfonamide 13: 76.3 mg, 78% yield, 93% ee. White solid, mp:126-127 ℃. ^{1}H NMR (400 MHz, Chloroform-*d*) δ 7.55 (d, *J* = 7.5 Hz, 2H), 7.37 (s, 1H), 7.29 (s, 3H), 7.23 – 7.20 (m, 3H), 7.16 (d, *J* = 6.9 Hz, 2H), 7.10 (d, *J* = 7.7 Hz, 2H), 6.88 (s, 1H), 6.80 (s, 1H), 5.55 (d, *J* = 6.7 Hz, 1H),

5.36 (d, *J* = 6.8 Hz, 1H), 3.31 (s, 3H), 2.33 (s, 3H), 2.18 (s, 3H); ^{13}C NMR (101 MHz, $CDCl_3$) δ 155.1, 143.2, 140.5, 140.0, 137.4, 135.9, 133.9, 133.1, 131.9, 129.8, 129.4, 129.2, 128.9, 128.6, 127.6, 127.4, 127.2, 127.2, 127.1, 61.0, 60.0, 21.4, 16.2; HRMS (m/z): $[M+Na]^+$ calcd. for $C_{28}H_{26}ClNO_3SNa$, 514.1214; found, 514.1226.

The ee value was determined by HPLC analysis: Chiralcel OD-H Column, hexane/2-propanol= 90/10, 25 ℃, 1.0 mL/min, 254 nm, retention time: 7.7 min (major) and 10.5 min (minor).

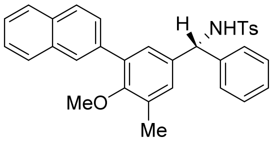

14

(R)-N-((4-methoxy-3-methyl-5-(naphthalen-2-yl)phenyl)(phenyl)methyl)-4-methylbenzenesulfonamide 14: 79.5 mg, 78% yield, 94% ee. White solid, mp:175-176 ℃. ^{1}H NMR (400 MHz, Chloroform-*d*) δ 7.85 – 7.82 (m, 4H), 7.59 – 7.56 (m, 3H), 7.50 – 7.48 (m, 2H), 7.22 – 7.18 (m, 5H), 7.08 (d, *J* = 8.0 Hz, 2H), 6.95 (s, 1H), 6.88 (s, 1H), 5.58 (d, *J* = 7.0 Hz, 1H), 5.33 (d, *J* = 7.0 Hz, 1H), 3.28 (s, 3H), 2.26 (s, 3H), 2.21 (s, 3H); ^{13}C NMR (101 MHz, $CDCl_3$) δ 155.4, 143.1, 140.5, 137.4, 135.9, 135.8, 134.4, 133.3, 132.4, 131.8, 129.4, 129.2, 128.6, 128.1, 127.9, 127.6, 127.5, 127.5, 127.3, 127.2, 126.0, 126.0, 61.1, 59.9, 21.4, 16.2; HRMS (m/z): $[M+Na]^+$ calcd. for $C_{32}H_{29}NO_3SNa$, 530.1760; found, 530.1772.

The ee value was determined by HPLC analysis: Chiralcel OD-H Column, hexane/2-propanol= 90/10, 25 ℃, 1.0 mL/min, 254 nm, retention time: 12.6 min (major) and 21.7 min (minor).

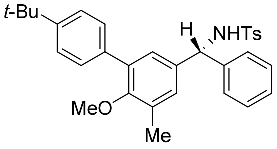

15

(R)-N-((4'-(tert-butyl)-6-methoxy-5-methyl-[1,1'-biphenyl]-3-yl)(phenyl)methyl)-4-methylbenzenesulfonamide 15: 71.0 mg, 69% yield, 94% ee. White solid, mp: 141-142 ℃. ^{1}H NMR (400 MHz, Chloroform-*d*) δ 7.56 (d, *J* = 8.3 Hz, 2H), 7.38 (d, *J* = 8.6 Hz, 2H), 7.33 (d, *J* = 8.6 Hz, 2H), 7.24 – 7.15 (m, 5H), 7.10 (d, *J* = 8.0 Hz, 2H), 6.82 (s, 2H), 5.52 (d, *J* = 7.1 Hz, 1H), 5.28 (d, *J* = 7.1 Hz, 1H), 3.31 (s, 3H), 2.32 (s, 3H), 2.17 (s, 3H), 1.35 (s, 9H); ^{13}C NMR (101 MHz, $CDCl_3$) δ 155.3, 150.0, 143.0, 140.5, 137.4, 135.6, 135.2, 134.4, 131.6, 129.2, 128.9, 128.5, 128.4, 127.8, 127.5, 127.2, 125.0, 61.0, 59.8, 34.5, 31.3, 21.4, 16.2; HRMS (m/z): $[M+Na]^+$ calcd. for $C_{23}H_{35}NO_3SNa$, 536.2230; found, 536.2235.

The ee value was determined by HPLC analysis: Chiralcel OD-H Column, hexane/2-propanol= 90/10, 25 ℃, 1.0 mL/min, 254 nm, retention time: 7.7 min (major) and 11.7 min (minor).

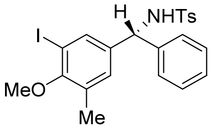

16

(R)-N-((3-iodo-4-methoxy-5-methylphenyl)(phenyl)methyl)-4-methylbenzenesulfonamide 16: 50.6 mg, 50% yield, 94% ee. White solid, mp:111-112 ℃. ^{1}H NMR (400 MHz, Chloroform-*d*) δ 7.53 (d, *J* = 7.7 Hz, 2H), 7.23 – 7.20 (m, 4H), 7.14 (d, *J* = 7.7 Hz, 2H), 7.11 – 7.09 (m, 2H), 6.84 (s, 1H), 5.45 (d, *J*

= 7.0 Hz, 1H), 5.29 (d, *J* = 6.8 Hz, 1H), 3.69 (s, 3H), 2.39 (s, 3H), 2.17 (s, 3H); ^{13}C NMR (101 MHz, $CDCl_3$) δ 157.3, 143.3, 140.0, 137.7, 137.2, 135.8, 132.1, 130.6, 129.4, 128.7, 127.8, 127.2, 127.2, 91.9, 60.3, 60.1, 21.6, 16.9; HRMS (m/z): $[M+Na]^+$ calcd. for $C_{22}H_{22}INO_3SNa$, 530.0257; found, 530.0269.
The ee value was determined by HPLC analysis: Chiralcel OD-H Column, hexane/2-propanol= 90/10, 25 ℃, 1.0 mL/min, 254 nm, retention time: 13.9 min (major) and 22.7 min (minor).

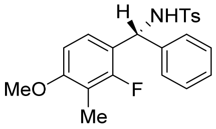

17

(R)-N-((2-fluoro-4-methoxy-3-methylphenyl)(phenyl)methyl)-4-methylbenzenesulfonamide 17: 48.9 mg, 61% yield, 99% ee. White solid, mp:91-92 ℃. ^{1}H NMR (400 MHz, Chloroform-*d*) δ 7.56 (d, *J* = 8.3 Hz, 2H), 7.24 – 7.16 (m, 5H), 7.10 (d, *J* = 8.0 Hz, 2H), 6.78 (d, *J* = 8.6 Hz, 1H), 6.35 (d, *J* = 12.3 Hz, 1H), 5.68 (d, *J* = 8.1 Hz, 1H), 5.29 (d, *J* = 8.0 Hz, 1H), 3.74 (s, 3H), 2.35 (s, 3H), 2.01 (s, 3H); ^{13}C NMR (101 MHz, $CDCl_3$) δ 158.7 (d, *J* (C, F) = 243.6 Hz), 158.0 (d, *J* (C, F) = 9.8 Hz), 143.0, 140.0, 137.3, 130.1 (d , *J* (C, F) = 5.7 Hz), 129.2, 128.5, 127.6, 127.1, 126.8, 122.1 (d, *J* (C, F) = 3.3 Hz), 117.9 (d, *J* (C, F) = 13.1 Hz), 98.4 (d, *J* (C, F) = 25.9 Hz), 56.1 (d, *J* (C, F) = 1.3 Hz), 55.5, 21.4, 15.4; HRMS (m/z): $[M+Na]^+$ calcd. for $C_{22}H_{22}FNO_3SNa$, 422.1197; found, 422.1199.
The ee value was determined by HPLC analysis: Chiralcel OD-H Column, hexane/2-propanol= 90/10, 25 ℃, 1.0 mL/min, 254 nm, retention time: 11.9 min (major) and 17.1 min (minor).

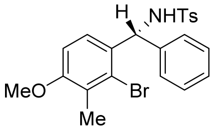

18a

(R)-N-((2-bromo-4-methoxy-3-methylphenyl)(phenyl)methyl)-4-methylbenzenesulfonamide 18a: 32.8 mg, 36% yield, 96% ee. White solid, mp:124-125 ℃. ^{1}H NMR (400 MHz, Chloroform-*d*) δ 7.61 (d, *J* = 7.6 Hz, 2H), 7.22 – 7.20 (m, 3H), 7.16 – 7.13 (m, 3H), 7.09 – 7.06 (m, 2H), 6.69 (d, *J* = 8.5 Hz, 1H), 5.90 (d, *J* = 6.8 Hz, 1H), 5.32 (d, *J* = 6.2 Hz, 1H), 3.79 (s, 3H), 2.38 (s, 3H), 2.22 (s, 3H); ^{13}C NMR (101 MHz, $CDCl_3$) δ 157.5, 143.1, 139.9, 137.0, 131.1, 129.3, 128.5, 127.6, 127.5, 127.4, 127.3, 127.3, 126.6, 108.9, 61.1, 55.8, 21.5, 16.2; HRMS (m/z): $[M+Na]^+$ calcd. for $C_{22}H_{22}BrNO_3SNa$, 482.0396; found, 482.0404.
The ee value was determined by HPLC analysis: Chiralcel OD-H Column, hexane/2-propanol= 90/10, 25 ℃, 1.0 mL/min, 254 nm, retention time: 15.6 min (minor) and 17.6 min (major).

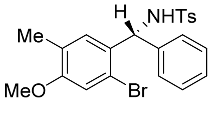

18b

(R)-N-((2-bromo-4-methoxy-5-methylphenyl)(phenyl)methyl)-4-methylbenzenesulfonamide 18b: 20.3 mg, 22% yield, 92% ee. White solid, mp:57-58 ℃. ^{1}H NMR (400 MHz, Chloroform-*d*) δ 7.59 (d, *J* = 7.3 Hz, 2H), 7.24 – 7.19 (m, 3H), 7.13 – 7.12 (m, 4H), 6.92 (s, 1H), 6.82 (s, 1H), 5.86 (d, *J* = 6.9 Hz, 1H), 5.36 (d, *J* = 6.6 Hz, 1H), 3.76 (s, 3H), 2.37 (s, 3H), 2.00 (s, 3H); ^{13}C NMR (101 MHz, $CDCl_3$) δ 157.4, 143.1, 139.8, 137.1, 131.0, 130.2, 129.2, 128.5, 127.6, 127.2, 126.3, 120.0, 114.3, 60.0, 55.6, 21.4, 15.8; HRMS (m/z): $[M+Na]^+$ calcd. for $C_{22}H_{22}BrNO_3SNa$, 482.0396; found, 482.0403.
The ee value was determined by HPLC analysis: Chiralcel OD-H Column, hexane/2-propanol= 90/10, 25 ℃, 1.0 mL/min, 214 nm, retention time: 10.7 min (major) and 15.6 min (minor).

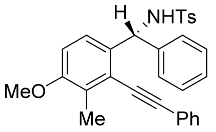

19a

(R)-N-((4-methoxy-3-methyl-2-(phenylethynyl)phenyl)(phenyl)methyl)-4-methylbenzenesulfonamide 19a: 33.4 mg, 35% yield, 95% ee. White solid, mp:153-154 ℃. ^{1}H NMR (400 MHz, Chloroform-*d*) δ 7.55 (d, *J* = 7.6 Hz, 2H), 7.34 – 7.28 (m, 5H), 7.22 – 7.17 (m, 5H), 7.06 – 7.02 (m, 3H), 6.68 (d, *J* = 8.4 Hz, 1H), 5.94 (d, *J* = 7.5 Hz, 1H), 5.61 (d, *J* = 7.4 Hz, 1H), 3.79 (s, 3H), 2.31 (s, 3H), 2.27 (s, 3H); ^{13}C NMR (101 MHz, $CDCl_3$) δ 156.8, 142.8, 140.6, 137.1, 133.6, 131.3, 129.6, 129.0, 128.5, 128.3, 127.3, 127.2, 127.1, 126.4, 122.8, 122.4, 110.0, 99.1, 86.2, 60.0, 55.5, 21.3, 13.9; HRMS (m/z): $[M+Na]^+$ calcd. for $C_{30}H_{27}NO_3SNa$, 504.1604; found, 504.1626.

The ee value was determined by HPLC analysis: Chiralcel OD-H Column, hexane/2-propanol= 90/10, 25 ℃, 1.0 mL/min, 254 nm, retention time: 16.4 min (minor) and 18.5 min (major).

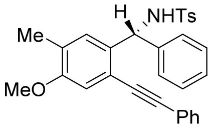

19b

(R)-N-((4-methoxy-5-methyl-2-(phenylethynyl)phenyl)(phenyl)methyl)-4-methylbenzenesulfonamide 19b: 22.4 mg, 23% yield, 97% ee. White solid, mp:61-62 ℃. ^{1}H NMR (400 MHz, Chloroform-*d*) δ 7.56 (d, *J* = 7.4 Hz, 2H), 7.34 – 7.29 (m, 5H), 7.25 – 7.19 (m, 5H), 7.05 (d, *J* = 7.6 Hz, 2H), 6.87 (s, 1H), 6.80 (s, 1H), 5.90 (d, *J* = 7.4 Hz, 1H), 5.49 (d, *J* = 7.3 Hz, 1H), 3.80 (s, 3H), 2.30 (s, 3H), 2.08 (s, 3H); ^{13}C NMR (101 MHz, $CDCl_3$) δ 156.6, 142.9, 140.6, 137.1, 133.8, 131.4, 130.3, 129.1, 128.5, 128.4, 128.3, 127.9, 127.4, 127.3, 127.0, 122.7, 119.5, 113.4, 94.3, 87.5, 59.5, 55.4, 21.4, 16.2; HRMS (m/z): $[M+Na]^+$ calcd. for $C_{30}H_{27}NO_3SNa$, 504.1604; found, 504.1610.

The ee value was determined by HPLC analysis: Chiralpak IB Column, hexane/2-propanol= 90/10, 25 ℃, 1.0 mL/min, 214 nm, retention time: 11.0 min (major) and 12.5 min (minor).

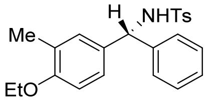

20

(R)-N-((4-ethoxy-3-methylphenyl)(phenyl)methyl)-4-methylbenzenesulfonamide 20: 52.7 mg, 67% yield, 94% ee. White solid, mp:89-90 ℃. ^{1}H NMR (400 MHz, Chloroform-*d*) δ 7.54 (d, *J* = 7.4 Hz, 2H), 7.22 – 7.18 (m, 3H), 7.16 – 7.11 (m, 4H), 6.81 (d, *J* = 8.3 Hz, 1H), 6.75 (s, 1H), 6.60 (d, *J* = 8.3 Hz, 1H), 5.47 (d, *J* = 6.9 Hz, 1H), 5.13 (d, *J* = 6.9 Hz, 1H), 3.95 (q, *J* = 6.9 Hz, 2H), 2.37 (s, 3H), 2.05 (s, 3H), 1.38 (t, *J* = 6.9 Hz, 3H); ^{13}C NMR (101 MHz, $CDCl_3$) δ 156.5, 143.0, 140.8, 137.4, 131.9, 129.7, 129.2, 128.4, 127.3, 127.2, 127.2, 126.9, 125.8, 110.7, 63.5, 60.9, 21.4, 16.1, 14.9; HRMS (m/z): $[M+Na]^+$ calcd. for $C_{23}H_{25}NO_3SNa$, 418.1447; found, 418.1459.

The ee value was determined by HPLC analysis: Chiralcel OD-H Column, hexane/2-propanol= 90/10, 25 ℃, 1.0 mL/min, 214 nm, retention time: 13.3 min (minor) and 22.6 min (major).

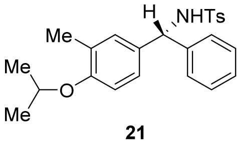

21

(R)-N-((4-isopropoxy-3-methylphenyl)(phenyl)methyl)-4-methylbenzenesulfonamide 21: 39.3 mg, 48% yield, 95% ee. White solid, mp:117-118 ℃. ^{1}H NMR (400 MHz, Chloroform-*d*) δ 7.54 (d, *J* = 7.7 Hz, 2H), 7.20 – 7.19 (m, 3H), 7.15 – 7.10 (m, 4H), 6.79 (d, *J* = 8.3 Hz, 1H), 6.74 (s, 1H), 6.62 (d, *J* = 8.3 Hz, 1H), 5.48 (d, *J* = 6.9 Hz, 1H), 5.15 (d, *J* = 6.8 Hz, 1H), 4.43 (hept, *J* = 7.6 Hz, 1H), 2.36 (s, 3H), 2.03 (s, 3H), 1.30 (s, 3H), 1.28 (s, 3H); ^{13}C NMR (101 MHz, $CDCl_3$) δ 155.5, 142.9, 140.8, 137.5, 131.8, 129.9, 129.2, 128.4, 127.8, 127.3, 127.3, 127.2, 125.7, 112.5, 70.1, 60.9, 22.1, 21.4, 16.3; HRMS (m/z): $[M+Na]^+$ calcd. for $C_{24}H_{27}NO_3SNa$, 432.1604; found, 432.1616.
The ee value was determined by HPLC analysis: Chiralcel OD-H Column, hexane/2-propanol= 90/10, 25 ℃, 1.0 mL/min, 214 nm, retention time: 12.1 min (minor) and 20.3 min (major).

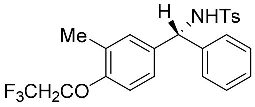

22

(R)-4-methyl-N-((3-methyl-4-(2,2,2-trifluoroethoxy)phenyl)(phenyl)methyl)benzenesulfonamide 22: 54.6 mg, 61% yield, 94% ee. White solid, mp:120-121 ℃. ^{1}H NMR (400 MHz, Chloroform-*d*) δ 7.54 (d, *J* = 7.7 Hz, 2H), 7.21 – 7.20 (m, 3H), 7.14 – 7.08 (m, 4H), 6.90 (d, *J* = 8.3 Hz, 1H), 6.83 (s, 1H), 6.60 (d, *J* = 8.3 Hz, 1H), 5.49 (d, *J* = 6.9 Hz, 1H), 5.20 (d, *J* = 6.6 Hz, 1H), 4.28 (q, *J* = 7.9 Hz, 2H), 2.37 (s, 3H), 2.09 (s, 3H); ^{13}C NMR (101 MHz, $CDCl_3$) δ 154.8, 143.1, 140.6, 137.4, 134.3, 130.2, 129.2, 128.5, 127.6, 127.4, 127.2, 127.1, 125.9, 123.3 (q, *J* (C, F) = 282.0 Hz), 111.5, 66.0 (q, *J* (C, F) = 35.5 Hz), 60.7, 21.3, 15.8; HRMS (m/z): $[M+Na]^+$ calcd. for $C_{23}H_{22}F_3NO_3SNa$, 472.1165; found, 472.1178.
The ee value was determined by HPLC analysis: Chiralpak IC Column, hexane/2-propanol= 80/20, 25 ℃, 1.0 mL/min, 254 nm, retention time: 11.9 min (minor) and 13.6 min (major).

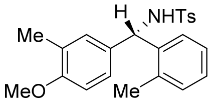

23

(S)-N-((4-methoxy-3-methylphenyl)(o-tolyl)methyl)-4-methylbenzenesulfonamide 23: 61.5 mg, 78% yield, 96% ee. White solid, mp:107-108 ℃. ^{1}H NMR (400 MHz, Chloroform-*d*) δ 7.54 (d, *J* = 7.9 Hz, 2H), 7.18 – 7.04 (m, 6H), 6.77 (d, *J* = 8.3 Hz, 2H), 6.73 (s, 1H), 6.60 (d, *J* = 8.3 Hz, 2H), 5.69 (d, *J* = 6.6 Hz, 1H), 5.00 (d, *J* = 6.3 Hz, 1H), 3.75 (s, 3H), 2.37 (s, 3H), 2.15 (s, 3H), 2.04 (s, 3H); ^{13}C NMR (101 MHz, $CDCl_3$) δ 157.1, 143.0, 138.5, 137.6, 135.3, 131.4, 130.5, 129.9, 129.2, 127.3, 127.1, 127.0, 126.7, 126.0, 109.7, 57.7, 55.3, 21.4, 19.3, 16.1; HRMS (m/z): $[M+Na]^+$ calcd. for $C_{23}H_{25}NO_3SNa$, 418.1447; found, 418.1448.
The ee value was determined by HPLC analysis: Chiralcel OD-H Column, hexane/2-propanol= 90/10, 25 ℃, 1.0 mL/min, 214 nm, retention time: 9.8 min (minor) and 18.9 min (major).

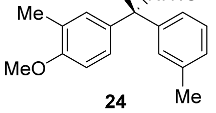

24

(R)-N-((4-methoxy-3-methylphenyl)(m-tolyl)methyl)-4-methylbenzenesulfonamide 24: 63.4 mg, 80% yield, 94% ee. White solid, mp:113-114 ℃. ^{1}H NMR (400 MHz, Chloroform-*d*) δ 7.54 (d, *J* = 7.5 Hz, 2H), 7.13 (d, *J* = 8.1 Hz, 2H), 7.08 (d, *J* = 7.3 Hz, 1H), 6.98 (d, *J* = 7.4 Hz, 1H), 6.91 – 6.85 (m, 3H), 6.77 (s, 1H), 6.63 (d, *J* = 8.2 Hz, 1H), 5.44 (d, *J* = 6.6 Hz, 1H), 5.05 (d, *J* = 6.3 Hz, 1H), 3.76 (s, 3H), 2.38 (s, 3H), 2.21 (s, 3H), 2.06 (s, 3H); ^{13}C NMR (101 MHz, $CDCl_3$) δ 157.1, 142.9, 140.7, 138.0, 137.6,

132.3, 129.7, 129.2, 128.3, 128.1, 127.9, 127.2, 126.7, 125.8, 124.3, 109.7, 60.9, 55.3, 21.4, 21.3, 16.1; HRMS (m/z): $[M+Na]^+$ calcd. for $C_{23}H_{25}NO_3SNa$, 418.1447; found, 418.1444.
The ee value was determined by HPLC analysis: Chiralcel OD-H Column, hexane/2-propanol= 90/10, 25 ℃, 1.0 mL/min, 214 nm, retention time: 14.2 min (minor) and 23.9 min (major).

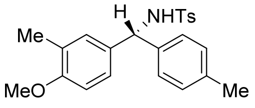

25

(R)-N-((4-methoxy-3-methylphenyl)(p-tolyl)methyl)-4-methylbenzenesulfonamide 25: 62.1 mg, 79% yield, 94% ee. White solid, mp:104-105 ℃. ^{1}H NMR (400 MHz, Chloroform-*d*) δ 7.54 (d, *J* = 8.1 Hz, 2H), 7.12 (d, *J* = 8.1 Hz, 2H), 7.00 (s, 4H), 6.86 (d, *J* = 8.4 Hz, 1H), 6.76 (s, 1H), 6.62 (d, *J* = 8.4 Hz, 1H), 5.44 (d, *J* = 7.0 Hz, 1H), 5.12 (d, *J* = 6.9 Hz, 1H), 3.75 (s, 3H), 2.37 (s, 3H), 2.27 (s, 3H), 2.05 (s, 3H); ^{13}C NMR (101 MHz, $CDCl_3$) δ 157.1, 142.9, 138.0, 137.5, 137.0, 132.3, 129.6, 129.2, 129.1, 127.2, 127.2, 126.6, 125.8, 109.7, 60.7, 55.3, 21.40, 21.0, 16.1; HRMS (m/z): $[M+Na]^+$ calcd. for $C_{23}H_{25}NO_3SNa$, 418.1447; found, 418.1450.
The ee value was determined by HPLC analysis: Chiralcel OD-H Column, hexane/2-propanol= 80/20, 25 ℃, 1.0 mL/min, 254 nm, retention time: 11.6 min (major) and 13.3 min (minor).

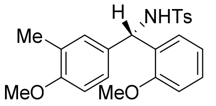

26

(S)-N-((4-methoxy-3-methylphenyl)(2-methoxyphenyl)methyl)-4-methylbenzenesulfonamide 26: 66.9 mg, 81% yield, 96% ee. White solid, mp:128-129 ℃. ^{1}H NMR (400 MHz, Chloroform-*d*) δ 7.50 (d, *J* = 7.9 Hz, 2H), 7.13 (t, *J* = 7.5 Hz, 1H), 7.04 (d, *J* = 7.7 Hz, 2H), 6.99 (d, *J* = 7.4 Hz, 1H), 6.93 – 6.89 (m, 2H), 6.77 (t, *J* = 7.2 Hz, 1H), 6.67 – 6.62 (m, 2H), 5.74 (d, *J* = 8.7 Hz, 1H), 5.61 (d, *J* = 8.8 Hz, 1H), 3.75 (s, 3H), 3.60 (s, 3H), 2.32 (s, 3H), 2.09 (s, 3H); ^{13}C NMR (101 MHz, $CDCl_3$) δ 156.8, 156.3, 142.6, 137.5, 132.0, 129.4, 129.3, 128.9, 128.7, 127.8, 127.0, 126.2, 125.2, 120.6, 110.9, 109.3, 58.4, 55.2, 55.2, 21.4, 16.2; HRMS (m/z): $[M+Na]^+$ calcd. for $C_{23}H_{25}NO_4SNa$, 434.1397; found, 434.1392.
The ee value was determined by HPLC analysis: Chiralcel OD-H Column, hexane/2-propanol= 90/10, 25 ℃, 1.0 mL/min, 254 nm, retention time: 14.9 min (minor) and 26.0 min (major).

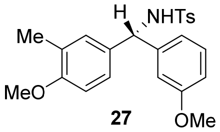

27

(S)-N-((4-methoxy-3-methylphenyl)(3-methoxyphenyl)methyl)-4-methylbenzenesulfonamide 27: 65.2 mg, 79% yield, 93% ee. White solid, mp:105-106 ℃. ^{1}H NMR (400 MHz, Chloroform-*d*) δ 7.55 (d, *J* = 7.7 Hz, 2H), 7.14 – 7.10 (m, 3H), 6.86 (d, *J* = 8.3 Hz, 1H), 6.77 (s, 1H), 6.71 (t, *J* = 5.9 Hz, 2H), 6.64 – 6.61 (m, 2H), 5.44 (d, *J* = 6.8 Hz, 1H), 5.12 (d, *J* = 6.6 Hz, 1H), 3.76 (s, 3H), 3.69 (s, 3H), 2.37 (s, 3H), 2.05 (s, 3H); ^{13}C NMR (101 MHz, $CDCl_3$) δ 159.6, 157.1, 143.0, 142.4, 137.5, 132.1, 129.6, 129.4, 129.2, 127.2, 126.7, 125.8, 119.6, 112.9, 109.7, 60.9, 55.3, 55.1, 21.4, 16.1; HRMS (m/z): $[M+Na]^+$ calcd. for $C_{23}H_{25}NO_4SNa$, 434.1397; found, 434.1395.
The ee value was determined by HPLC analysis: Chiralcel OD-H Column, hexane/2-propanol= 90/10, 25 ℃, 1.0 mL/min, 254 nm, retention time: 20.2 min (minor) and 28.3 min (major).

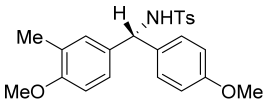

28

(R)-N-((4-methoxy-3-methylphenyl)(4-methoxyphenyl)methyl)-4-methylbenzenesulfonamide 28: 47.7 mg, 58% yield, 93% ee. White solid, mp:94-95 ℃. ^{1}H NMR (400 MHz, Chloroform-*d*) δ 7.54 (d, *J* = 8.0 Hz, 2H), 7.13 (d, *J* = 8.0 Hz, 2H), 7.02 (d, *J* = 8.5 Hz, 2H), 6.85 (d, *J* = 8.2 Hz, 1H), 6.76 (s, 1H), 6.73 (d, *J* = 8.5 Hz, 2H), 6.63 (d, *J* = 8.4 Hz, 1H), 5.43 (d, *J* = 6.7 Hz, 1H), 5.05 (d, *J* = 6.8 Hz, 1H), 3.76 (s, 3H), 3.75 (s, 3H), 2.38 (s, 3H), 2.05 (s, 3H); ^{13}C NMR (101 MHz, $CDCl_3$) δ 158.9, 157.1, 142.9, 137.6, 133.1, 132.4, 129.6, 129.2, 128.5, 127.2, 126.7, 125.7, 113.8, 109.7, 60.4, 55.3, 55.2, 21.4, 16.1; HRMS (m/z): $[M+Na]^+$ calcd. for $C_{23}H_{25}NO_4SNa$, 434.1397; found, 434.1397.

The ee value was determined by HPLC analysis: Chiralcel OD-H Column, hexane/2-propanol= 80/20, 25 ℃, 1.0 mL/min, 254 nm, retention time: 16.2 min (major) and 21.8 min (minor).

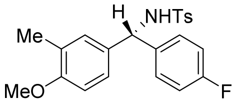

29

(R)-N-((4-fluorophenyl)(4-methoxy-3-methylphenyl)methyl)-4-methylbenzenesulfonamide 29: 66.4 mg, 84% yield, >99% ee. White solid, mp:116-117 ℃. ^{1}H NMR (400 MHz, Chloroform-*d*) δ 7.54 (d, *J* = 8.3 Hz, 2H), 7.14 – 7.09 (m, 4H), 6.88 (t, *J* = 8.7 Hz, 2H), 6.81 (dd, *J* = 8.4, 2.2 Hz, 1H), 6.72 (d, *J* = 1.8 Hz, 1H), 6.62 (d, *J* = 8.4 Hz, 1H), 5.45 (d, *J* = 7.1 Hz, 1H), 5.30 (d, *J* = 7.1 Hz, 1H), 3.76 (s, 3H), 2.38 (s, 3H), 2.05 (s, 3H); ^{13}C NMR (101 MHz, $CDCl_3$) δ 161.9 (d, *J* (C, F) = 246.2 Hz), 157.2, 143.2, 137.3, 136.6 (d, *J* (C, F) = 3.2 Hz), 131.9, 129.6, 129.3, 128.9 (d, *J* (C, F) = 8.3 Hz), 127.2, 126.8, 125.7, 115.2 (d, *J* (C, F) = 21.5 Hz), 109.7, 60.2, 55.3, 21.4, 16.1; HRMS (m/z): $[M+Na]^+$ calcd. for $C_{22}H_{22}FNO_3SNa$, 422.1197; found, 422.1207.

The ee value was determined by HPLC analysis: Chiralcel OD-H Column, hexane/2-propanol= 90/10, 25 ℃, 1.0 mL/min, 214 nm, retention time: 22.3 min (minor) and 26.7 min (major).

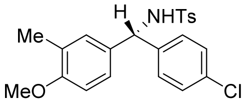

30

(R)-N-((4-chlorophenyl)(4-methoxy-3-methylphenyl)methyl)-4-methylbenzenesulfonamide 30: 64.5 mg, 78% yield, 94% ee. White solid, mp:118-119 ℃. ^{1}H NMR (400 MHz, Chloroform-*d*) δ 7.54 (d, *J* = 8.2 Hz, 2H), 7.18 – 7.14 (m, 4H), 7.08 (d, *J* = 8.4 Hz, 2H), 6.79 (dd, *J* = 8.4, 1.8 Hz, 1H), 6.70 (d, *J* = 1.8 Hz, 1H), 6.63 (d, *J* = 8.4 Hz, 1H), 5.44 (d, *J* = 6.9 Hz, 1H), 5.10 (d, *J* = 6.9 Hz, 1H), 3.76 (s, 3H), 2.40 (s, 3H), 2.05 (s, 3H); ^{13}C NMR (101 MHz, $CDCl_3$) δ 157.3, 143.3, 139.3, 137.2, 133.2, 131.7, 129.6, 129.3, 128.7, 128.5, 127.2, 127.0, 125.8, 109.8, 60.3, 55.3, 21.5, 16.1; HRMS (m/z): $[M+Na]^+$ calcd. for $C_{22}H_{22}ClNO_3SNa$, 438.0901; found, 438.0901.

The ee value was determined by HPLC analysis: Chiralpak IB Column, hexane/2-propanol= 90/10, 25 ℃, 1.0 mL/min, 254 nm, retention time: 16.4 min (minor) and 18.5 min (major).

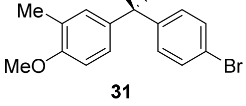

31

(R)-N-((4-bromophenyl)(4-methoxy-3-methylphenyl)methyl)-4-methylbenzenesulfonamide 31: 71.3 mg, 78% yield, 95% ee. White solid, mp:124-125 ℃. ^{1}H NMR (400 MHz, Chloroform-*d*) δ 7.54 (d, *J* = 7.8 Hz, 2H), 7.32 (d, *J* = 8.0 Hz, 2H), 7.15 (d, *J* = 7.7 Hz, 2H), 7.02 (d, *J* = 7.9 Hz, 2H), 6.79 (d, *J* = 8.3 Hz, 1H), 6.70 (s, 1H), 6.62 (d, *J* = 8.3 Hz, 1H), 5.42 (d, *J* = 6.8 Hz, 1H), 5.16 (d, *J* = 7.0 Hz, 1H), 3.76 (s, 3H), 2.40 (s, 3H), 2.05 (s, 3H); ^{13}C NMR (101 MHz, $CDCl_3$) δ 157.3, 143.3, 139.9, 137.3, 131.7, 131.4, 129.6, 129.3, 129.0, 127.2, 127.0, 125.8, 121.3, 109.8, 60.4, 55.3, 21.4, 16.1; HRMS (m/z): $[M+Na]^+$ calcd. for $C_{22}H_{22}BrNO_3SNa$, 482.0396; found, 482.0390.
The ee value was determined by HPLC analysis: Chiralpak IB Column, hexane/2-propanol= 90/10, 25 ℃, 1.0 mL/min, 254 nm, retention time: 17.1 min (minor) and 18.8 min (major).

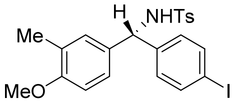

32

(R)-N-((4-iodophenyl)(4-methoxy-3-methylphenyl)methyl)-4-methylbenzenesulfonamide 32: 74.0 mg, 73% yield, 97% ee. White solid, mp:130-131 ℃. ^{1}H NMR (400 MHz, Chloroform-*d*) δ 7.53 – 7.50 (m, 4H), 7.14 (d, *J* = 7.6 Hz, 2H), 6.89 (d, *J* = 7.8 Hz, 2H), 6.79 (d, *J* = 8.3 Hz, 1H), 6.71 (s, 1H), 6.62 (d, *J* = 8.3 Hz, 1H), 5.40 (d, *J* = 6.8 Hz, 1H), 5.23 (d, *J* = 6.6 Hz, 1H), 3.76 (s, 3H), 2.40 (s, 3H), 2.05 (s, 3H); ^{13}C NMR (101 MHz, $CDCl_3$) δ 157.3, 143.2, 140.5, 137.4, 137.3, 131.6, 129.6, 129.3, 127.2, 126.9, 125.7, 109.8, 92.8, 60.5, 55.3, 21.5, 16.1; HRMS (m/z): $[M+Na]^+$ calcd. for $C_{22}H_{22}INO_3SNa$, 530.0257; found, 530.0266.
The ee value was determined by HPLC analysis: Chiralcel OD-H Column, hexane/2-propanol= 80/20, 25 ℃, 1.0 mL/min, 254 nm, retention time: 15.9 min (major) and 18.4 min (minor).

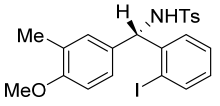

33

(S)-N-((2-iodophenyl)(4-methoxy-3-methylphenyl)methyl)-4-methylbenzenesulfonamide 33: 68.9 mg, 68% yield, 93% ee. White solid, mp:154-155 ℃. ^{1}H NMR (400 MHz, Chloroform-*d*) δ 7.71 (dd, *J* = 7.9, 0.9 Hz, 1H), 7.62 (d, *J* = 8.2 Hz, 2H), 7.38 (dd, *J* = 7.8, 1.4 Hz, 1H), 7.21 (t, *J* = 7.5 Hz, 1H), 7.16 (d, *J* = 8.1 Hz, 2H), 6.87 (td, *J* = 7.7, 1.5 Hz, 1H), 6.78 – 6.75 (m, 2H), 6.60 (d, *J* = 8.1 Hz, 1H), 5.70 (d, *J* = 6.5 Hz, 1H), 5.44 (d, *J* = 6.5 Hz, 1H), 3.73 (s, 3H), 2.37 (s, 3H), 2.05 (s, 3H);^{13}C NMR (101 MHz, $CDCl_3$) δ 157.2, 143.2, 142.3, 139.6, 136.9, 130.6, 130.1, 129.3, 128.9, 128.8, 128.3, 127.3, 126.8, 126.3, 109.6, 99.0, 64.2, 55.2, 21.4, 16.2; HRMS (m/z): $[M+Na]^+$ calcd. for $C_{22}H_{22}INO_3SNa$, 530.0257; found, 530.0269.
The ee value was determined by HPLC analysis: Chiralcel OD-H Column, hexane/2-propanol= 90/10, 25 ℃, 1.0 mL/min, 254 nm, retention time: 15.0 min (minor) and 21.8 min (major).

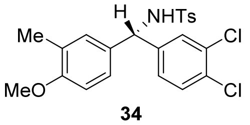

34

(S)-N-((3,4-dichlorophenyl)(4-methoxy-3-methylphenyl)methyl)-4-methylbenzenesulfonamide 34: 71.0 mg, 79% yield, 96% ee. White solid, mp:126-127 ℃. ^{1}H NMR (400 MHz, Chloroform-*d*) δ 7.54 (d, *J* = 7.6 Hz, 2H), 7.26 (d, *J* = 6.2 Hz, 1H), 7.16 – 7.15 (m, 3H), 7.02 (d, *J* = 8.2 Hz, 1H), 6.78 (d, *J* = 8.3 Hz, 1H), 6.70 (s, 1H), 6.63 (d, *J* = 8.3 Hz, 1H), 5.41 (d, *J* = 6.8 Hz, 1H), 5.34 (d, *J* = 6.6 Hz, 1H), 3.76

(s, 3H), 2.40 (s, 3H), 2.06 (s, 3H); ^{13}C NMR (101 MHz, $CDCl_3$) δ 157.5, 143.6, 140.9, 137.0, 132.4, 131.4, 131.1, 130.3, 129.5, 129.4, 129.3, 127.3, 127.2, 126.7, 125.7, 109.9, 60.0, 55.3, 21.5, 16.1; HRMS (m/z): $[M+Na]^+$ calcd. for $C_{22}H_{21}Cl_2NO_3SNa$, 472.0511; found, 472.0528.

The ee value was determined by HPLC analysis: Chiralpak IB Column, hexane/2-propanol= 90/10, 25 ℃, 1.0 mL/min, 254 nm, retention time: 18.4 min (minor) and 27.8 min (major).

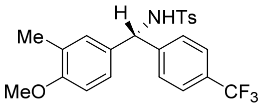

35

(R)-N-((4-methoxy-3-methylphenyl)(4-(trifluoromethyl)phenyl)methyl)-4-methylbenzenesulfonamide 35: 68.0 mg, 76% yield, 92% ee. White solid, mp:134-135 ℃. ^{1}H NMR (400 MHz, Chloroform-*d*) δ 7.53 (d, *J* = 8.2 Hz, 2H), 7.43 (d, *J* = 8.2 Hz, 2H), 7.28 (d, *J* = 8.1 Hz, 2H), 7.12 (d, *J* = 8.1 Hz, 2H), 6.79 (dd, *J* = 8.4, 2.4 Hz, 1H), 6.72 (d, *J* = 2.4 Hz, 1H), 6.63 (d, *J* = 8.4 Hz, 1H), 5.51 (d, *J* = 7.0 Hz, 1H), 5.32 (d, *J* = 7.0 Hz, 1H), 3.76 (s, 3H), 2.37 (s, 3H), 2.06 (s, 3H); ^{13}C NMR (101 MHz, $CDCl_3$) δ 157.3, 144.6, 143.2, 137.0, 131.3, 129.5, 129.3 (q, *J* (C, F) = 32.3 Hz), 129.2, 127.5, 127.1, 127.0, 125.7, 125.2 (q, *J* (C, F) = 3.4 Hz), 123.9 (q, *J* (C, F) = 272.1 Hz), 109.7, 60.5, 55.2, 21.3, 16.0; HRMS (m/z): $[M+Na]^+$ calcd. for $C_{23}H_{22}F_3NO_3SNa$, 472.1165; found, 472.1164.

The ee value was determined by HPLC analysis: Chiralpak IB Column, hexane/2-propanol= 90/10, 25 ℃, 1.0 mL/min, 254 nm, retention time: 21.6 min (minor) and 22.9 min (major).

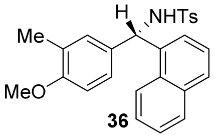

36

(S)-N-((4-methoxy-3-methylphenyl)(naphthalen-1-yl)methyl)-4-methylbenzenesulfonamide 36: 63.8 mg, 74% yield, 97% ee. White solid, mp:123-124 ℃. ^{1}H NMR (400 MHz, Chloroform-*d*) δ 7.81 (t, *J* = 7.8 Hz, 2H), 7.71 (d, *J* = 7.7 Hz, 1H), 7.50 (d, *J* = 7.6 Hz, 2H), 7.45 – 7.36 (m, 2H), 7.33 – 7.25 (m, 2H), 7.06 (d, *J* = 7.6 Hz, 2H), 6.85 – 6.80 (m, 2H), 6.59 (d, *J* = 8.2 Hz, 1H), 6.23 (d, *J* = 6.6 Hz, 1H), 5.14 (d, *J* = 6.4 Hz, 1H), 3.74 (s, 3H), 2.35 (s, 3H), 2.04 (s, 3H); ^{13}C NMR (101 MHz, $CDCl_3$) δ 157.2, 142.9, 137.4, 135.8, 133.9, 131.7, 130.5, 129.8, 129.1, 128.7, 128.3, 127.2, 126.8, 126.4, 126.0, 125.8, 125.6, 125.0, 123.5, 109.7, 58.0, 55.3, 21.4, 16.1; HRMS (m/z): $[M+Na]^+$ calcd. for $C_{26}H_{25}NO_3SNa$, 454.1447; found, 454.1441.

The ee value was determined by HPLC analysis: Chiralpak IB Column, hexane/2-propanol= 90/10, 25 ℃, 1.0 mL/min, 254 nm, retention time: 18.5 min (minor) and 22.4 min (major).

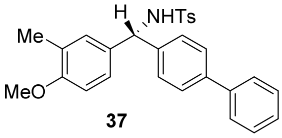

37

(R)-N-([1,1'-biphenyl]-4-yl(4-methoxy-3-methylphenyl)methyl)-4-methylbenzenesulfonamide 37: 74.5 mg, 82% yield, 94% ee. White solid, mp:152-153 ℃. ^{1}H NMR (400 MHz, Chloroform-*d*) δ 7.56 (d, *J* = 8.3 Hz, 2H), 7.53 – 7.50 (m, 2H), 7.43 – 7.39 (m, 4H), 7.33 (t, *J* = 7.3 Hz, 1H), 7.20 (d, *J* = 8.1 Hz, 2H), 7.12 (d, *J* = 8.0 Hz, 2H), 6.89 (dd, *J* = 8.4, 2.3 Hz, 1H), 6.80 (d, *J* = 2.1 Hz, 1H), 6.64 (d, *J* = 8.4 Hz, 1H), 5.52 (d, *J* = 7.0 Hz, 1H), 5.20 (d, *J* = 7.0 Hz, 1H), 3.76 (s, 3H), 2.35 (s, 3H), 2.07 (s, 3H); ^{13}C NMR (101 MHz, $CDCl_3$) δ 157.2, 143.0, 140.6, 140.2, 139.8, 137.5, 132.1, 129.7, 129.2, 128.7, 127.7,

127.3, 127.2, 127.1, 127.0, 126.8, 125.8, 109.8, 60.7, 55.3, 21.4, 16.1; HRMS (m/z): $[M+Na]^+$ calcd. for $C_{28}H_{27}NO_3SNa$, 480.1604; found, 480.1622.

The ee value was determined by HPLC analysis: Chiralcel OD-H Column, hexane/2-propanol= 75/25, 25 ℃, 1.0 mL/min, 214 nm, retention time: 13.9 min (major) and 33.1 min (minor).

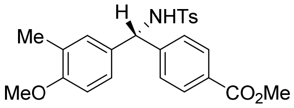

38

Methyl (R)-4-((4-methoxy-3-methylphenyl)((4-methylphenyl)sulfonamido)methyl)benzoate 38: 40.0 mg, 46% yield, 88% ee. White solid, mp:116-117 ℃. ^{1}H NMR (400 MHz, Chloroform-*d*) δ 7.88 (d, *J* = 7.9 Hz, 2H), 7.56(d, *J* = 7.7 Hz, 2H), 7.24 (d, *J* = 7.9 Hz, 2H), 7.15 (d, *J* = 7.7 Hz, 2H), 6.78 (d, *J* = 8.3 Hz, 1H), 6.70 (s, 1H), 6.63 (d, *J* = 8.2 Hz, 1H), 5.51 (d, *J* = 6.7 Hz, 1H), 5.12 (d, *J* = 6.2 Hz, 1H), 3.90 (s, 3H), 3.76 (s, 3H), 2.38 (s, 3H), 2.05 (s, 3H); ^{13}C NMR (101 MHz, $CDCl_3$) δ 166.7, 157.4, 145.9, 143.3, 137.3, 131.6, 129.7, 129.3, 129.2, 127.2, 127.2, 127.1, 125.9, 109.9, 60.7, 55.3, 52.1, 21.4, 16.1; HRMS (m/z): $[M+Na]^+$ calcd. for $C_{24}H_{25}NO_5SNa$, 462.1346; found, 462.1341.

The ee value was determined by HPLC analysis: Chiralpak IA Column, hexane/2-propanol= 85/15, 25 ℃, 1.0 mL/min, 214 nm, retention time: 17.9 min (major) and 23.3 min (minor).

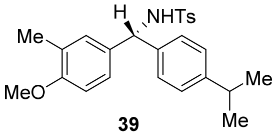

39

(R)-N-((4-isopropylphenyl)(4-methoxy-3-methylphenyl)methyl)-4-methylbenzenesulfonamide 39: 70.6 mg, 84% yield, 93% ee. White solid, mp:115-116 ℃. ^{1}H NMR (400 MHz, Chloroform-*d*) δ 7.52 (d, *J* = 8.1 Hz, 2H), 7.09 (d, *J* = 8.0 Hz, 2H), 7.03 (s, 4H), 6.87 (d, *J* = 8.4 Hz, 1H), 6.80 (s, 1H), 6.62 (d, *J* = 8.4 Hz, 1H), 5.46 (d, *J* = 7.0 Hz, 1H), 5.15 (d, *J* = 7.0 Hz, 1H), 3.75 (s, 3H), 2.81 (hept, *J* = 6.9 Hz, 1H), 2.36 (s, 3H), 2.06 (s,3H), 1.19 (d, *J* = 6.9 Hz, 6H); ^{13}C NMR (101 MHz, $CDCl_3$) δ 157.1, 148.0, 142.8, 138.2, 137.6, 132.3, 129.7, 129.1, 127.2, 127.2, 126.6, 126.4, 125.8, 109.7, 60.8, 55.3, 33.7, 23.9, 23.9, 21.4, 16.1; HRMS (m/z): $[M+Na]^+$ calcd. for $C_{25}H_{29}NO_3SNa$, 446.1760; found, 446.1759.

The ee value was determined by HPLC analysis: Chiralcel OD-H Column, hexane/2-propanol= 90/10, 25 ℃, 1.0 mL/min, 214 nm, retention time: 18.9 min (major) and 39.1 min (minor).

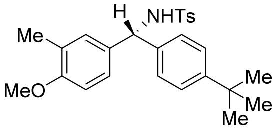

40

(R)-N-((4-(tert-butyl)phenyl)(4-methoxy-3-methylphenyl)methyl)-4-methylbenzenesulfonamide 40: 75.6 mg, 87% yield, 95% ee. White solid, mp:129-130 ℃. ^{1}H NMR (400 MHz, Chloroform-*d*) δ 7.51 (d, *J* = 8.1 Hz, 2H), 7.19 (d, *J* = 8.2 Hz, 2H), 7.09 (d, *J* = 8.0 Hz, 2H), 7.02 (d, *J* = 8.2 Hz, 2H), 6.88 (dd, *J* =8.4 Hz, 1H), 6.81 (s, 1H), 6.62 (d, *J* = 8.4 Hz, 1H), 5.46 (d, *J* = 7.0 Hz, 1H), 5.16 (d, *J* = 7.0 Hz, 1H), 3.75 (s, 3H), 2.35 (s, 3H), 2.06 (s, 3H), 1.25 (s, 9H); ^{13}C NMR (101 MHz, $CDCl_3$) δ 157.1, 150.3, 142.8, 137.7, 137.6, 132.3, 129.7, 129.1, 127.2, 126.9, 126.6, 125.8, 125.3, 109.6, 60.7, 55.3, 34.4, 31.3, 21.4, 16.1; HRMS (m/z): $[M+Na]^+$ calcd. for $C_{26}H_{31}NO_3SNa$, 460.1917; found, 460.1916.

The ee value was determined by HPLC analysis: Chiralcel OD-H Column, hexane/2-propanol= 80/20, 25 ℃, 1.0 mL/min, 254 nm, retention time: 9.7 min (major) and 26.0 min (minor).

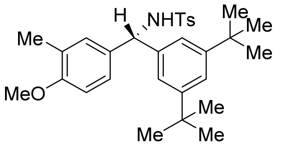

41

(S)-N-((3,5-di-tert-butylphenyl)(4-methoxy-3-methylphenyl)methyl)-4-methylbenzenesulfonamide 41: 76.1 mg, 77% yield, 95% ee. White solid, mp:130-131 ℃. ^{1}H NMR (400 MHz, Chloroform-*d*) δ 7.53 (d, *J* = 7.4 Hz, 2H), 7.21 (s, 1H), 7.08 (d, *J* = 7.6 Hz, 2H), 6.94 – 6.91 (m, 2H), 6.88 (s, 2H), 6.65 (d, *J* = 8.1 Hz, 1H), 5.50 (d, *J* = 6.9 Hz, 1H), 5.17 (d, *J* = 6.8 Hz, 1H), 3.77 (s, 3H), 2.33 (s, 3H), 2.09 (s, 3H), 1.20 (s, 18H); ^{13}C NMR (101 MHz, $CDCl_3$) δ 156.9, 150.8, 142.7, 139.6, 137.6, 132.4, 129.8, 129.2, 127.2, 126.4, 125.8, 121.5, 121.4, 109.5, 61.5, 55.3, 34.7, 31.3, 21.4, 16.2; HRMS (m/z): $[M+Na]^+$ calcd. for $C_{30}H_{39}NO_3SNa$, 516.2543; found, 516.2543.

The ee value was determined by HPLC analysis: Chiralcel OD-H Column, hexane/2-propanol= 90/10, 25 ℃, 1.0 mL/min, 254 nm, retention time: 4.8 min (minor) and 7.9 min (major).

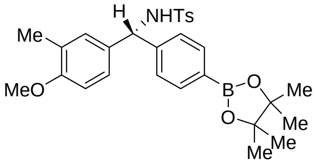

42

(R)-N-((4-methoxy-3-methylphenyl)(4-(4,4,5,5-tetramethyl-1,3,2-dioxaborolan-2-yl)phenyl)methyl)-4-methylbenzenesulfonamide 42: 69.4 mg, 68% yield, 95% ee. White solid, mp:157-158 ℃. ^{1}H NMR (400 MHz, Chloroform-*d*) δ 7.64 (d, *J* = 7.8 Hz, 2H), 7.54 (d, *J* = 8.1 Hz, 2H), 7.12 (d, *J* = 7.8 Hz, 4H), 6.84 (d, *J* = 8.4 Hz, 1H), 6.75 (s, 1H), 6.62 (d, *J* = 8.4 Hz, 1H), 5.48 (d, *J* = 7.0 Hz, 1H), 5.07 (d, *J* = 7.0 Hz, 1H), 3.76 (s, 3H), 2.37 (s, 3H), 2.05 (s, 3H), 1.33 (s, 12H); ^{13}C NMR (101 MHz, $CDCl_3$) δ 157.2, 143.7, 143.1, 137.3, 134.9, 132.0, 129.7, 129.3, 127.2, 126.8, 126.6, 125.9, 109.7, 83.8, 60.9, 55.3, 24.8, 24.8, 21.4, 16.1; HRMS (m/z): $[M+Na]^+$ calcd. for $C_{28}H_{34}BNO_5SNa$, 530.2148; found, 530.2168.

The ee value was determined by HPLC analysis: Chiralcel OD-H Column, hexane/2-propanol= 90/10, 25 ℃, 1.0 mL/min, 254 nm, retention time: 15.3 min (minor) and 22.1 min (major).

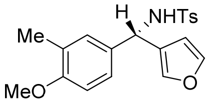

43

(S)-N-(furan-3-yl(4-methoxy-3-methylphenyl)methyl)-4-methylbenzenesulfonamide 43: 43.8 mg, 59% yield, 80% ee. White solid, mp:118-119 ℃. ^{1}H NMR (400 MHz, Chloroform-*d*) δ 7.56 (d, *J* = 7.6 Hz, 2H), 7.27 (s, 1H), 7.15 (d, *J* = 7.6 Hz, 2H), 7.05 (s, 1H), 6.90 (d, *J* = 8.2 Hz, 1H), 6.76 (s, 1H), 6.63 (d, *J* = 8.2 Hz, 1H), 6.13 (s, 1H), 5.38 (d, *J* = 7.4 Hz, 1H), 5.13 (d, *J* = 7.1 Hz, 1H), 3.77 (s, 3H), 2.38 (s, 3H), 2.05 (s, 3H); ^{13}C NMR (101 MHz, $CDCl_3$) δ 157.2, 143.4, 143.0, 140.4, 137.5, 131.3, 129.4, 129.2, 127.1, 126.6, 126.6, 125.5, 109.6, 109.5, 55.3, 53.8, 21.4, 16.1; HRMS (m/z): $[M+Na]^+$ calcd. for $C_{20}H_{21}NO_4SNa$, 394.1083; found, 394.1083.

The ee value was determined by HPLC analysis: Chiralcel OD-H Column, hexane/2-propanol= 90/10, 25 ℃, 1.0 mL/min, 254 nm, retention time: 15.4 min (major) and 21.4 min (minor).

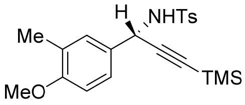

44

(S)-N-(1-(4-methoxy-3-methylphenyl)-3-(trimethylsilyl)prop-2-yn-1-yl)-4-methylbenzenesulfonamide 44: 52.6 mg, 66% yield, 45% ee. Yellow solid, mp:106-107 ℃. ^{1}H NMR (400 MHz, Chloroform-*d*) δ 7.77 (d, *J* = 8.1 Hz, 2H), 7.29 – 7.25 (m, 3H), 7.18 (s, 1H), 6.74 (d, *J* = 8.4 Hz, 1H), 5.25 (d, *J* = 8.9 Hz, 1H), 4.82 (d, *J* = 8.8 Hz, 1H), 3.81 (s, 3H), 2.43 (s, 3H), 2.17 (s, 3H), 0.03 (s, 9H); ^{13}C NMR (101 MHz, $CDCl_3$) δ 157.7, 143.3, 137.5, 129.7, 129.4, 128.7, 127.5, 126.9, 125.7, 109.6, 101.9, 91.1, 55.3, 49.2, 21.5, 16.2, -0.4; HRMS (m/z): $[M+Na]^+$ calcd. for $C_{21}H_{27}NO_3SSiNa$, 424.1373; found, 424.1360.

The ee value was determined by HPLC analysis: Chiralpak IB Column, hexane/2-propanol= 90/10, 25 ℃, 1.0 mL/min, 214 nm, retention time: 6.6 min (major) and 7.5 min (minor).

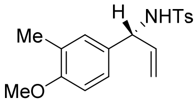

45

(R)-N-(1-(4-methoxy-3-methylphenyl)allyl)-4-methylbenzenesulfonamide 45: 31.4 mg, 48% yield, 33% ee. Yellow solid, mp:77-78 ℃. ^{1}H NMR (400 MHz, Chloroform-*d*) δ 7.62 (d, *J* = 8.2 Hz, 2H), 7.19 (d, *J* = 8.1 Hz, 2H), 6.89 (dd, *J* = 8.3, 1.9 Hz, 1H), 6.75 (d, *J* = 1.8 Hz, 1H), 6.65 (d, *J* = 8.4 Hz, 1H), 5.89 – 5.81 (m, 1H), 5.17 – 5.10 (m, 2H), 4.86 – 4.80 (m, 2H), 3.78 (s, 3H), 2.40 (s, 3H), 2.07 (s, 3H); ^{13}C NMR (101 MHz, $CDCl_3$) δ 157.3, 143.1, 137.7, 137.4, 131.0, 129.4, 129.3, 127.3, 126.8, 125.5, 116.3, 109.8, 59.4, 55.3, 21.5, 16.1; HRMS (m/z): $[M+Na]^+$ calcd. for $C_{18}H_{21}NO_3SNa$, 354.1134; found, 354.1133.

The ee value was determined by HPLC analysis: Chiralpak IC Column, hexane/2-propanol= 90/10, 25 ℃, 1.0 mL/min, 214 nm, retention time: 32.7 min (minor) and 35.2 min (major).

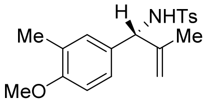

46

(R)-N-(1-(4-methoxy-3-methylphenyl)-2-methylallyl)-4-methylbenzenesulfonamide 46: 21.2 mg, 31% yield, 96% ee. Yellow solid, mp:98-99 ℃. ^{1}H NMR (400 MHz, Chloroform-*d*) δ 7.62 (d, *J* = 8.2 Hz, 2H), 7.19 (d, *J* = 8.1 Hz, 2H), 6.86 (dd, *J* = 8.3, 2.1 Hz, 1H), 6.71 (d, *J* = 1.7 Hz, 1H), 6.64 (d, *J* = 8.4 Hz, 1H), 5.01 (s, 1H), 4.90 (s, 1H), 4.81 (d, *J* = 7.2 Hz, 1H), 4.71 (d, *J* = 7.1 Hz, 1H), 3.77 (s, 3H), 2.40 (s, 3H), 2.07 (s, 3H), 1.54 (s, 3H); ^{13}C NMR (101 MHz, $CDCl_3$) δ 157.3, 143.6, 143.0, 137.6, 130.4, 129.3, 129.3, 127.3, 126.7, 125.4, 112.8, 109.7, 62.3, 55.3, 21.5, 19.7, 16.1; HRMS (m/z): $[M+Na]^+$ calcd. for $C_{19}H_{23}NO_3SNa$, 368.1291; found, 368.1293.

The ee value was determined by HPLC analysis: Chiralpak IC Column, hexane/2-propanol= 90/10, 25 ℃, 1.0 mL/min, 214 nm, retention time: 12.5 min (minor) and 13.4 min (major).

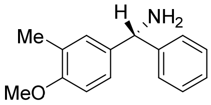

57

(R)-(4-methoxy-3-methylphenyl)(phenyl)methanamine 48: 44.9 mg, 66% yield, 90% ee. Light yellow oil. ^{1}H NMR (400 MHz, Chloroform-*d*) δ 7.38 – 7.36 (m, 2H), 7.30 (t, *J* = 7.5 Hz, 2H), 7.21 (t, *J* = 7.2 Hz, 1H), 7.16 – 7.13 (m, 2H), 6.75 (d, *J* = 8.1 Hz, 1H), 5.14 (s, 1H), 3.79 (s, 3H), 2.18 (s, 3H), 1.80 (s, 2H); ^{13}C NMR (101 MHz, $CDCl_3$) δ 156.69, 145.91, 137.33, 129.24, 128.38, 126.74, 126.58, 125.00, 109.69, 59.11, 55.30, 16.29; HRMS (m/z): $[M+Na]^+$ calcd. for $C_{15}H_{17}NONa$, 250.1202; found, 250.1185. The ee value was determined by HPLC analysis: Chiralpak AS-H Column, hexane/2-propanol/NEt_3= 95/5/0.1, 25 ℃, 1.0 mL/min, 254 nm, retention time: 9.4 min (major) and 10.8 min (minor).

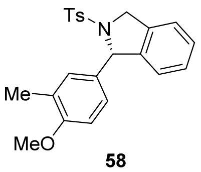

58

(S)-1-(4-methoxy-3-methylphenyl)-2-tosylisoindoline 49: 16.1 mg, 41% yield, 96% ee. Yellow solid, mp:145-146 ℃. ^{1}H NMR (400 MHz, Chloroform-*d*) δ 7.51 (d, *J* = 8.2 Hz, 2H), 7.23 – 7.22 (m, 2H), 7.19 – 7.13 (m, 3H), 7.04 (dd, *J* = 8.3, 2.1 Hz, 1H), 6.88 (d, *J* = 7.6 Hz, 1H), 6.81 (d, *J* = 1.7 Hz, 1H), 6.70 (d, *J* = 8.3 Hz, 1H), 5.84 (s, 1H), 4.83 (s, 2H), 3.80 (s, 3H), 2.35 (s, 3H), 2.07 (s, 3H); ^{13}C NMR (101 MHz, $CDCl_3$) δ 157.4, 143.0, 141.4, 135.6, 134.9, 133.3, 129.8, 129.3, 127.9, 127.8, 127.3, 126.6, 126.3, 123.6, 122.3, 109.5, 69.1, 55.3, 53.9, 21.4, 16.2; HRMS (m/z): $[M+Na]^+$ calcd. for $C_{23}H_{23}NO_3SNa$, 416.1291; found, 416.1289.

The ee value was determined by HPLC analysis: Chiralcel OD-H Column, hexane/2-propanol= 90/10, 25 ℃, 1.0 mL/min, 214 nm, retention time: 20.5 min (minor) and 22.4 min (major).

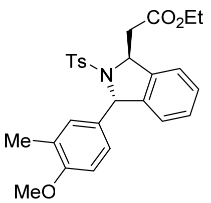

59

Ethyl 2-((1S,3S)-3-(4-methoxy-3-methylphenyl)-2-tosylisoindolin-1-yl)acetate 50: 184.4 mg, 77% yield, 90:10 d.r., 93% ee. Yellow solid, mp:45-46 ℃. ^{1}H NMR (400 MHz, Chloroform-*d*) δ 7.73 (d, *J* = 8.3 Hz, 2H), 7.25 – 7.15 (m, 6H), 7.11 (dt, *J* = 8.3, 2.2 Hz, 1H), 7.02 (d, *J* = 1.8 Hz, 1H), 6.88 (d, *J* = 7.5 Hz, 1H), 6.76 (d, *J* = 8.4 Hz, 1H), 5.73 (s, 1H), 5.45 (dd, *J* = 9.0, 3.8 Hz, 1H), 4.26 (qd, *J* = 7.1, 1.8 Hz, 2H), 3.80 (s, 3H), 3.45 (dd, *J* = 16.2, 3.9 Hz, 1H), 2.88 (dd, *J* = 16.2, 9.0 Hz, 1H), 2.36 (s, 3H), 2.17 (s, 3H), 1.32 (t, *J* = 7.1 Hz, 3H); ^{13}C NMR (101 MHz, $CDCl_3$) δ 171.04, 157.37, 143.63, 140.00, 138.65, 134.48, 133.86, 129.72, 129.65, 128.32, 128.09, 127.78, 126.65, 125.99, 123.62, 122.63, 109.65, 69.03, 62.00, 60.79, 55.28, 44.20, 21.44, 16.35, 14.18; HRMS (m/z): $[M+Na]^+$ calcd. for $C_{27}H_{29}NO_5SNa$, 502.1659; found, 502.1653.

The ee value was determined by HPLC analysis: Chiralpak IG-3 Column, hexane/2-propanol= 70/30, 25 ℃, 2.0 mL/min, 254 nm, retention time: 24.3 min (major) and 46.5 min (minor).

Supplementary Figures

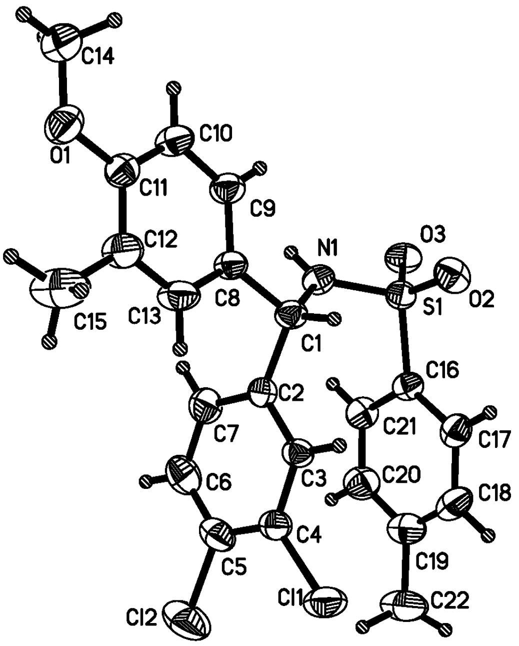

Supplementary Figure 1. X-ray crystal structures of compound **34**

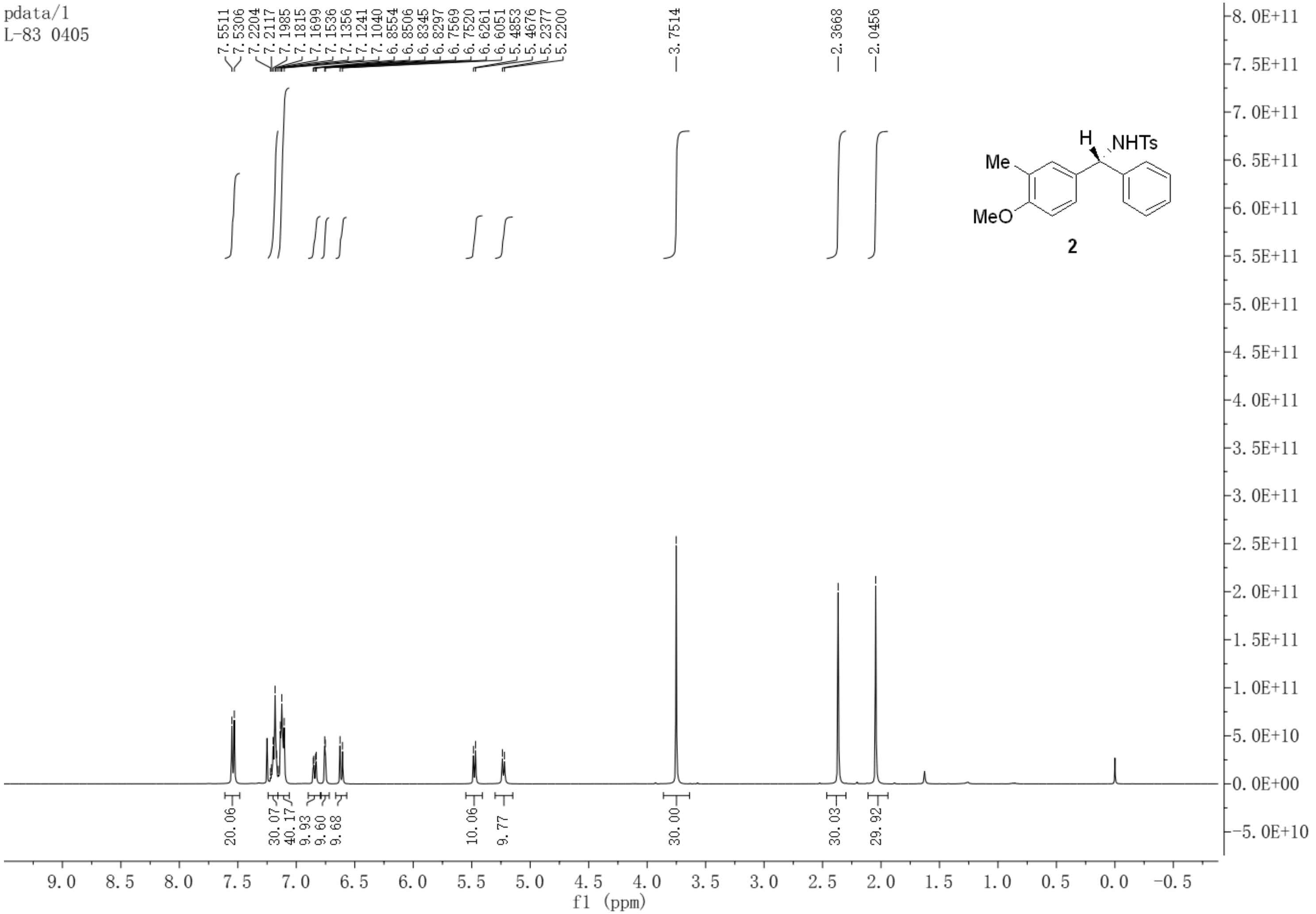

Supplementary Figure 2. ^{1}H NMR spectra of compound **2**

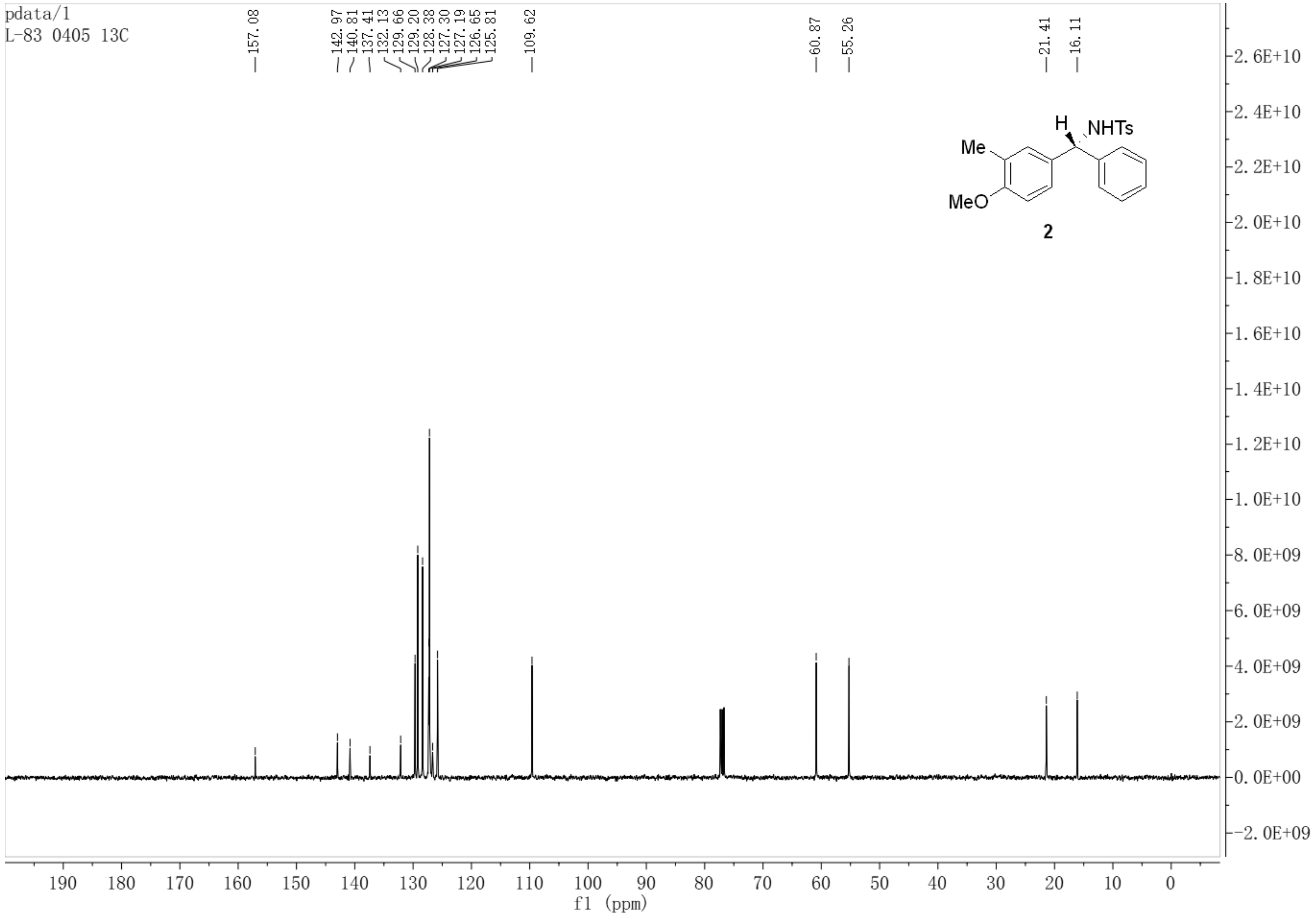

Supplementary Figure 3. ^{13}C NMR spectra of compound **2**

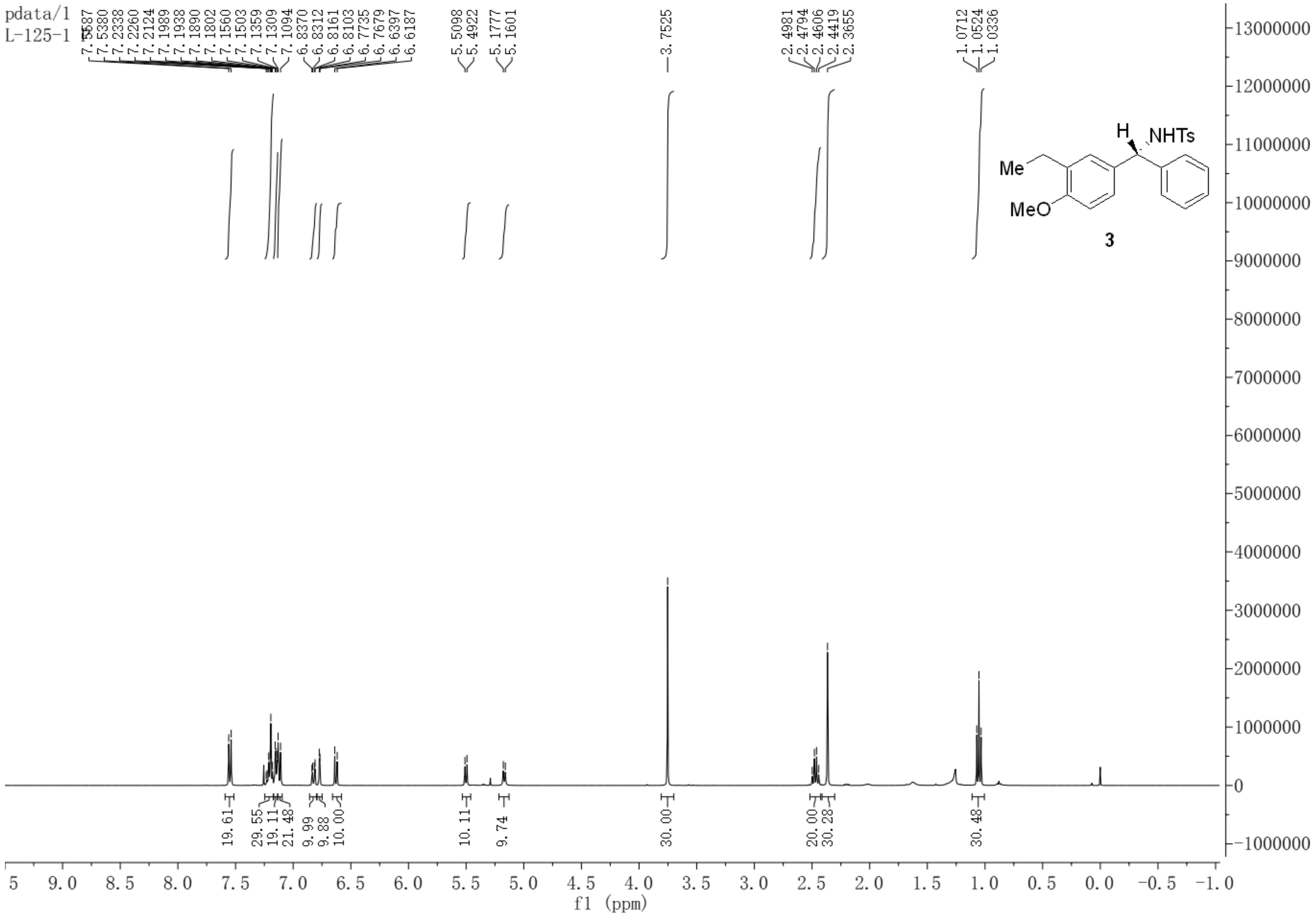

Supplementary Figure 4. ^{1}H NMR spectra of compound **3**

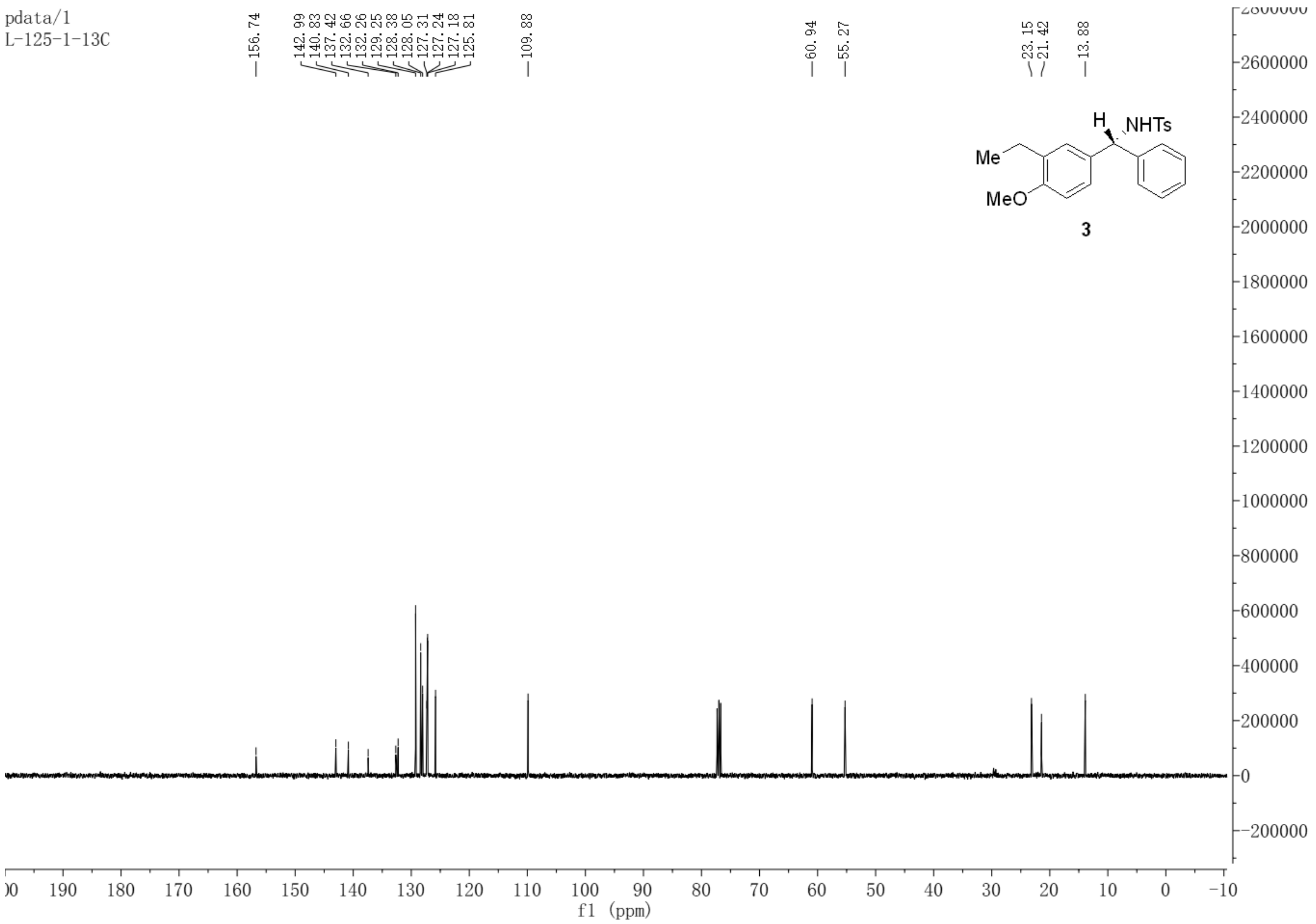

Supplementary Figure 5. ^{13}C NMR spectra of compound **3**

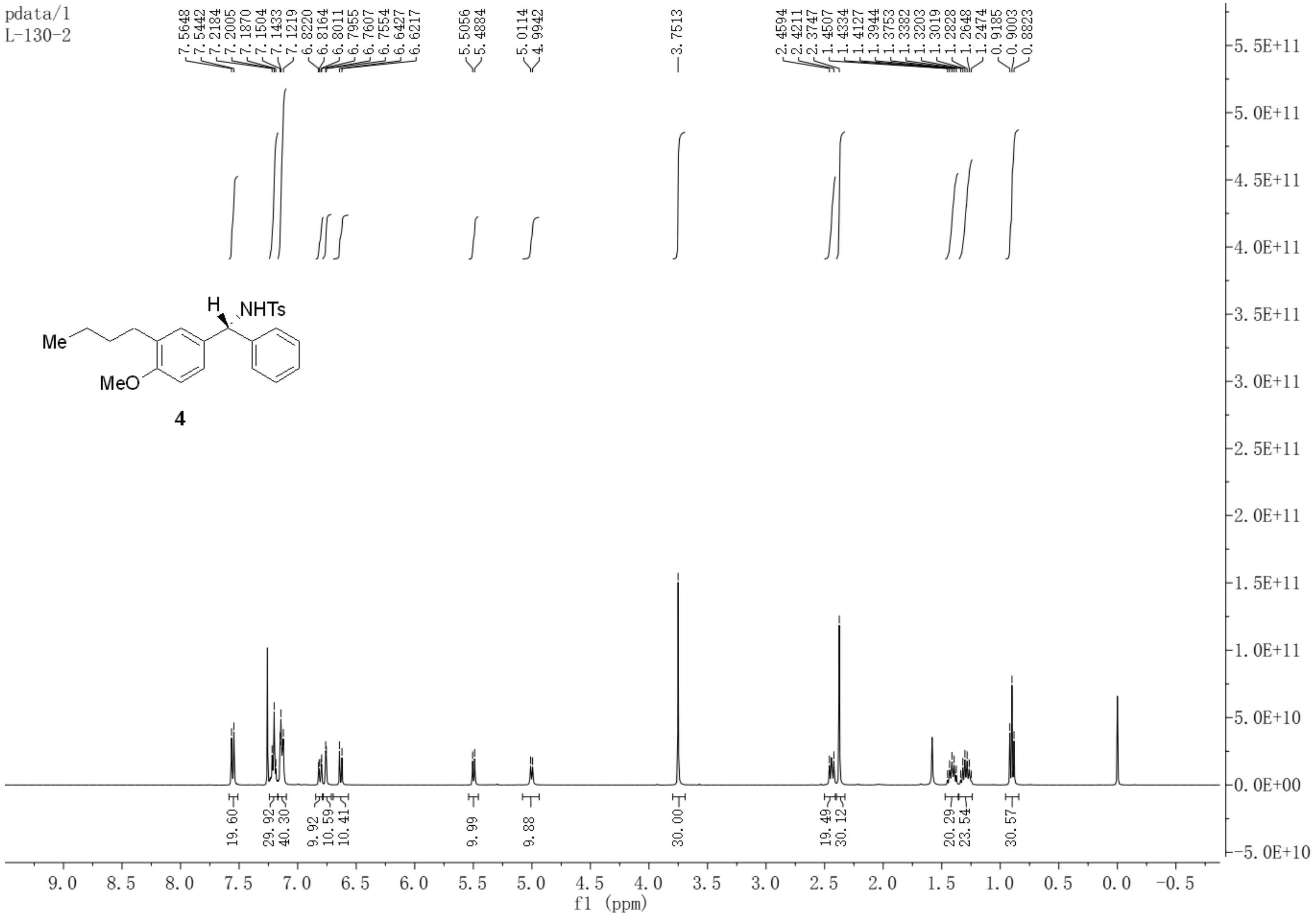

Supplementary Figure 6. ^{1}H NMR spectra of compound **4**

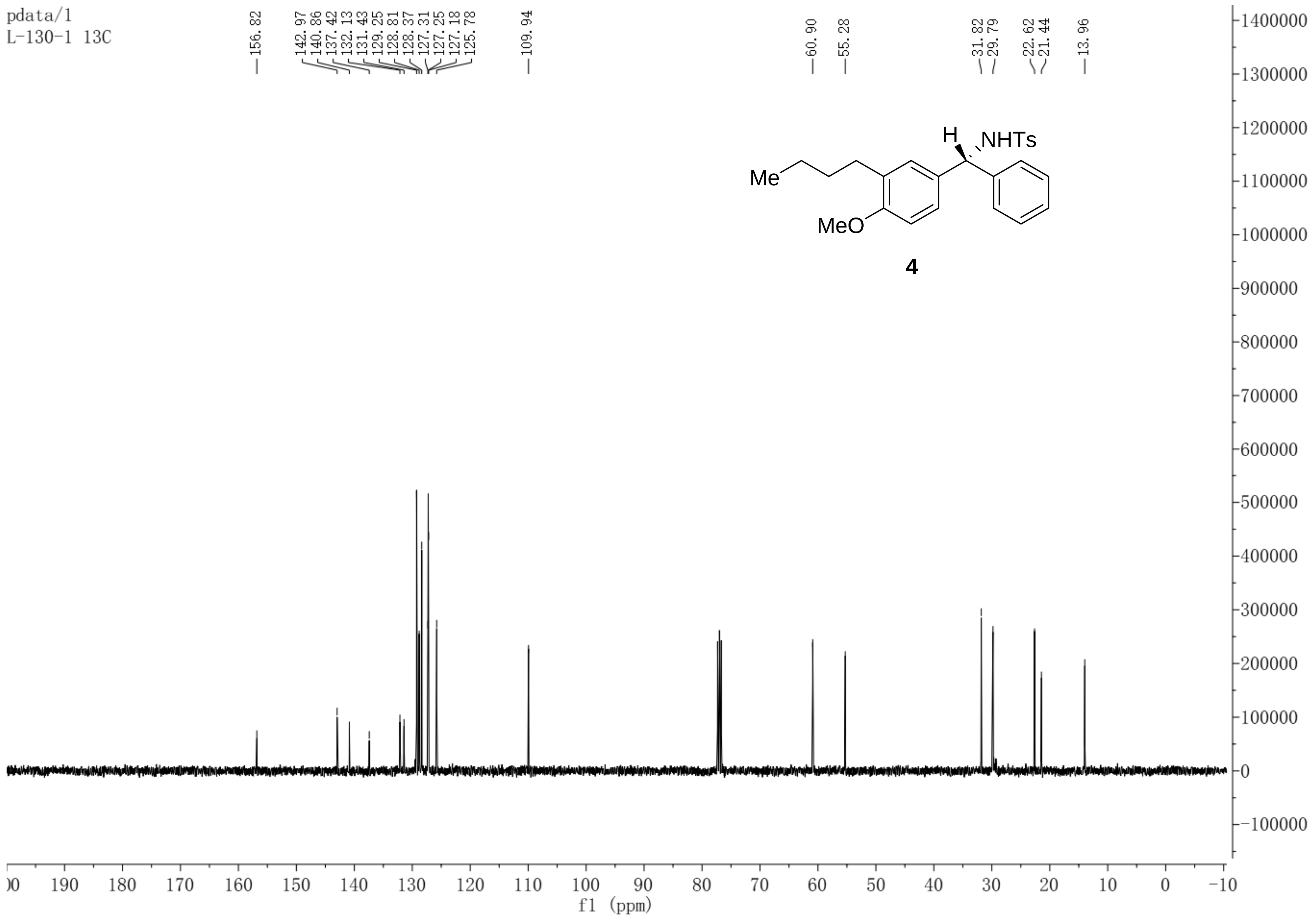

Supplementary Figure 7. ^{13}C NMR spectra of compound **4**

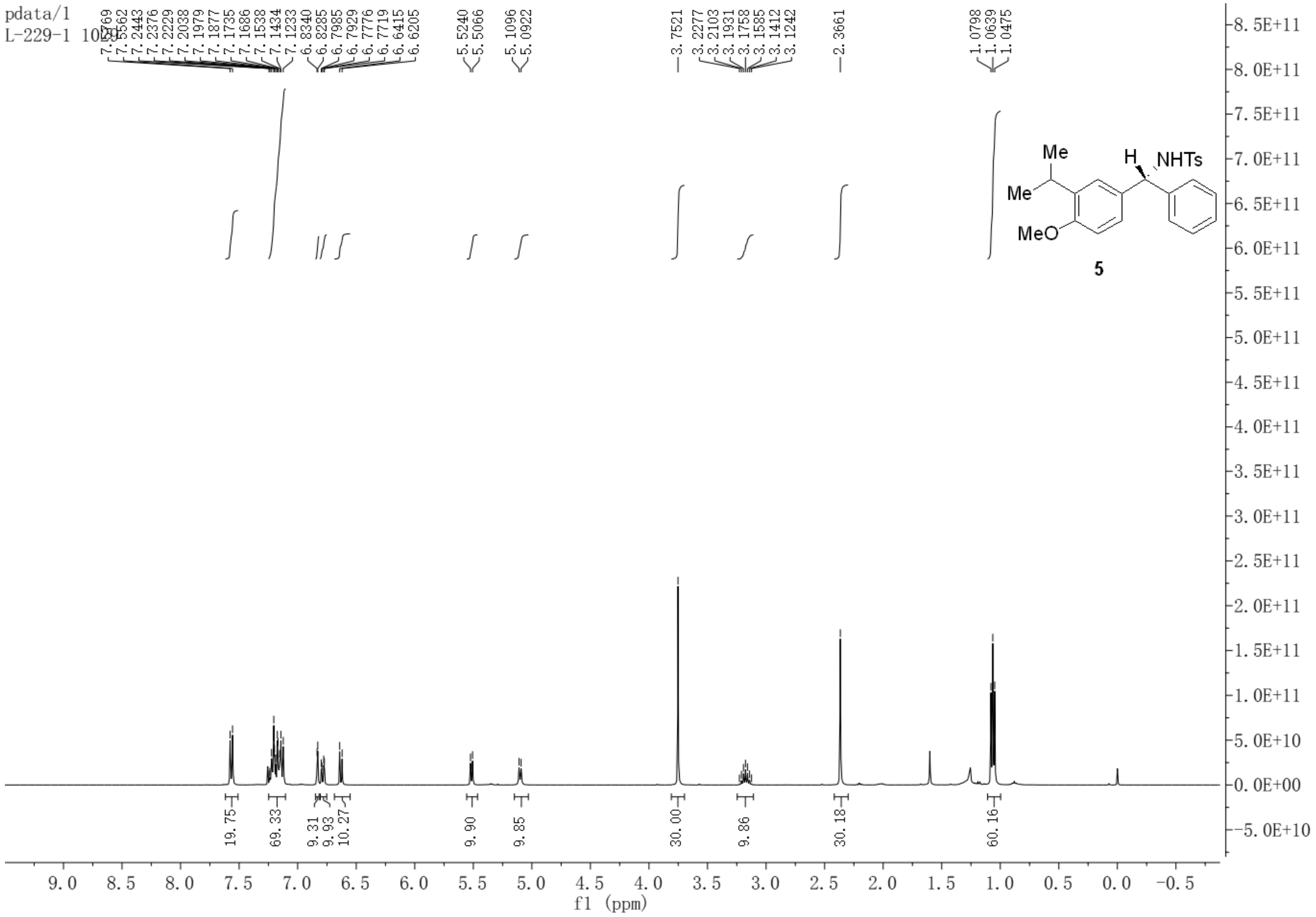

Supplementary Figure 8. ^{1}H NMR spectra of compound **5**

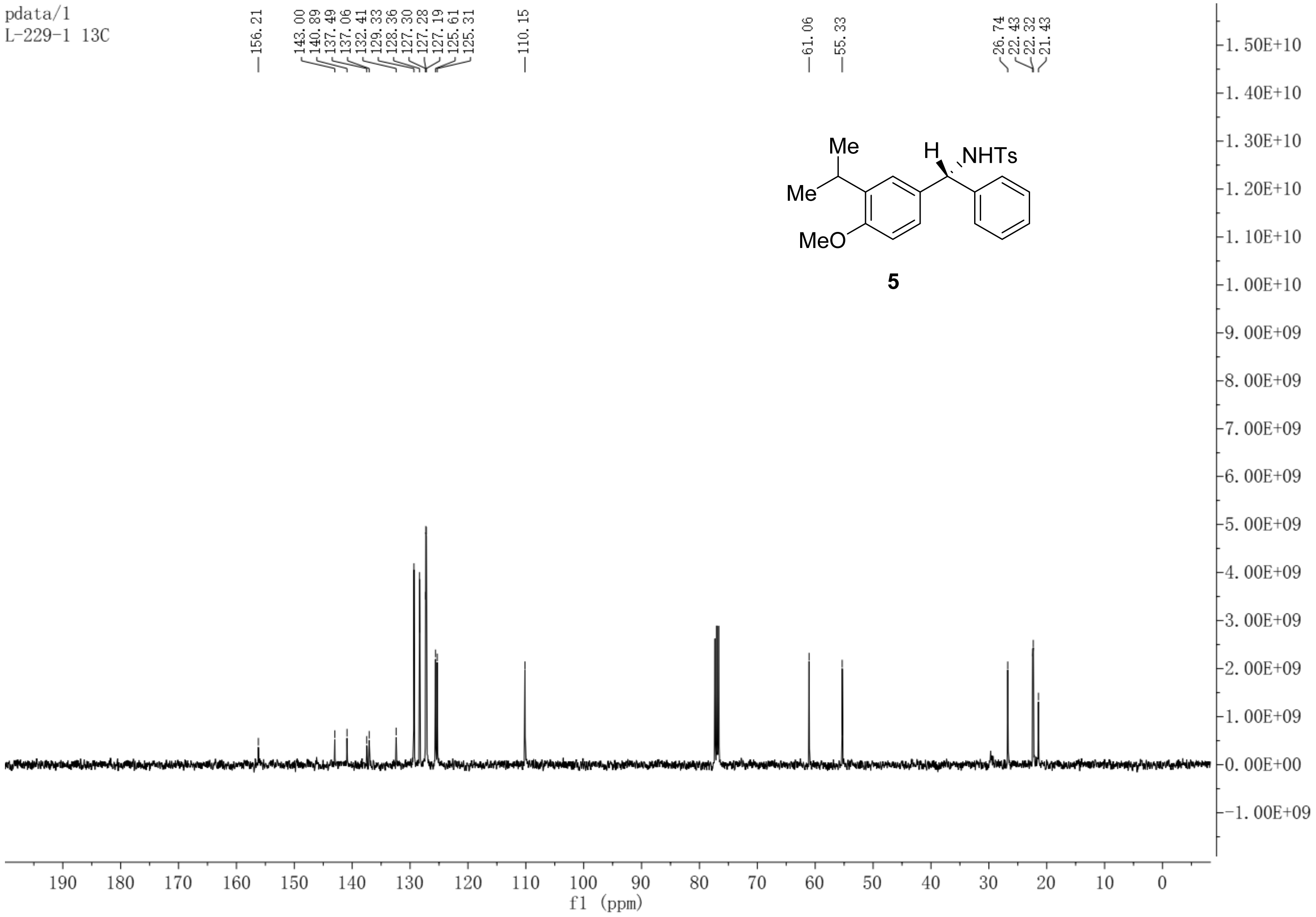

Supplementary Figure 9. ^{13}C NMR spectra of compound **5**

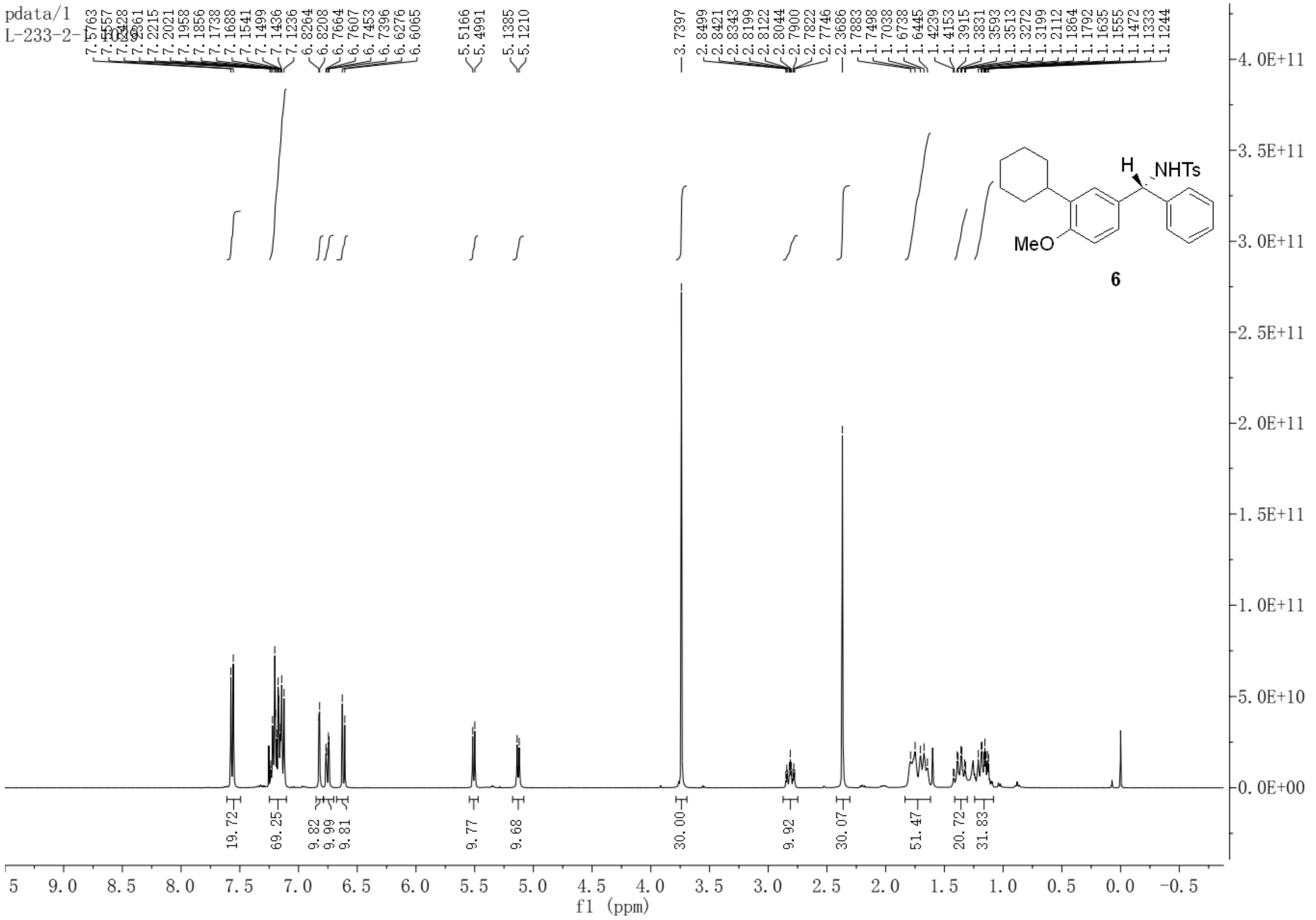

Supplementary Figure 10. ^{1}H NMR spectra of compound **6**

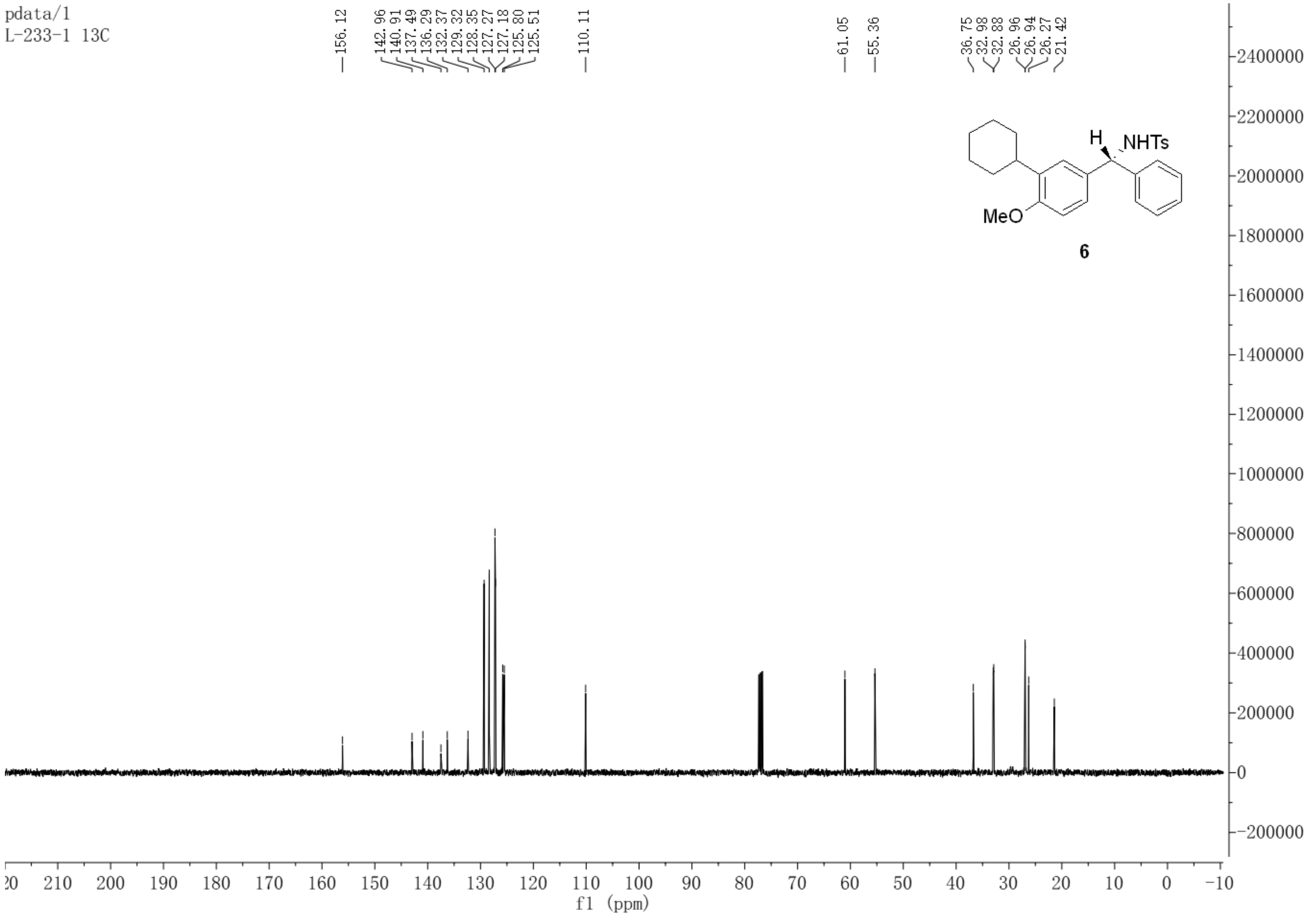

Supplementary Figure 11. ^{13}C NMR spectra of compound **6**

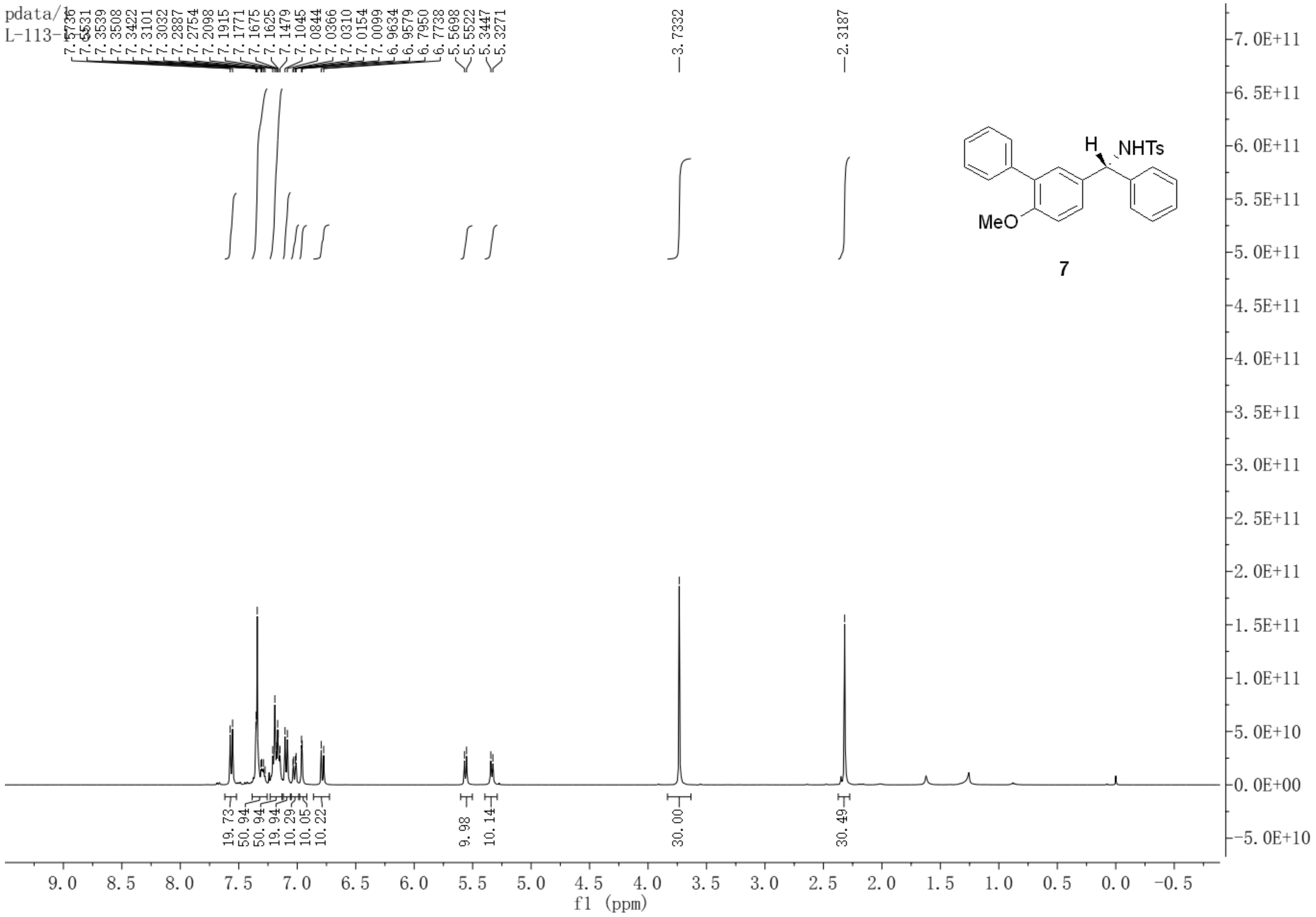

Supplementary Figure 12. ^{1}H NMR spectra of compound **7**

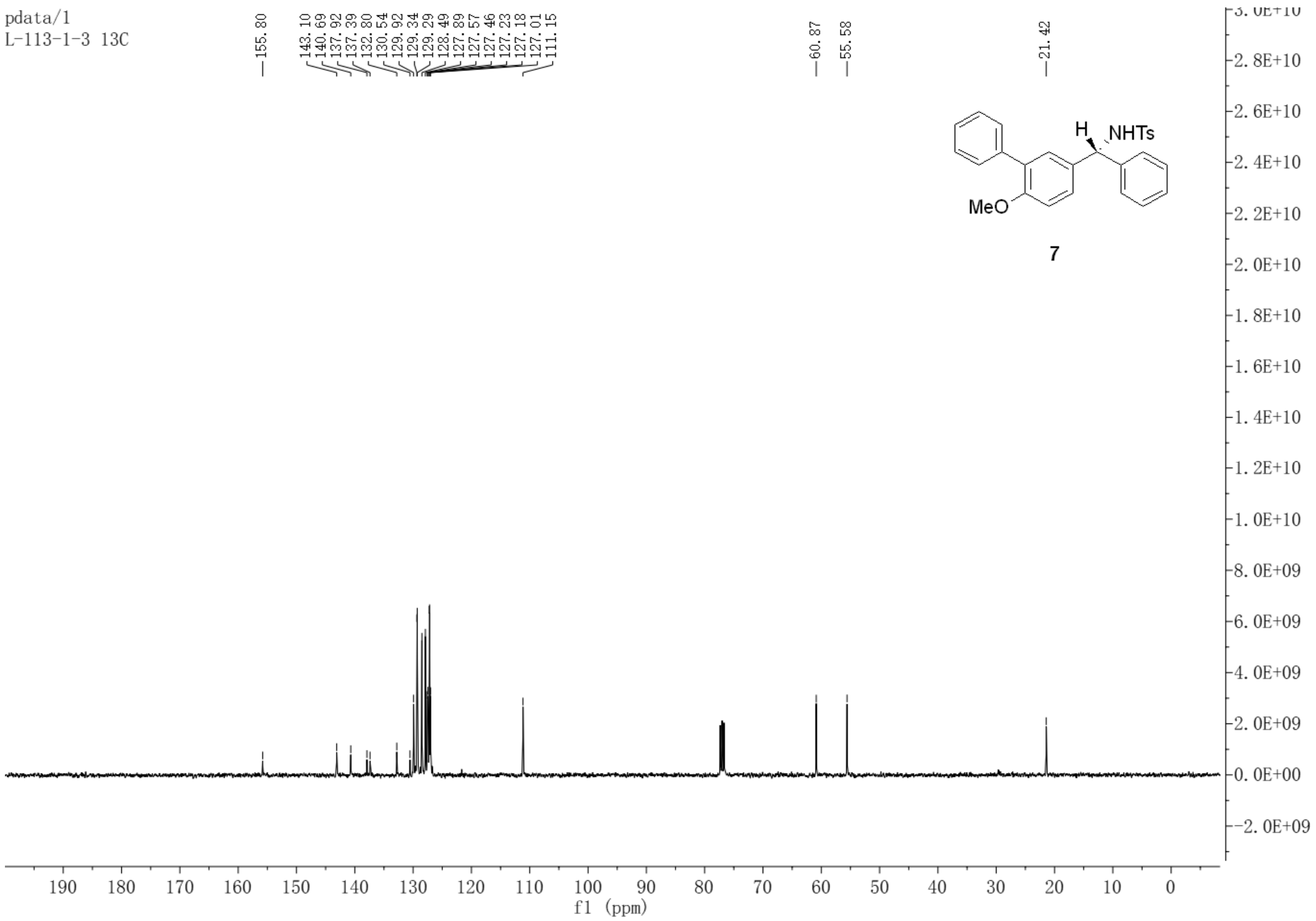

Supplementary Figure 13. ^{13}C NMR spectra of compound **7**

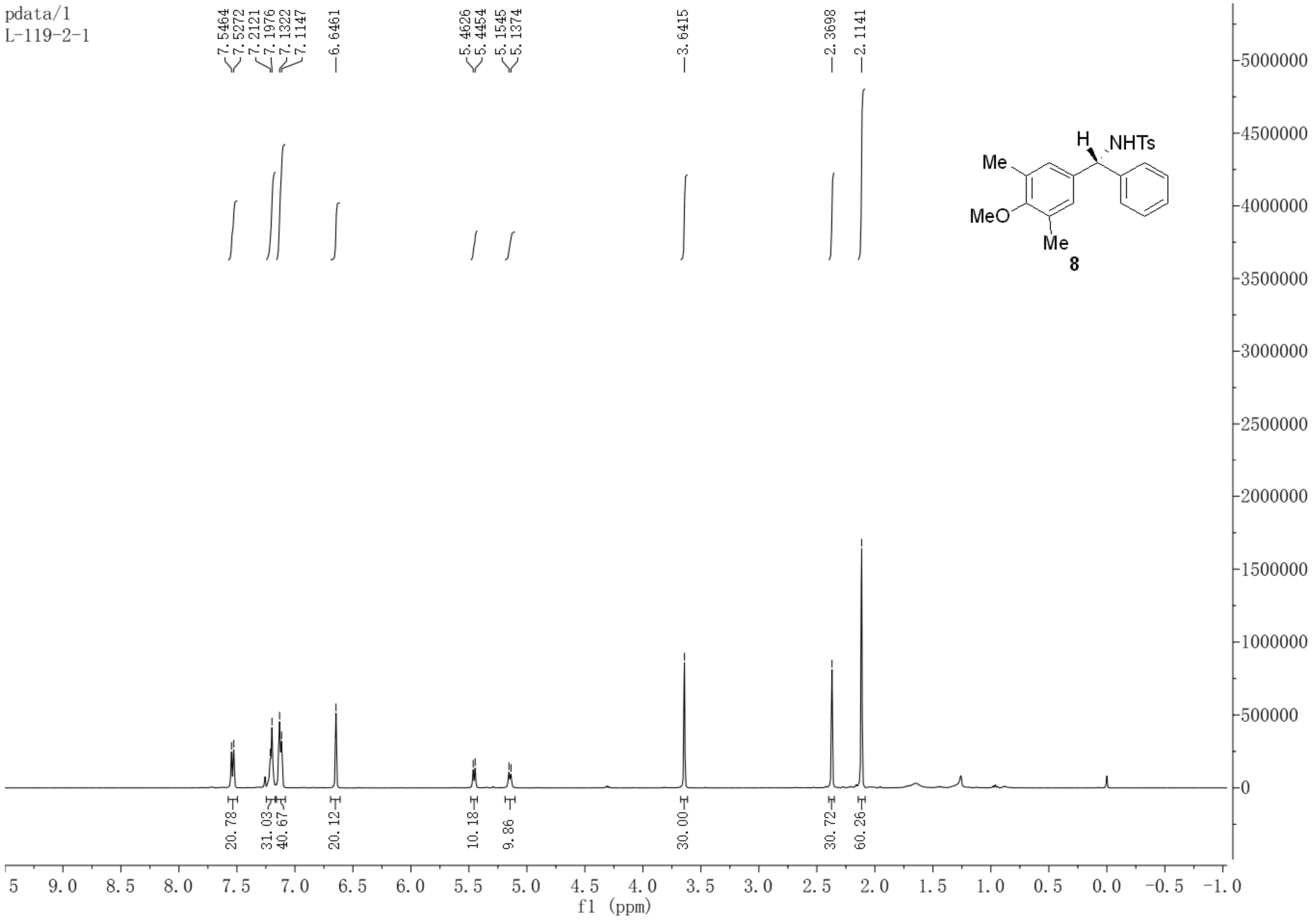

Supplementary Figure 14. ^{1}H NMR spectra of compound **8**

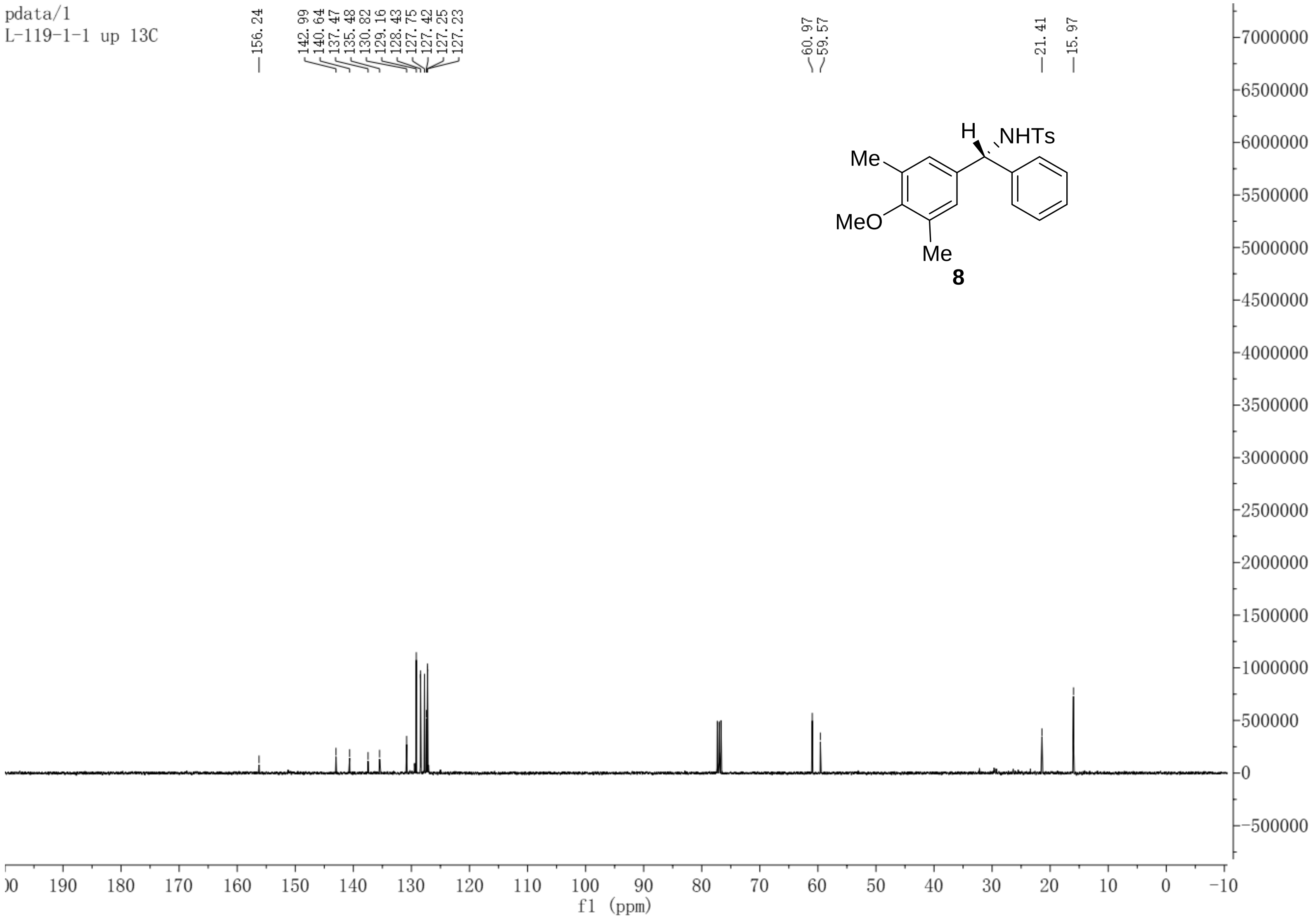

Supplementary Figure 15. ^{13}C NMR spectra of compound **8**

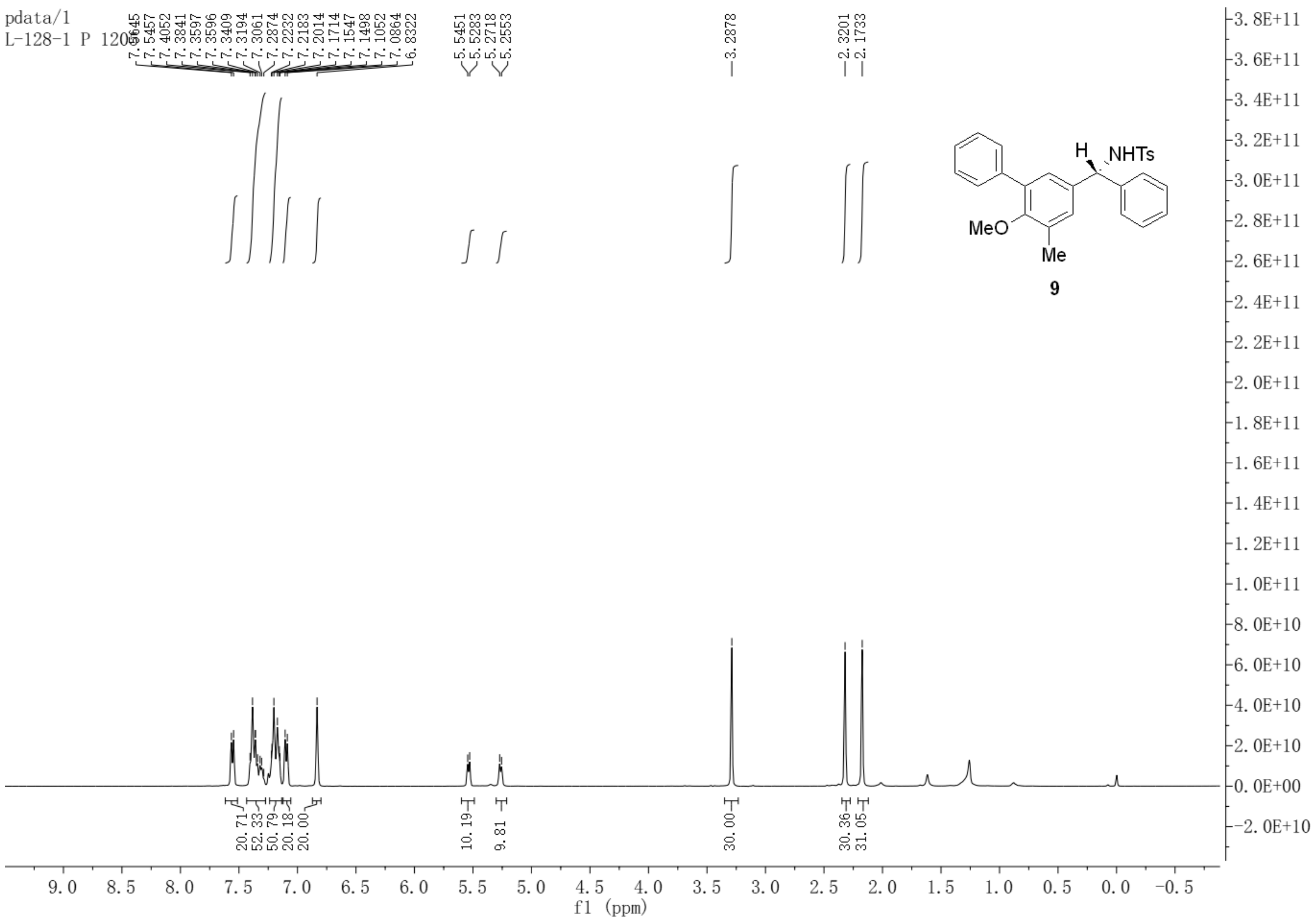

Supplementary Figure 16. ^{1}H NMR spectra of compound **9**

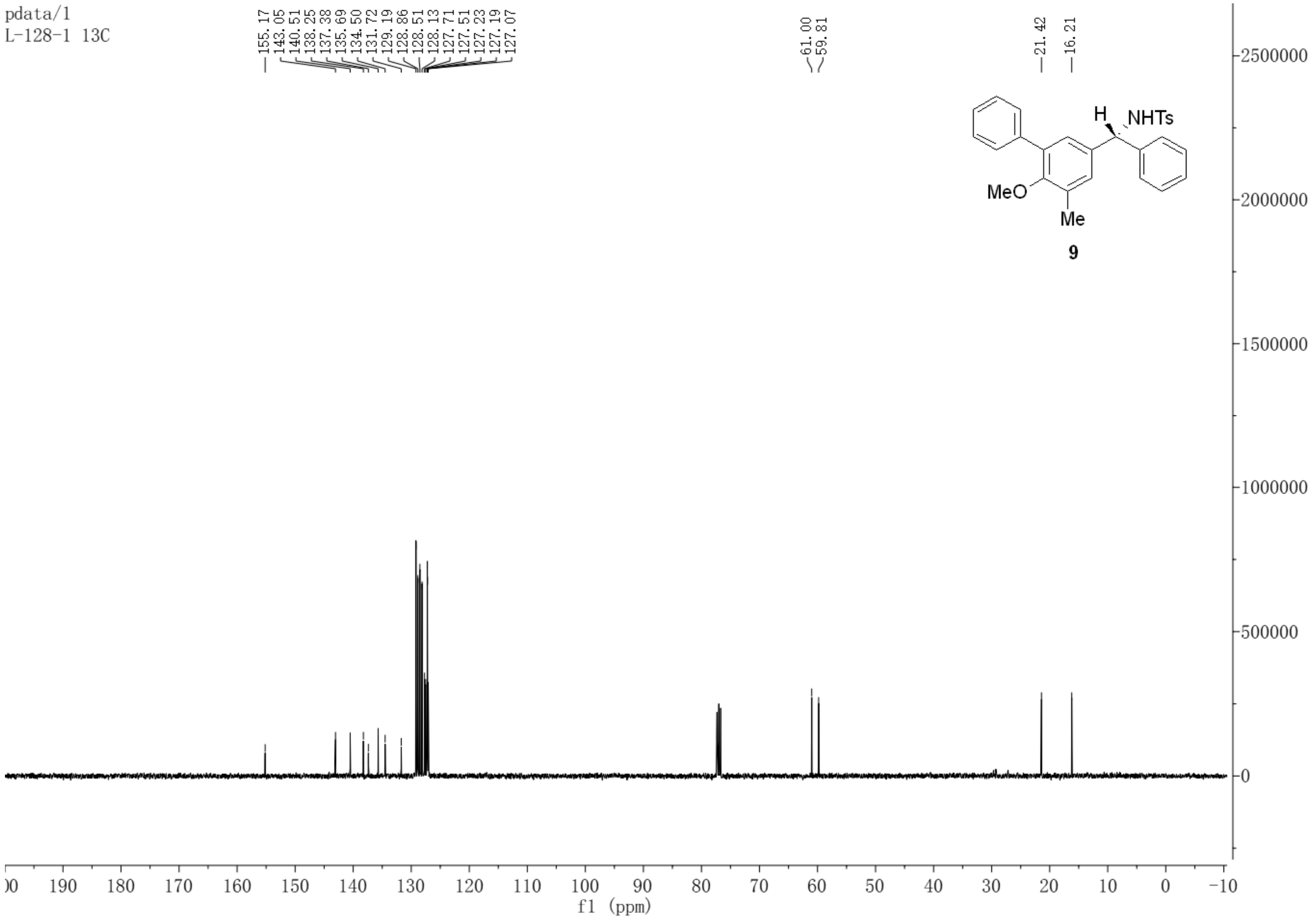

Supplementary Figure 17. ^{13}C NMR spectra of compound **9**

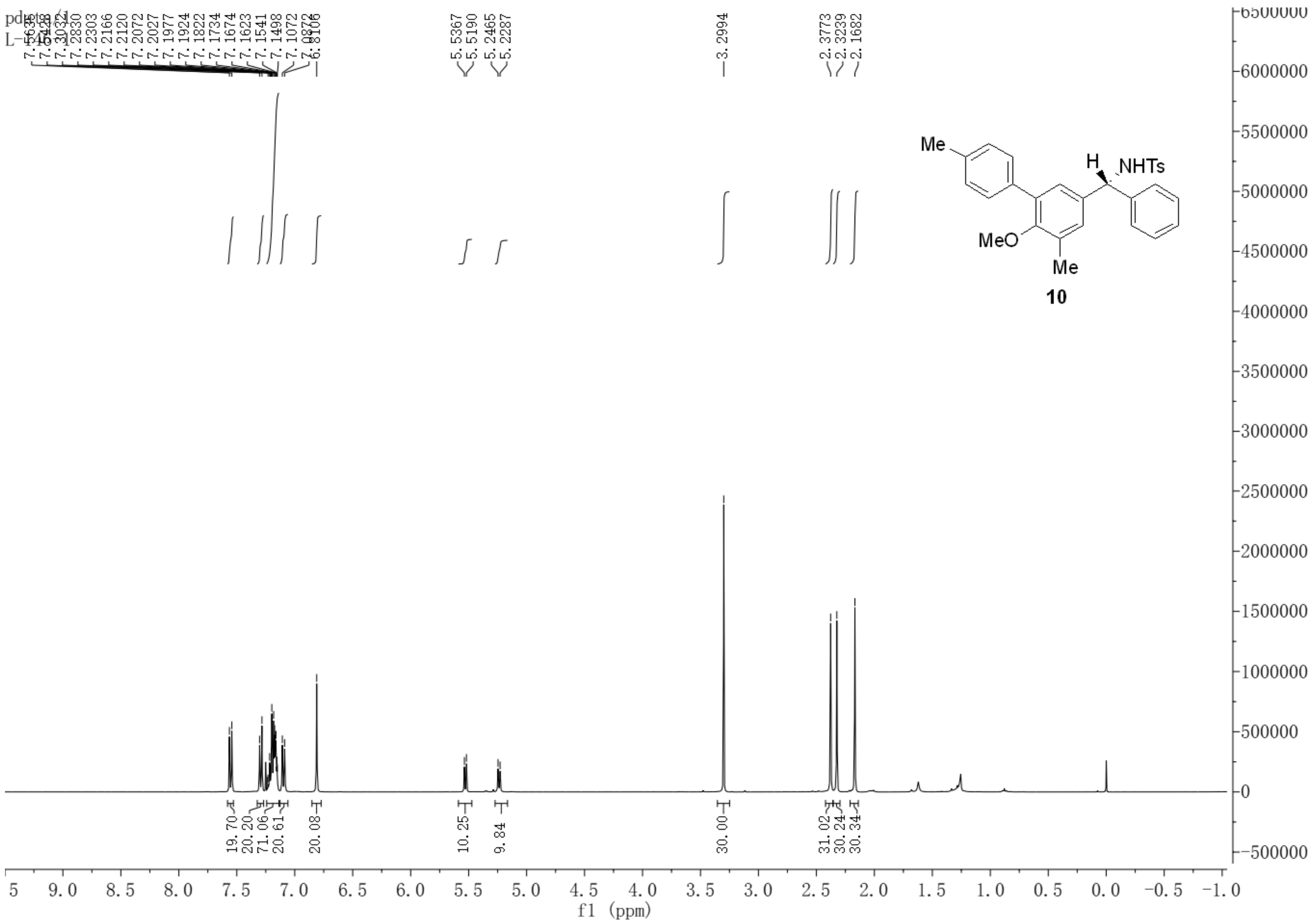

Supplementary Figure 18. ^{1}H NMR spectra of compound **10**

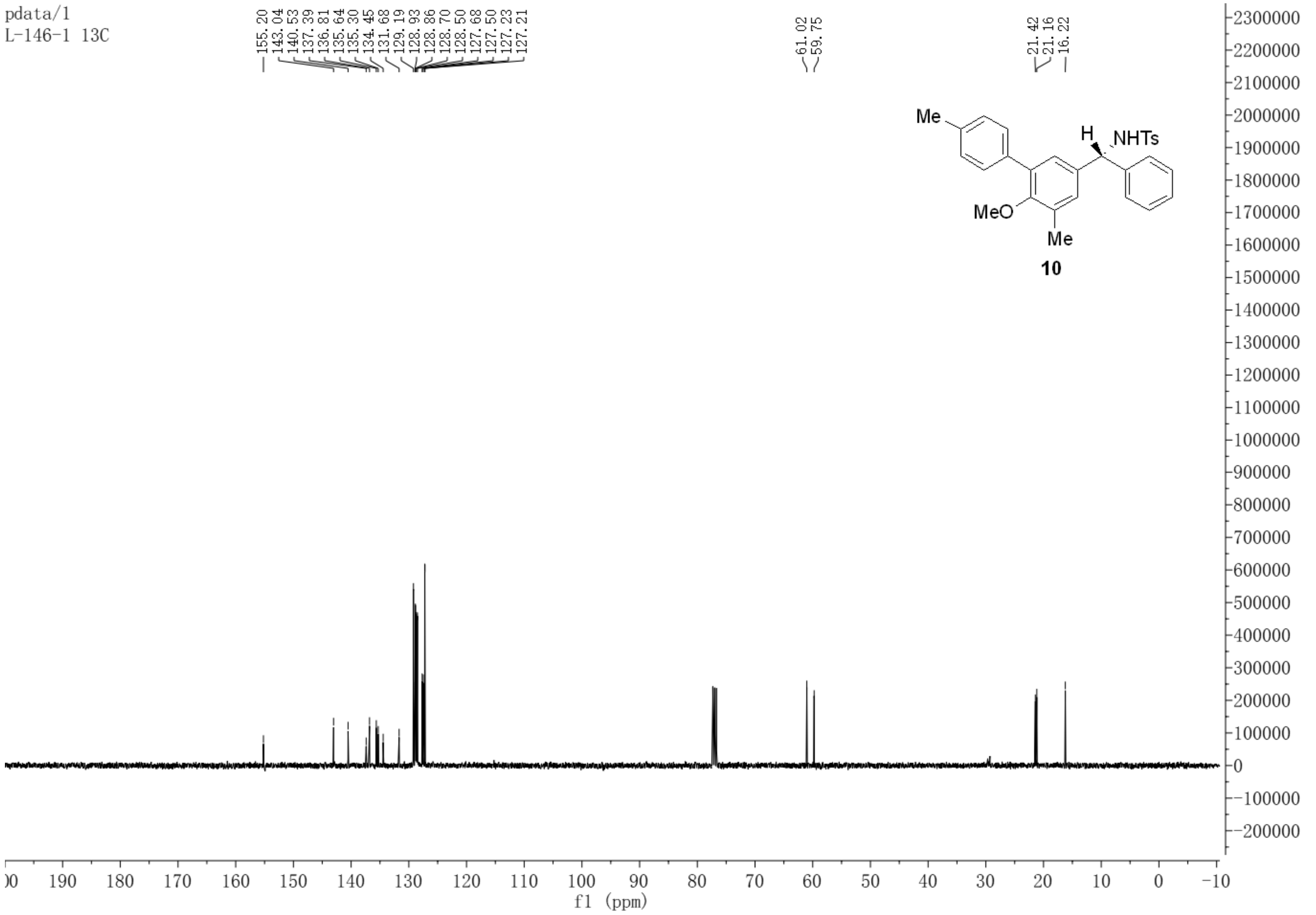

Supplementary Figure 19. ^{13}C NMR spectra of compound **10**

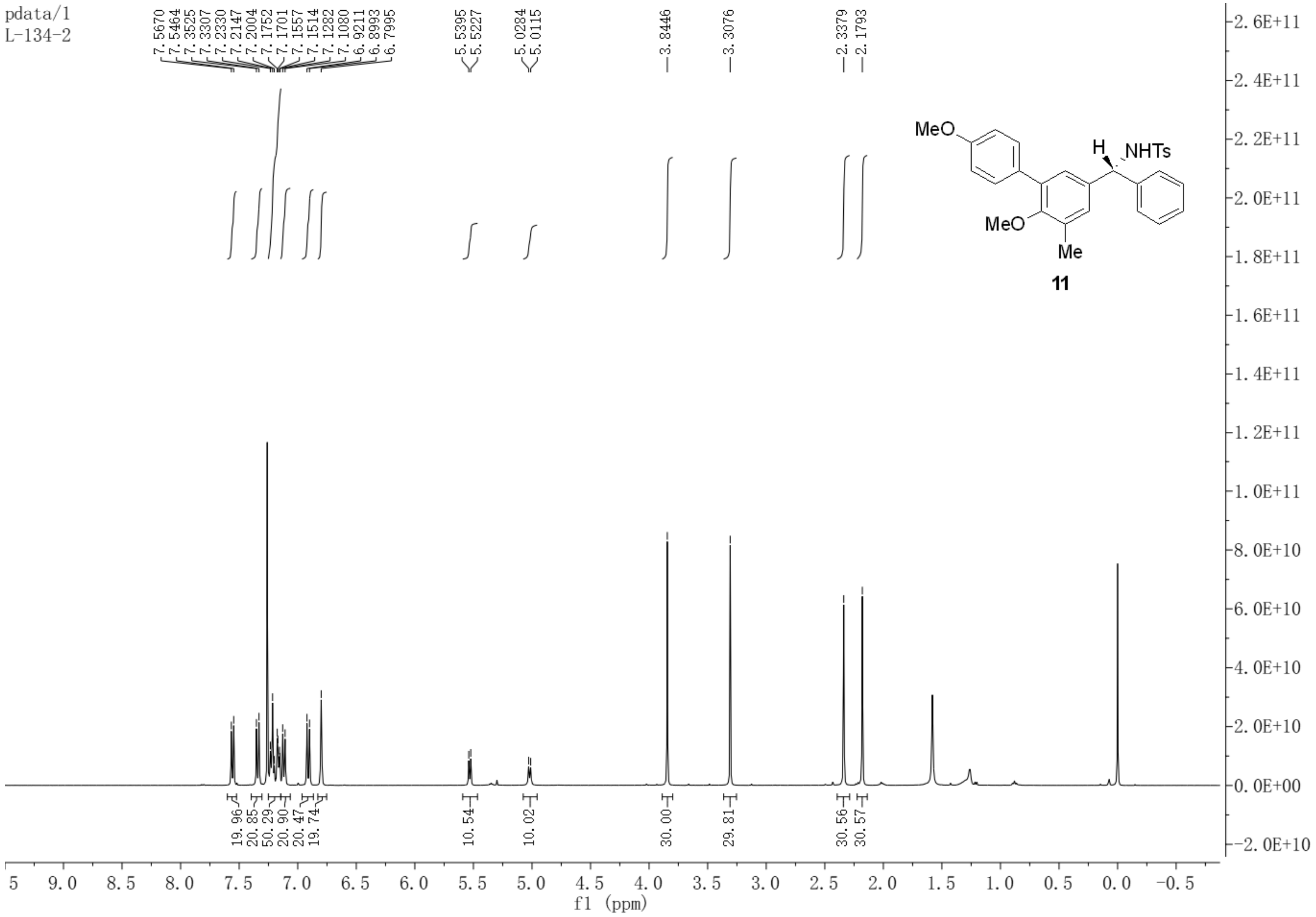

Supplementary Figure 20. ^{1}H NMR spectra of compound **11**

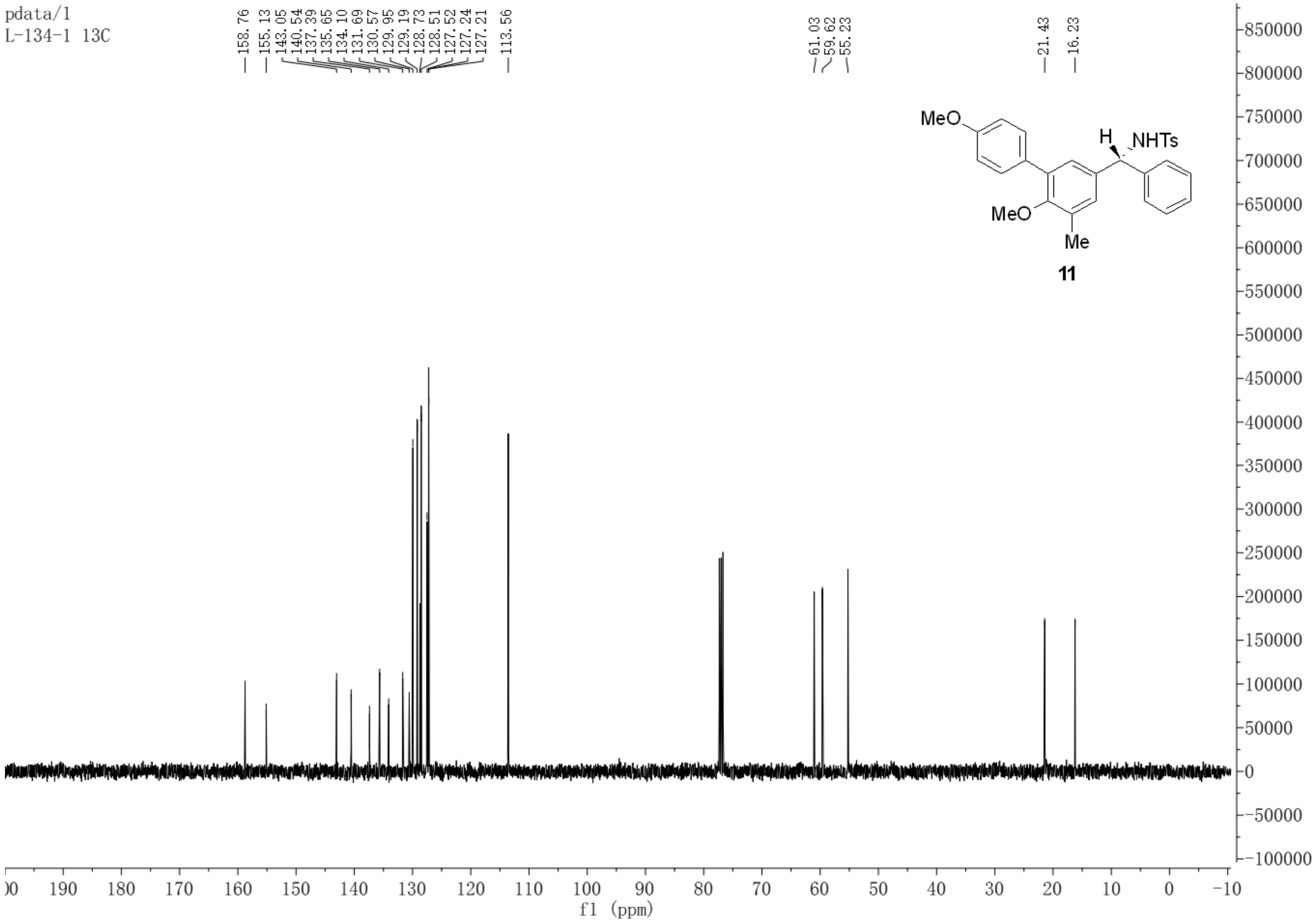

Supplementary Figure 21. ^{13}C NMR spectra of compound **11**

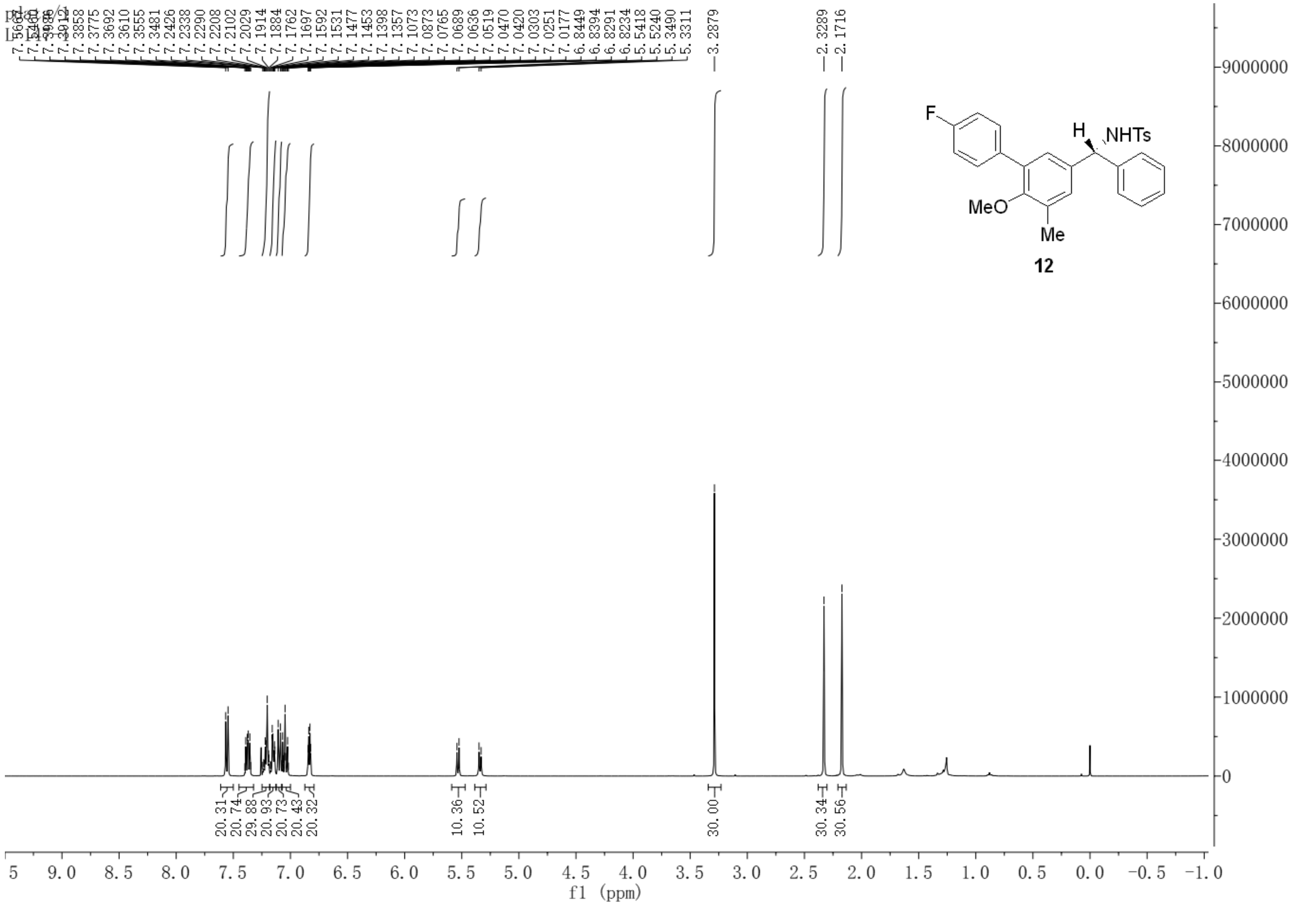

Supplementary Figure 22. ^{1}H NMR spectra of compound **12**

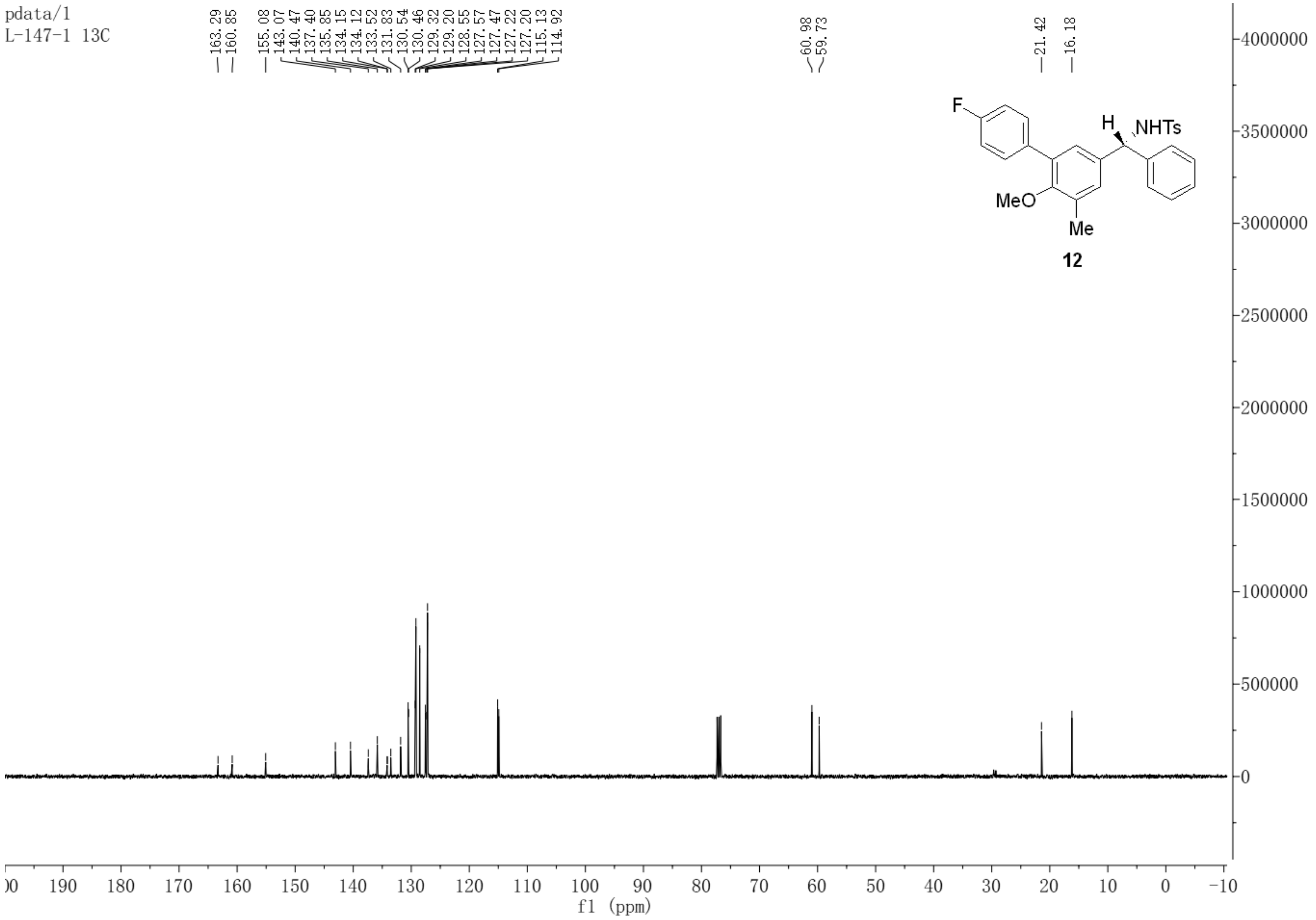

Supplementary Figure 23. ^{13}C NMR spectra of compound **12**

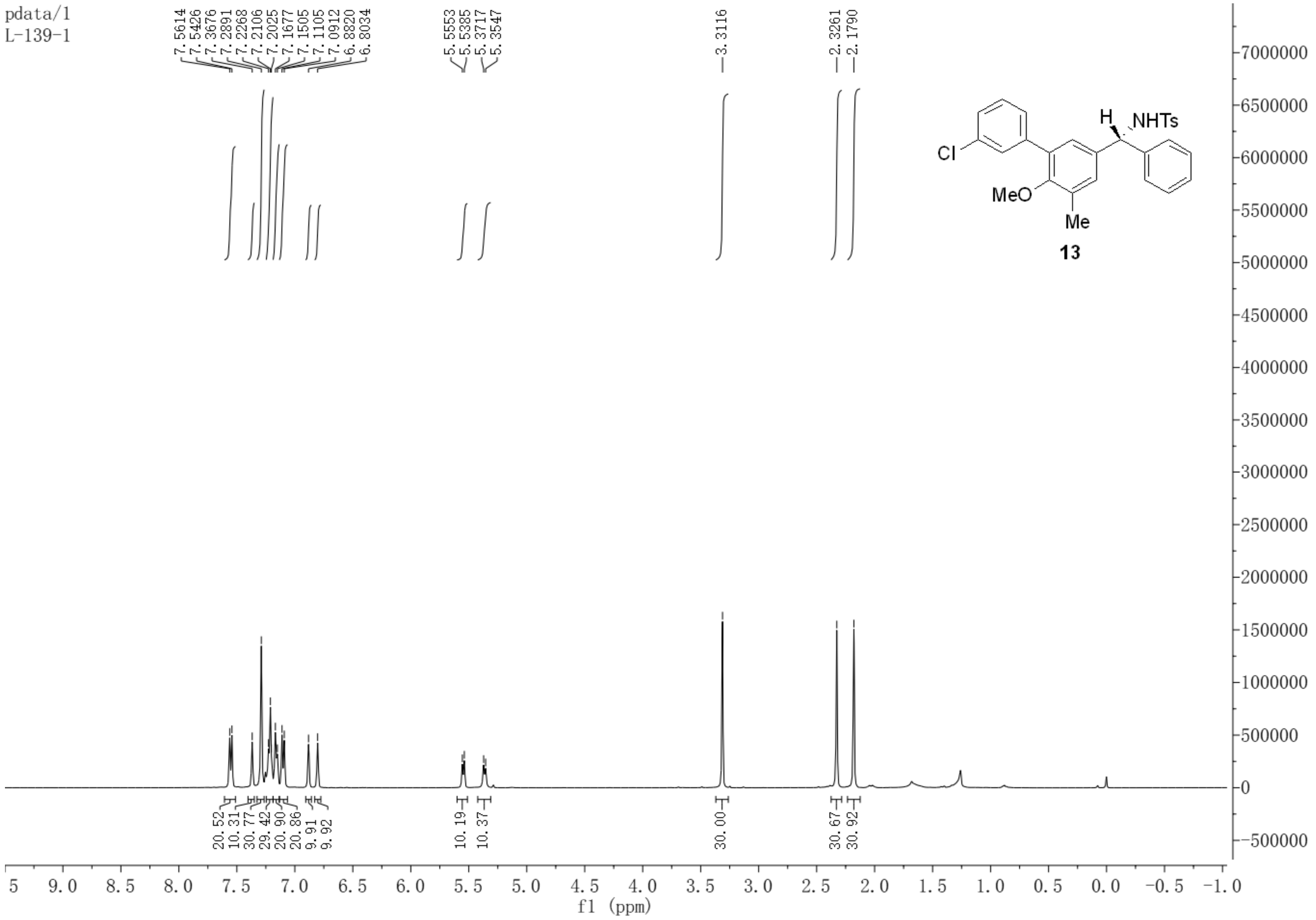

Supplementary Figure 24. ^{1}H NMR spectra of compound **13**

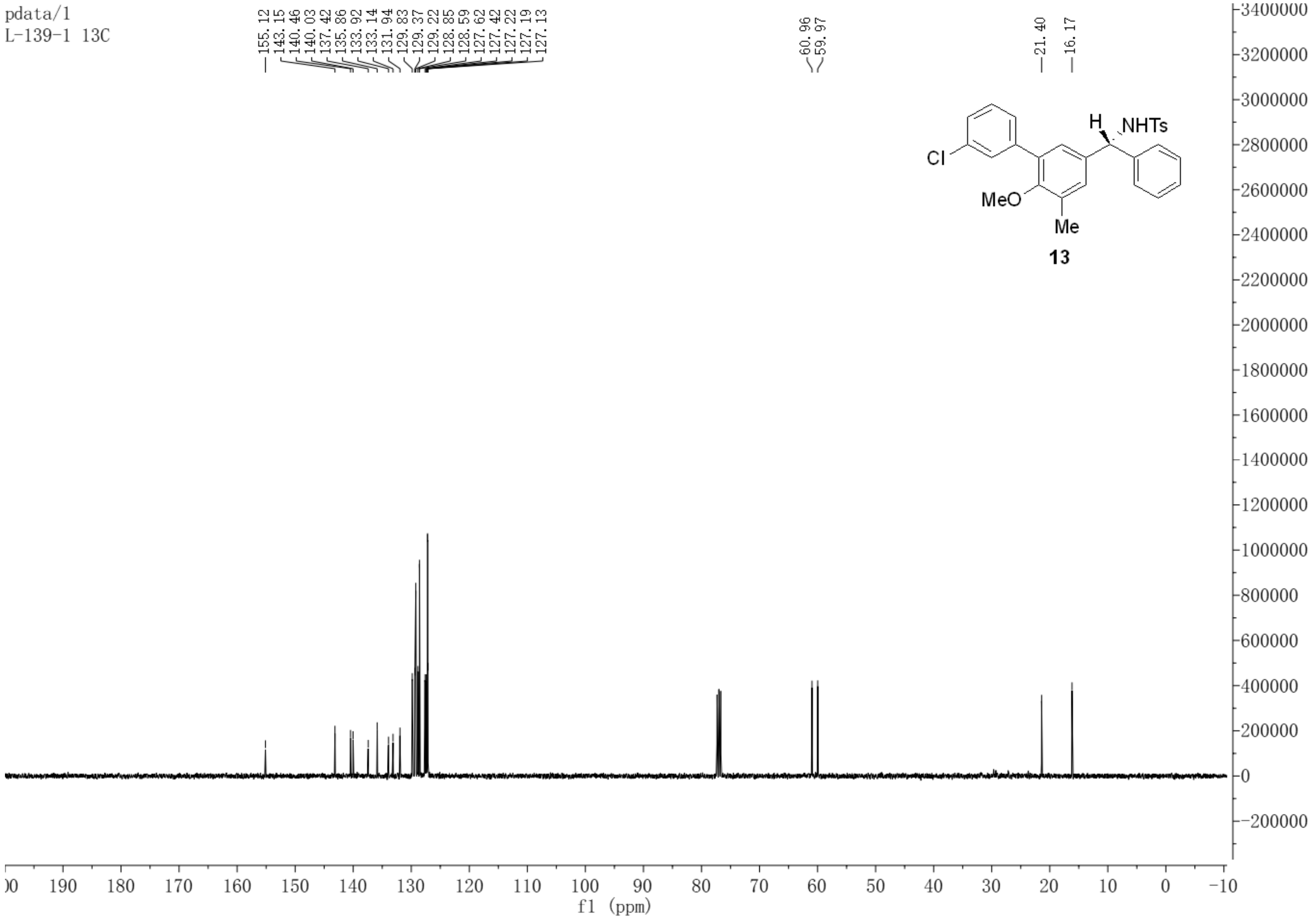

Supplementary Figure 25. ^{13}C NMR spectra of compound **13**

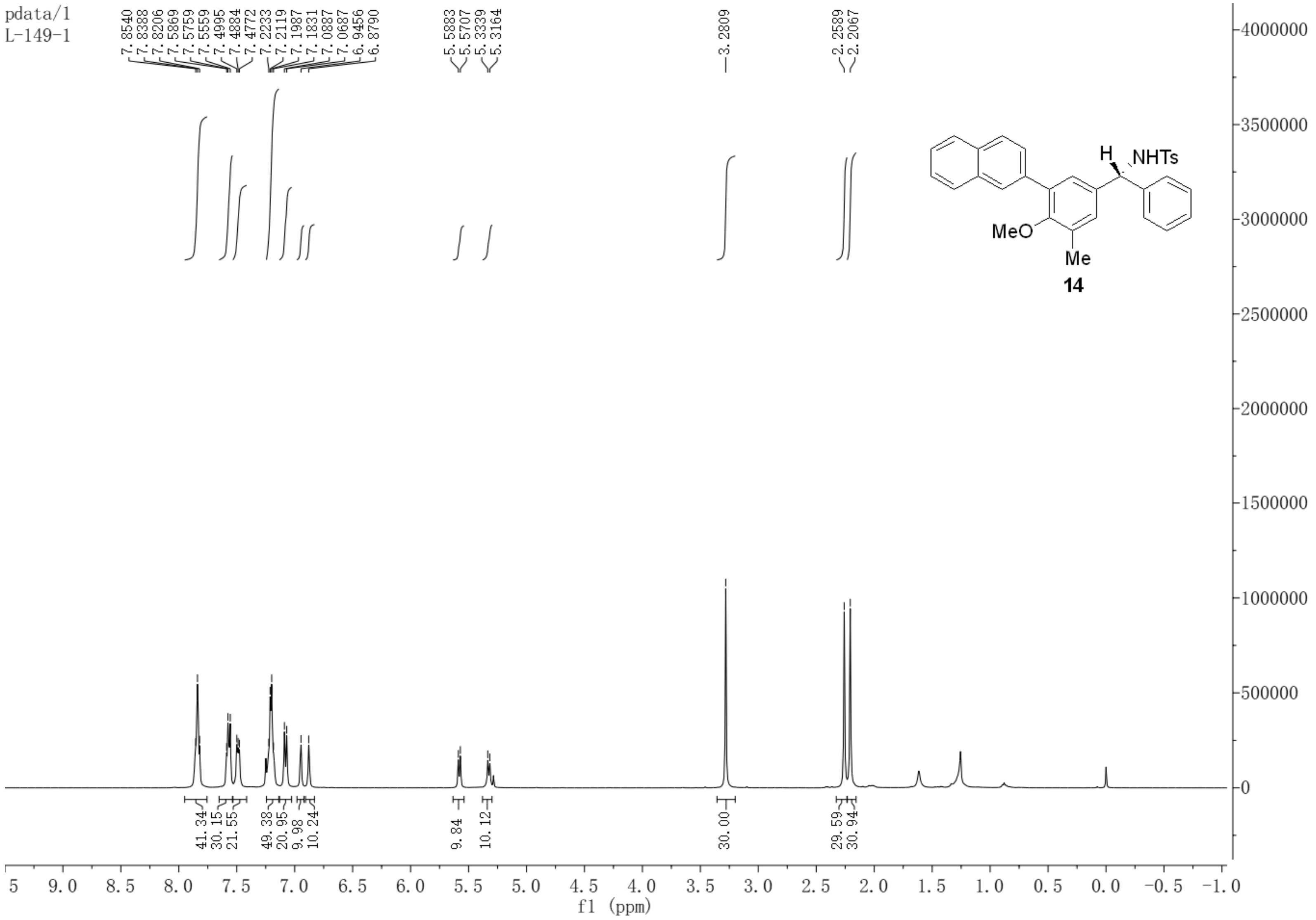

Supplementary Figure 26. ^{1}H NMR spectra of compound **14**

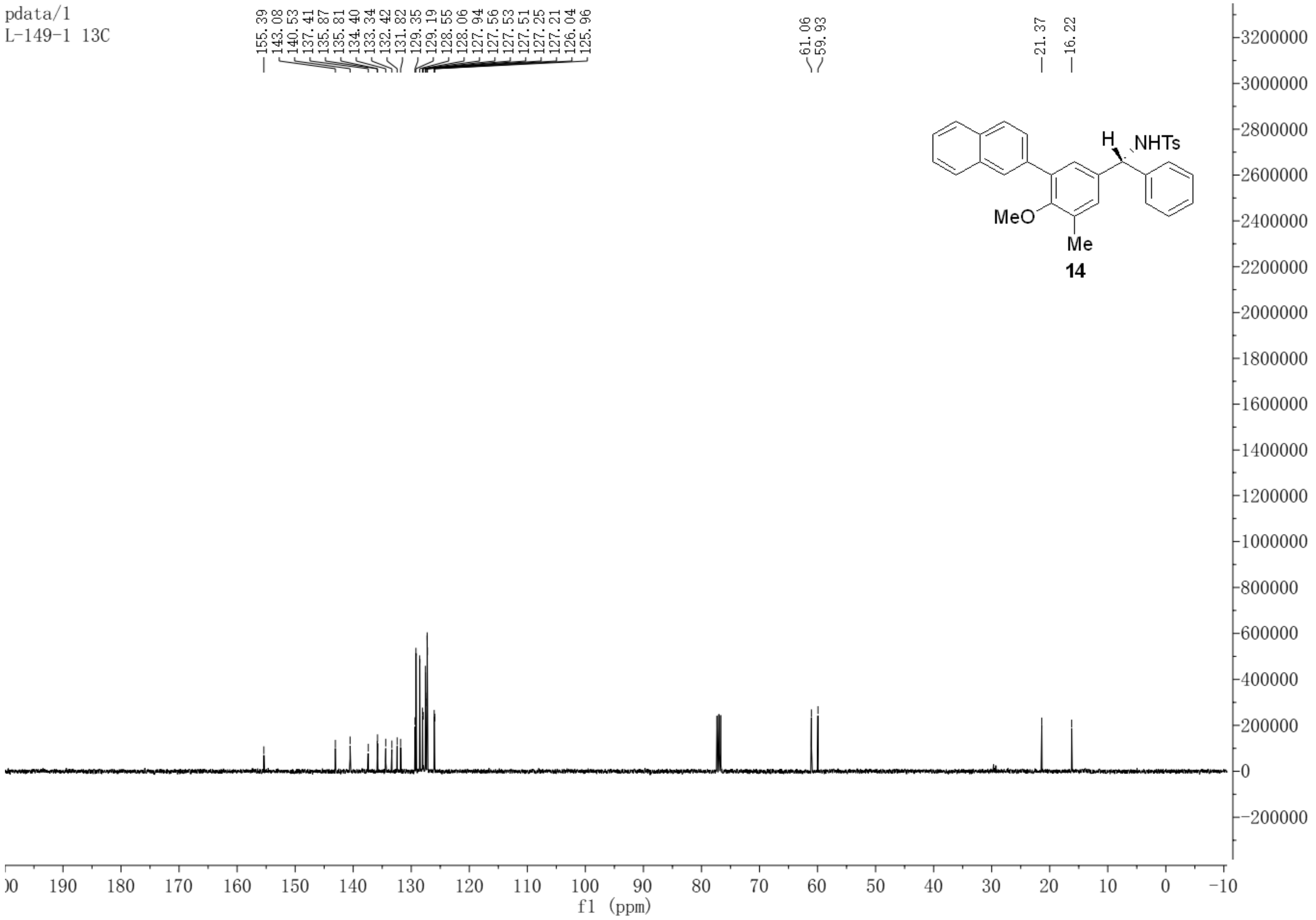

Supplementary Figure 27. ^{13}C NMR spectra of compound **14**

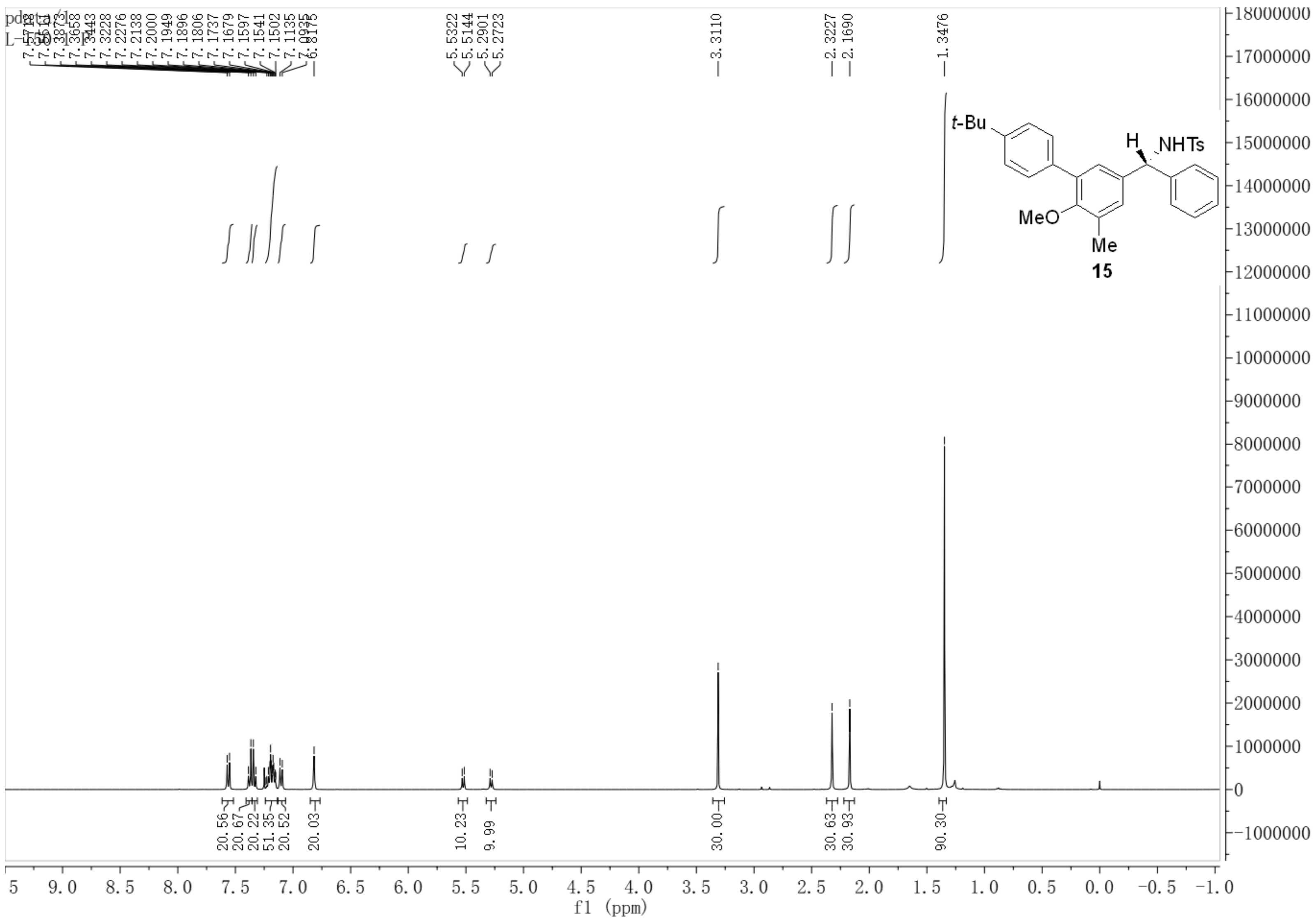

Supplementary Figure 28. ^{1}H NMR spectra of compound **15**

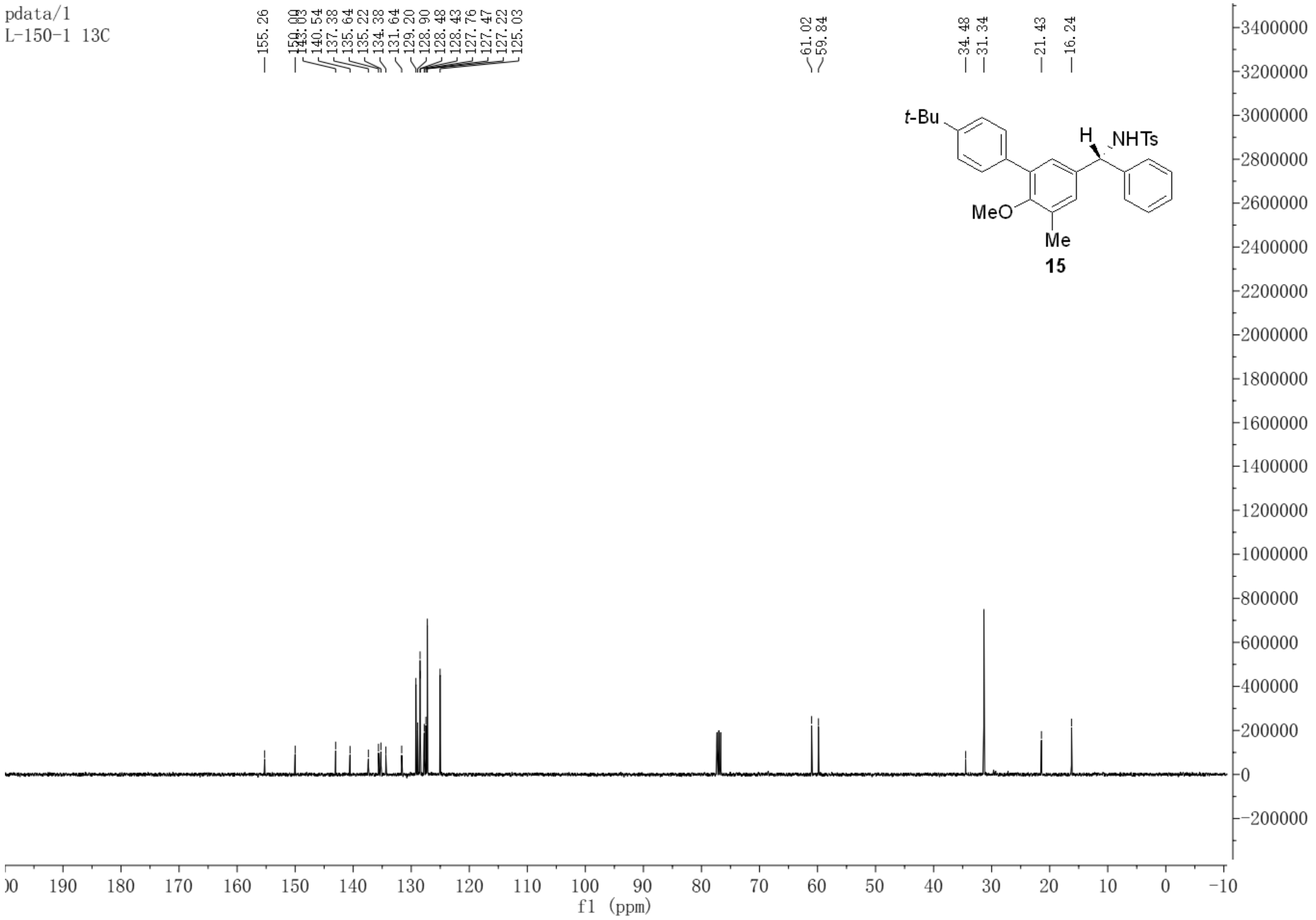

Supplementary Figure 29. ^{13}C NMR spectra of compound **15**

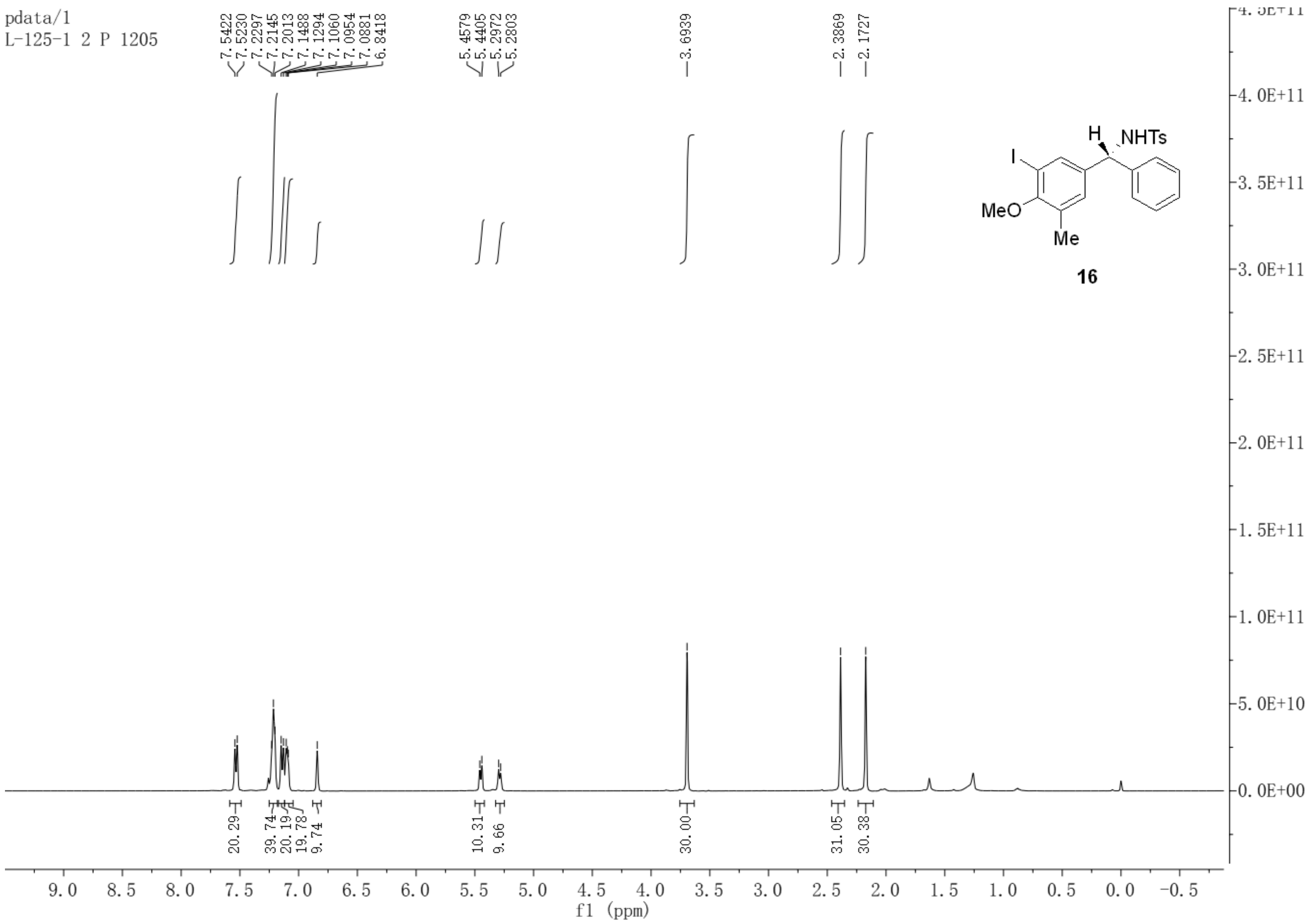

16

Supplementary Figure 30. ^{1}H NMR spectra of compound **16**

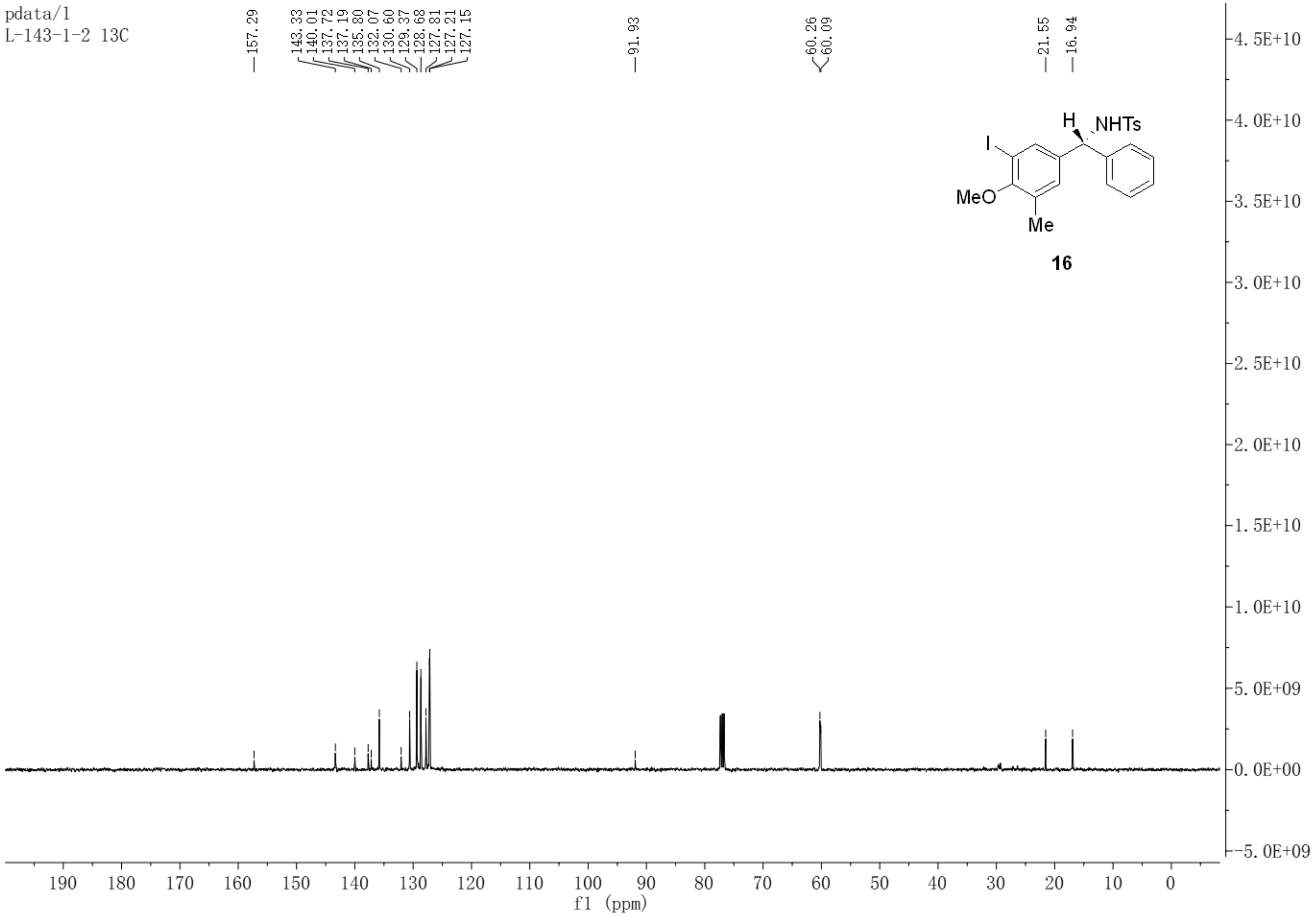

Supplementary Figure 31. ^{13}C NMR spectra of compound **16**

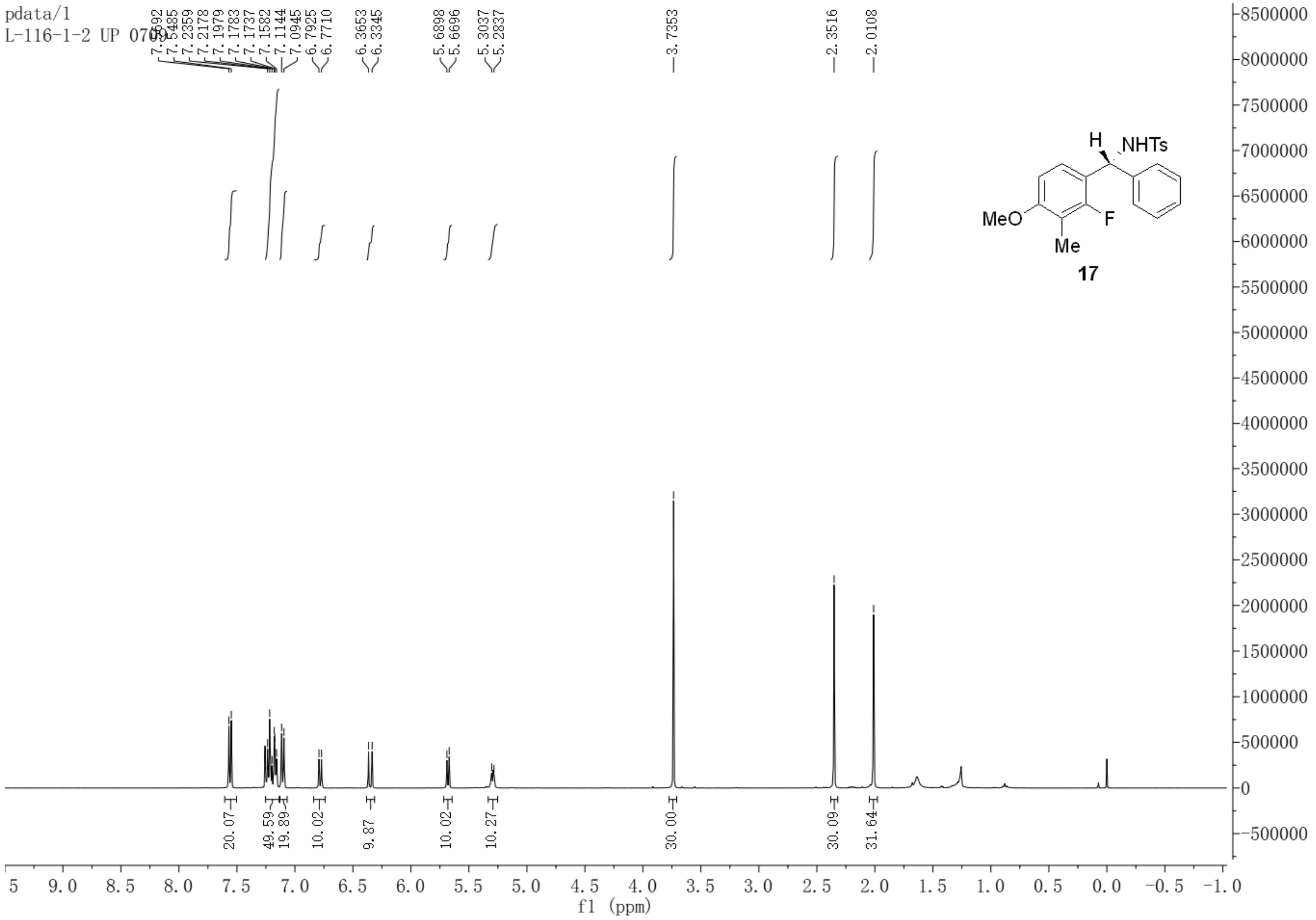

Supplementary Figure 32. ^{1}H NMR spectra of compound **17**

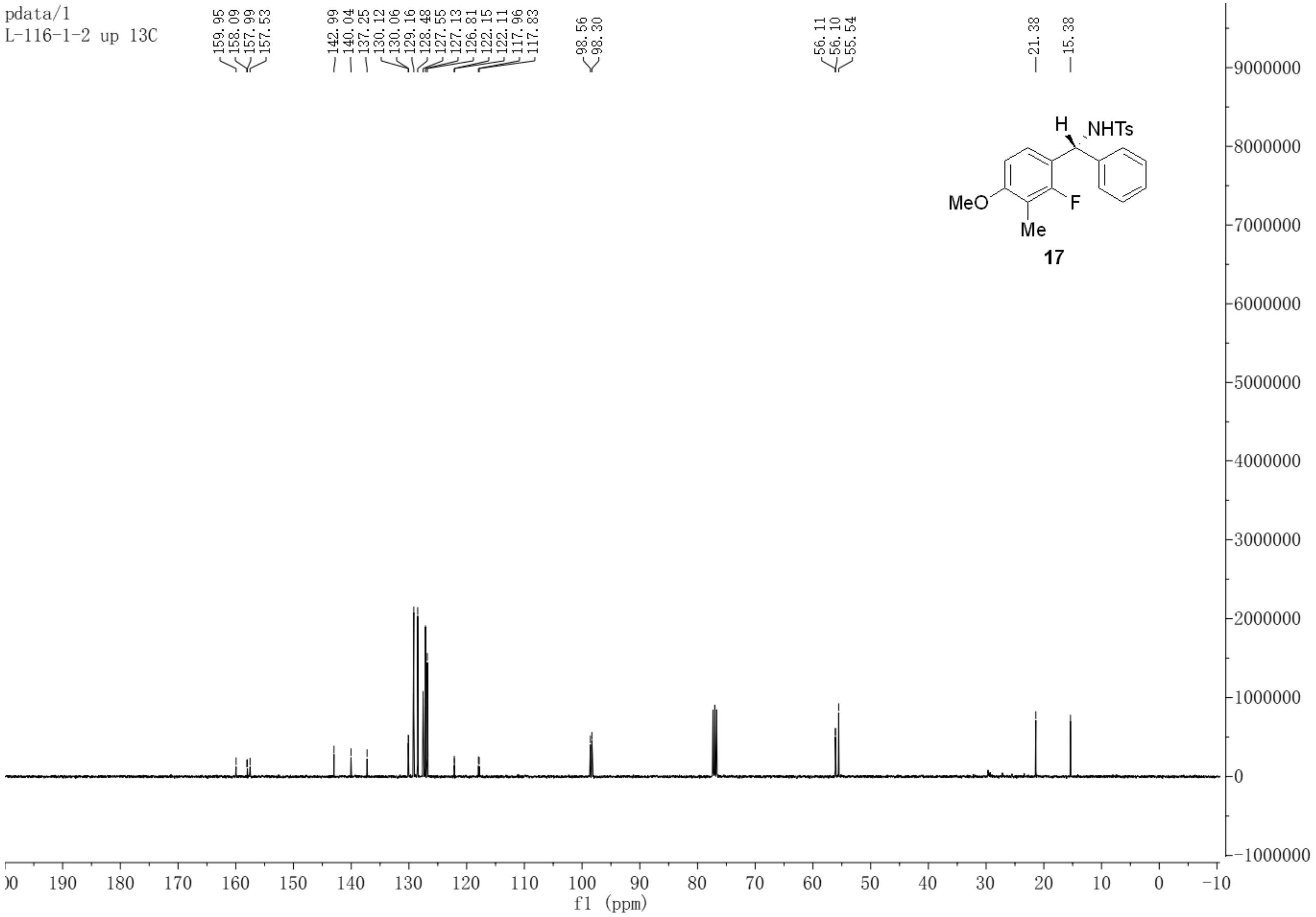

Supplementary Figure 33. ^{13}C NMR spectra of compound **17**

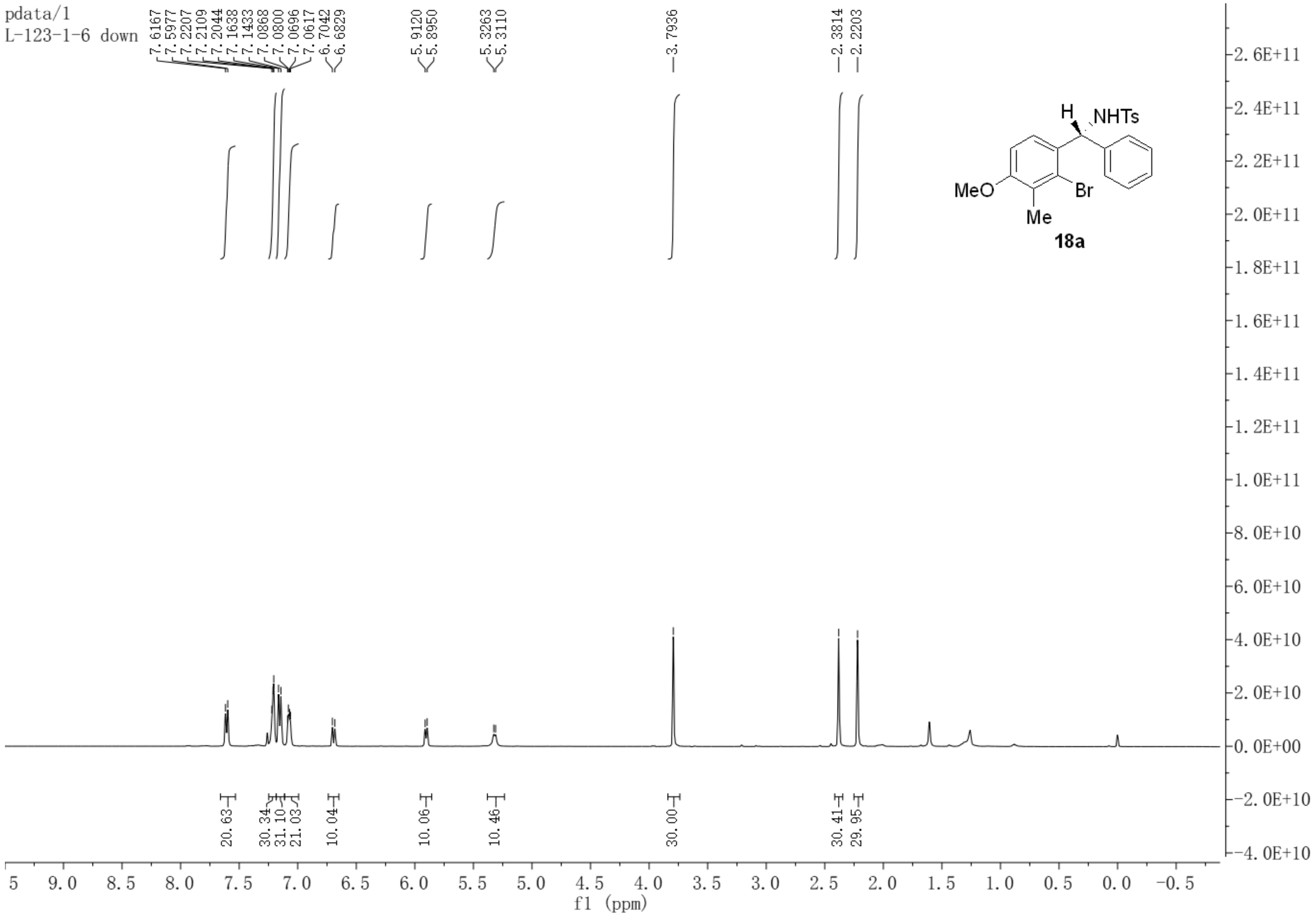

Supplementary Figure 34. ^{1}H NMR spectra of compound **18a**

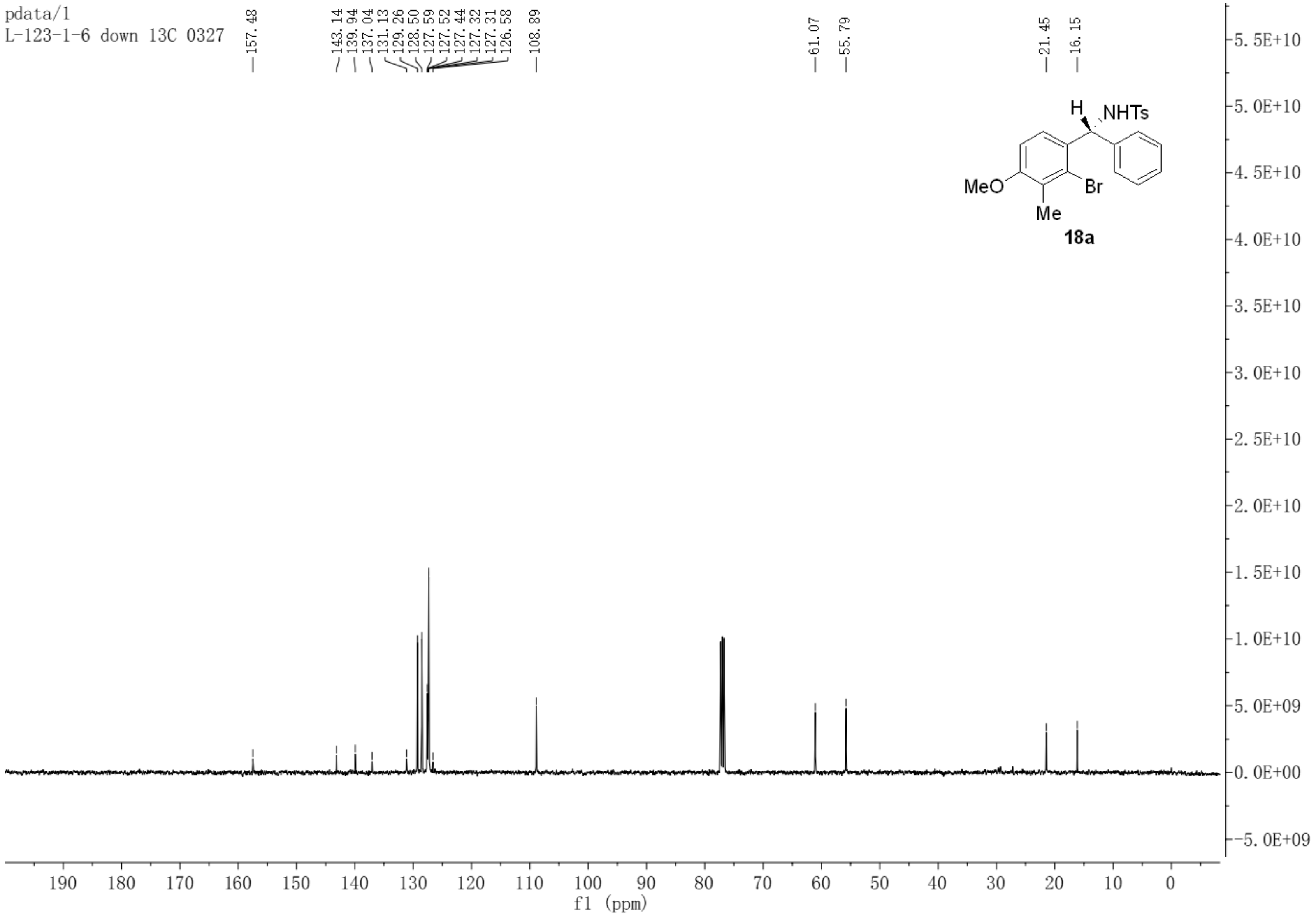

Supplementary Figure 35. ^{13}C NMR spectra of compound **18a**

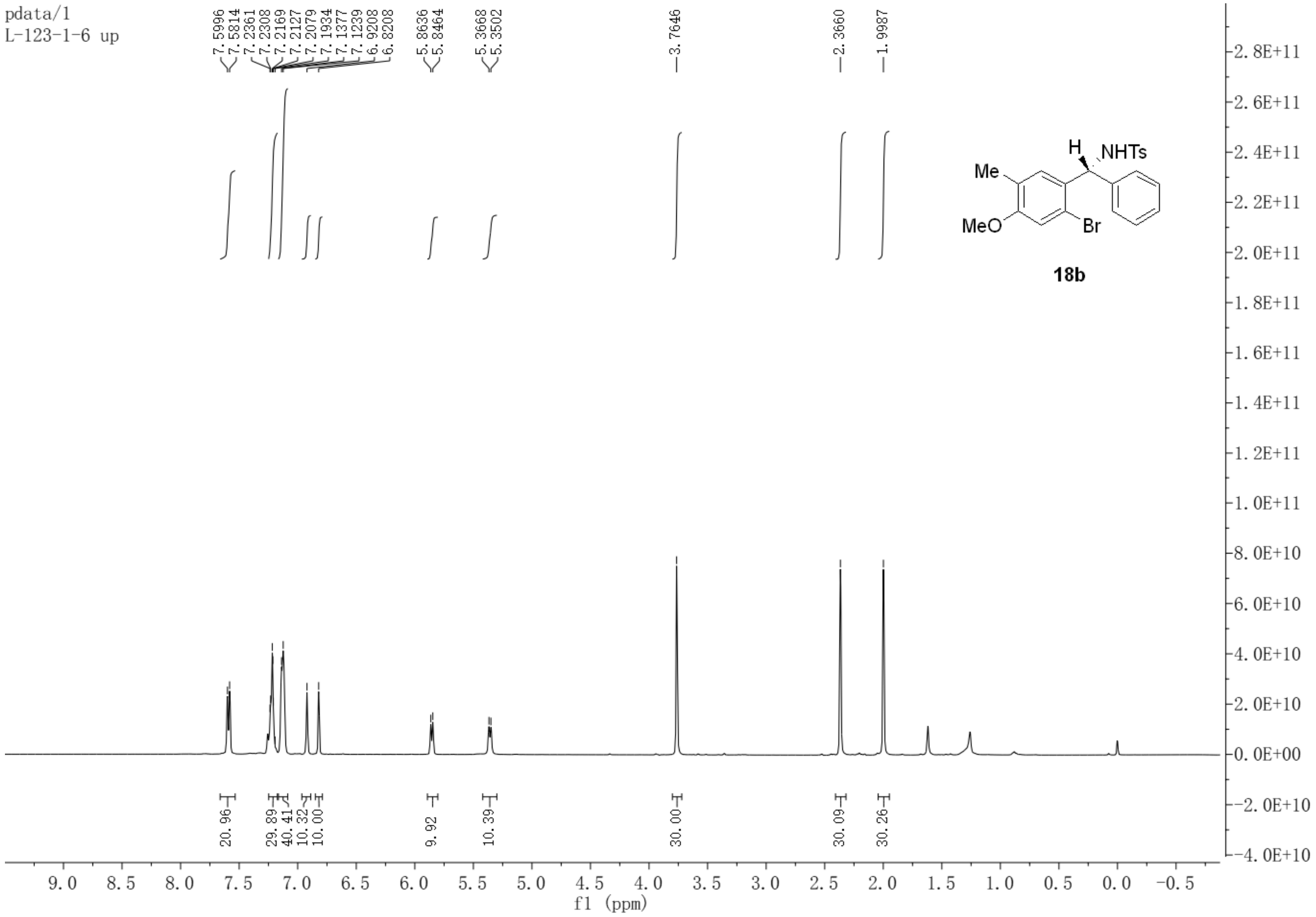

Supplementary Figure 36. ^{1}H NMR spectra of compound **18b**

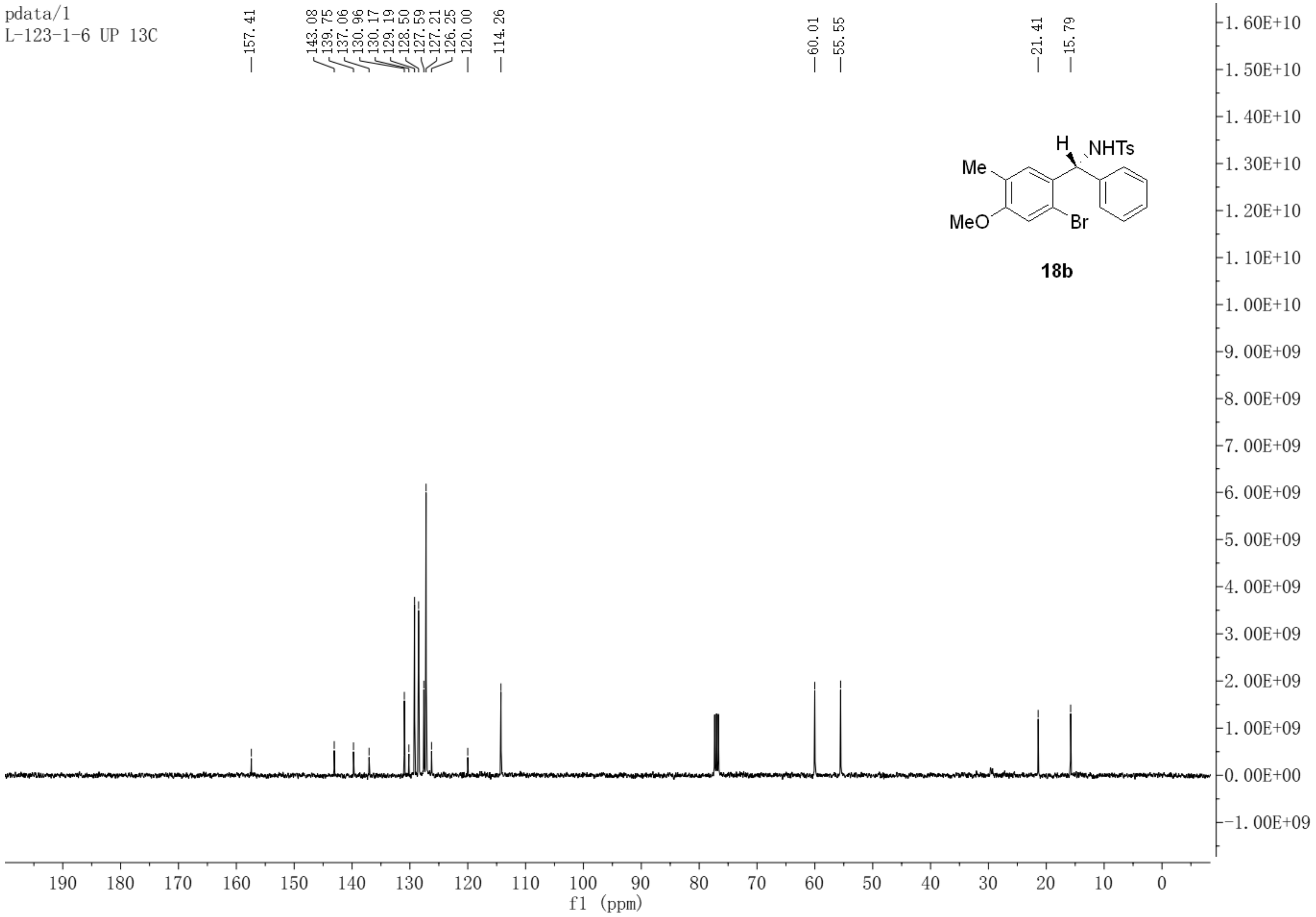

Supplementary Figure 37. ^{13}C NMR spectra of compound **18b**

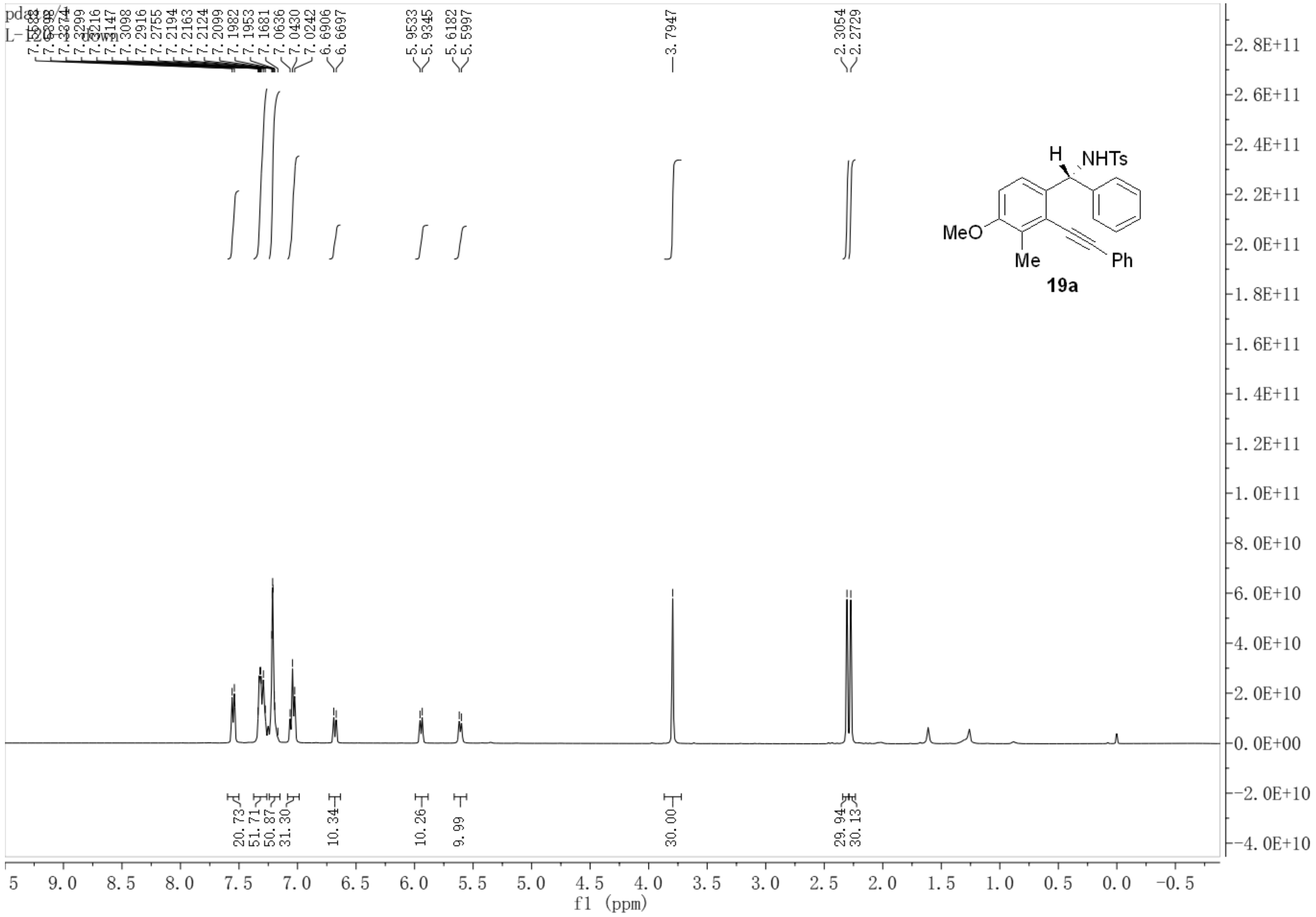

Supplementary Figure 38. ^{1}H NMR spectra of compound **19a**

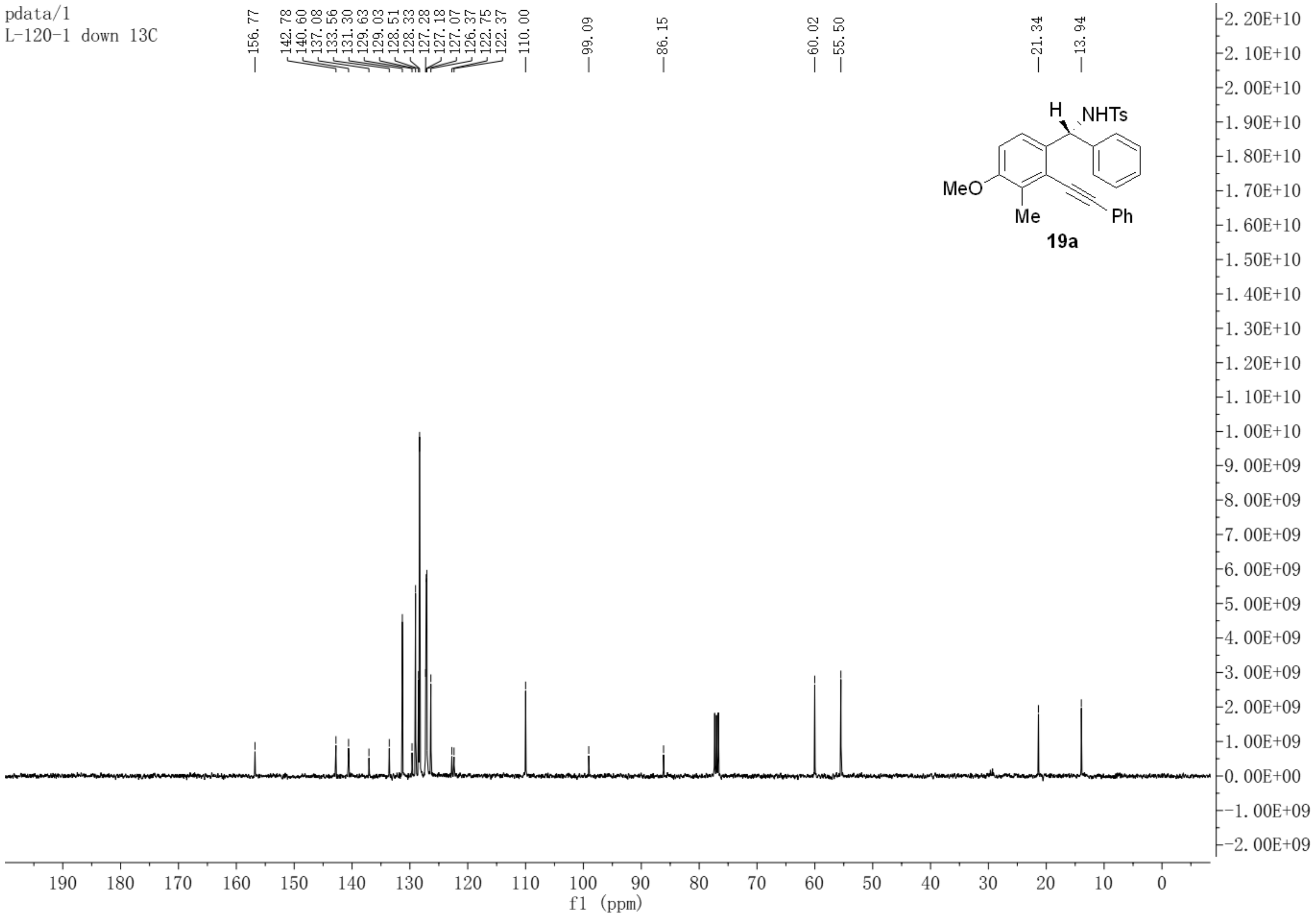

Supplementary Figure 39. ^{13}C NMR spectra of compound **19a**

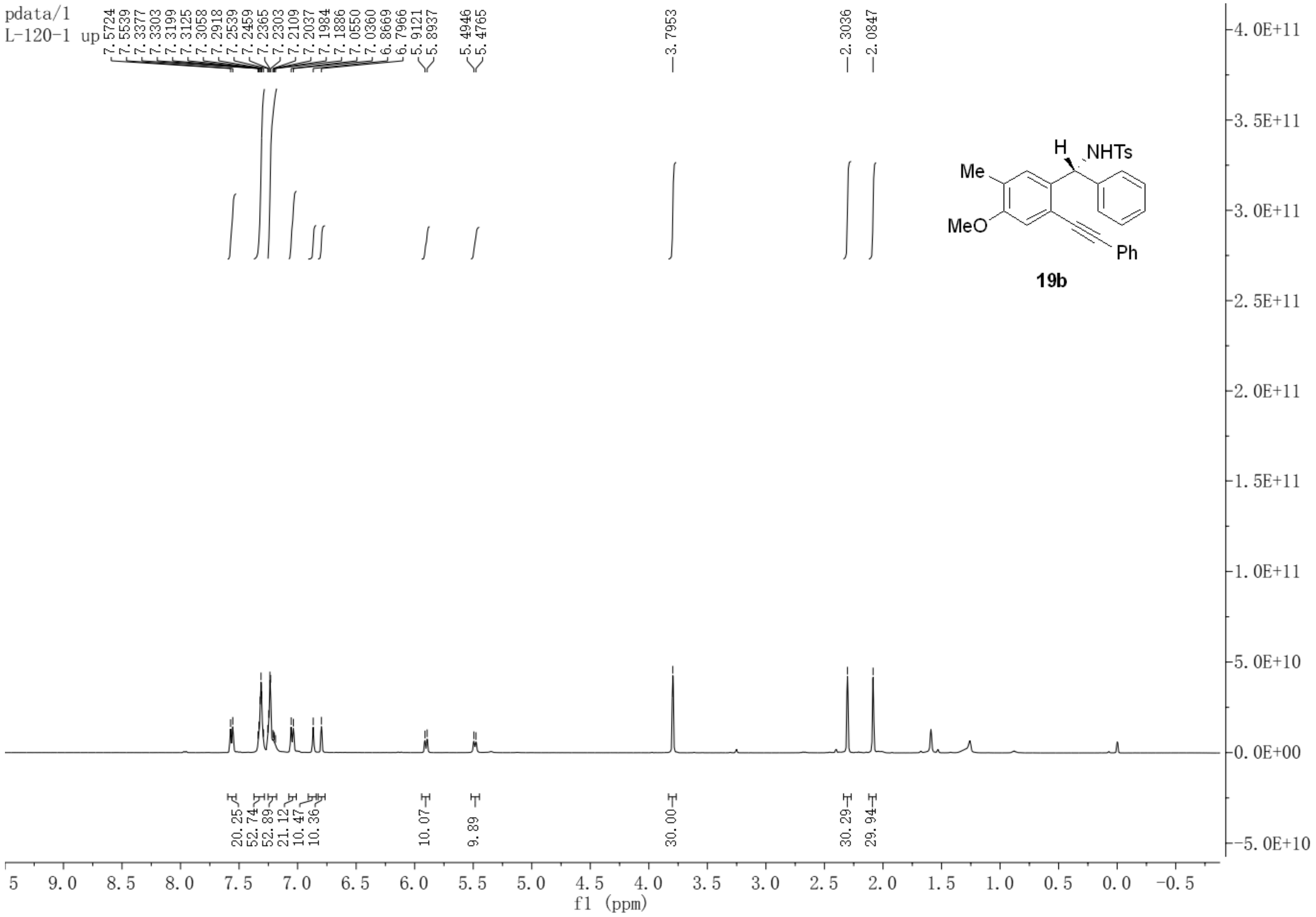

Supplementary Figure 40. ^{1}H NMR spectra of compound **19b**

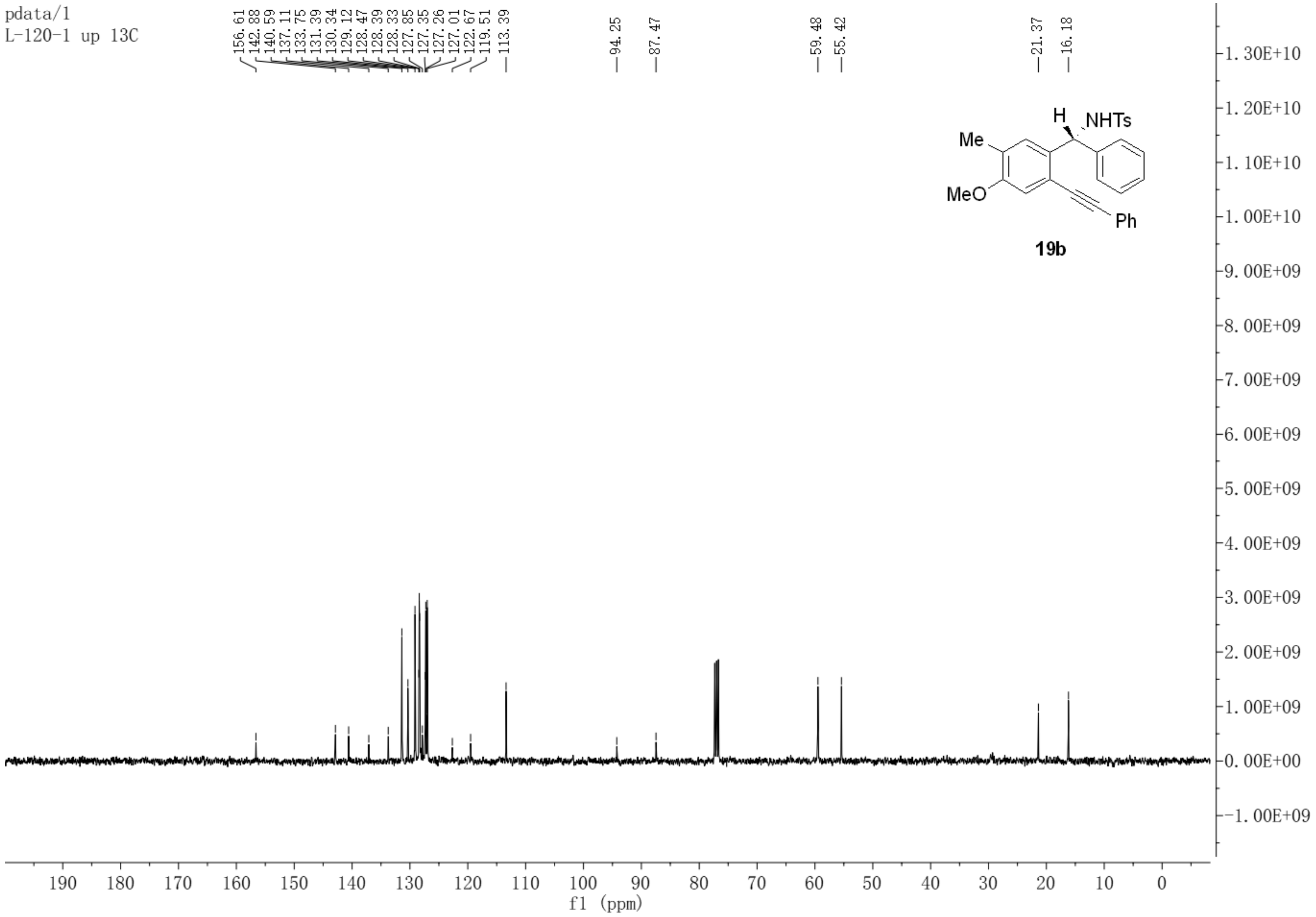

Supplementary Figure 41. ^{13}C NMR spectra of compound **19b**

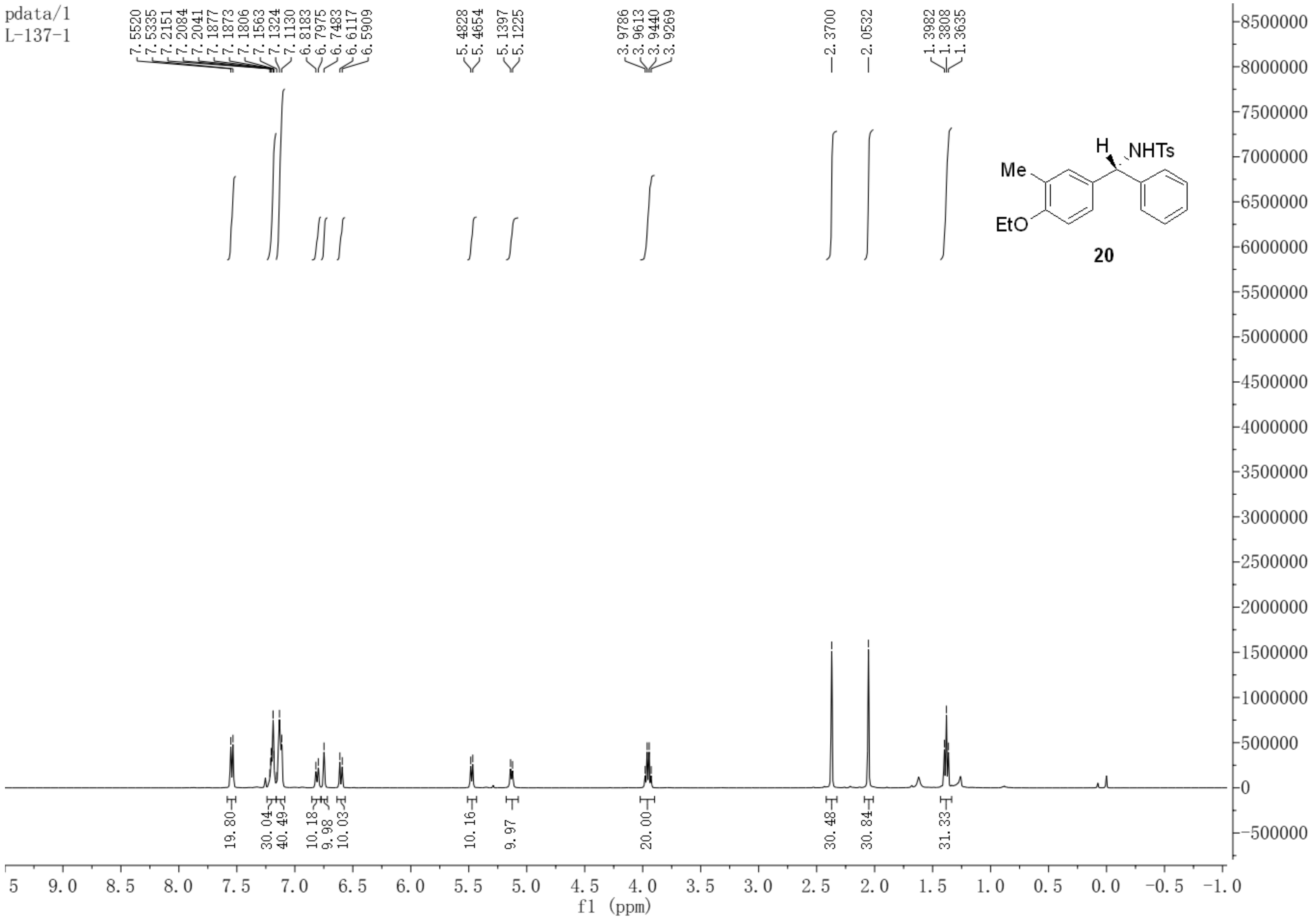

Supplementary Figure 42. ^{1}H NMR spectra of compound **20**

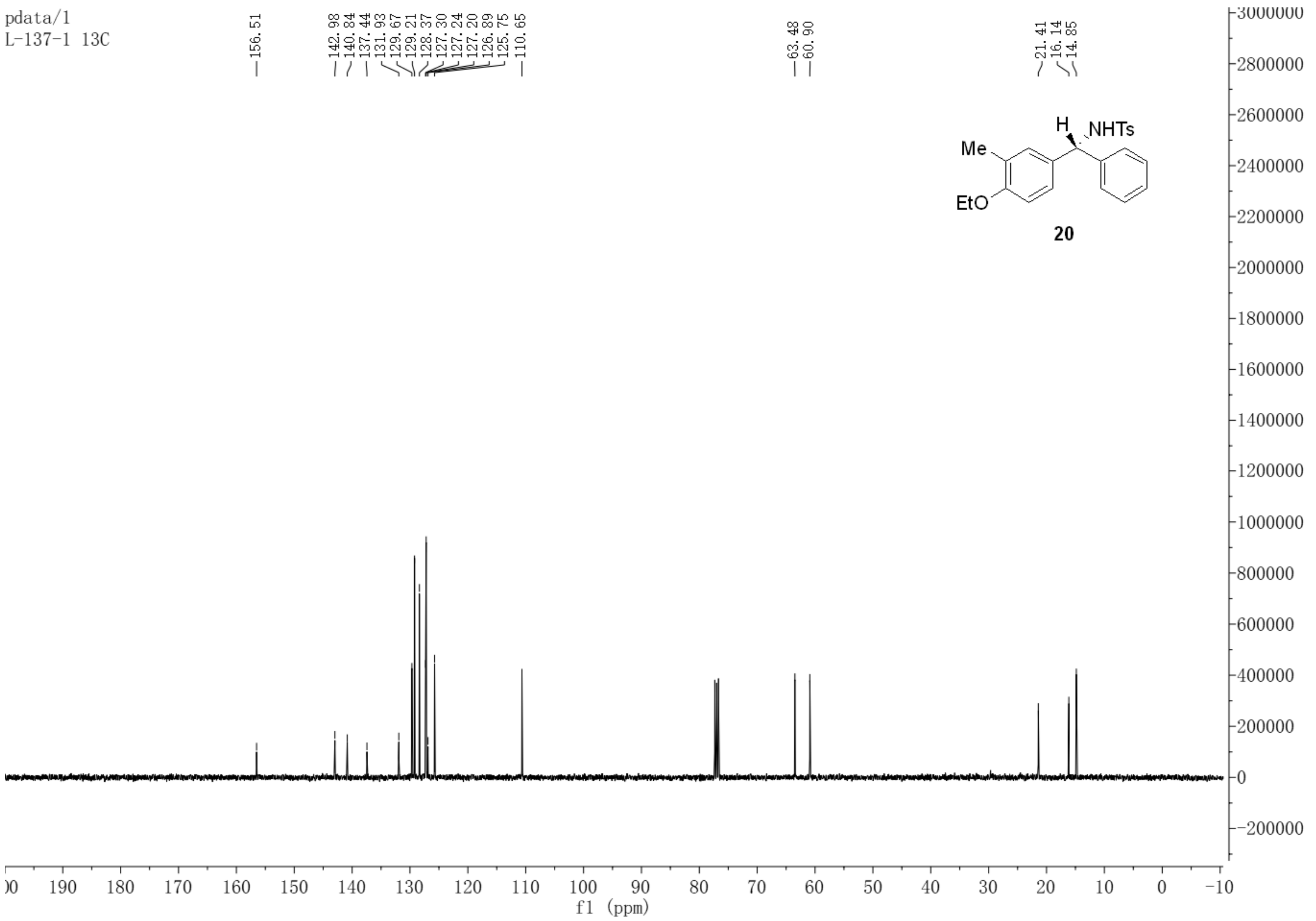

Supplementary Figure 43. ^{13}C NMR spectra of compound **20**

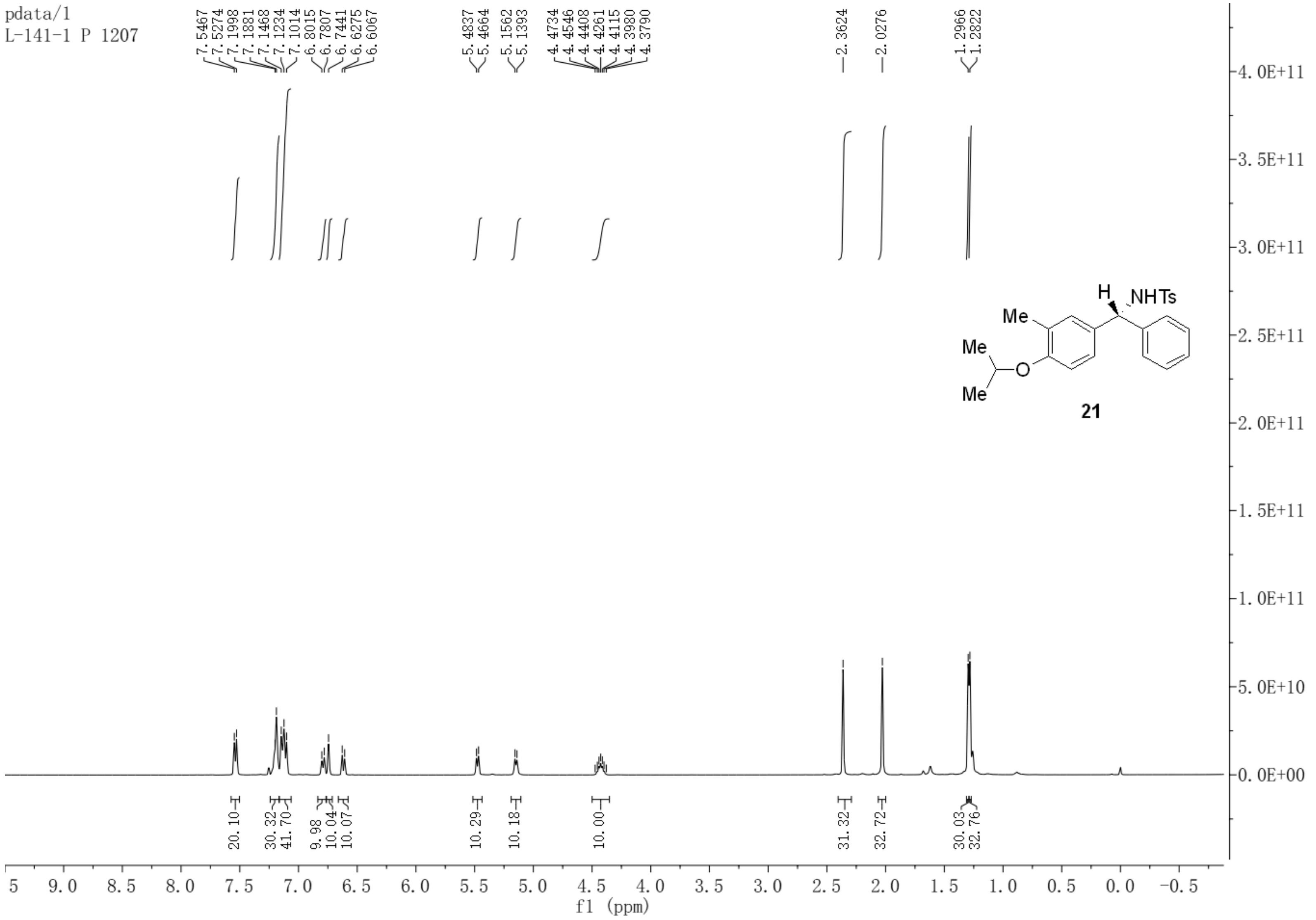

Supplementary Figure 44. ^{1}H NMR spectra of compound **21**

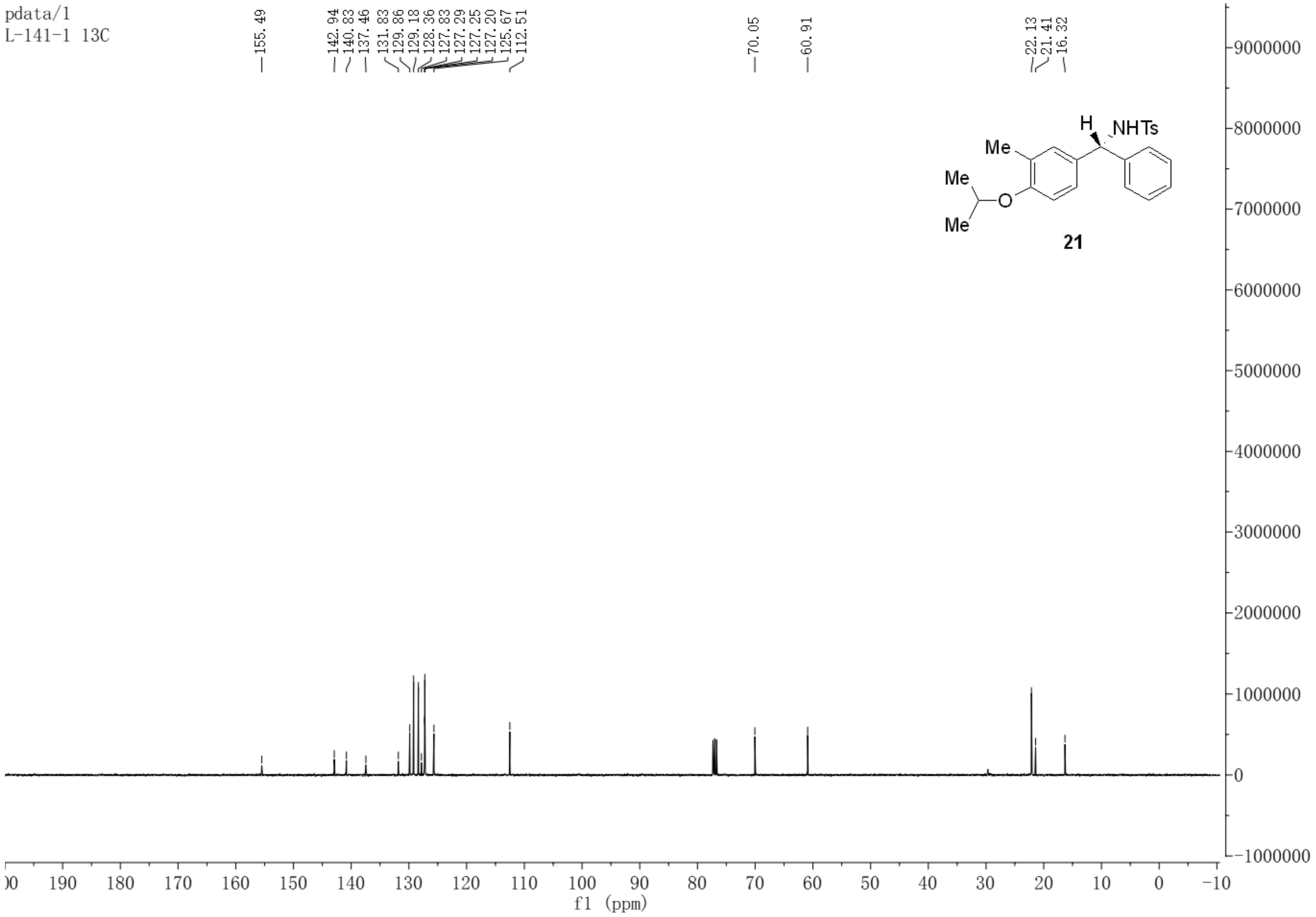

Supplementary Figure 45. ^{13}C NMR spectra of compound **21**

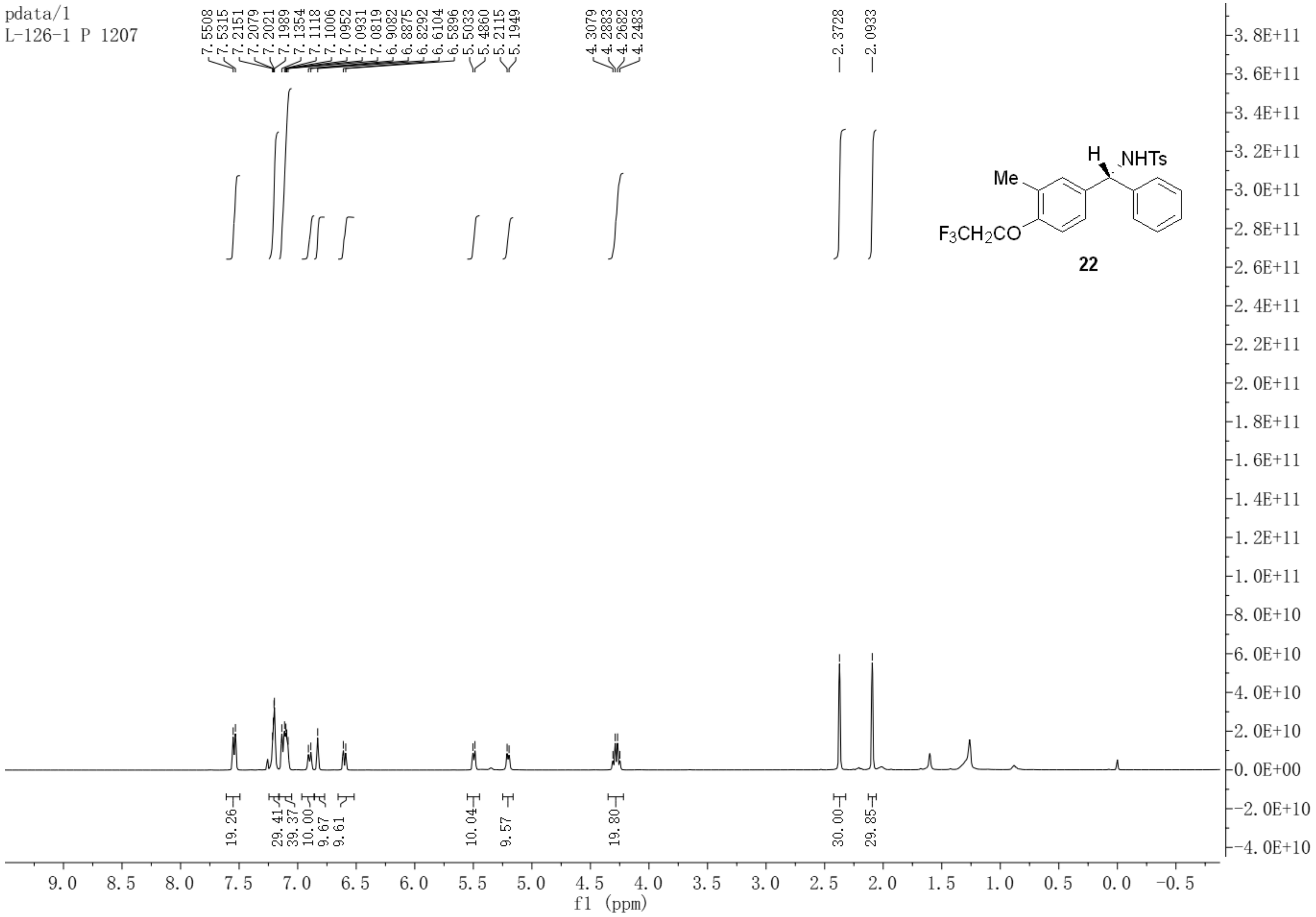

Supplementary Figure 46. ^{1}H NMR spectra of compound **22**

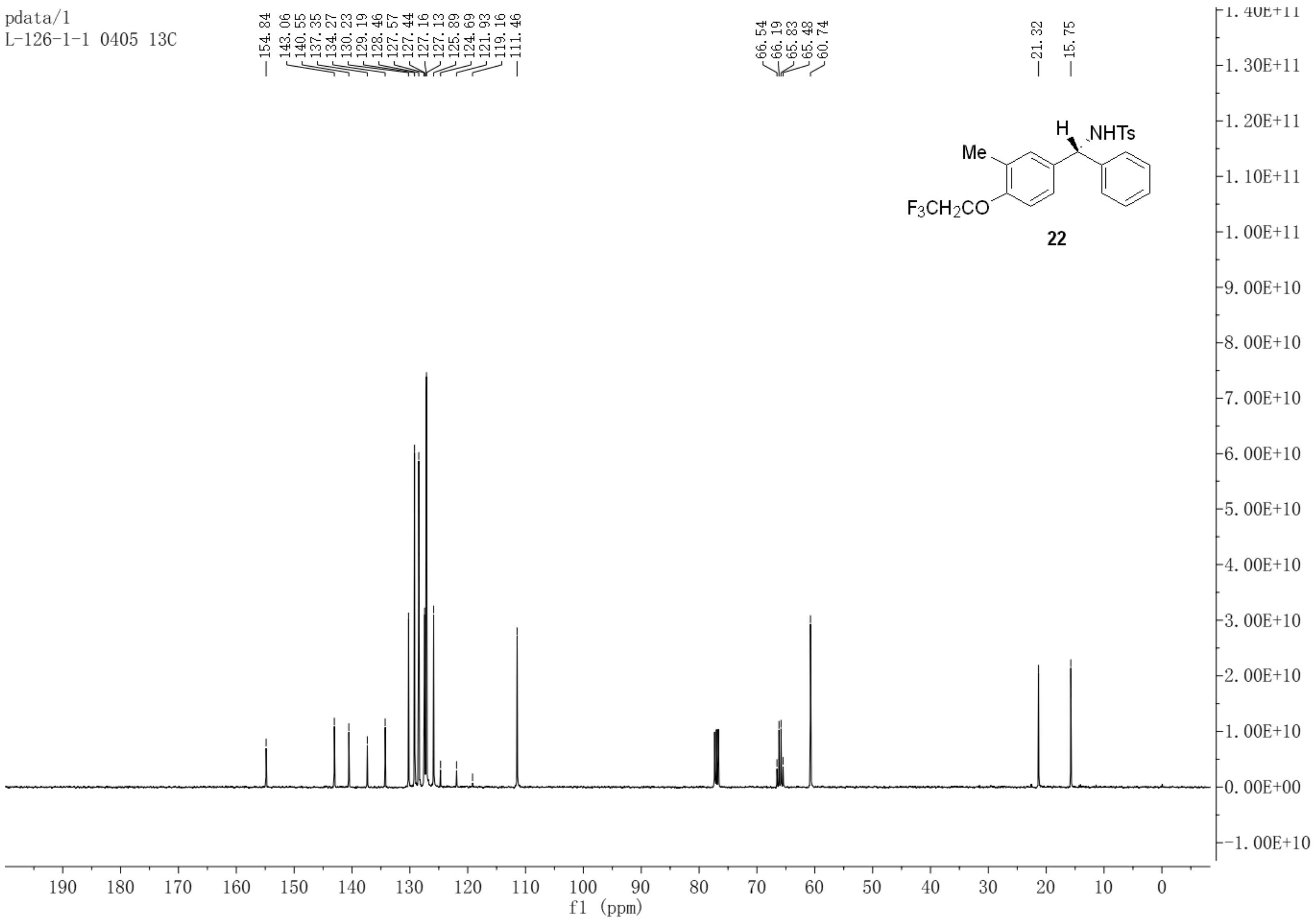

Supplementary Figure 47. ^{13}C NMR spectra of compound **22**

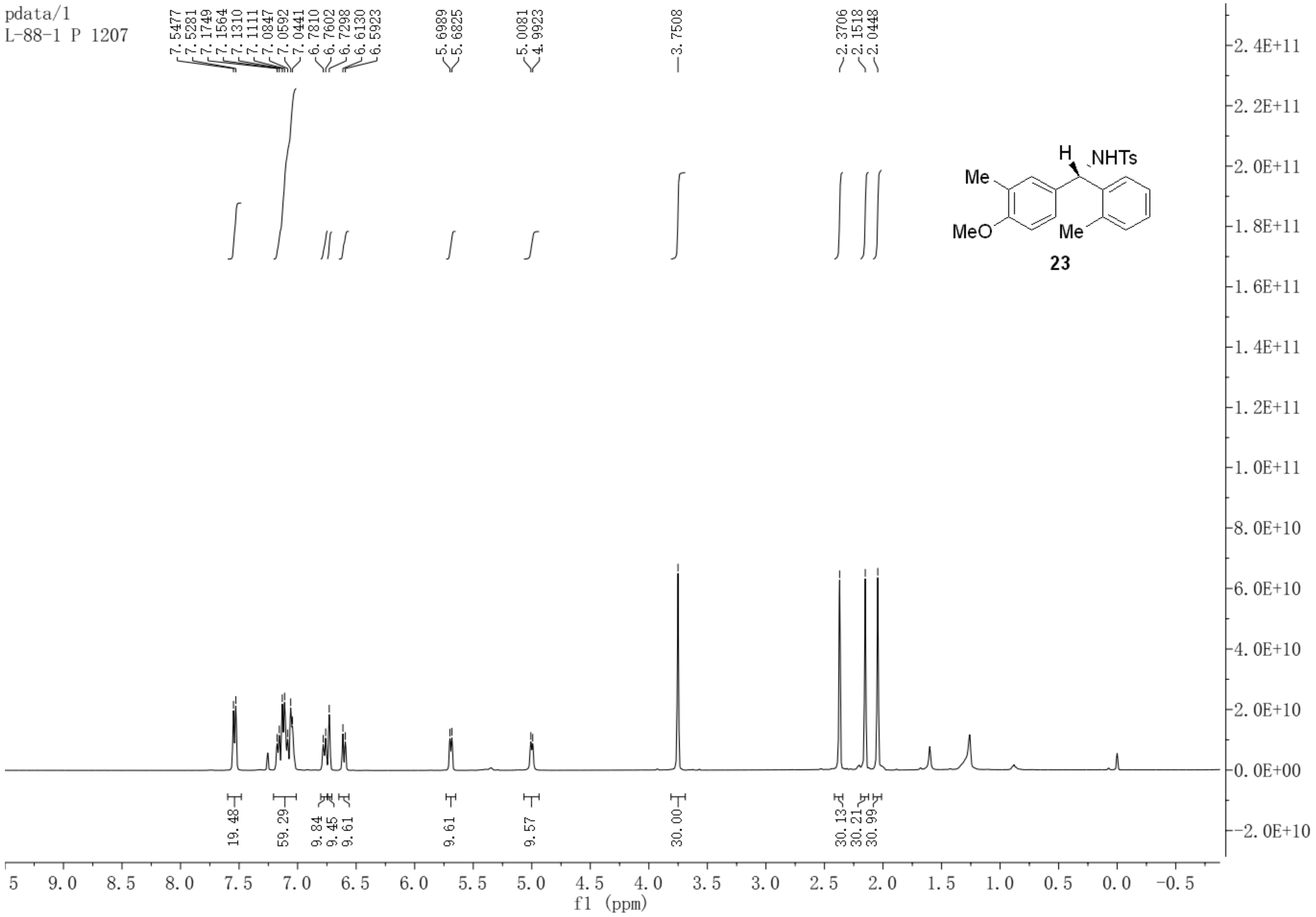

Supplementary Figure 48. ^{1}H NMR spectra of compound **23**

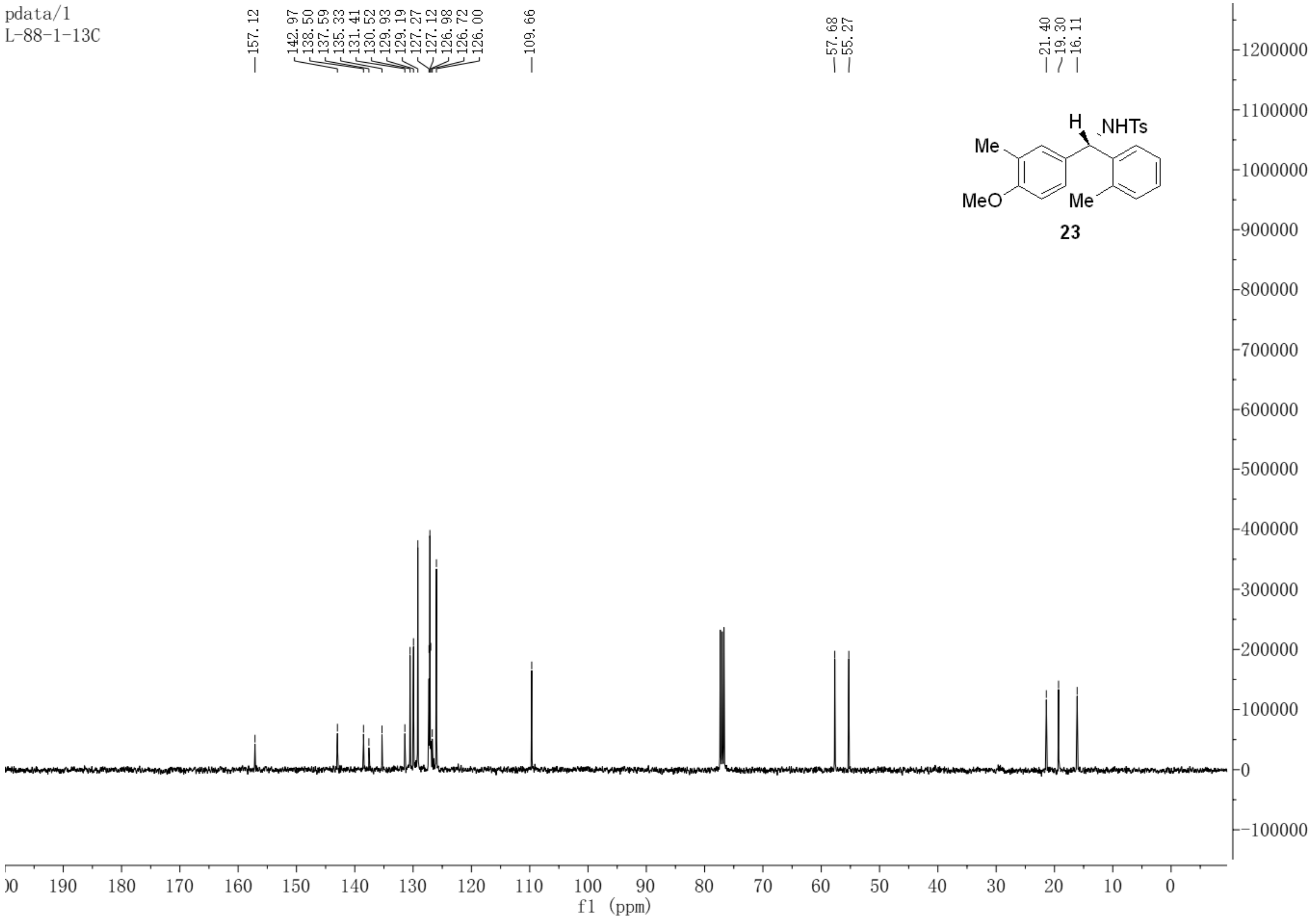

Supplementary Figure 49. ^{13}C NMR spectra of compound **23**

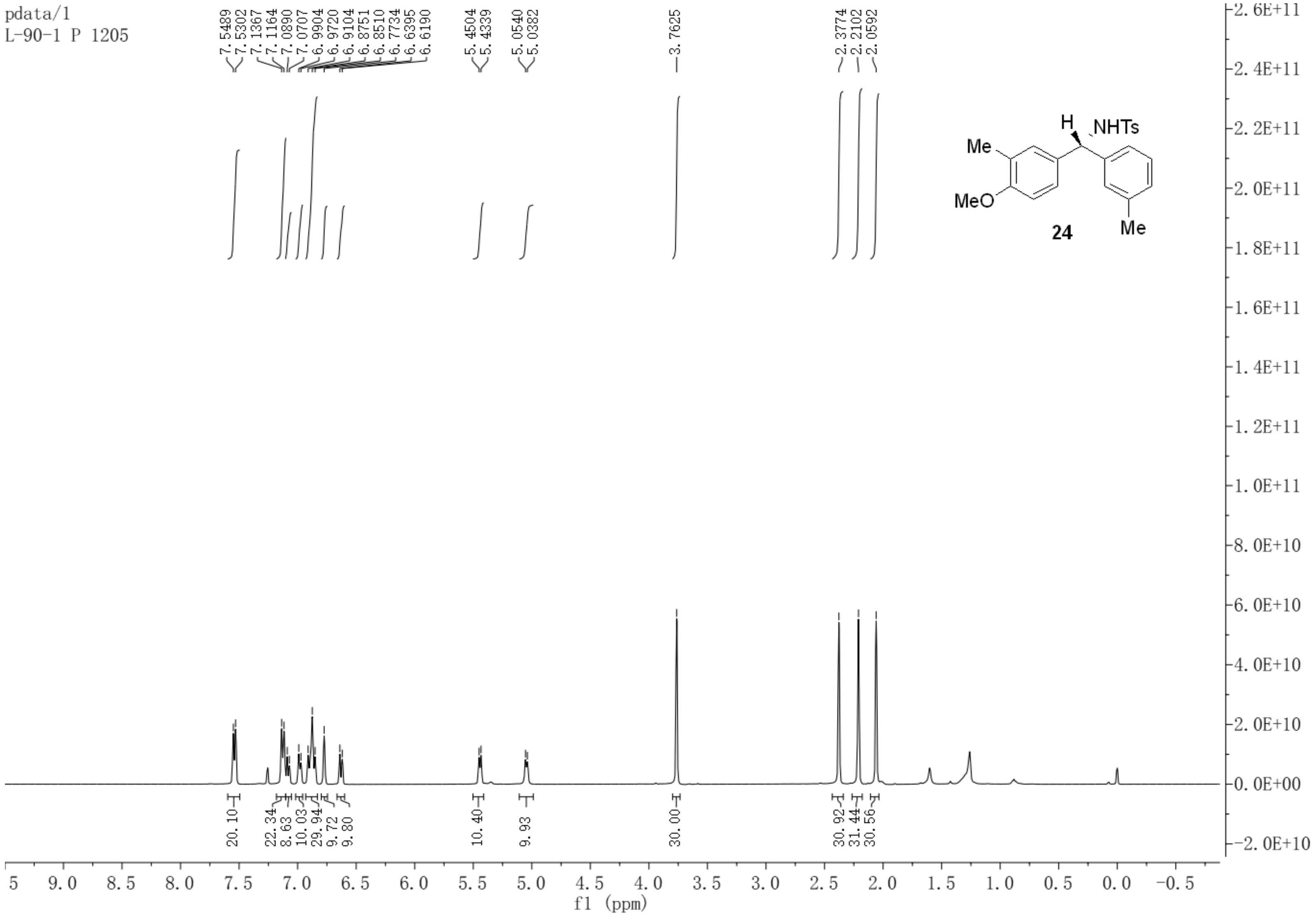

Supplementary Figure 50. ^{1}H NMR spectra of compound **24**

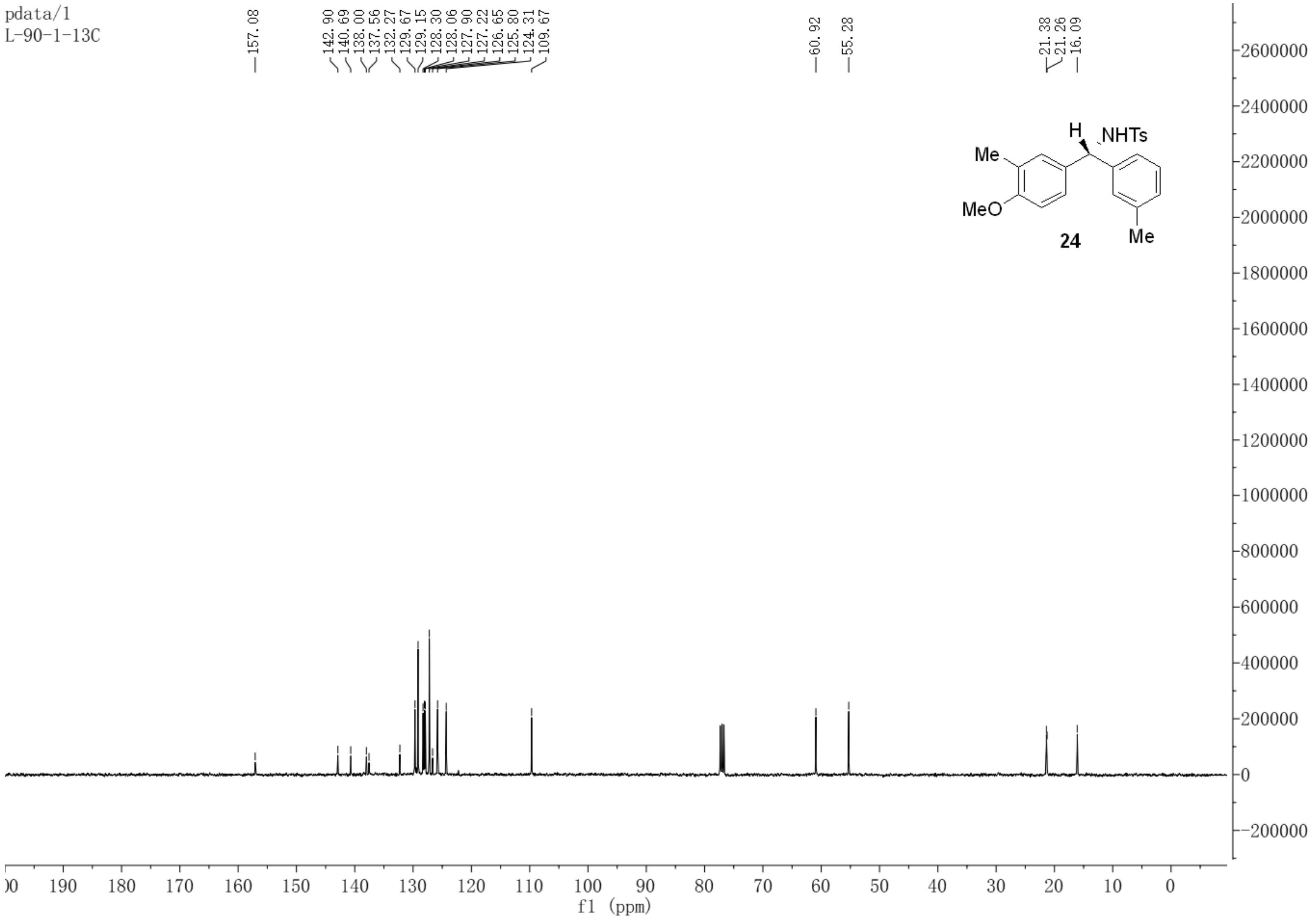

Supplementary Figure 51. ^{13}C NMR spectra of compound **24**

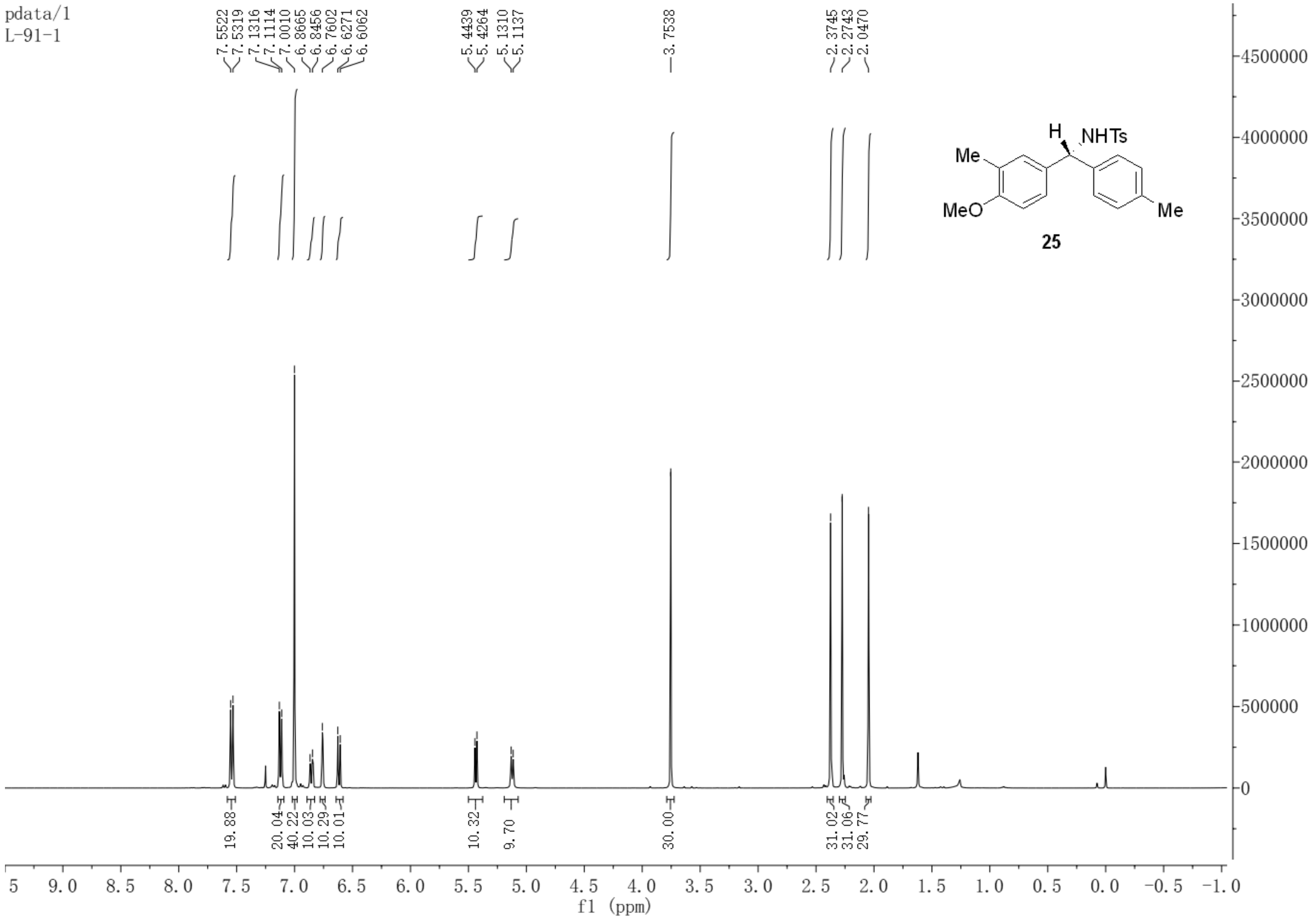

Supplementary Figure 52. ^{1}H NMR spectra of compound **25**

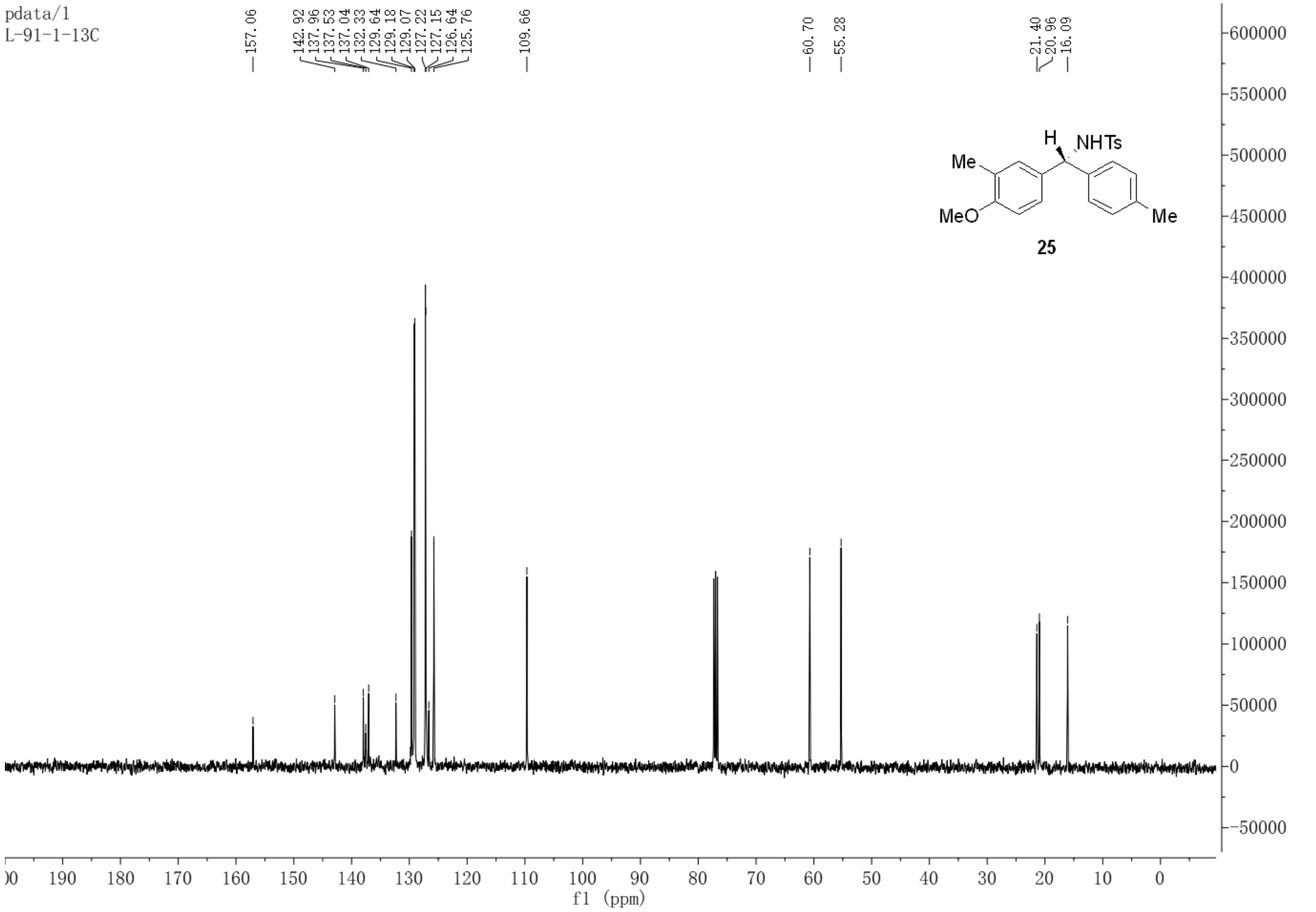

Supplementary Figure 53. ^{13}C NMR spectra of compound **25**

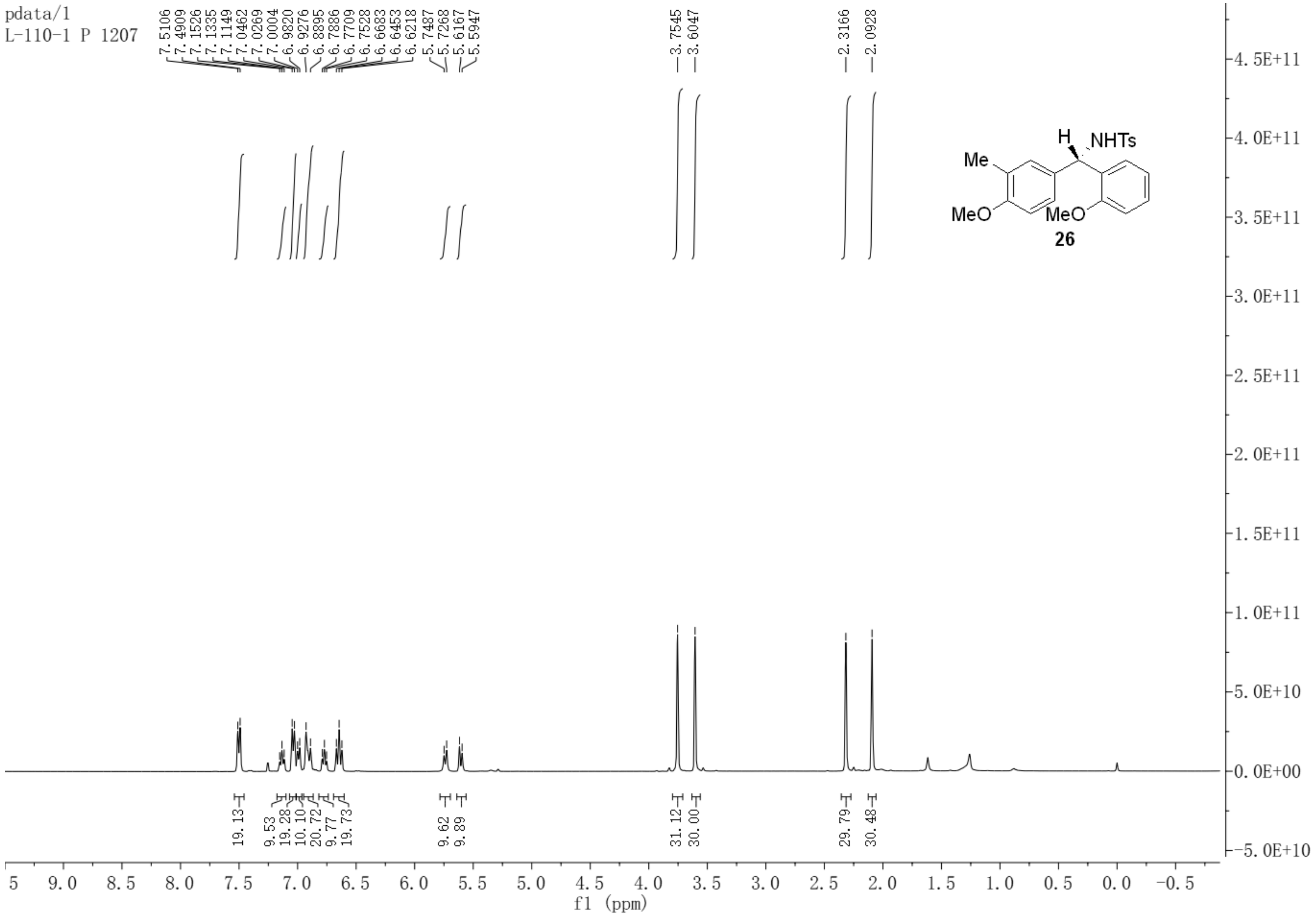

Supplementary Figure 54. ^{1}H NMR spectra of compound **26**

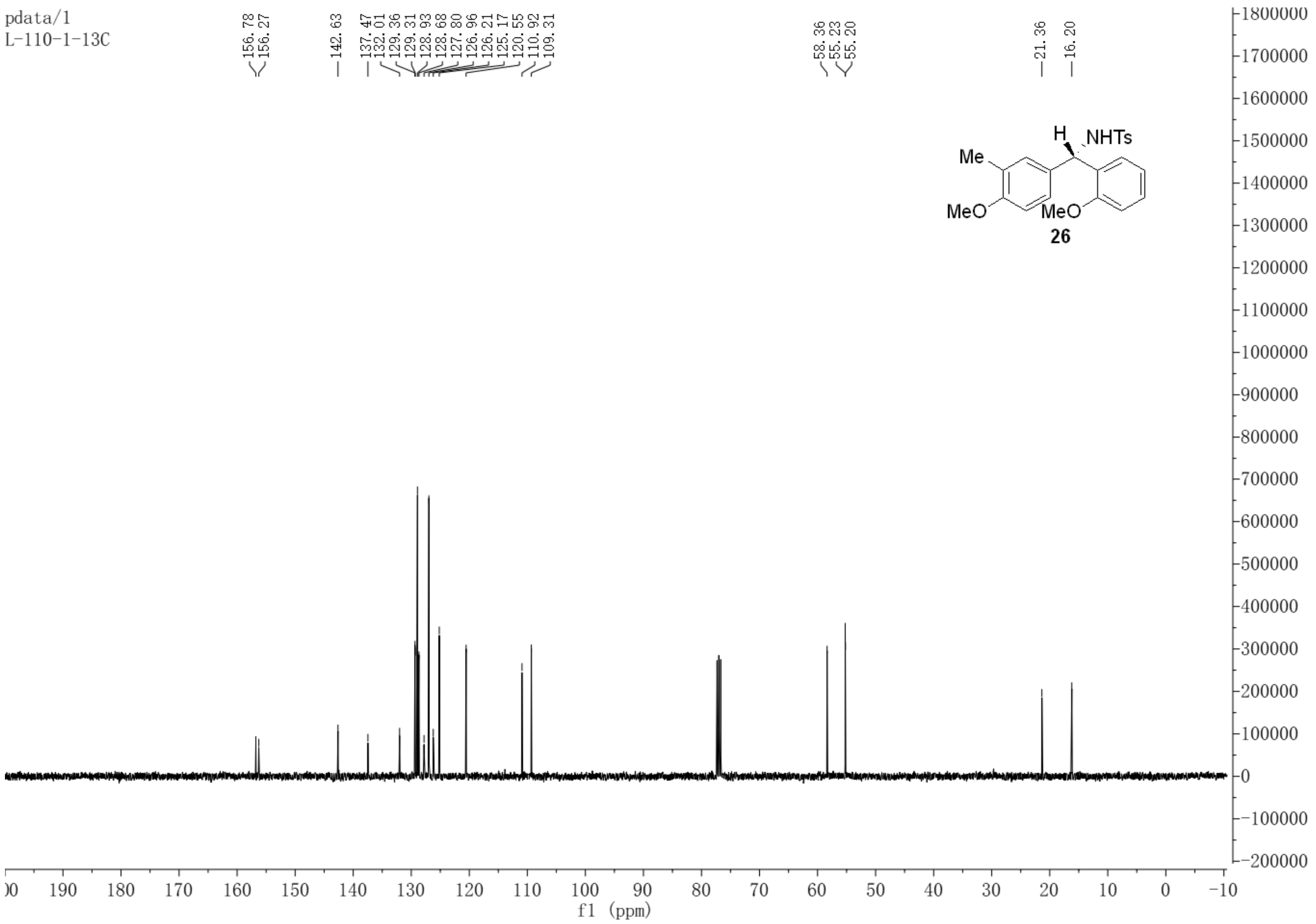

Supplementary Figure 55. ^{13}C NMR spectra of compound **26**

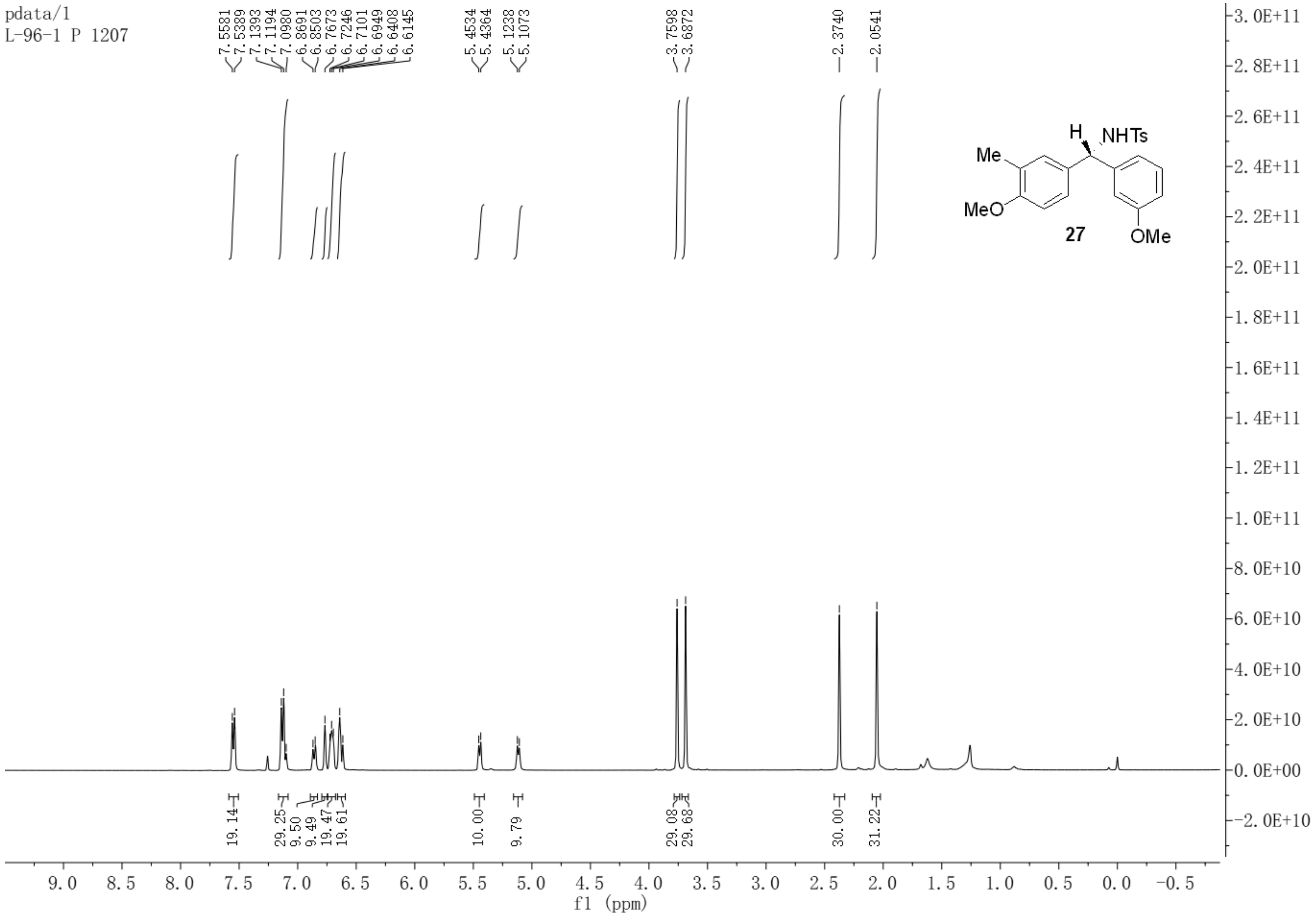

Supplementary Figure 56. ^{1}H NMR spectra of compound **27**

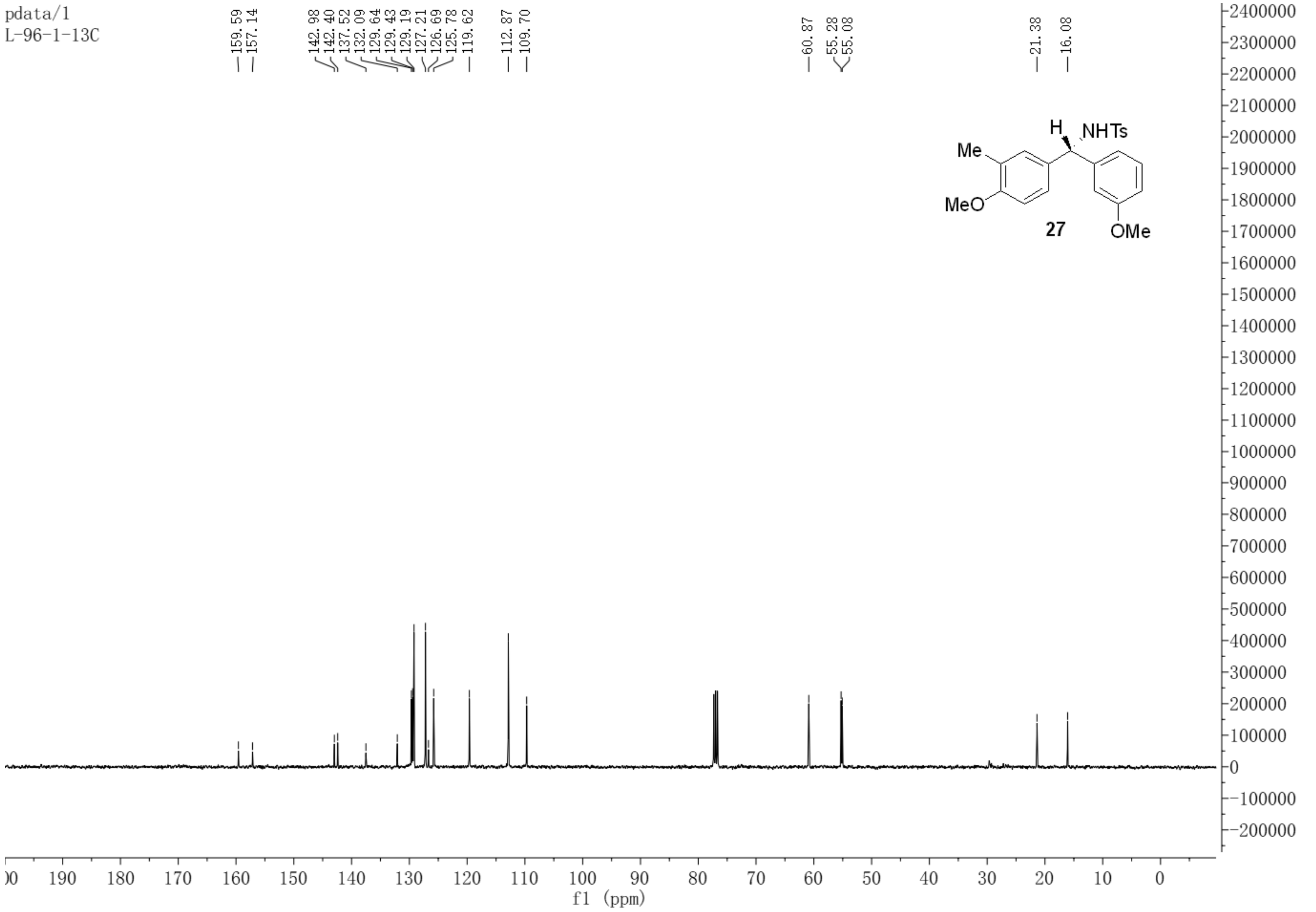

Supplementary Figure 57. ^{13}C NMR spectra of compound **27**

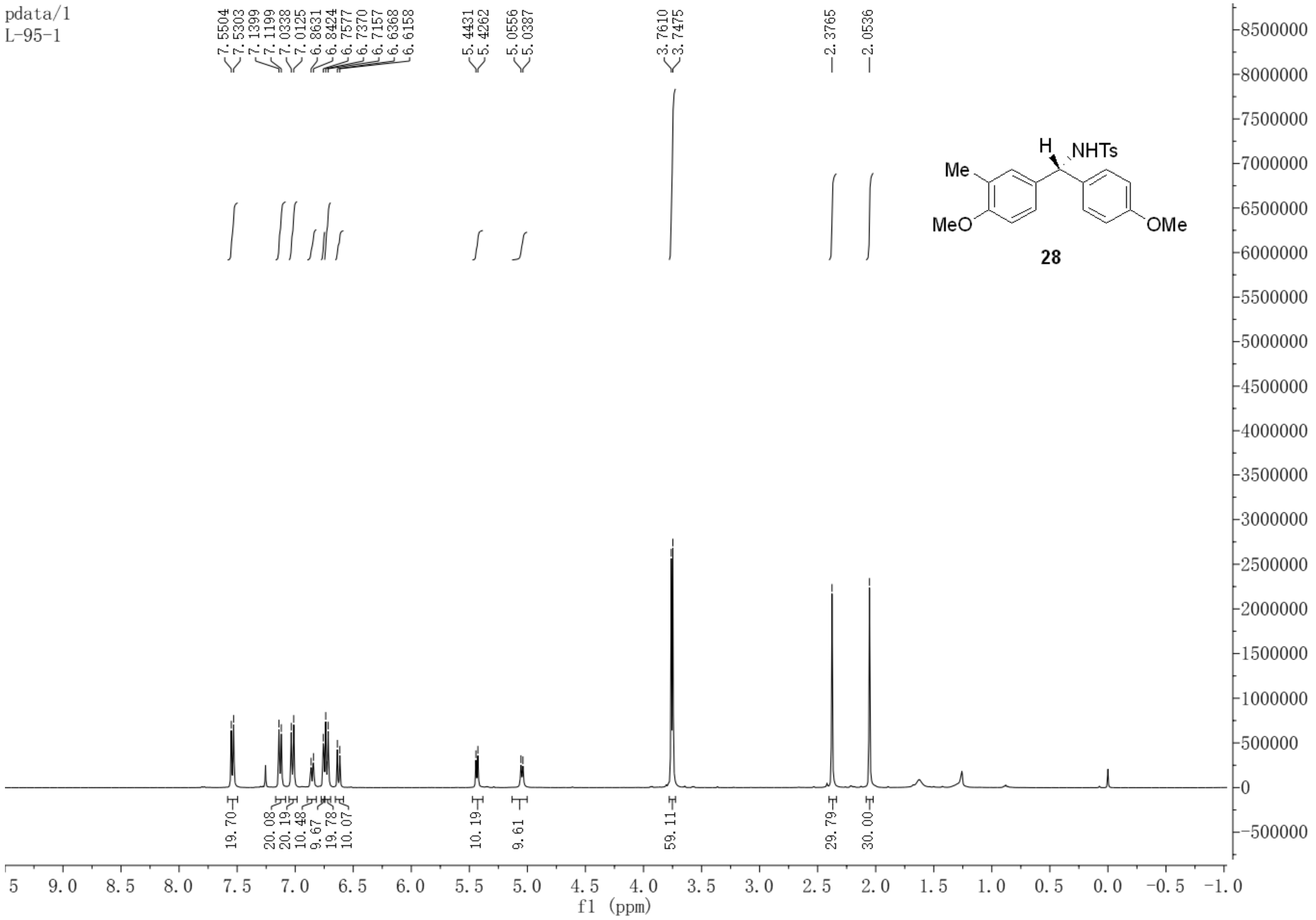

Supplementary Figure 58. ^{1}H NMR spectra of compound **28**

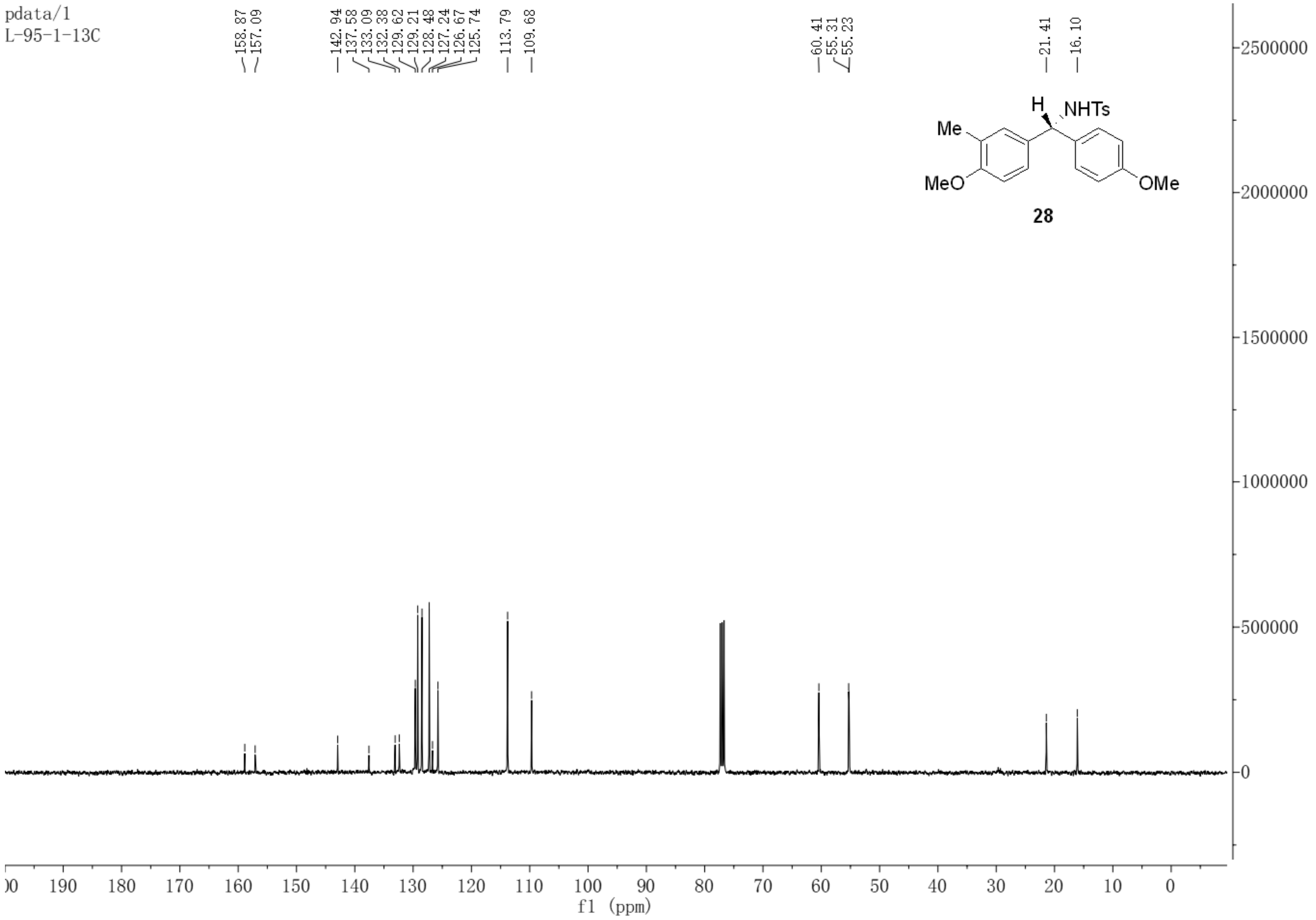

Supplementary Figure 59. ^{13}C NMR spectra of compound **28**

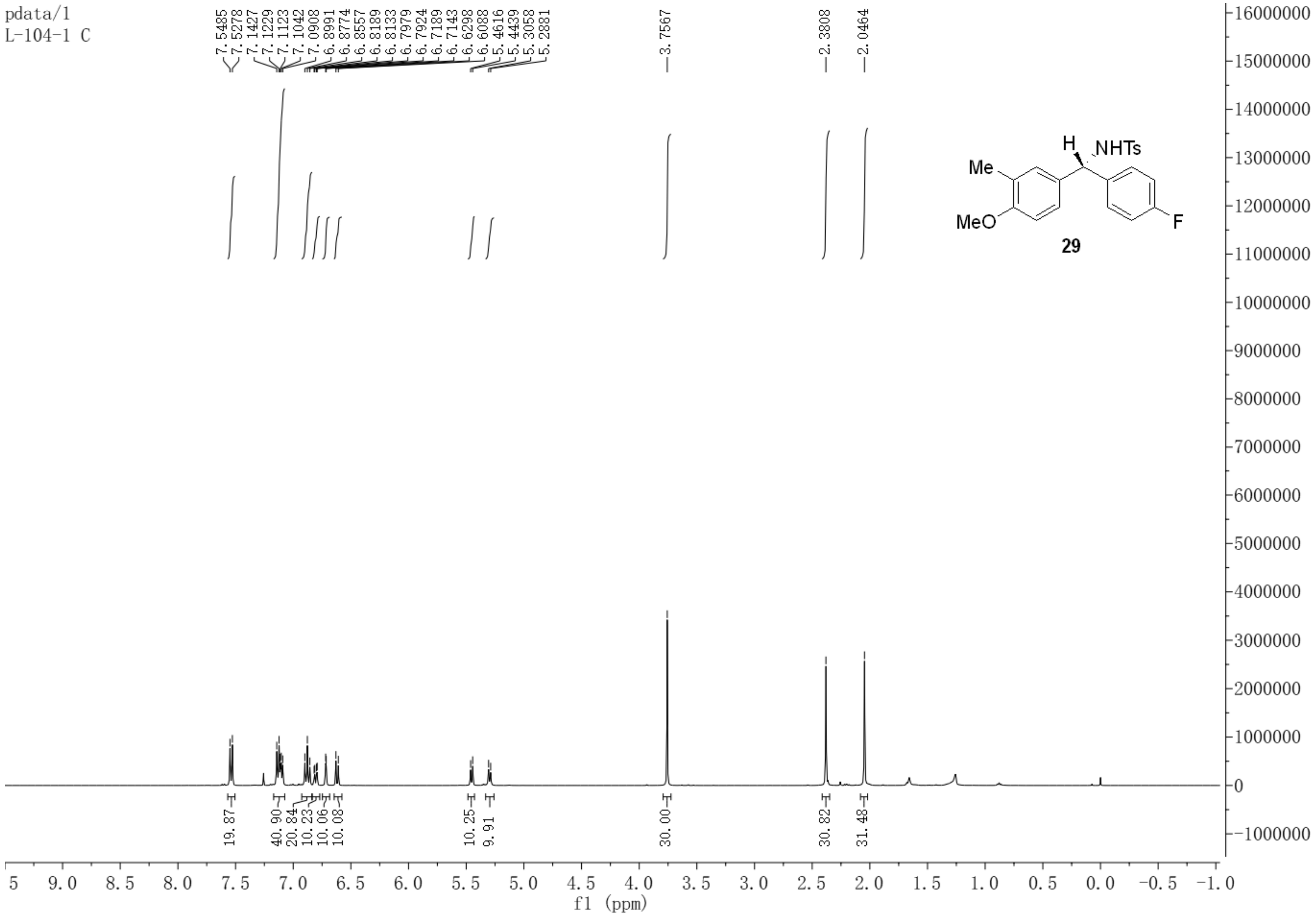

Supplementary Figure 60. ^{1}H NMR spectra of compound **29**

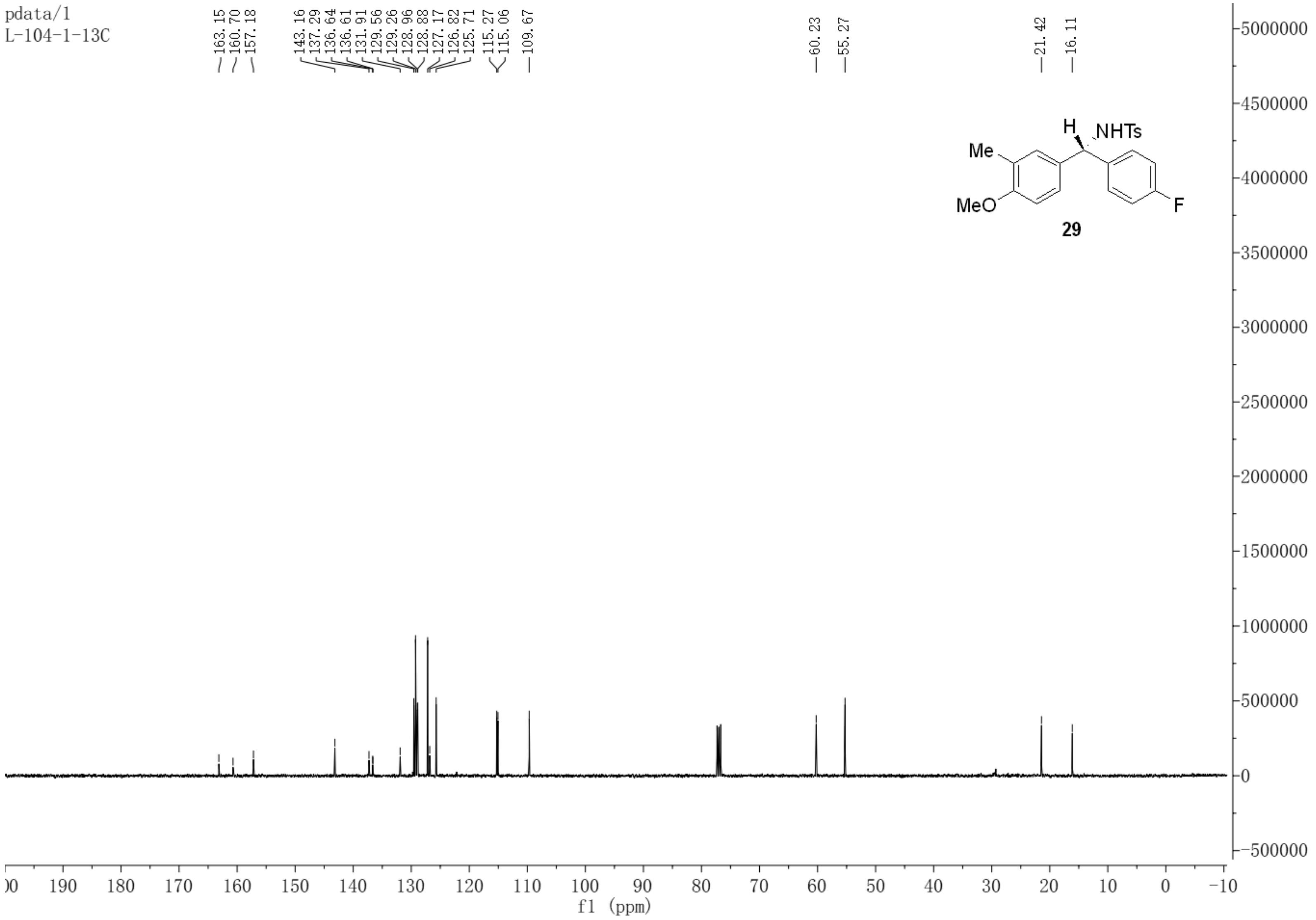

Supplementary Figure 61. ^{13}C NMR spectra of compound **29**

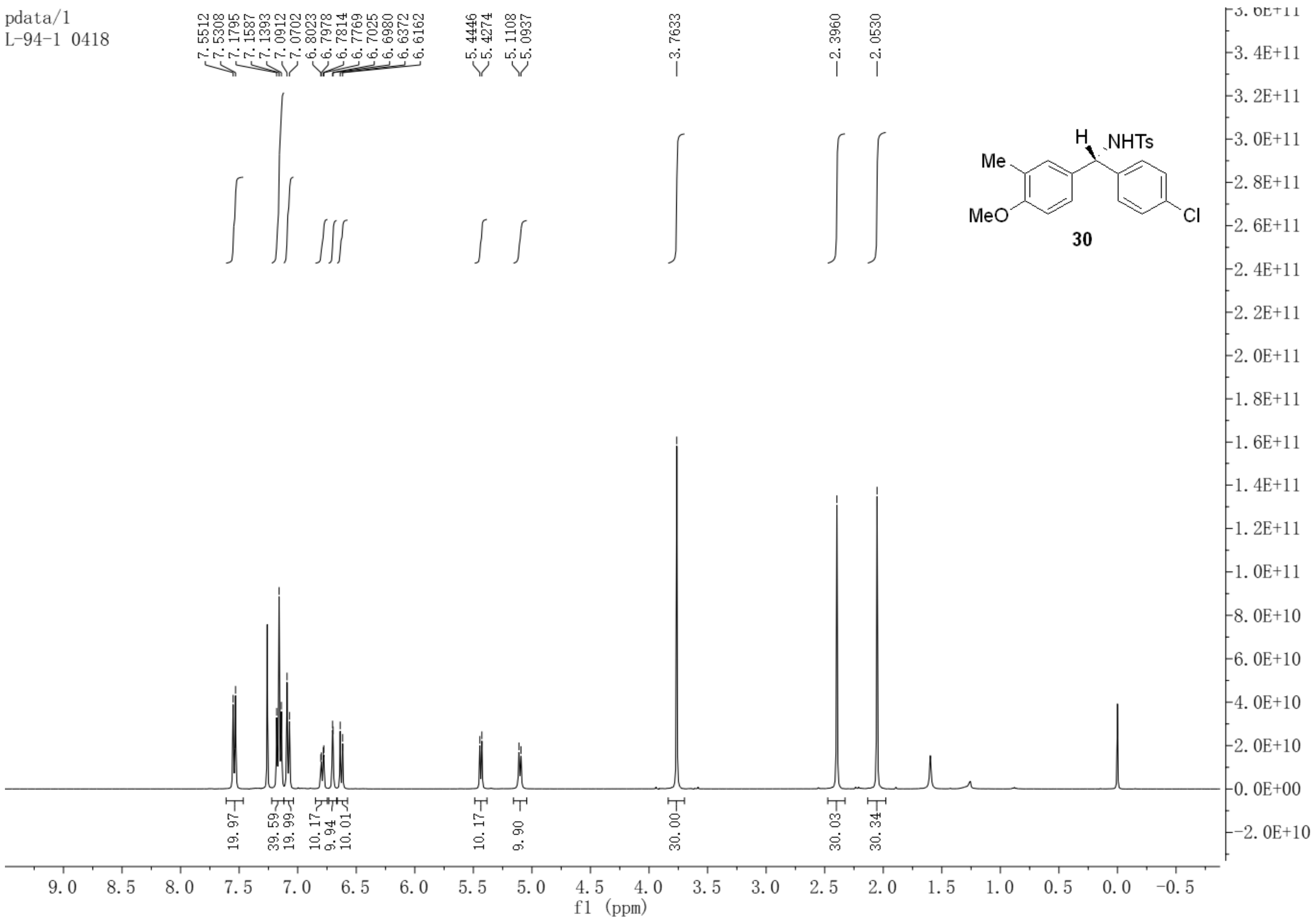

Supplementary Figure 62. ^{1}H NMR spectra of compound **30**

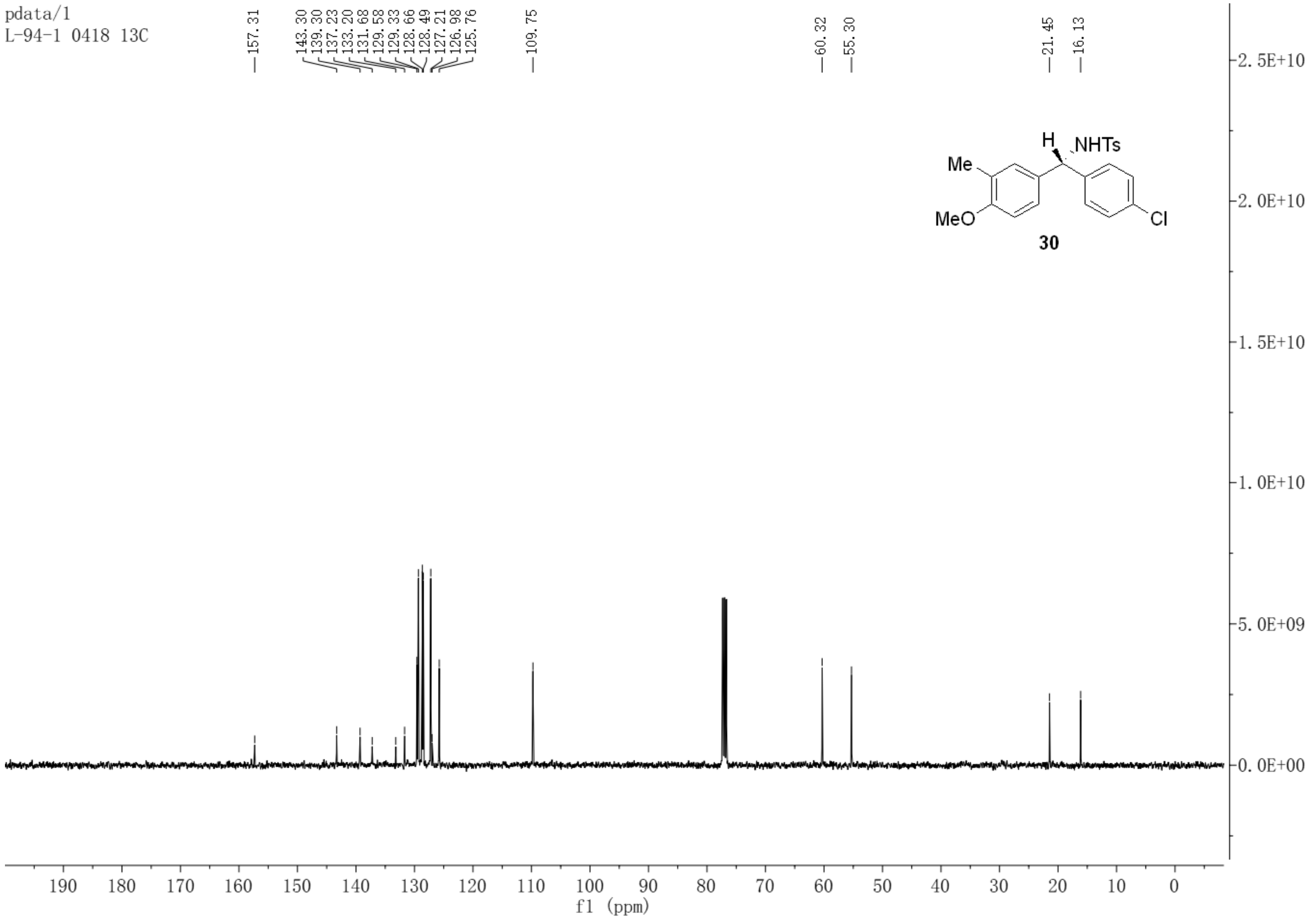

Supplementary Figure 63. ^{13}C NMR spectra of compound **30**

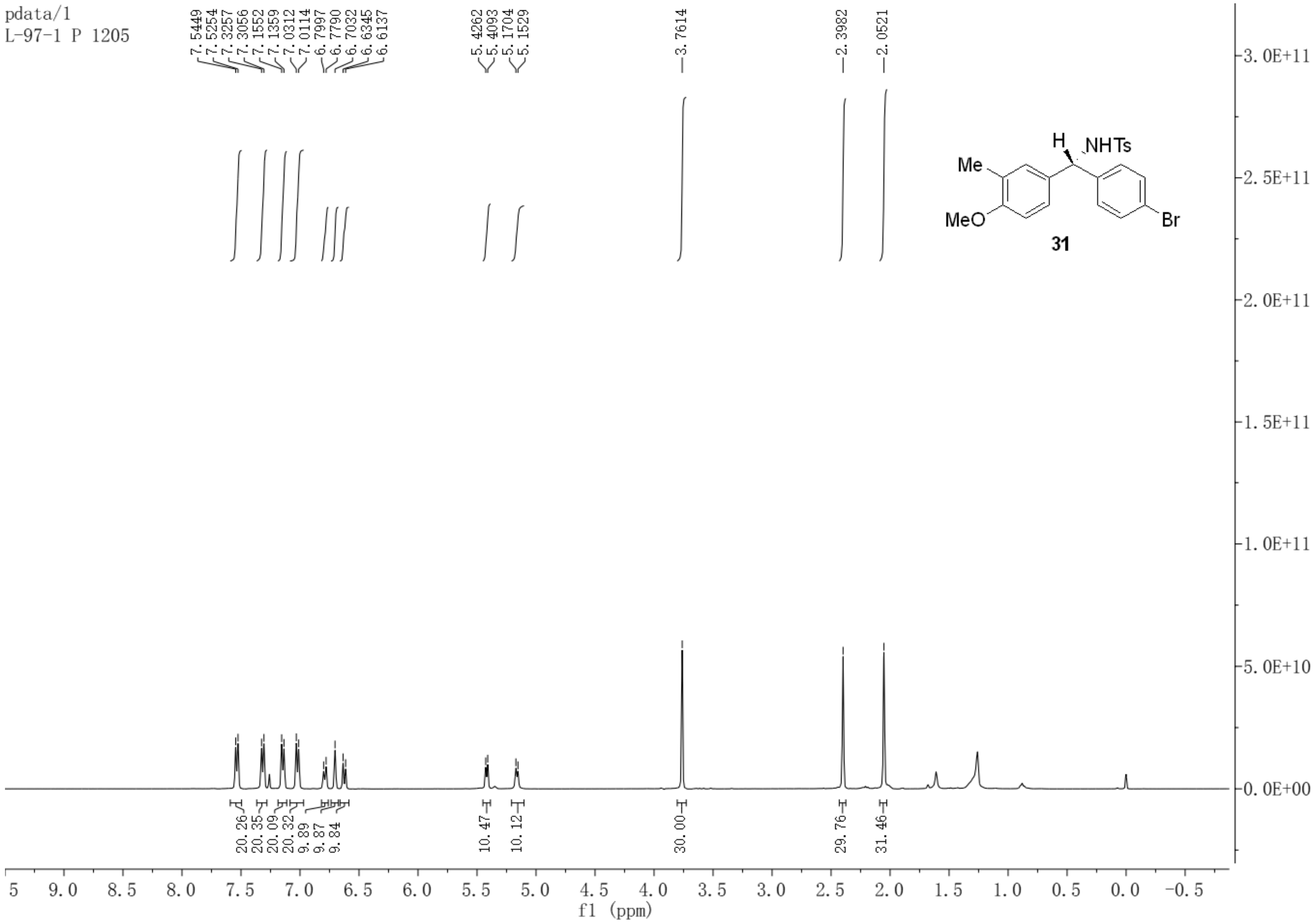

Supplementary Figure 64. ^{1}H NMR spectra of compound **31**

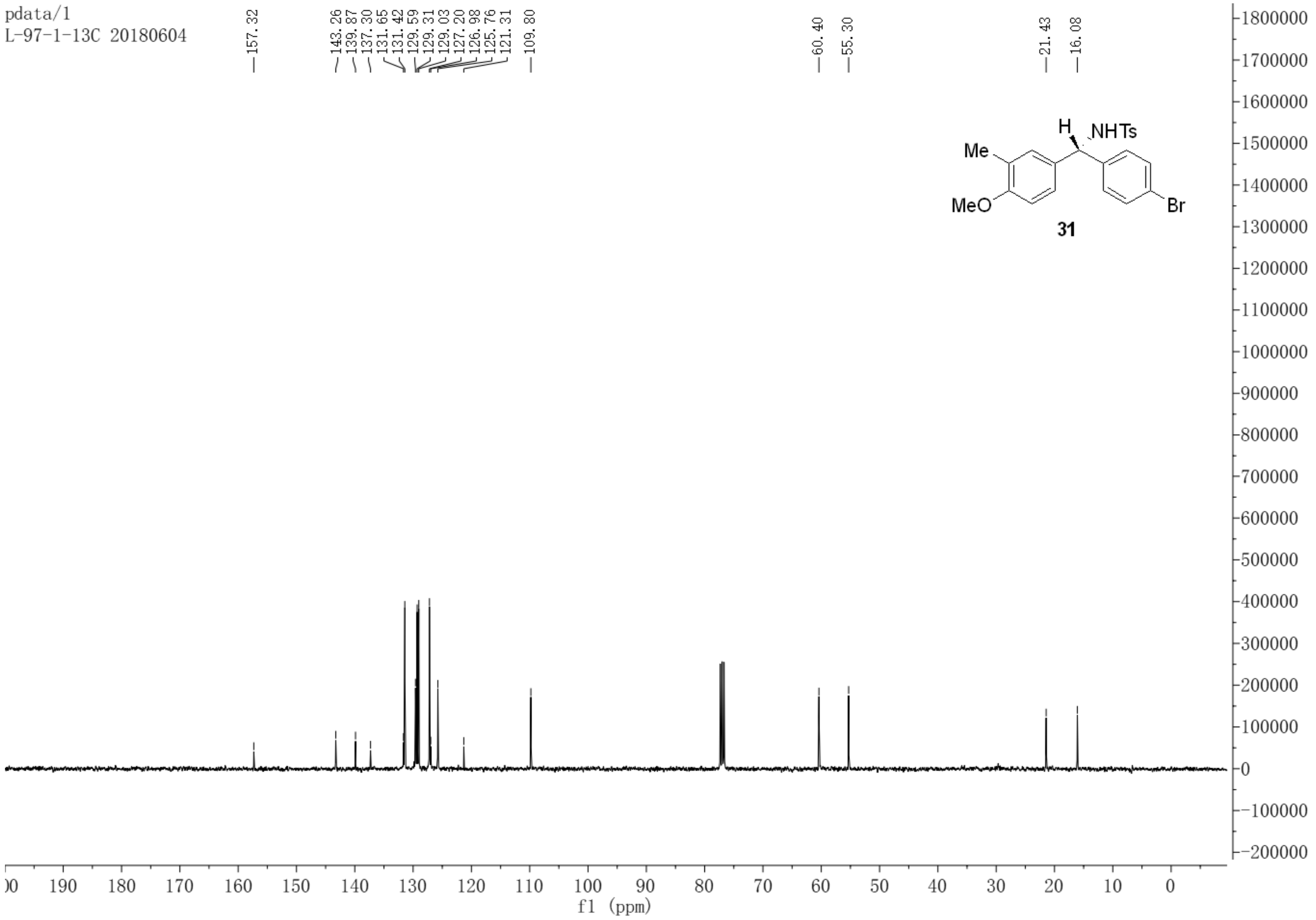

Supplementary Figure 65. ^{13}C NMR spectra of compound **31**

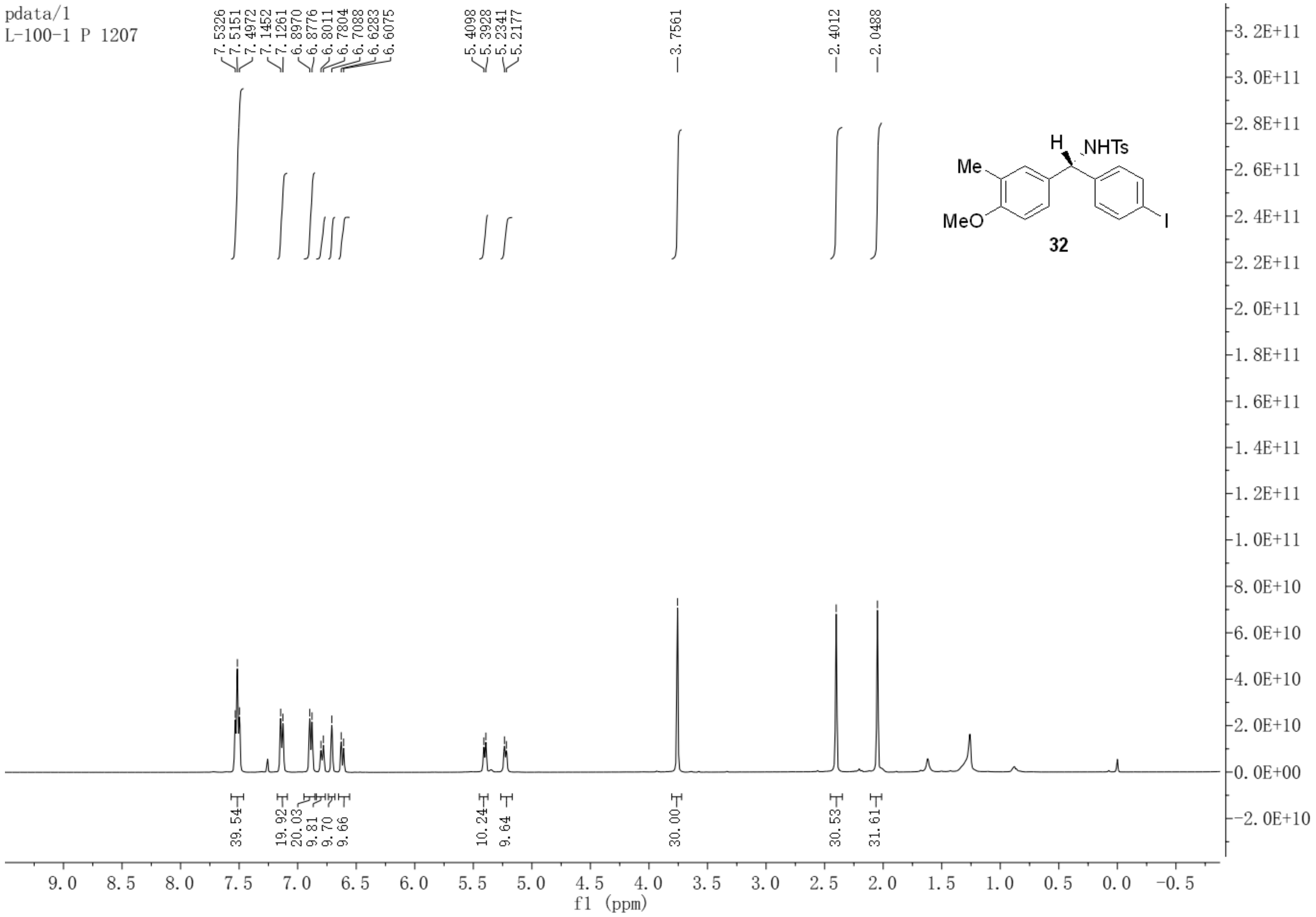

Supplementary Figure 66. ^{1}H NMR spectra of compound **32**

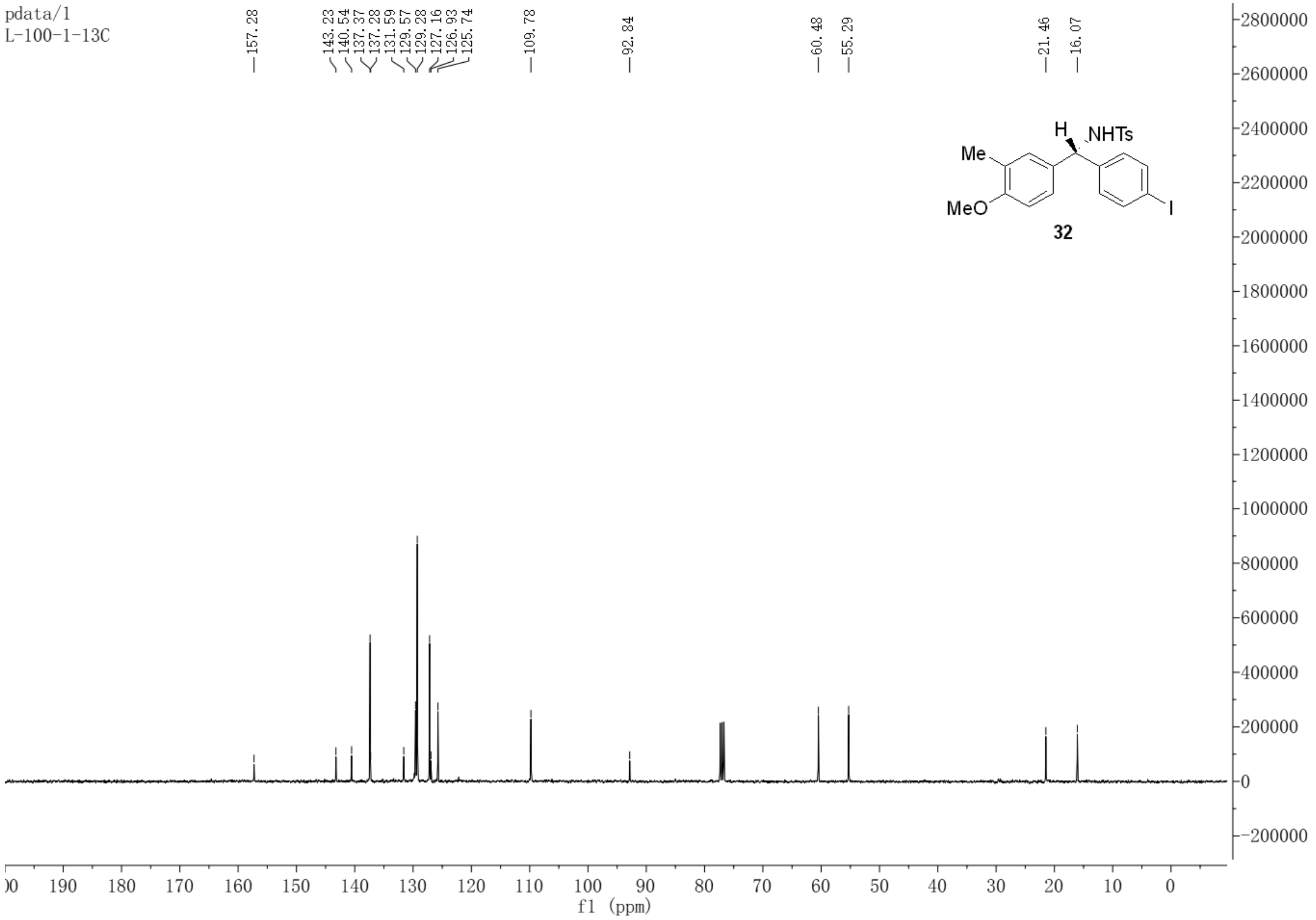

Supplementary Figure 67. ^{13}C NMR spectra of compound **32**

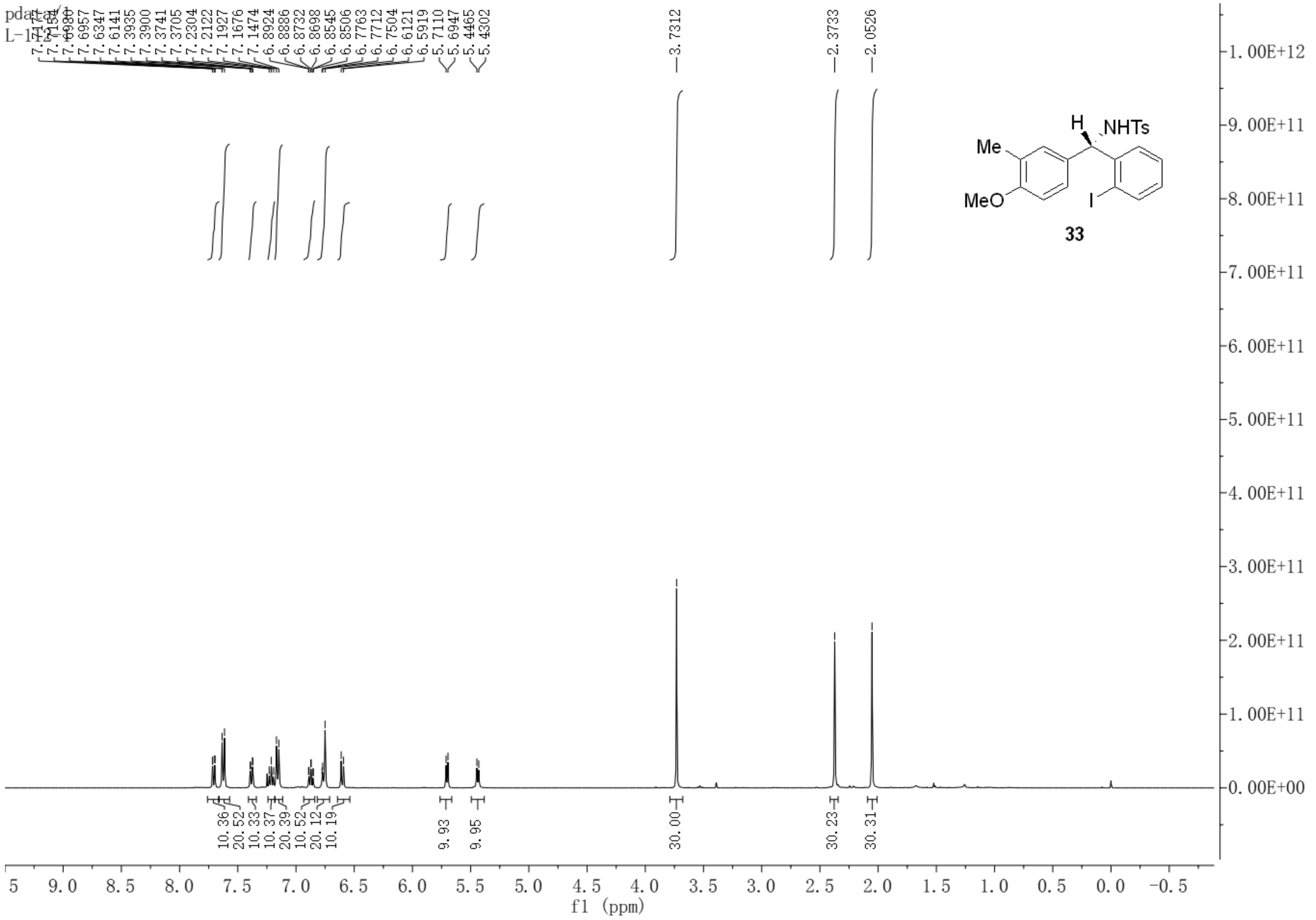

Supplementary Figure 68. ^{1}H NMR spectra of compound **33**

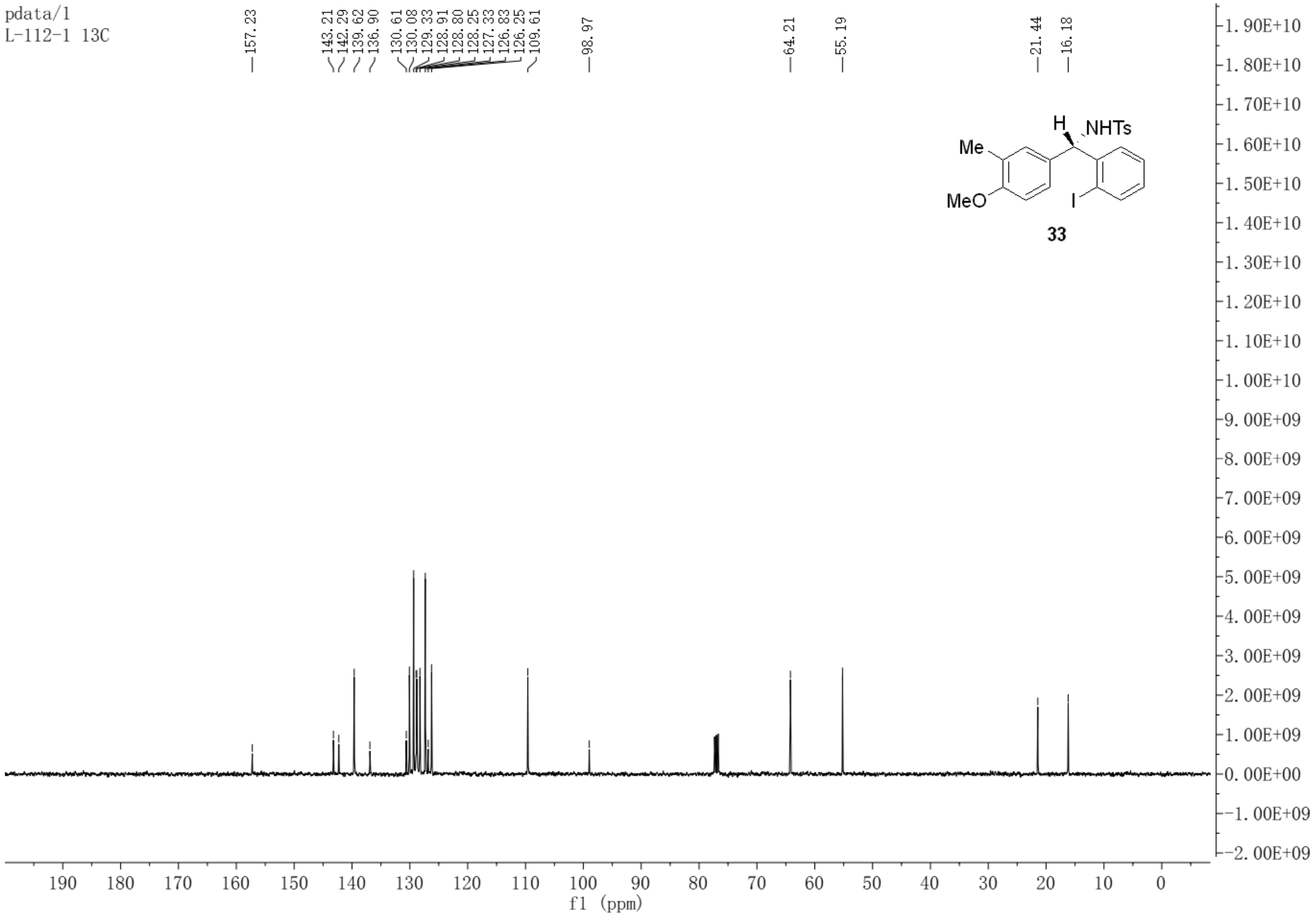

Supplementary Figure 69. ^{13}C NMR spectra of compound **33**

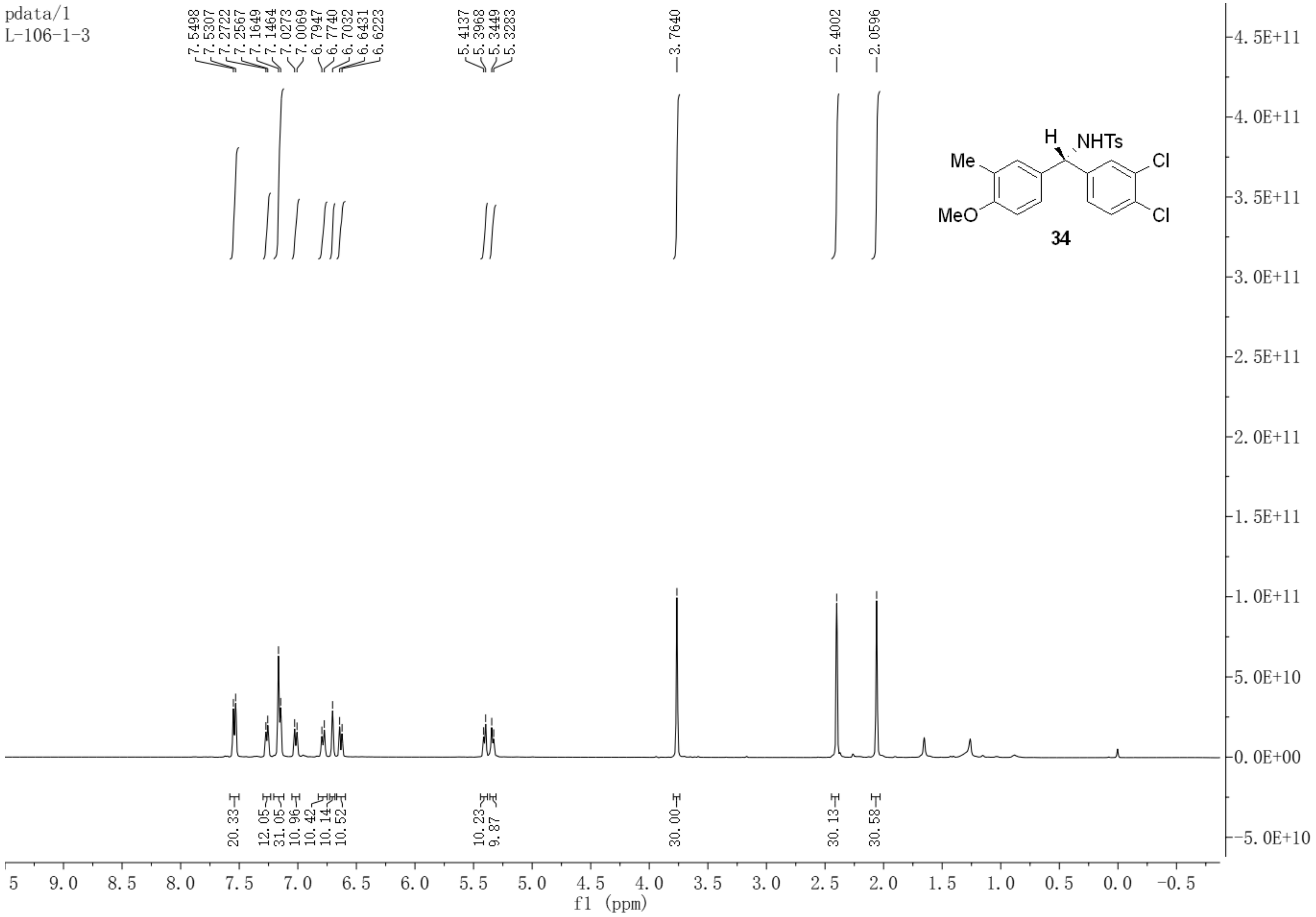

Supplementary Figure 70. ^{1}H NMR spectra of compound **34**

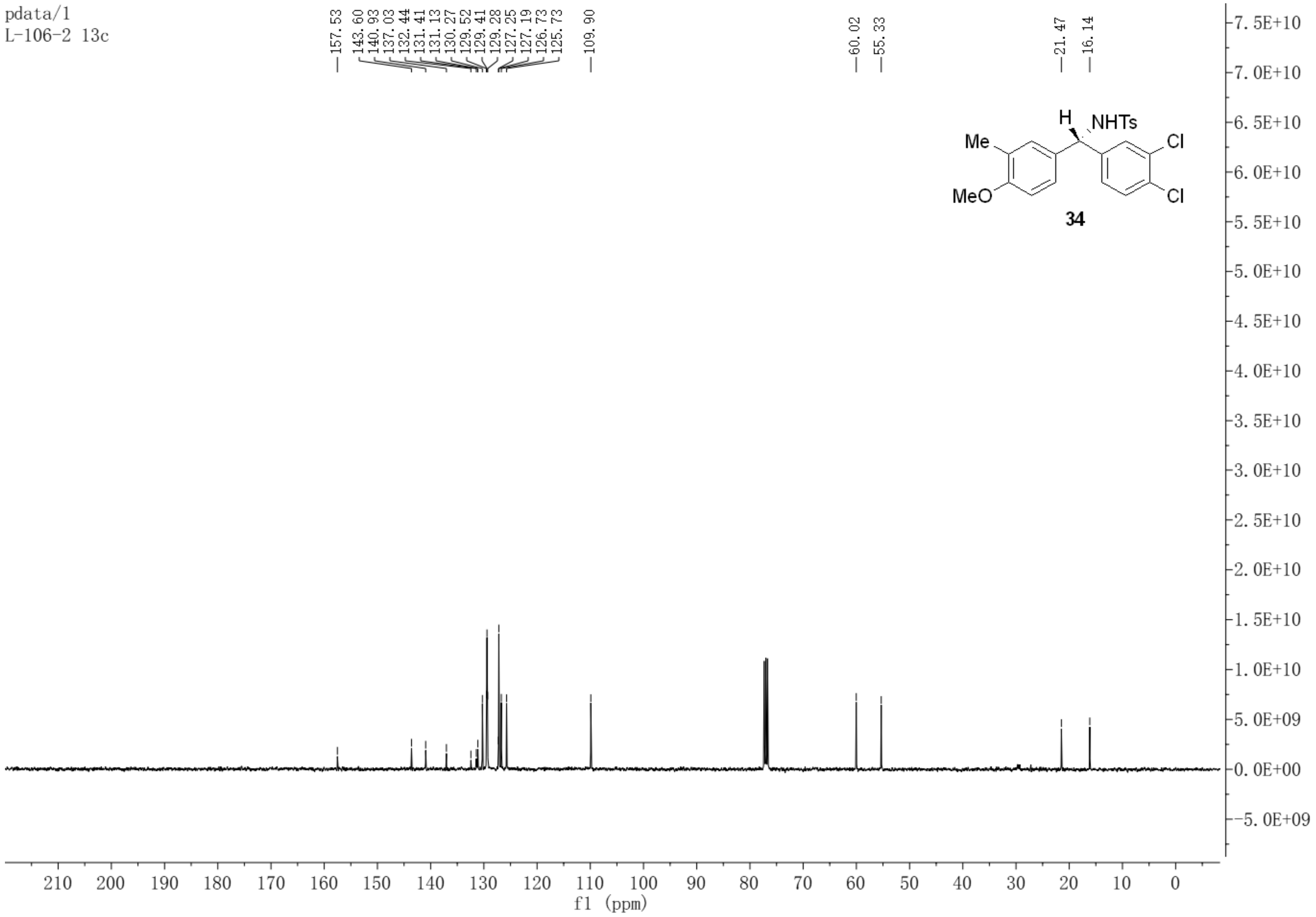

Supplementary Figure 71. ^{13}C NMR spectra of compound **34**

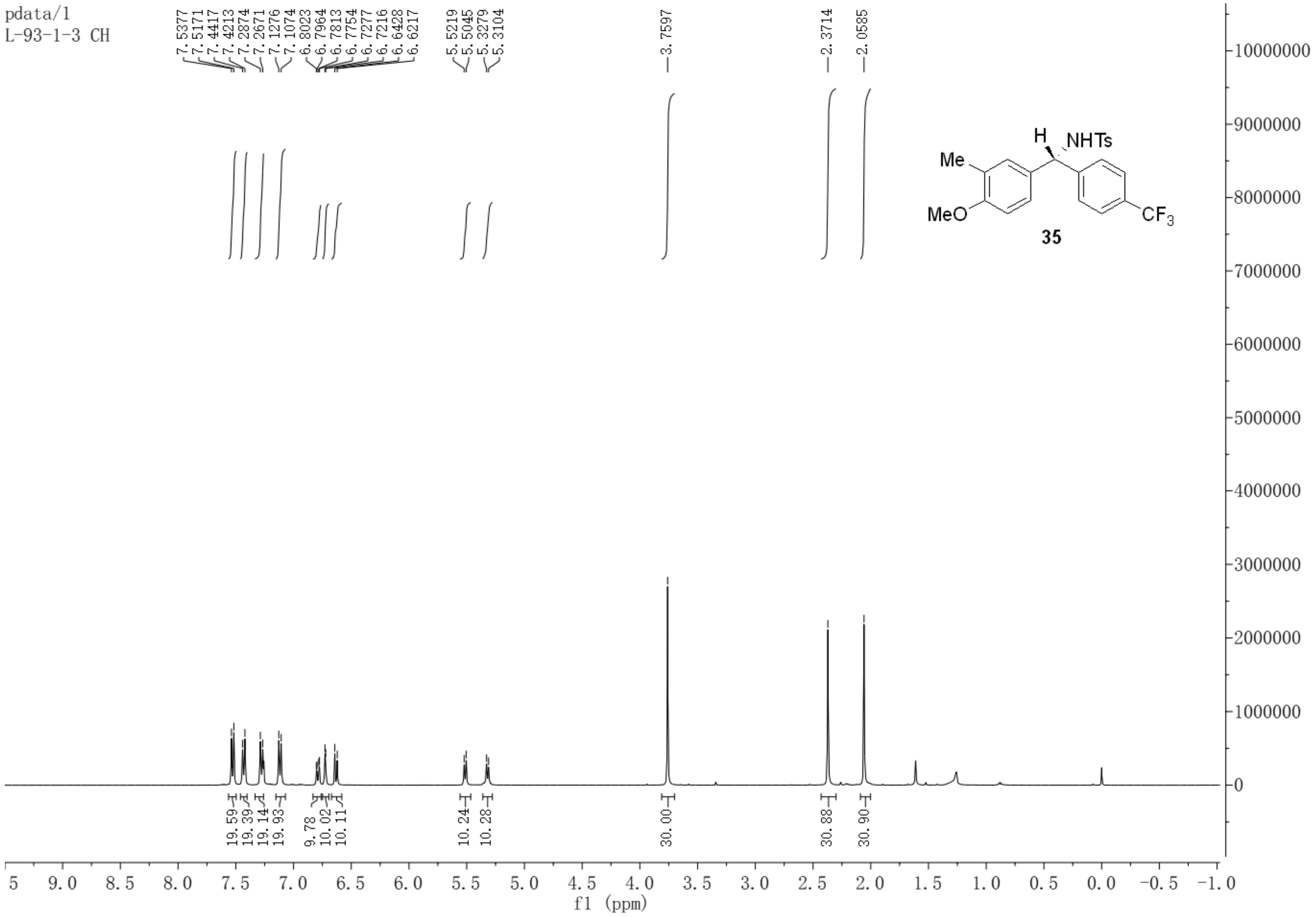

Supplementary Figure 72. ^{1}H NMR spectra of compound **35**

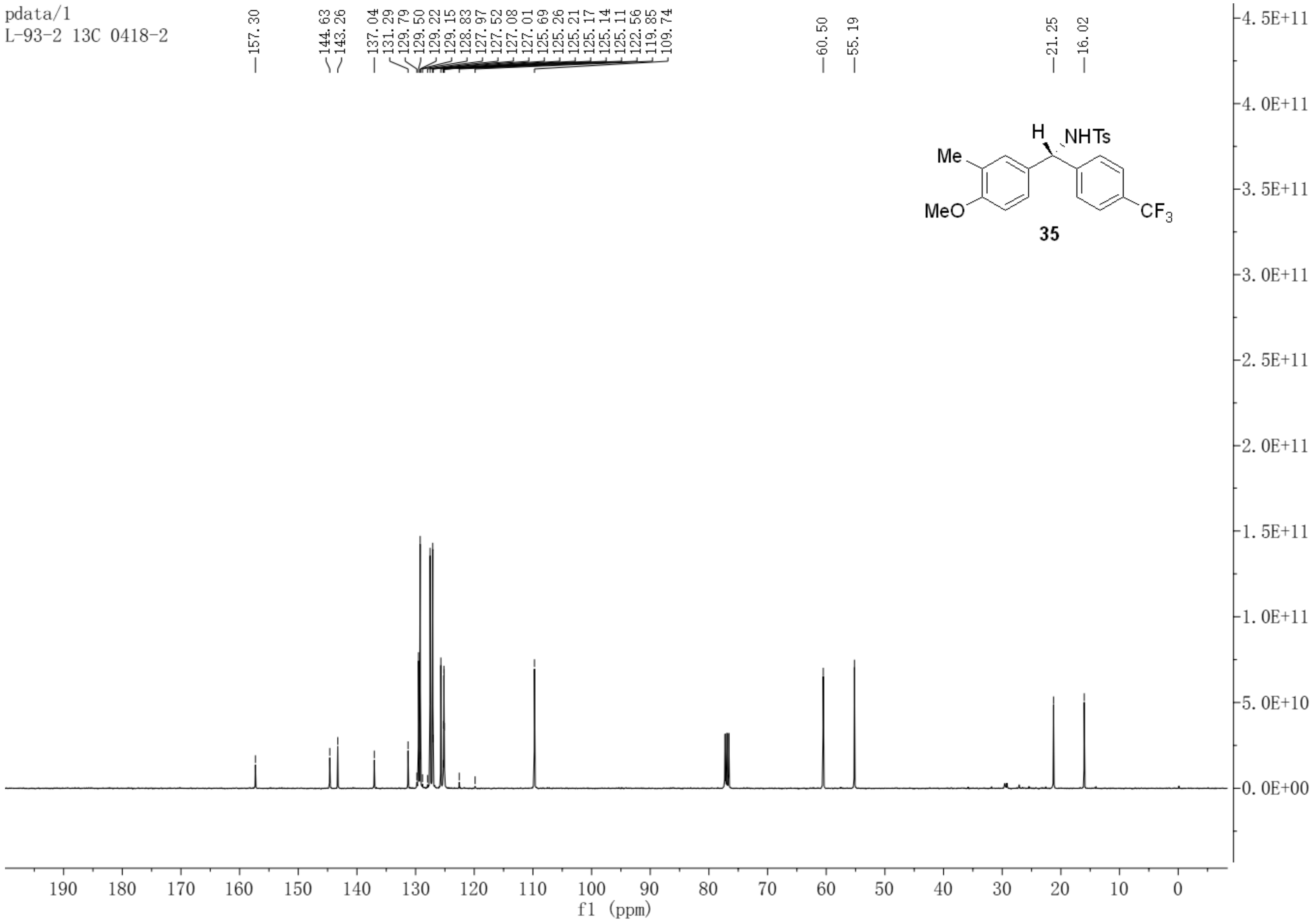

Supplementary Figure 73. ^{13}C NMR spectra of compound **35**

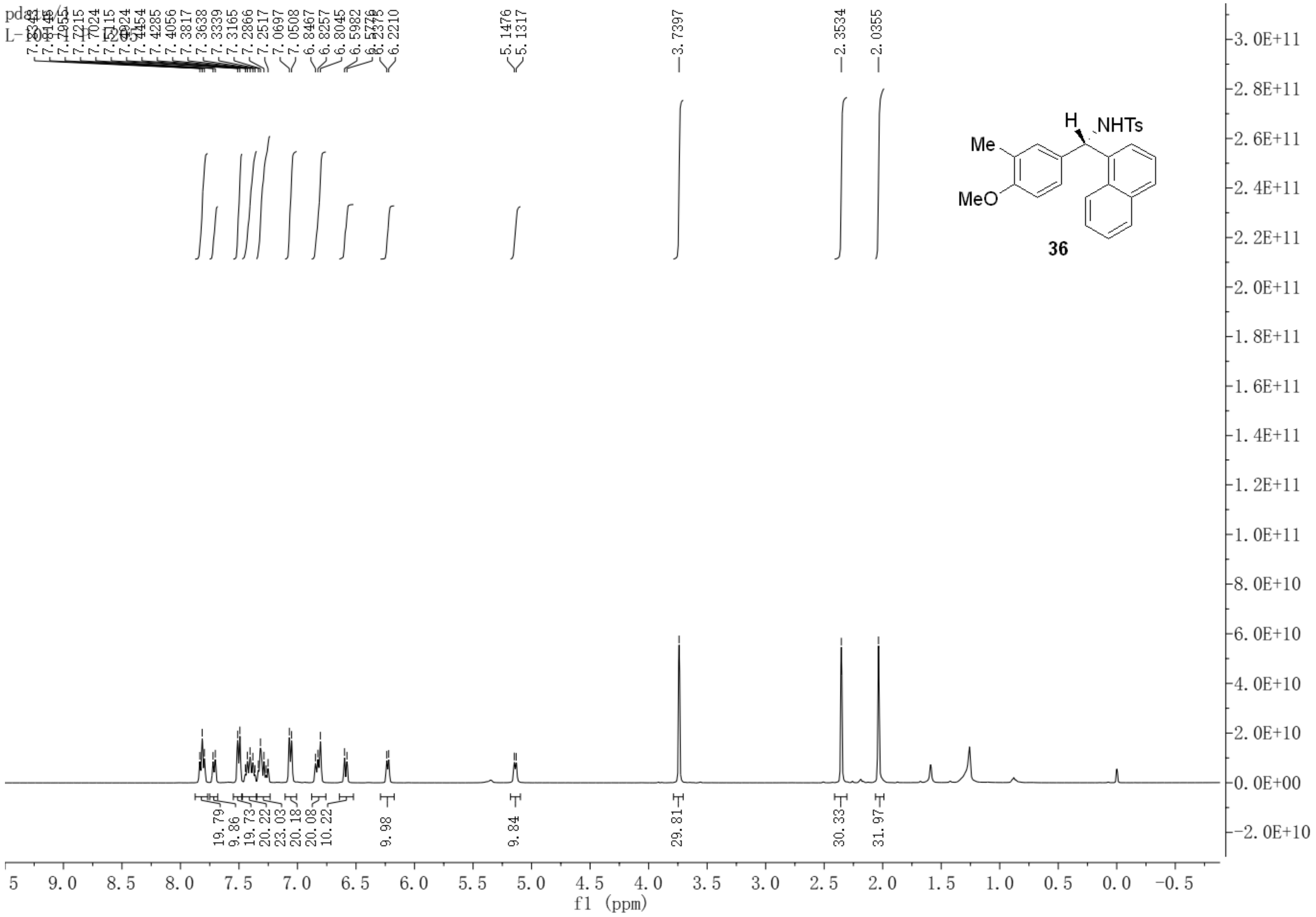

Supplementary Figure 74. ^{1}H NMR spectra of compound **36**

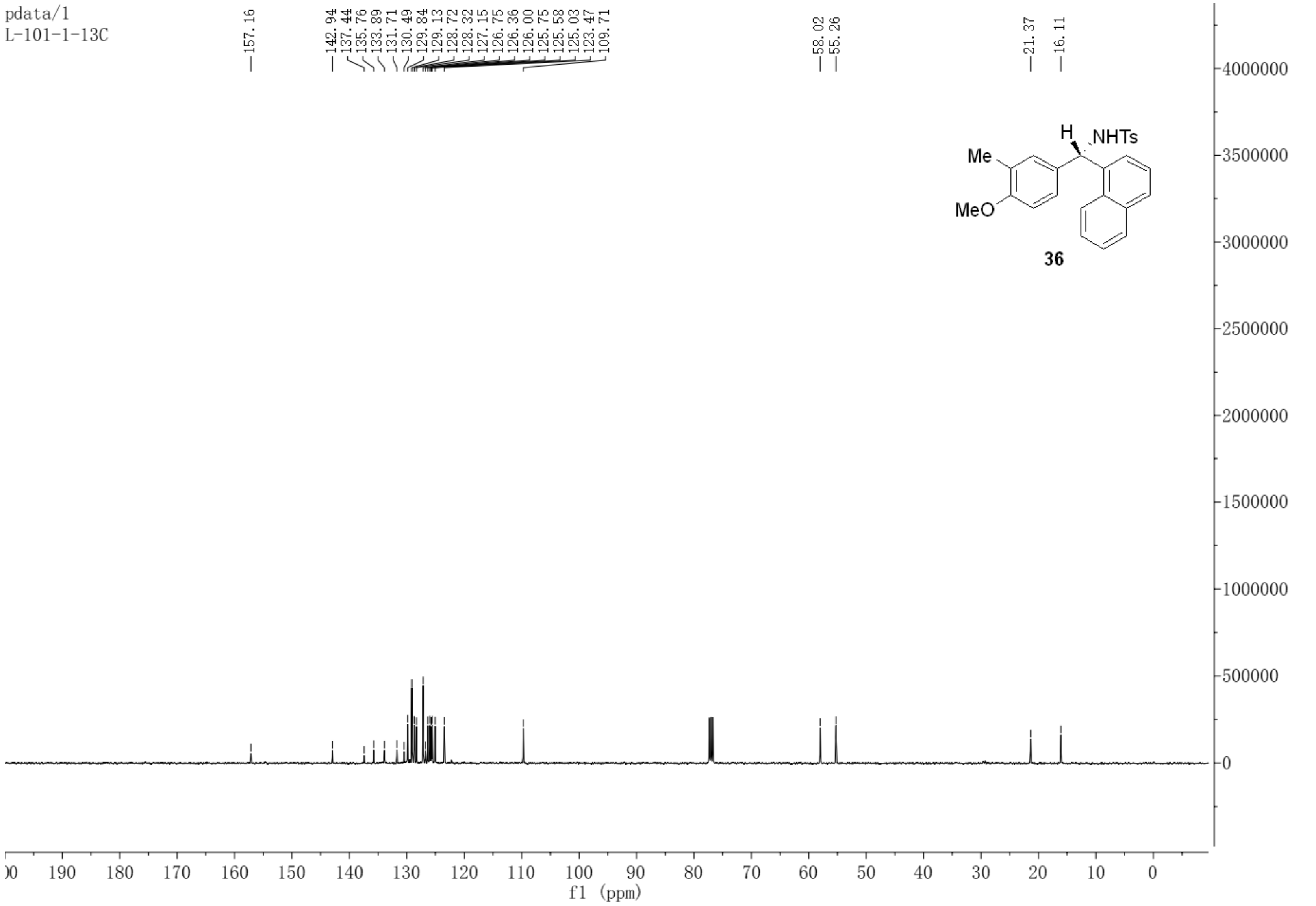

Supplementary Figure 75. ^{13}C NMR spectra of compound **36**

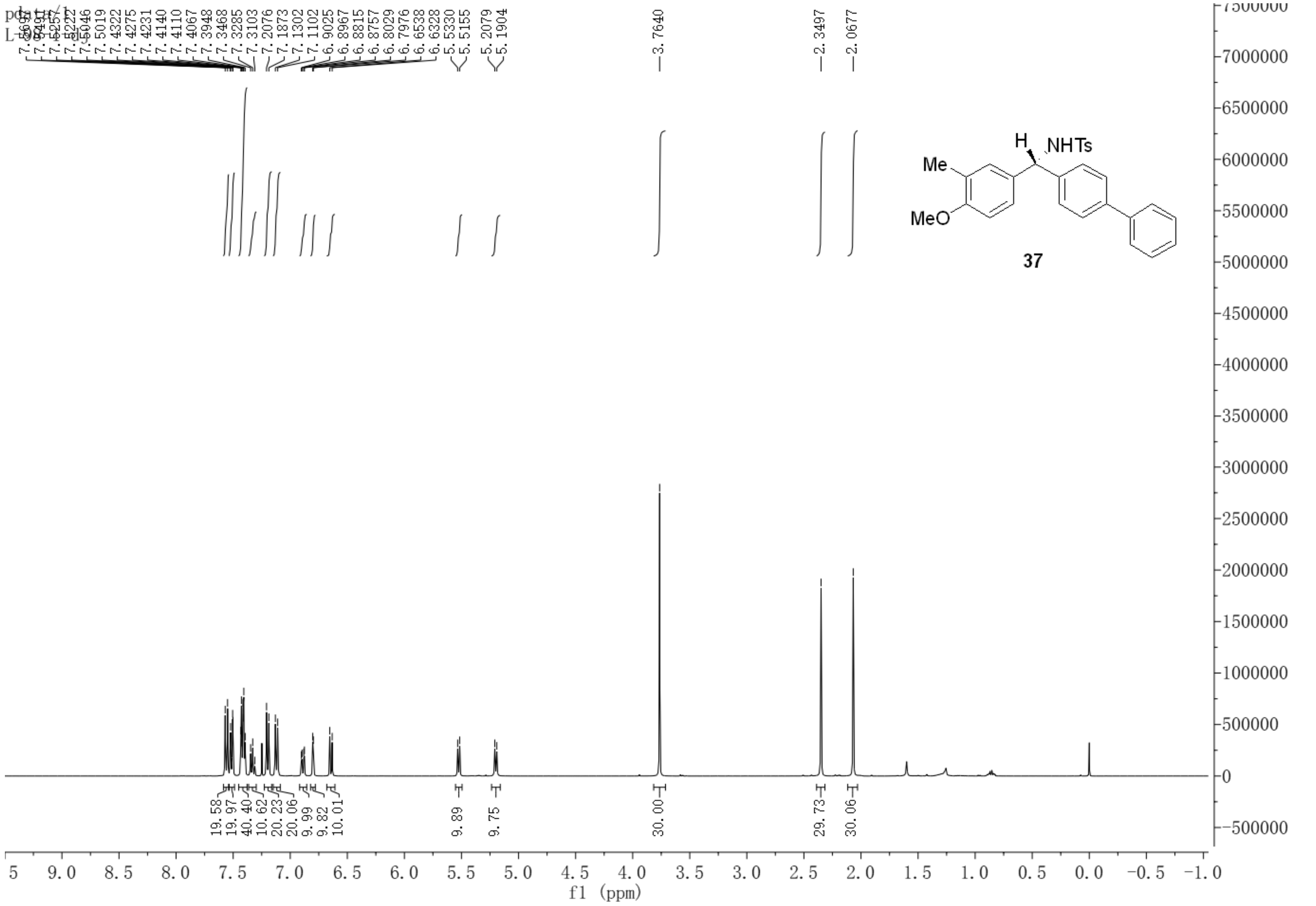

Supplementary Figure 76. ^{1}H NMR spectra of compound **37**

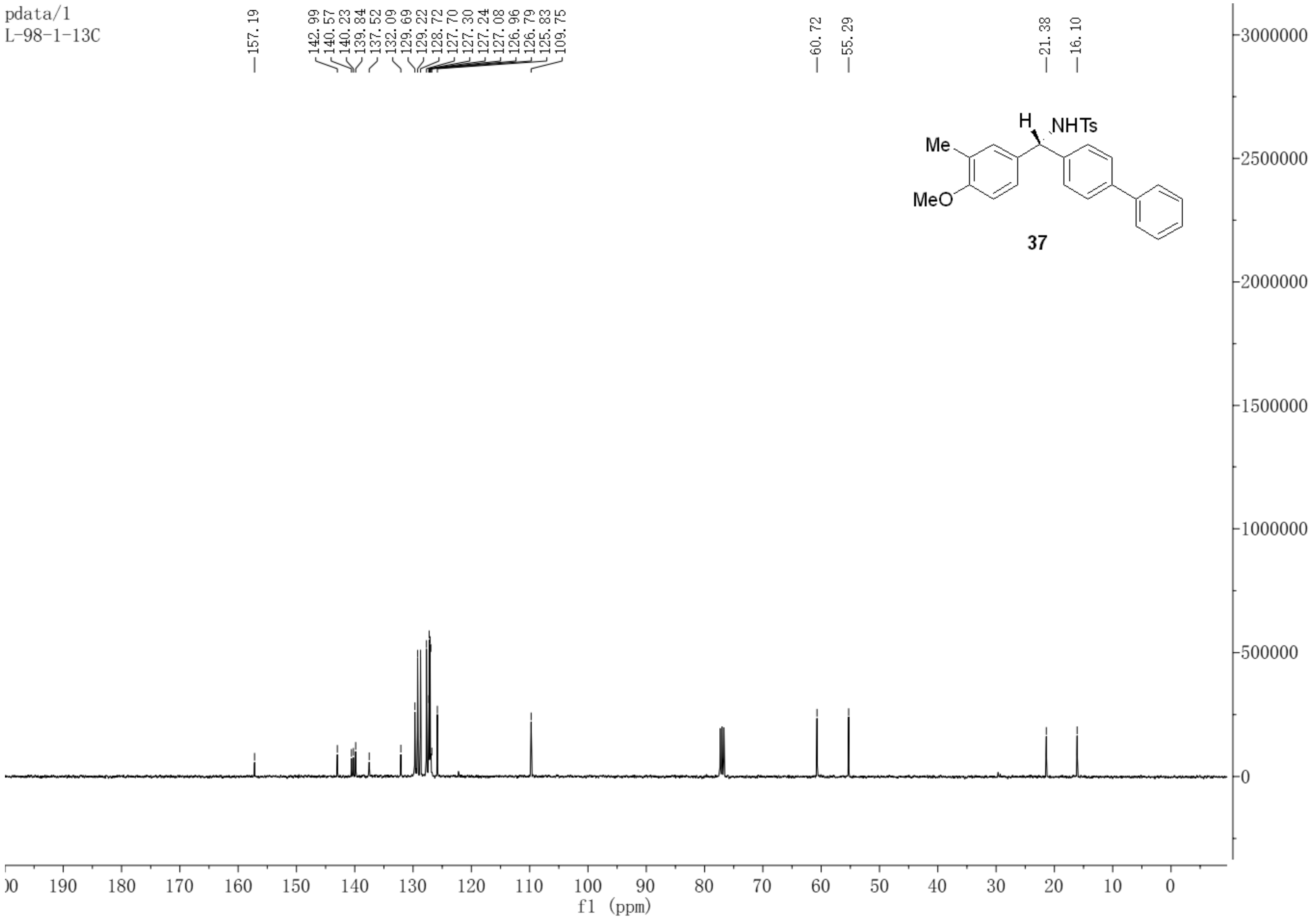

Supplementary Figure 77. ^{13}C NMR spectra of compound **37**

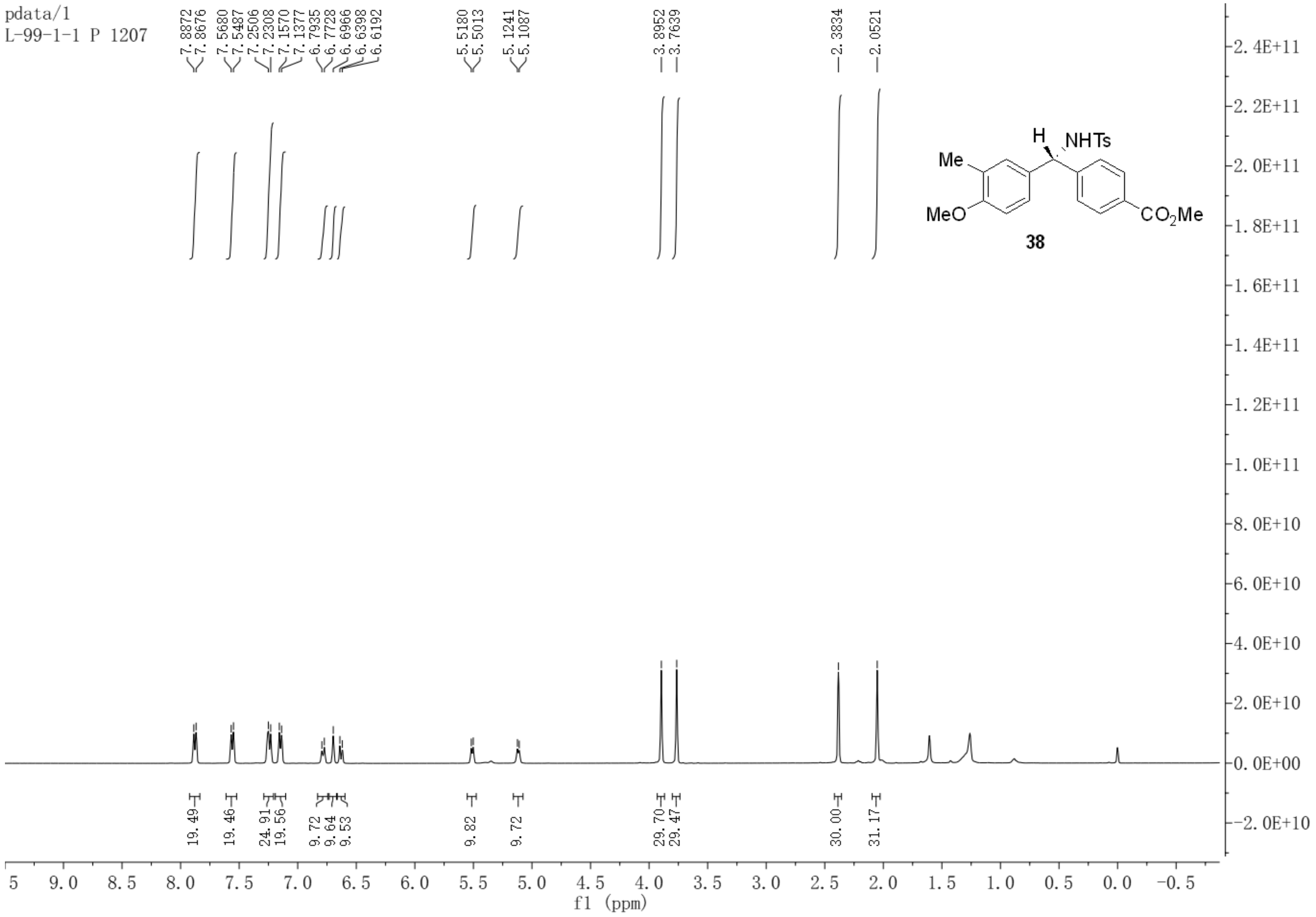

Supplementary Figure 78. ^{1}H NMR spectra of compound **38**

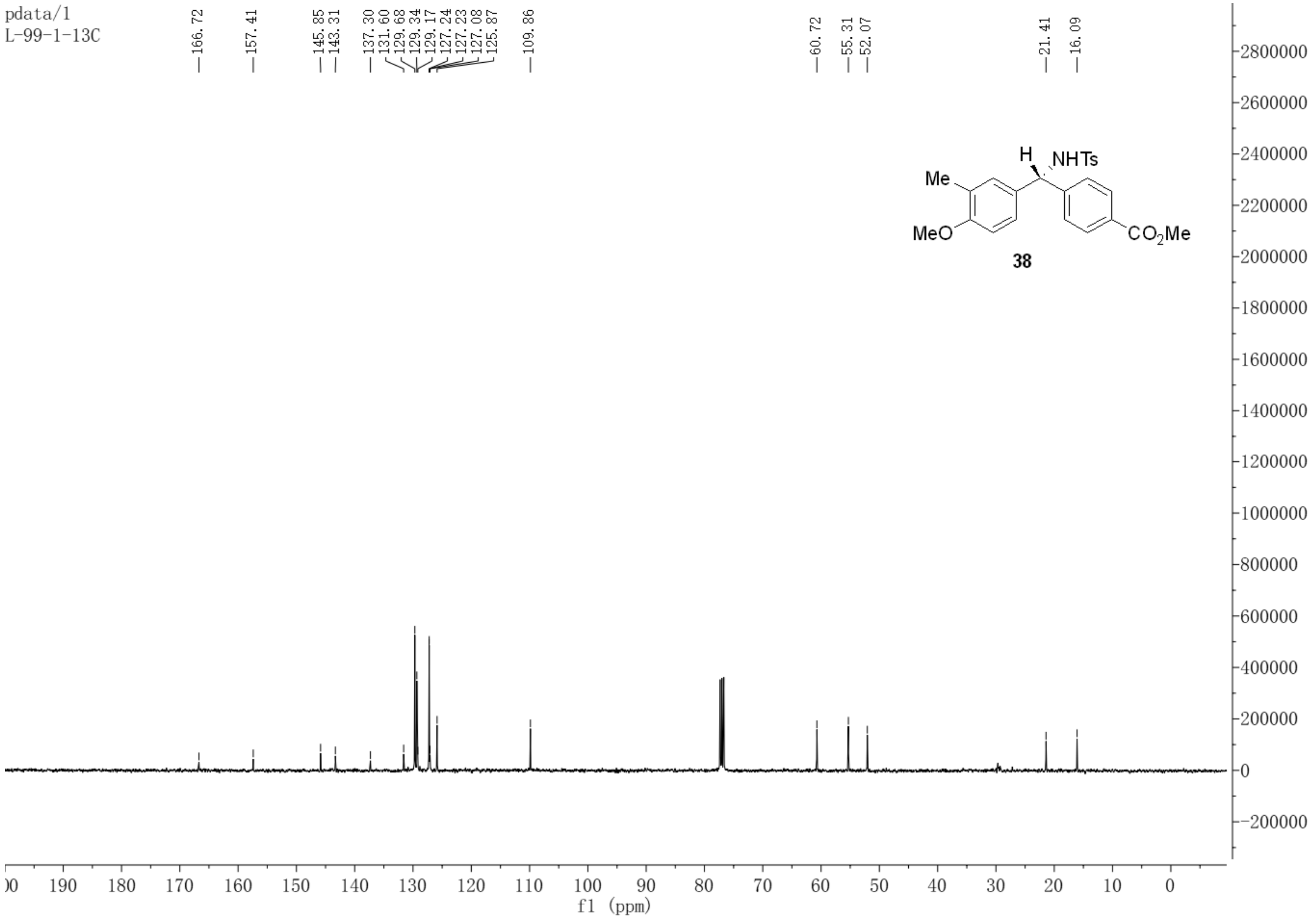

Supplementary Figure 79. ^{13}C NMR spectra of compound **38**

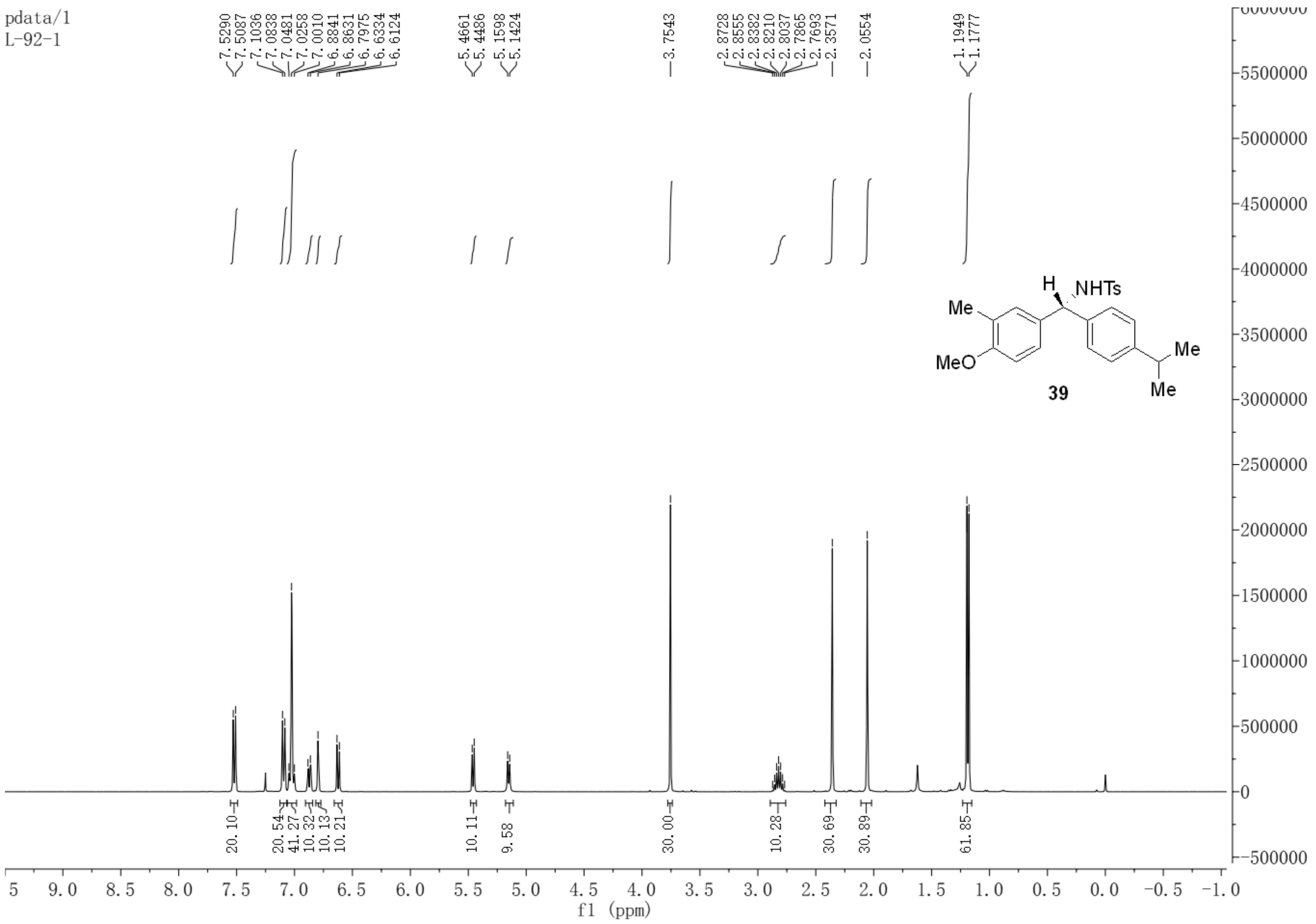

Supplementary Figure 80. ^{1}H NMR spectra of compound **39**

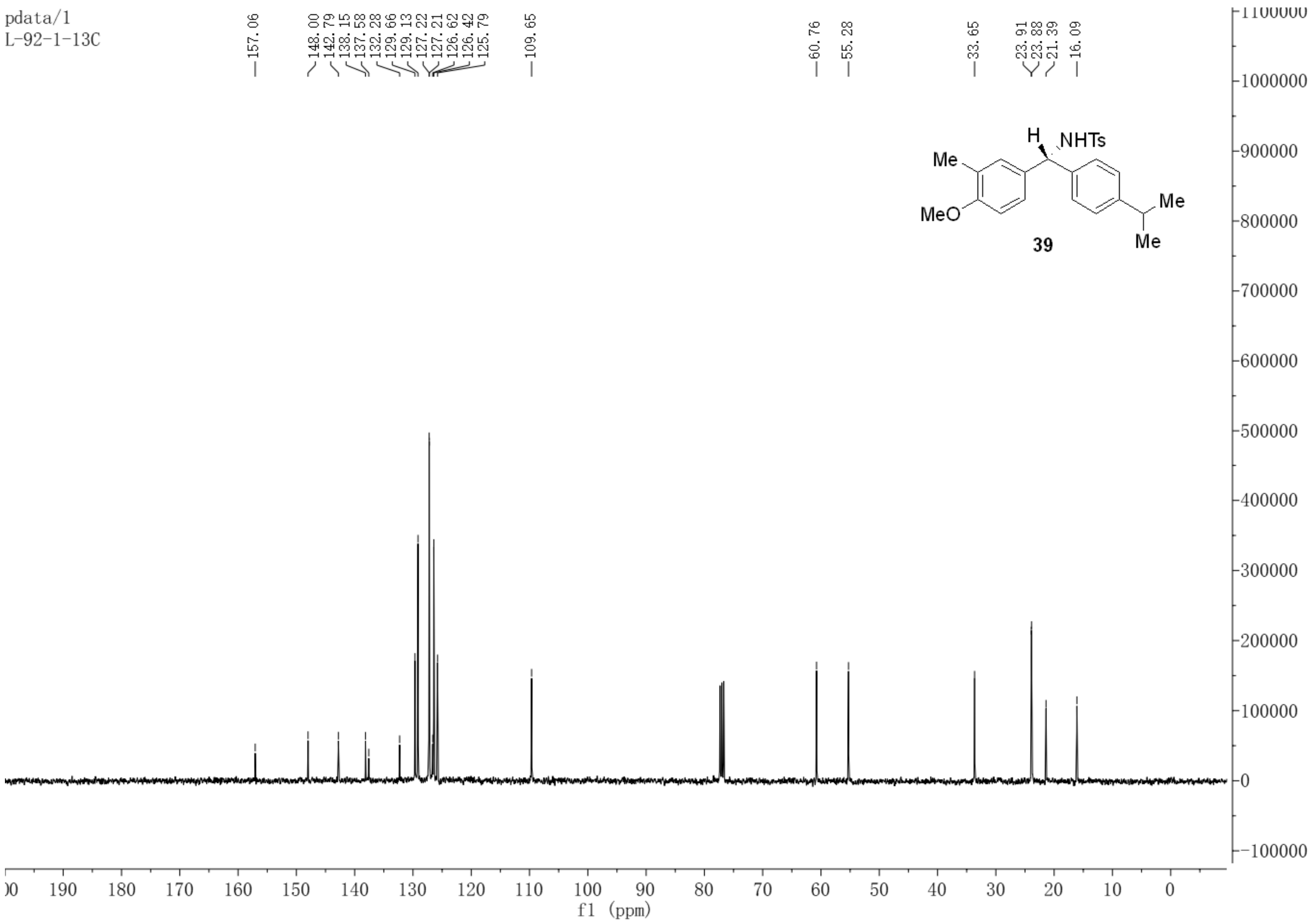

Supplementary Figure 81. ^{13}C NMR spectra of compound **39**

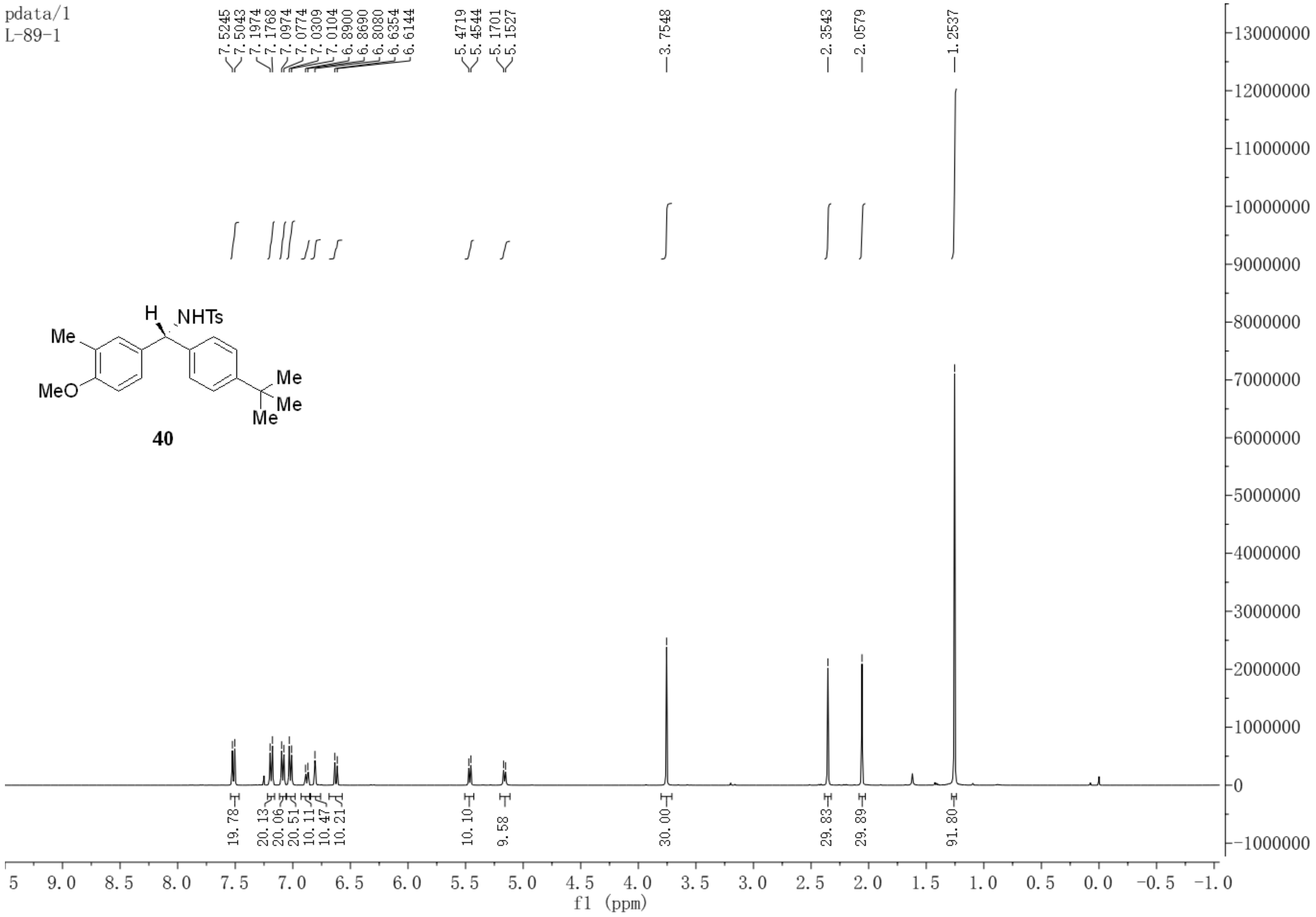

Supplementary Figure 82. ^{1}H NMR spectra of compound **40**

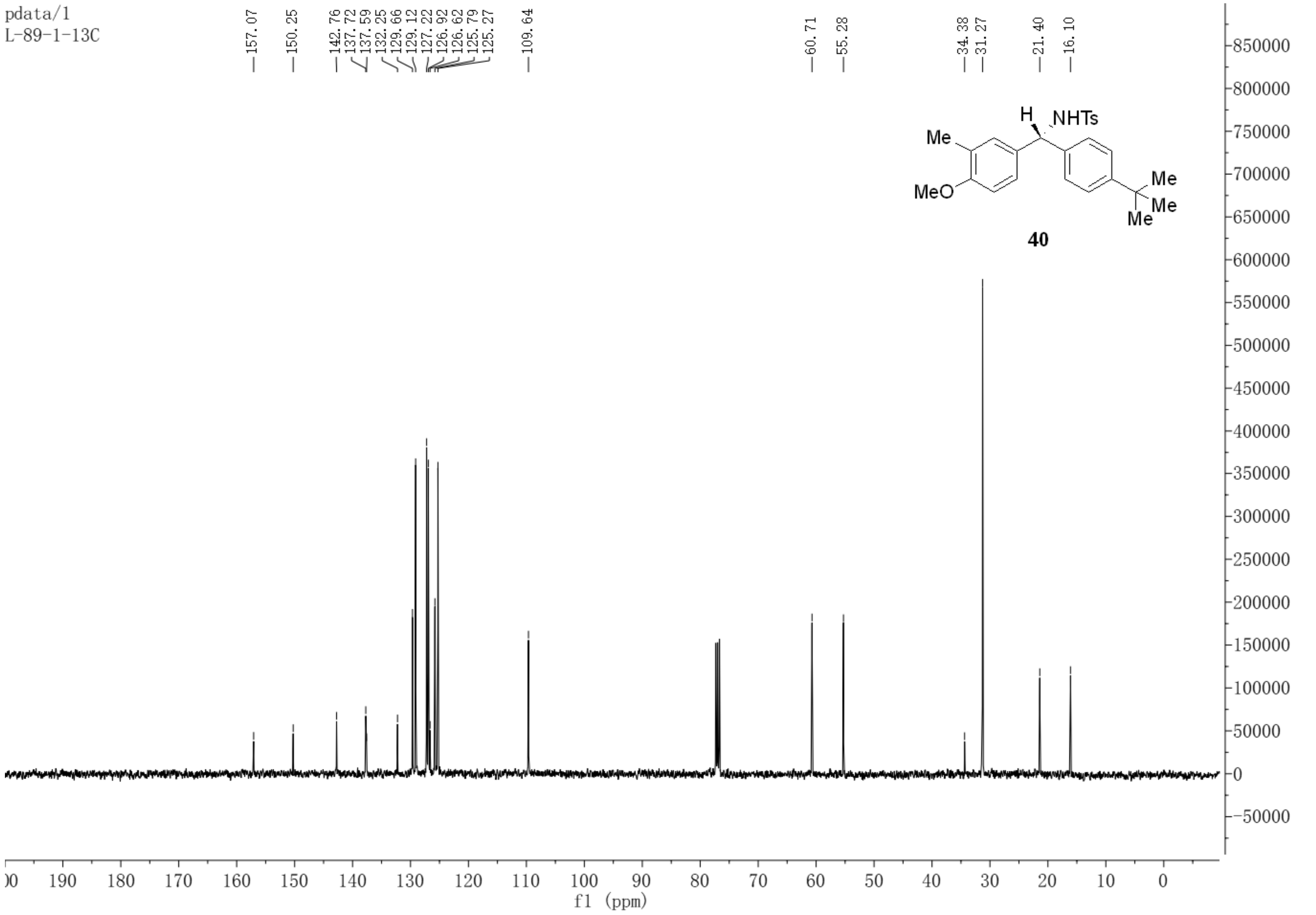

Supplementary Figure 83. ^{13}C NMR spectra of compound **40**

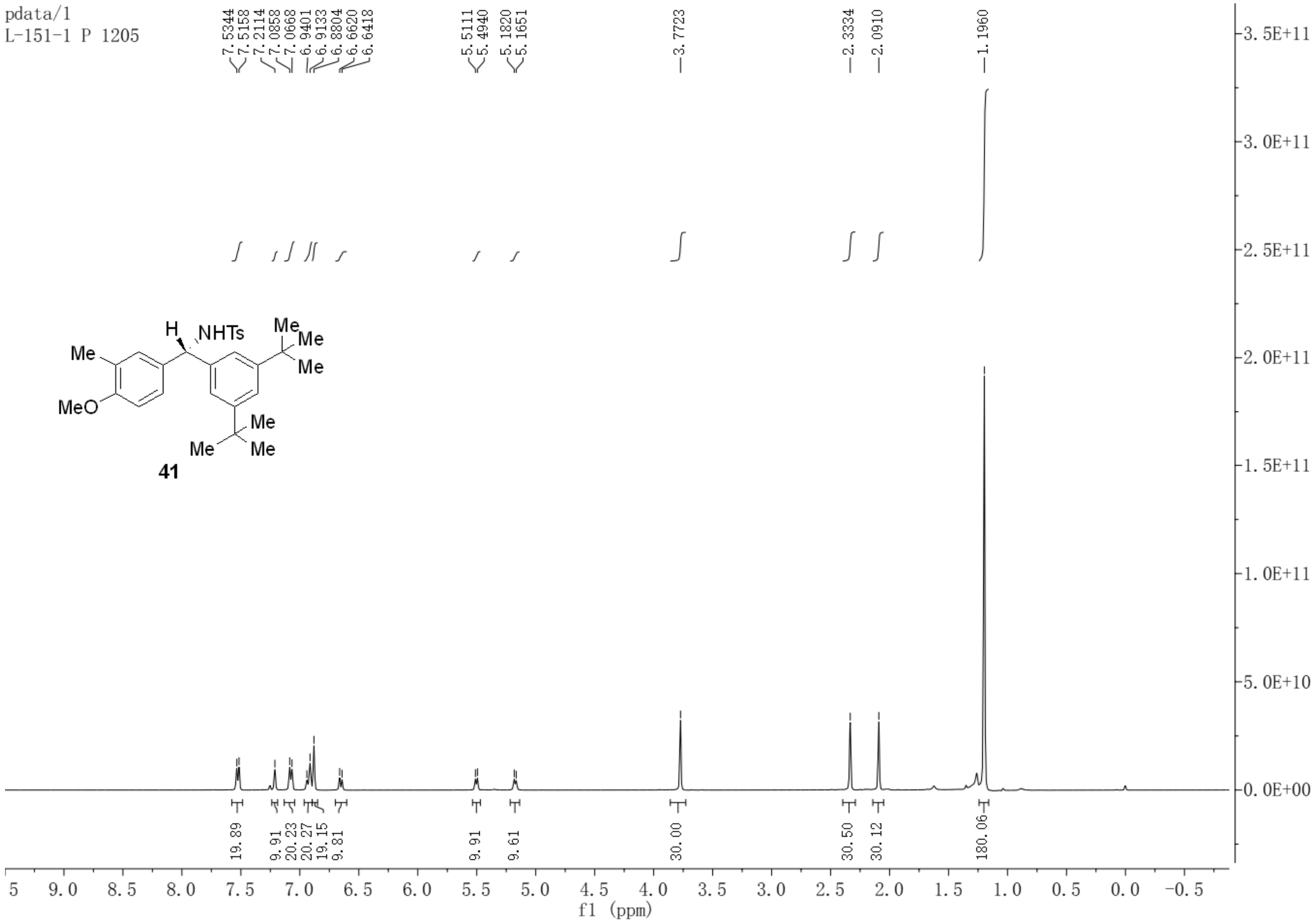

Supplementary Figure 84. ^{1}H NMR spectra of compound **41**

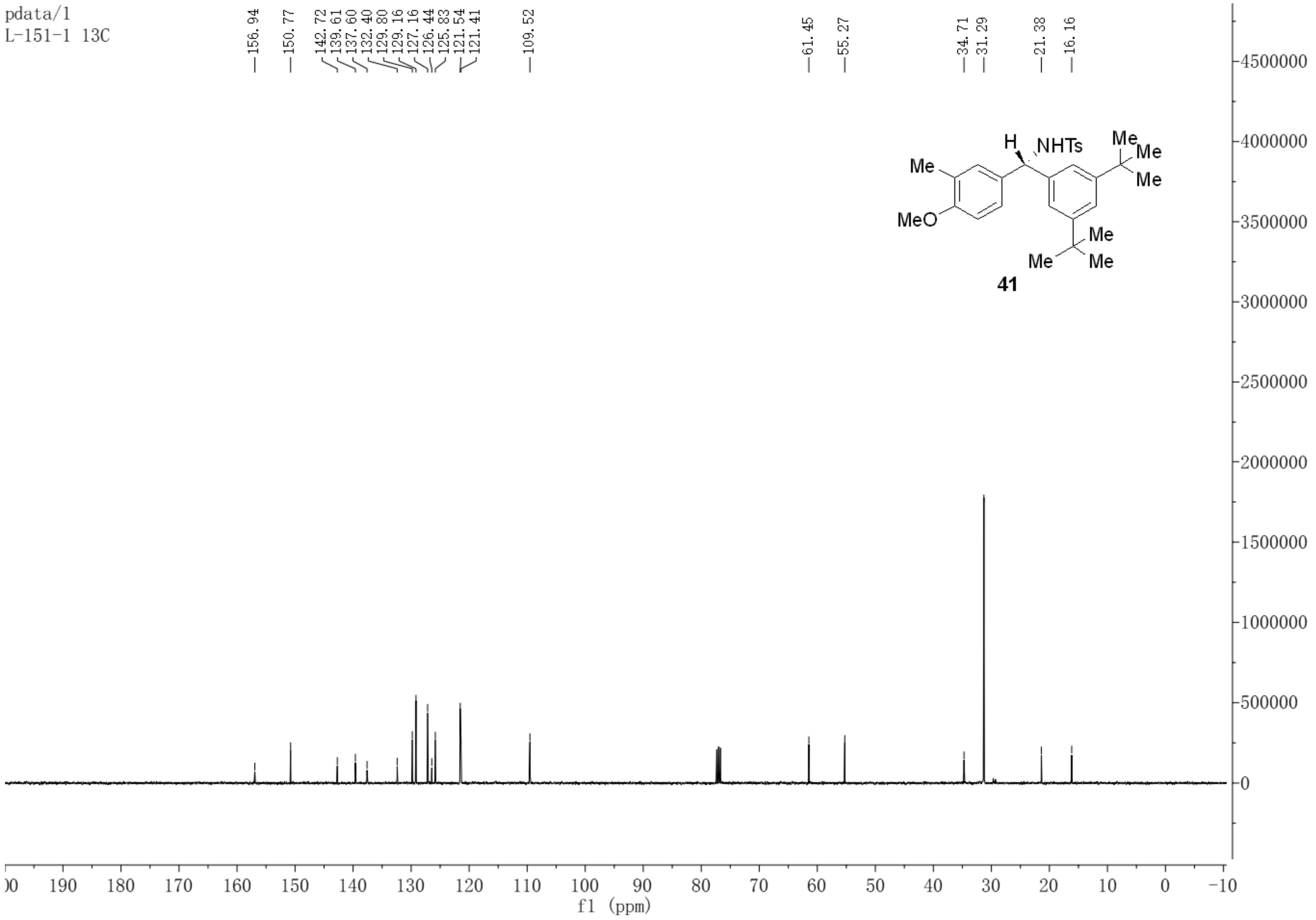

Supplementary Figure 85. ^{13}C NMR spectra of compound **41**

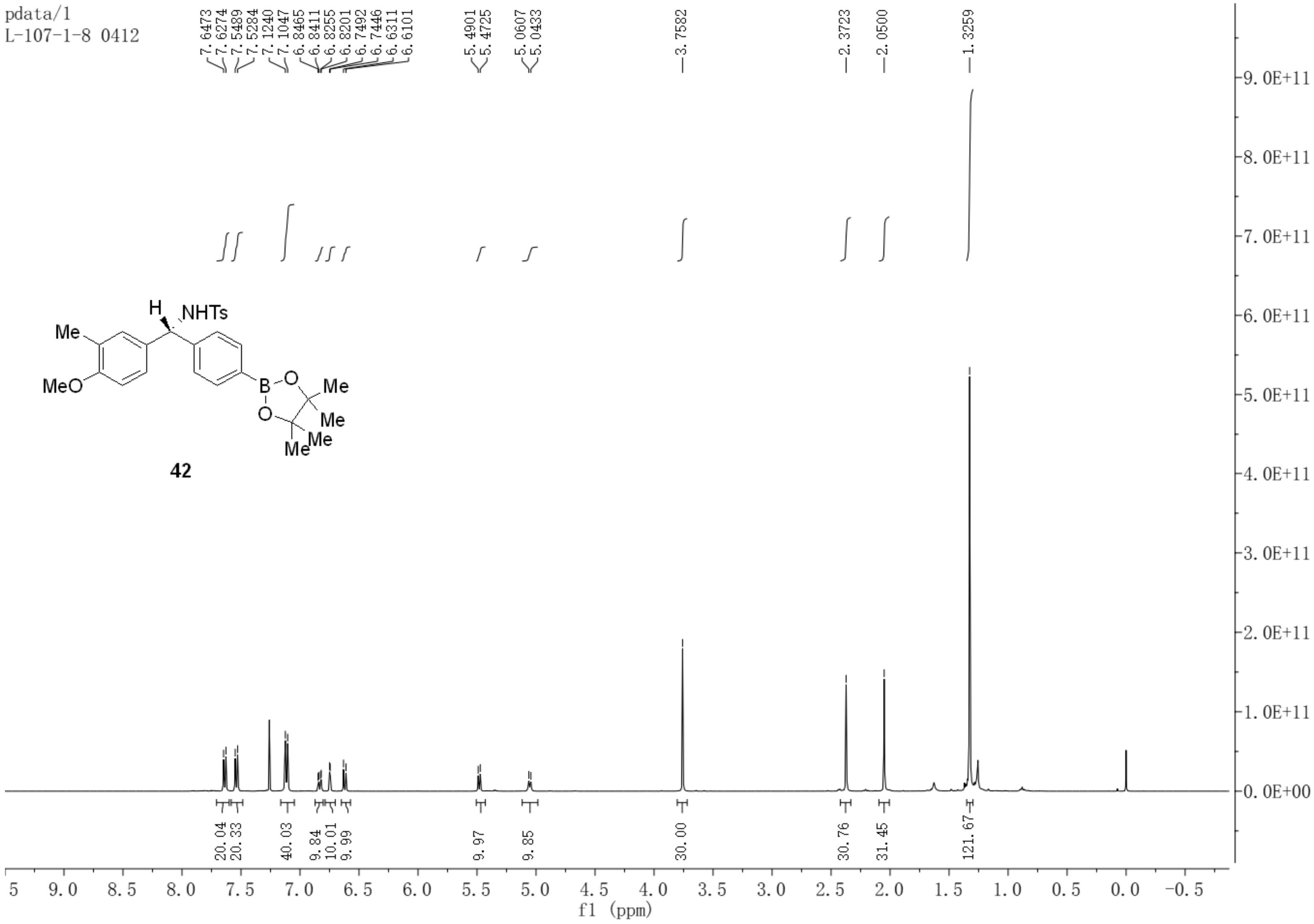

Supplementary Figure 86. ^{1}H NMR spectra of compound **42**

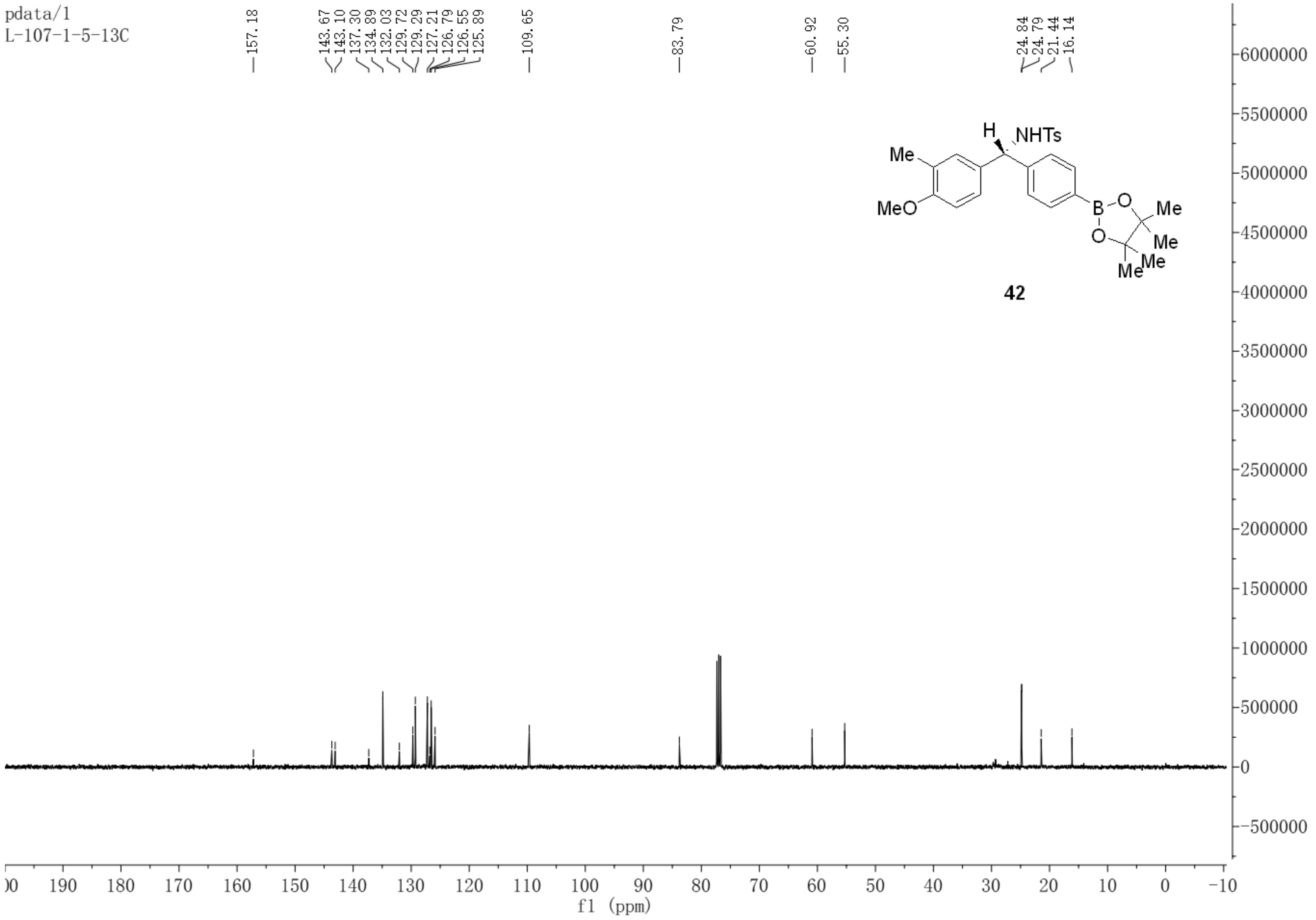

Supplementary Figure 87. ^{13}C NMR spectra of compound **42**

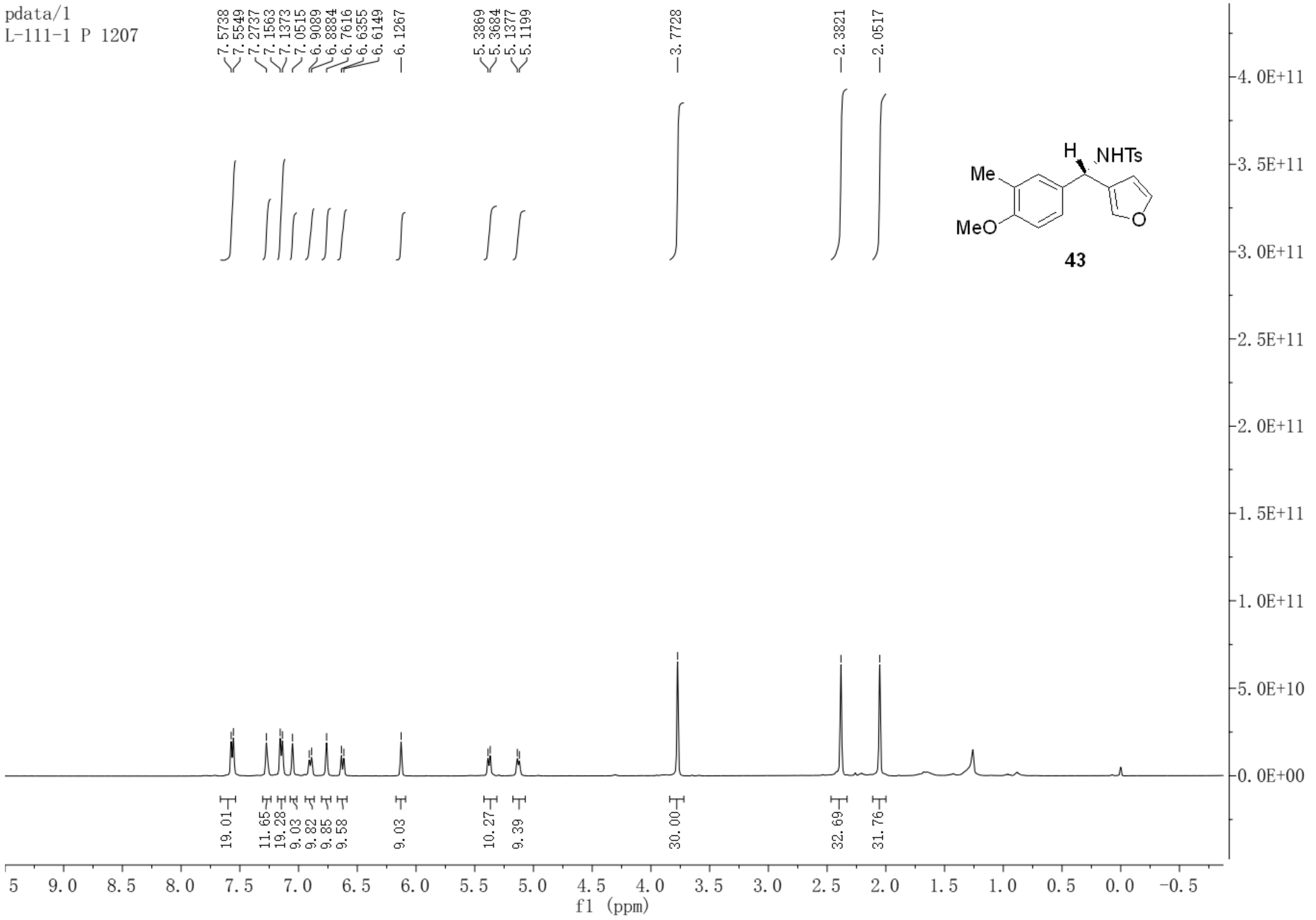

Supplementary Figure 88. ^{1}H NMR spectra of compound **43**

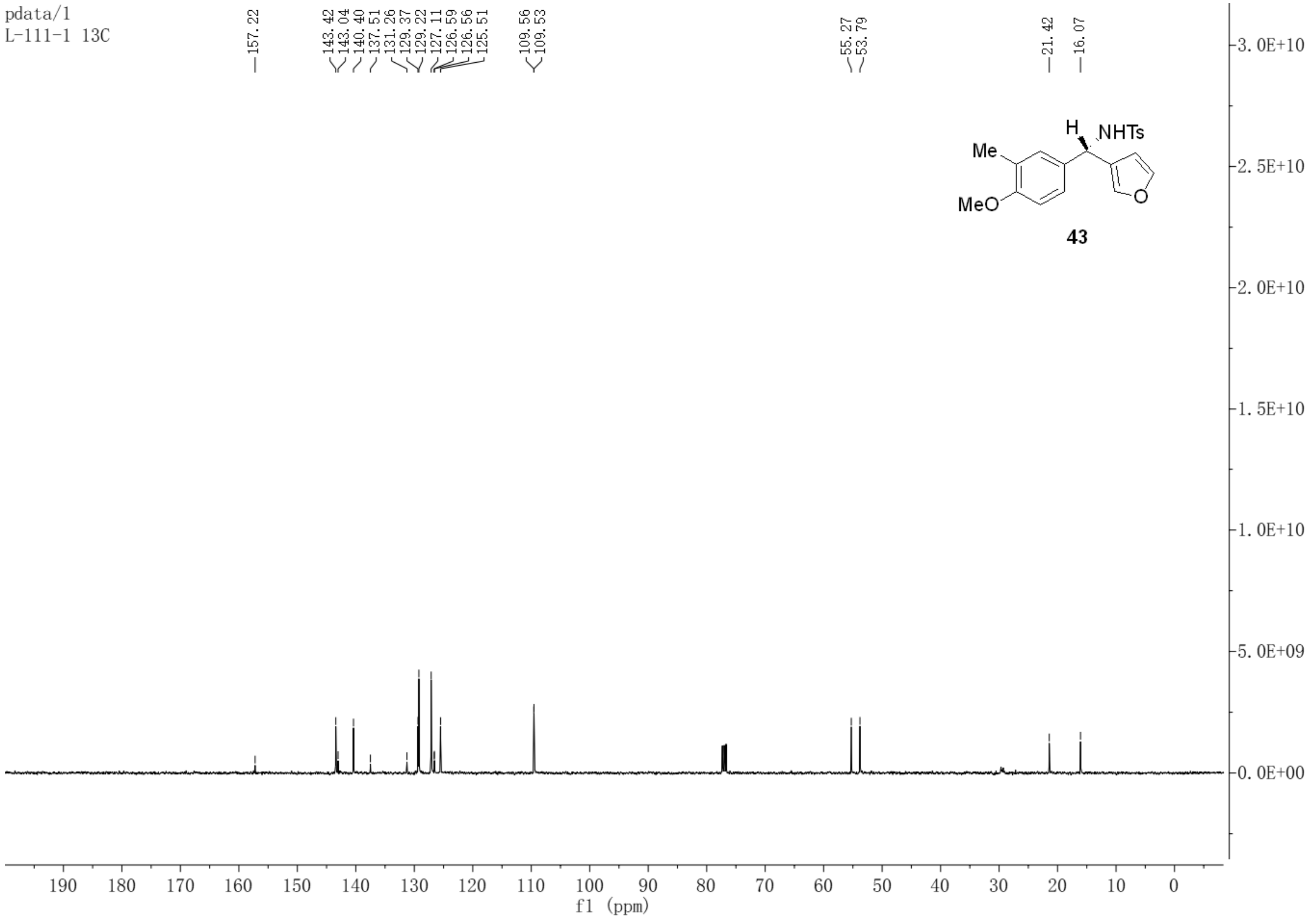

Supplementary Figure 89. ^{13}C NMR spectra of compound **43**

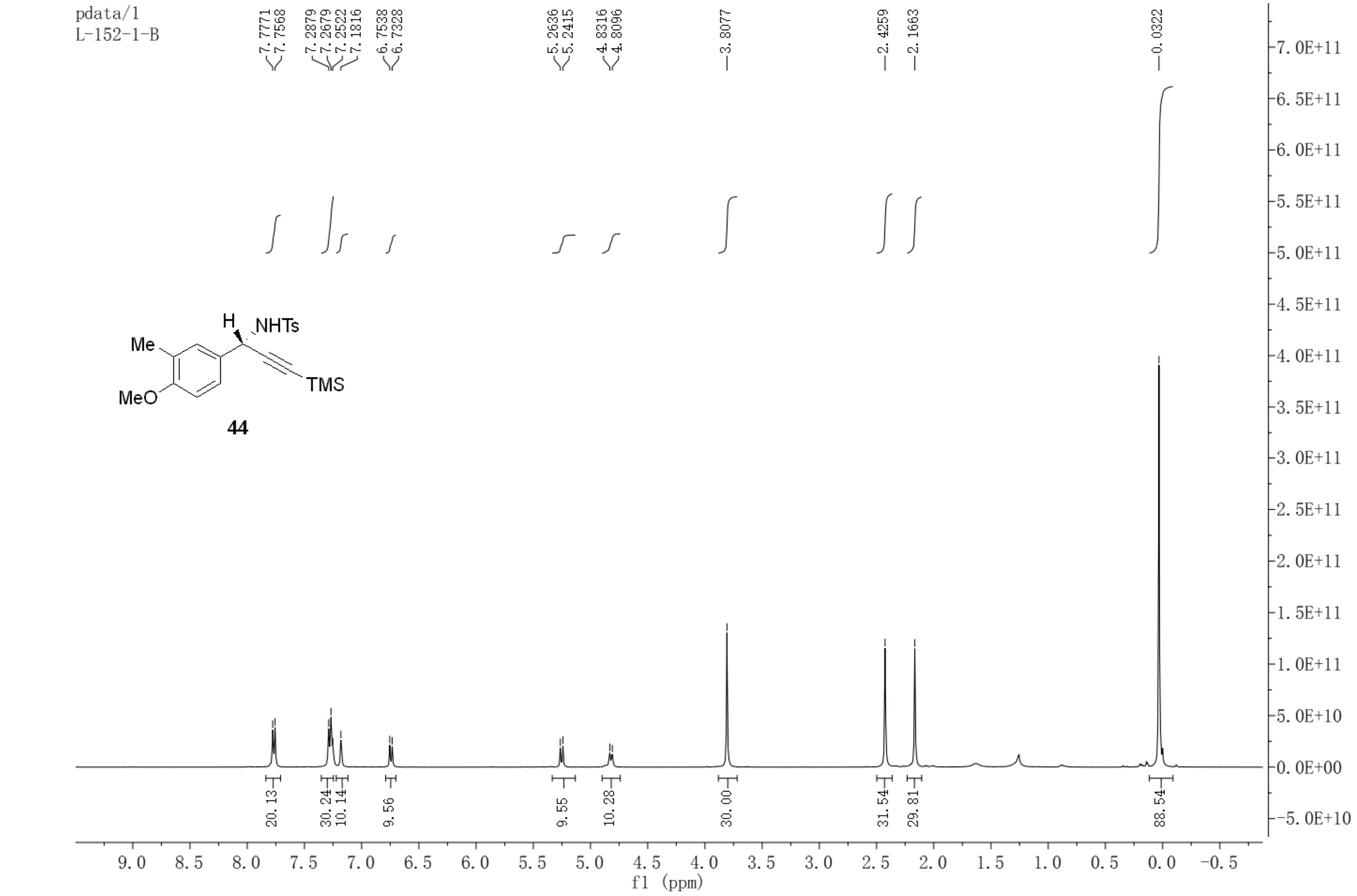

Supplementary Figure 90. ^{1}H NMR spectra of compound **44**

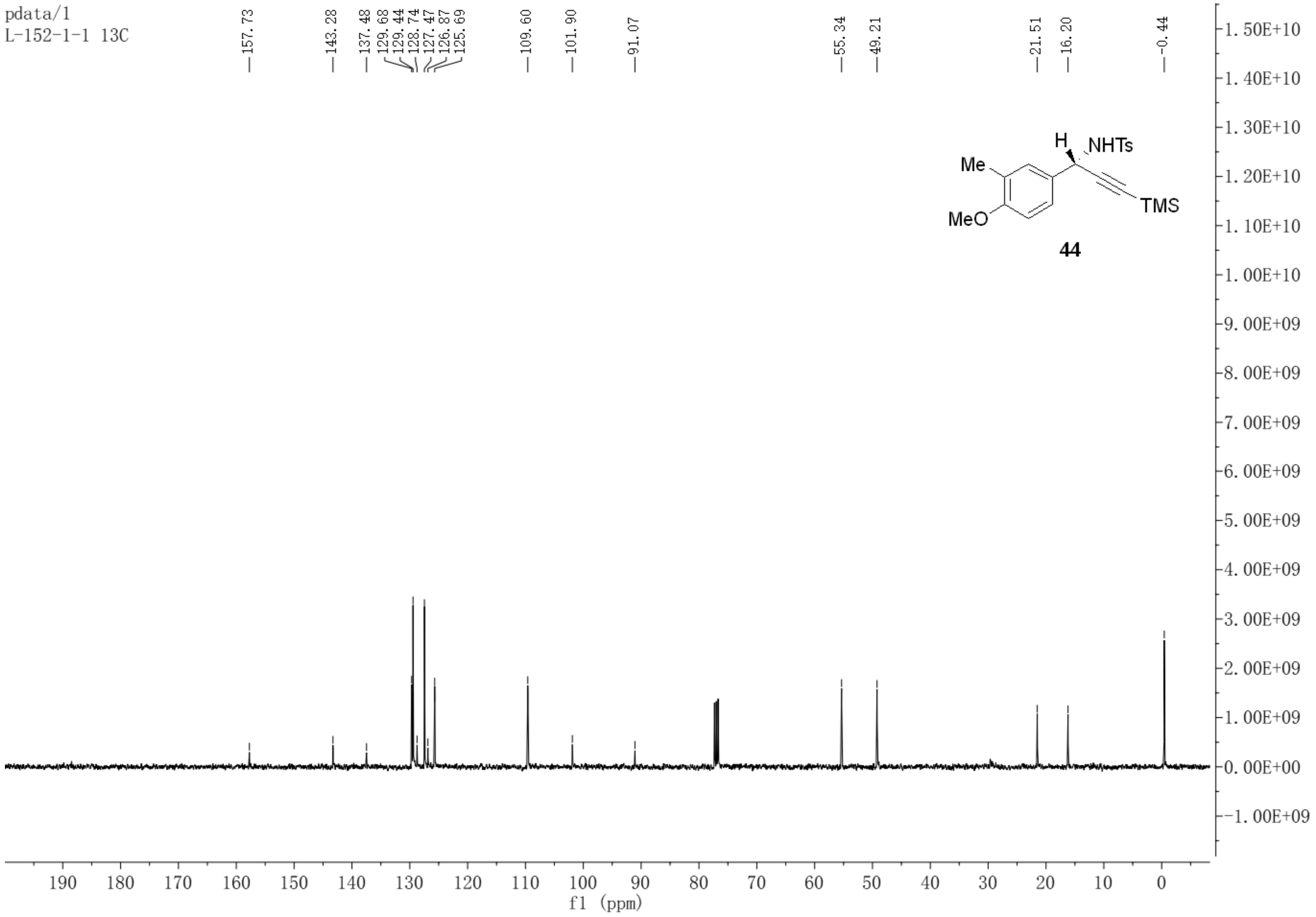

Supplementary Figure 91. ^{13}C NMR spectra of compound **44**

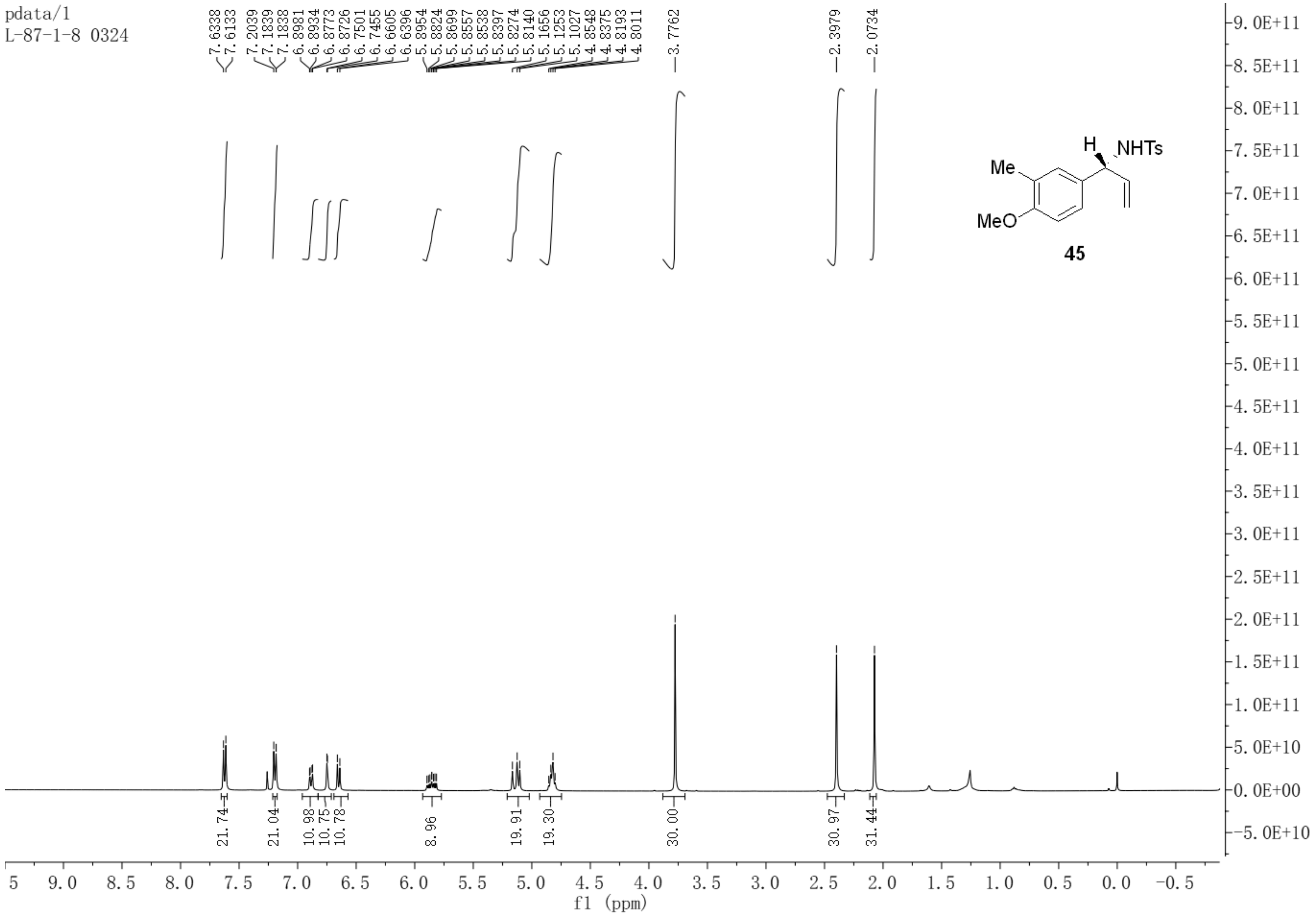

Supplementary Figure 92. ^{1}H NMR spectra of compound **45**

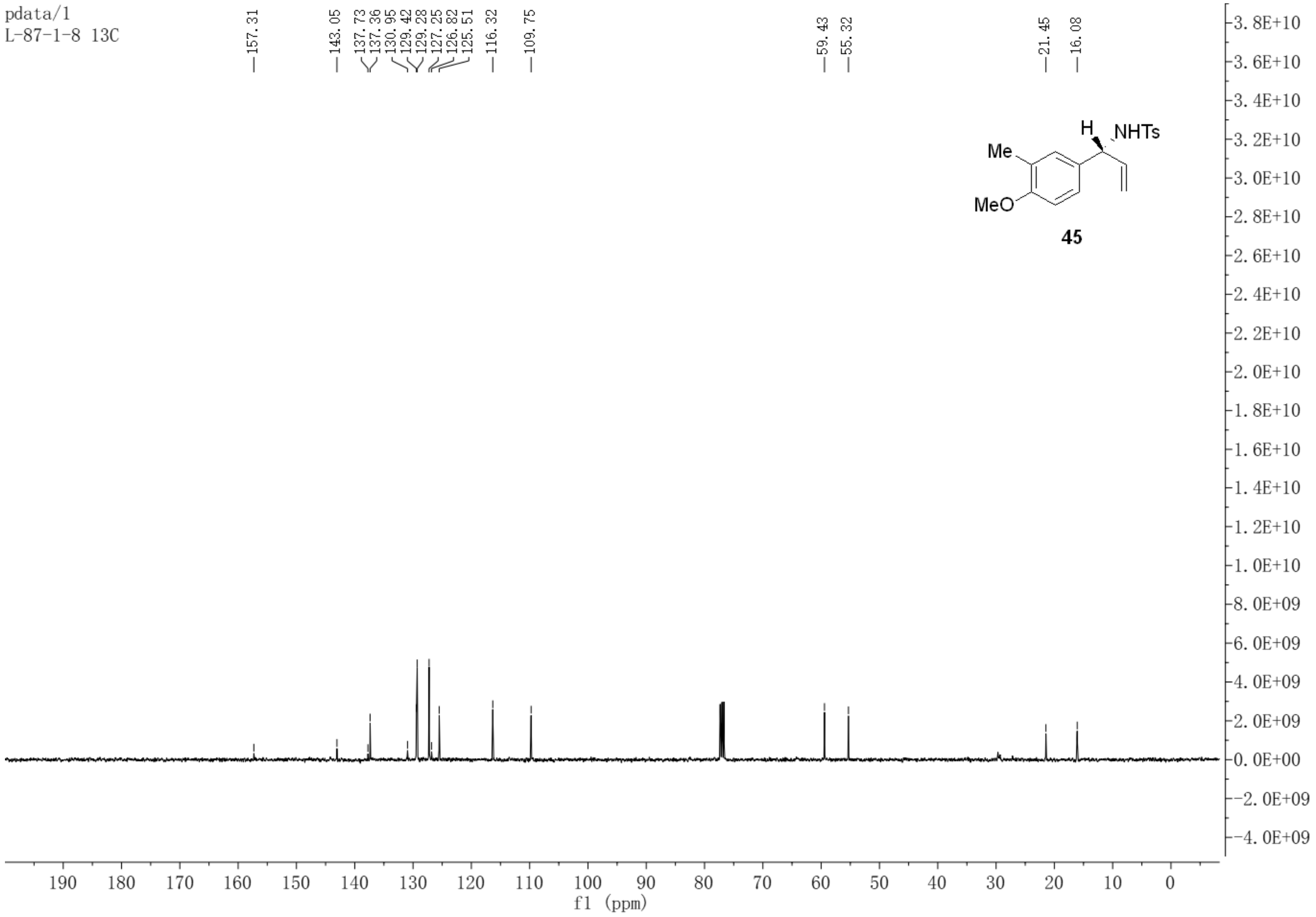

Supplementary Figure 93. ^{13}C NMR spectra of compound **45**

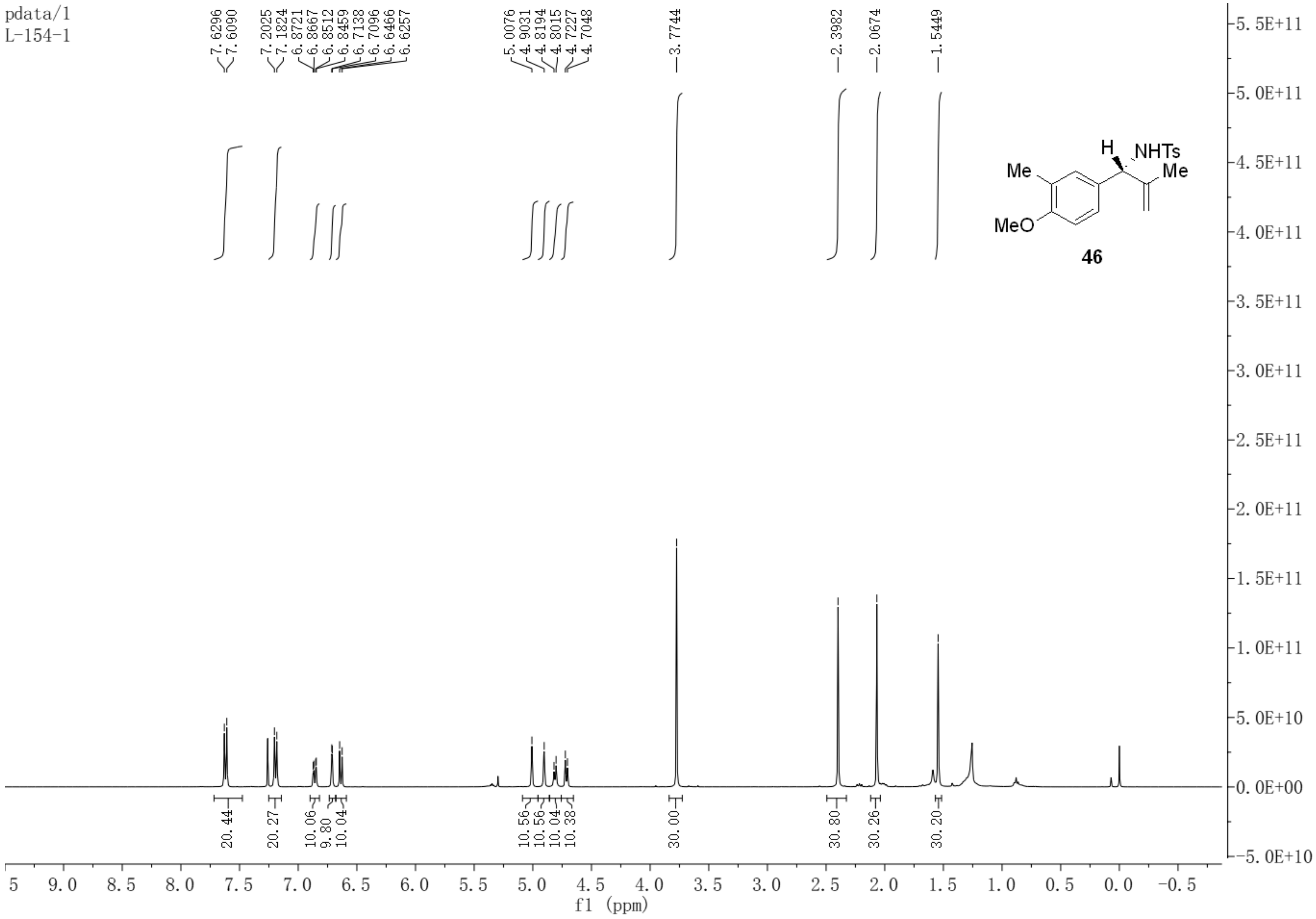

Supplementary Figure 94. ^{1}H NMR spectra of compound **46**

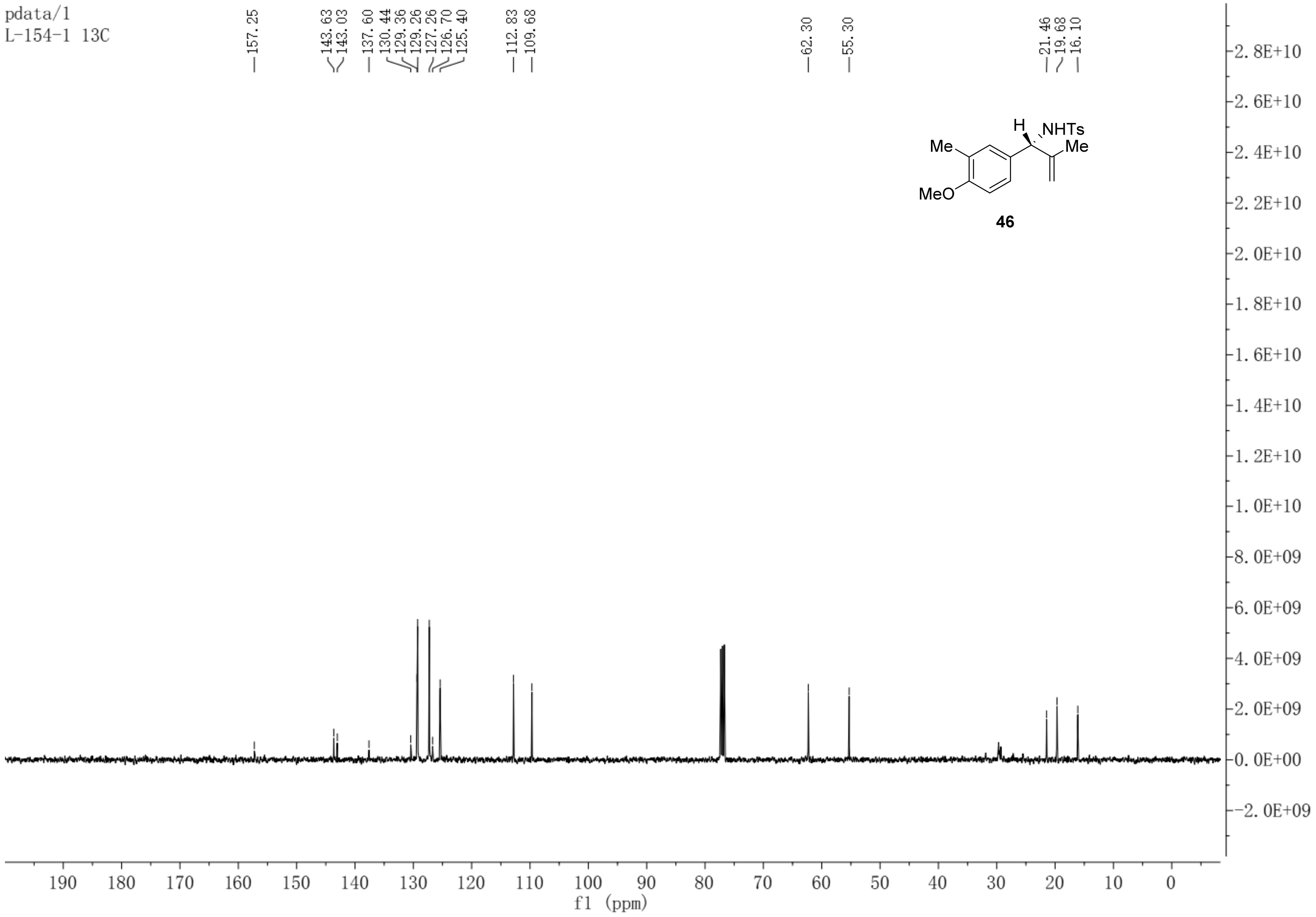

Supplementary Figure 95. ^{13}C NMR spectra of compound **46**

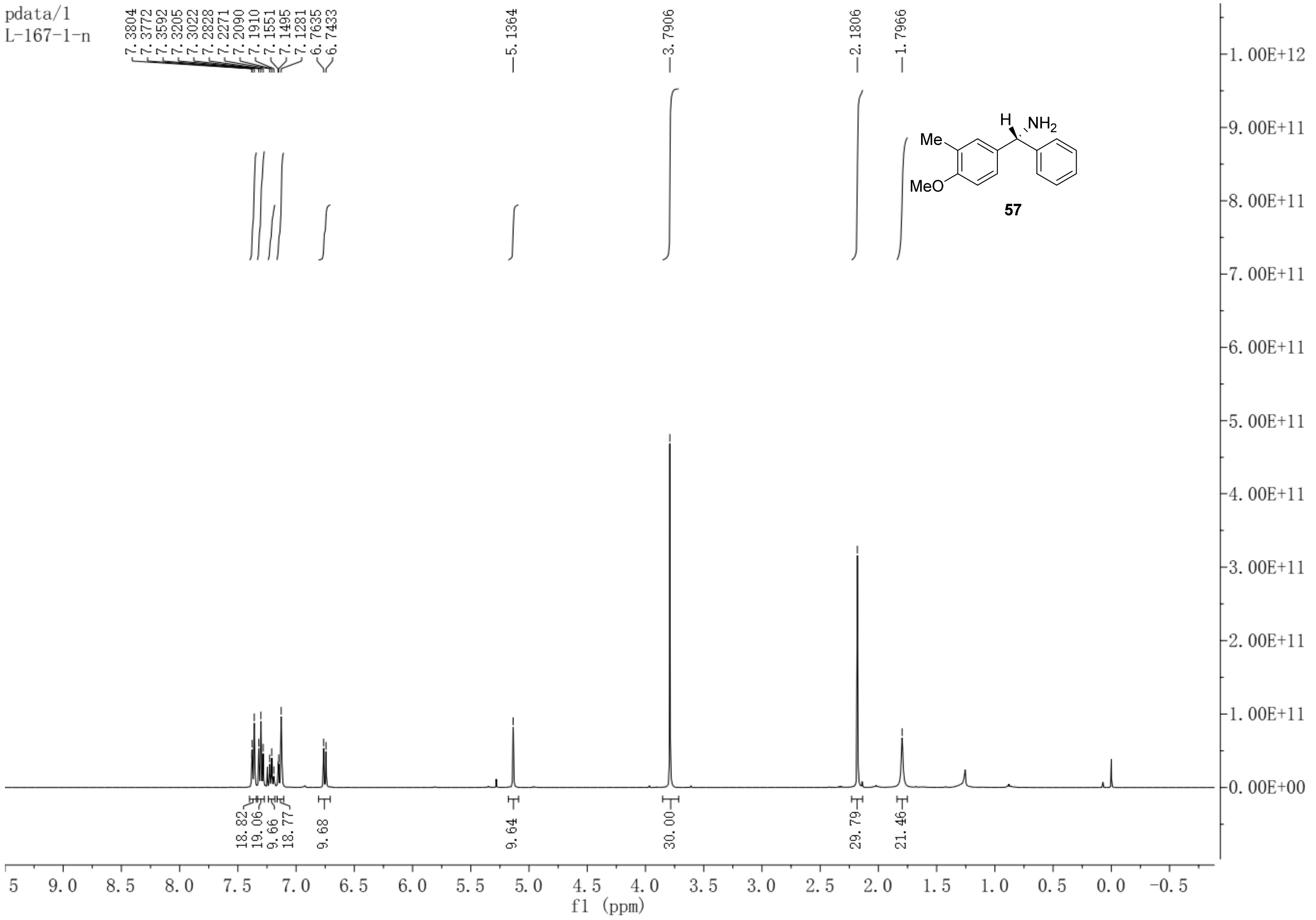

Supplementary Figure 96. ^{1}H NMR spectra of compound **57**

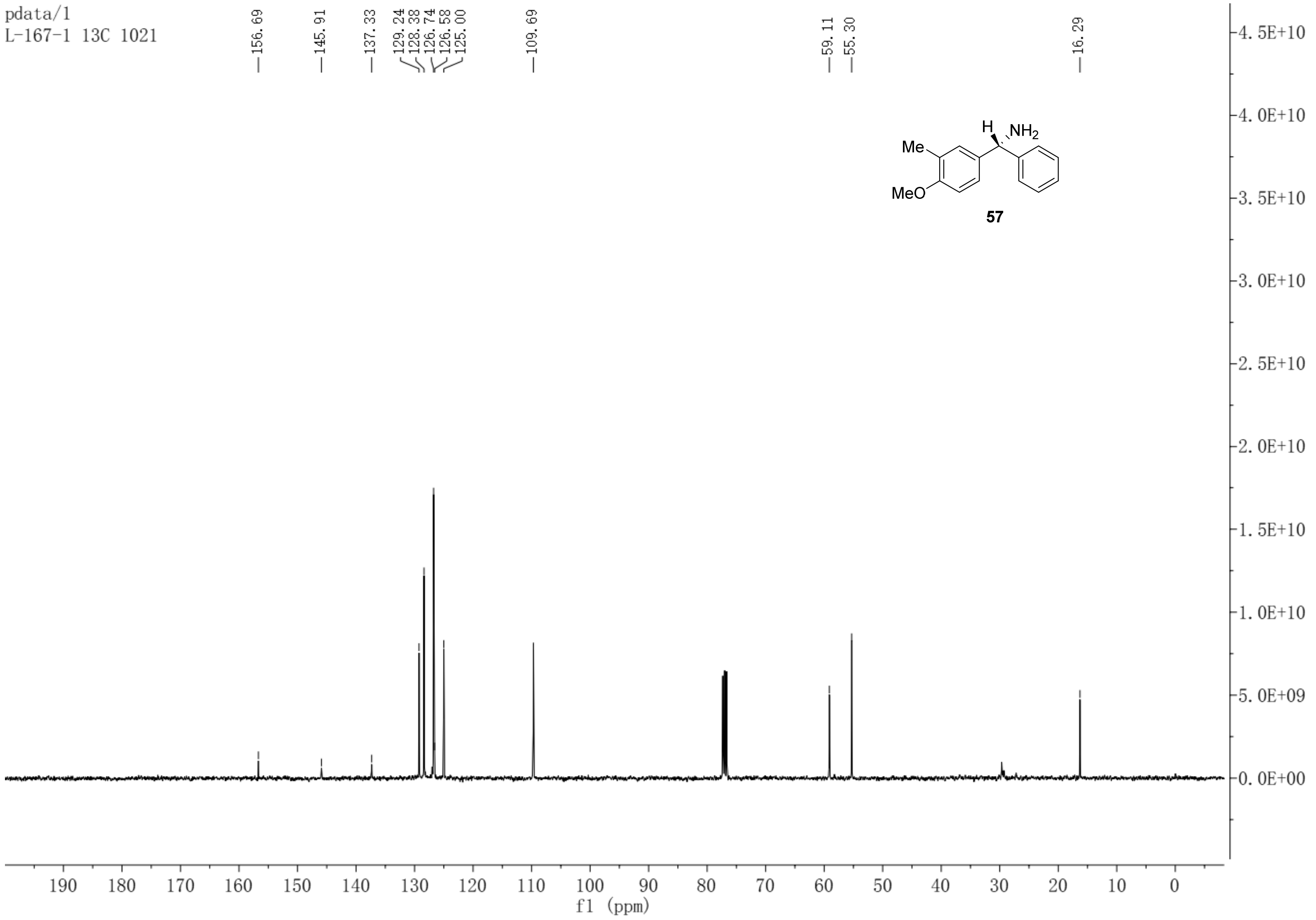

Supplementary Figure 97. ^{13}C NMR spectra of compound **57**

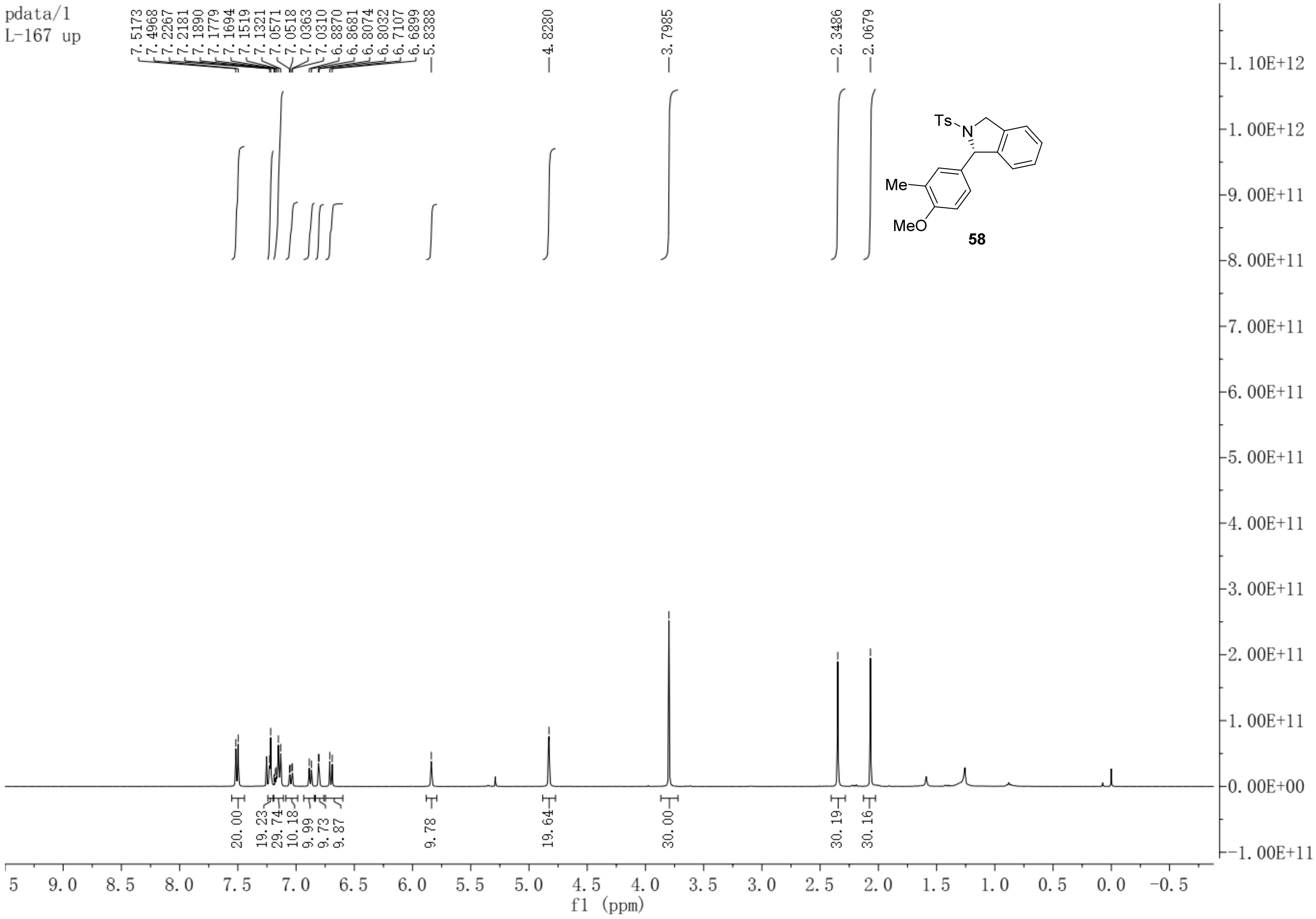

Supplementary Figure 98. ^{1}H NMR spectra of compound **58**

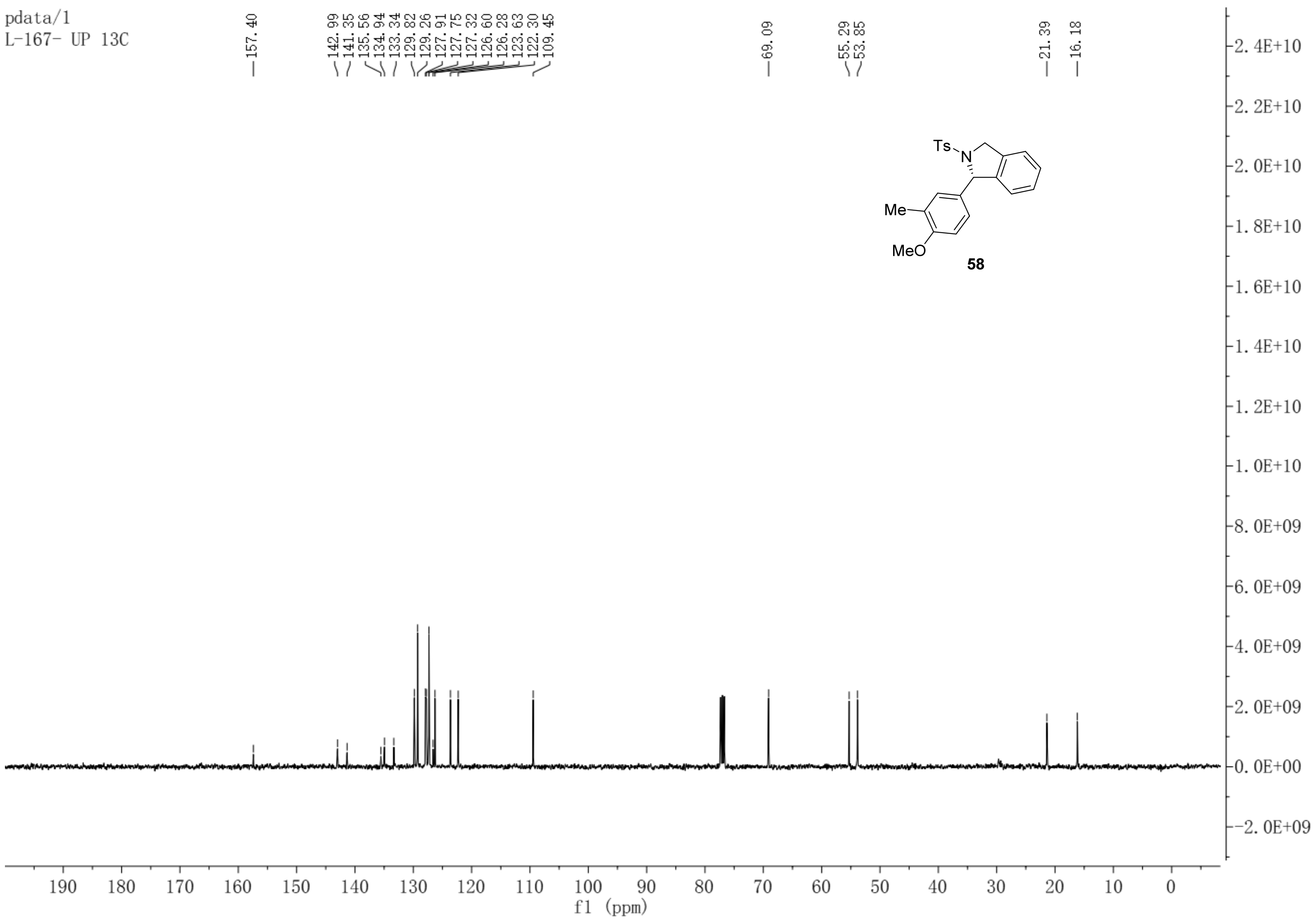

Supplementary Figure 99. ^{13}C NMR spectra of compound **58**

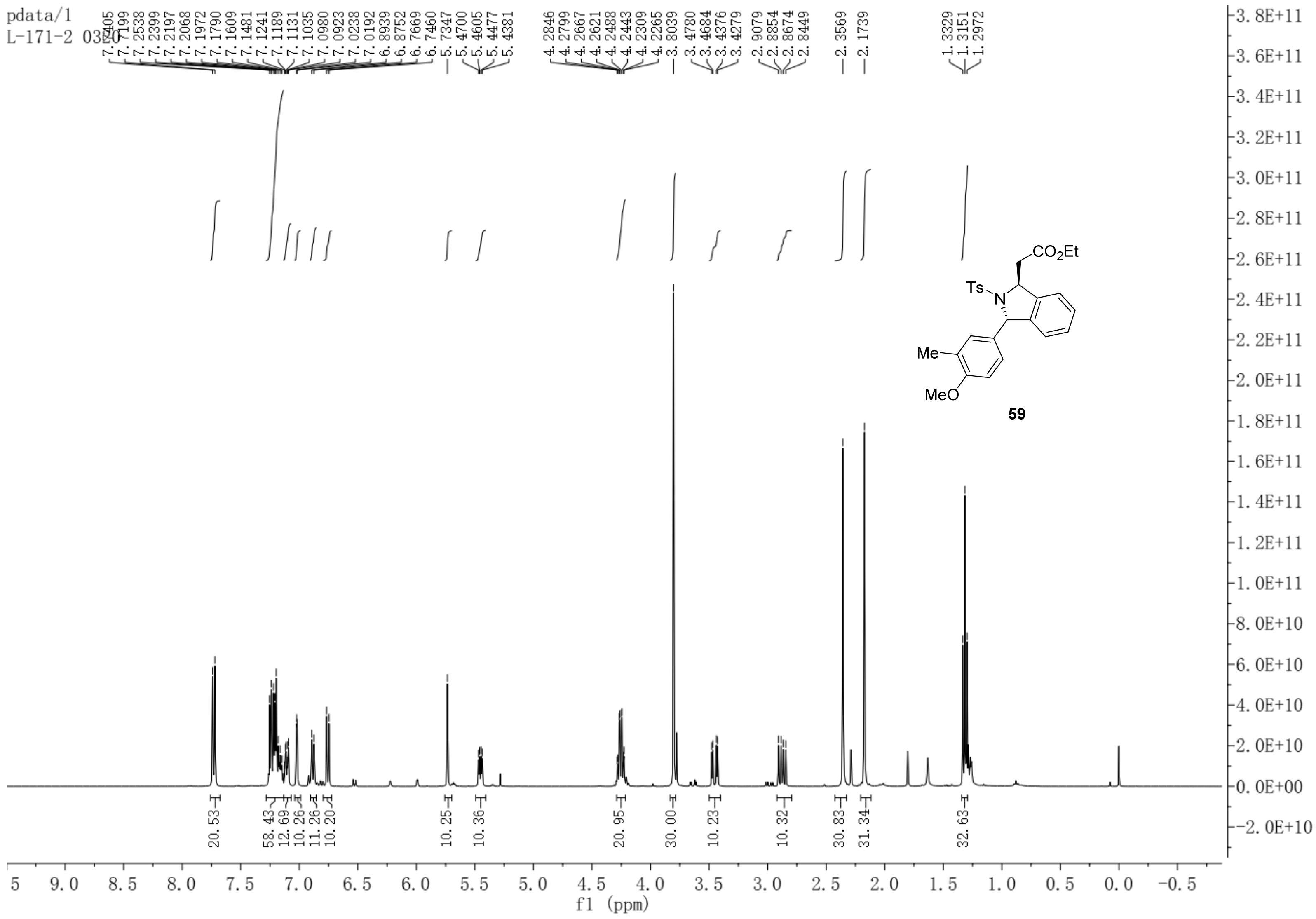

Supplementary Figure 100. ^{1}H NMR spectra of compound **59**

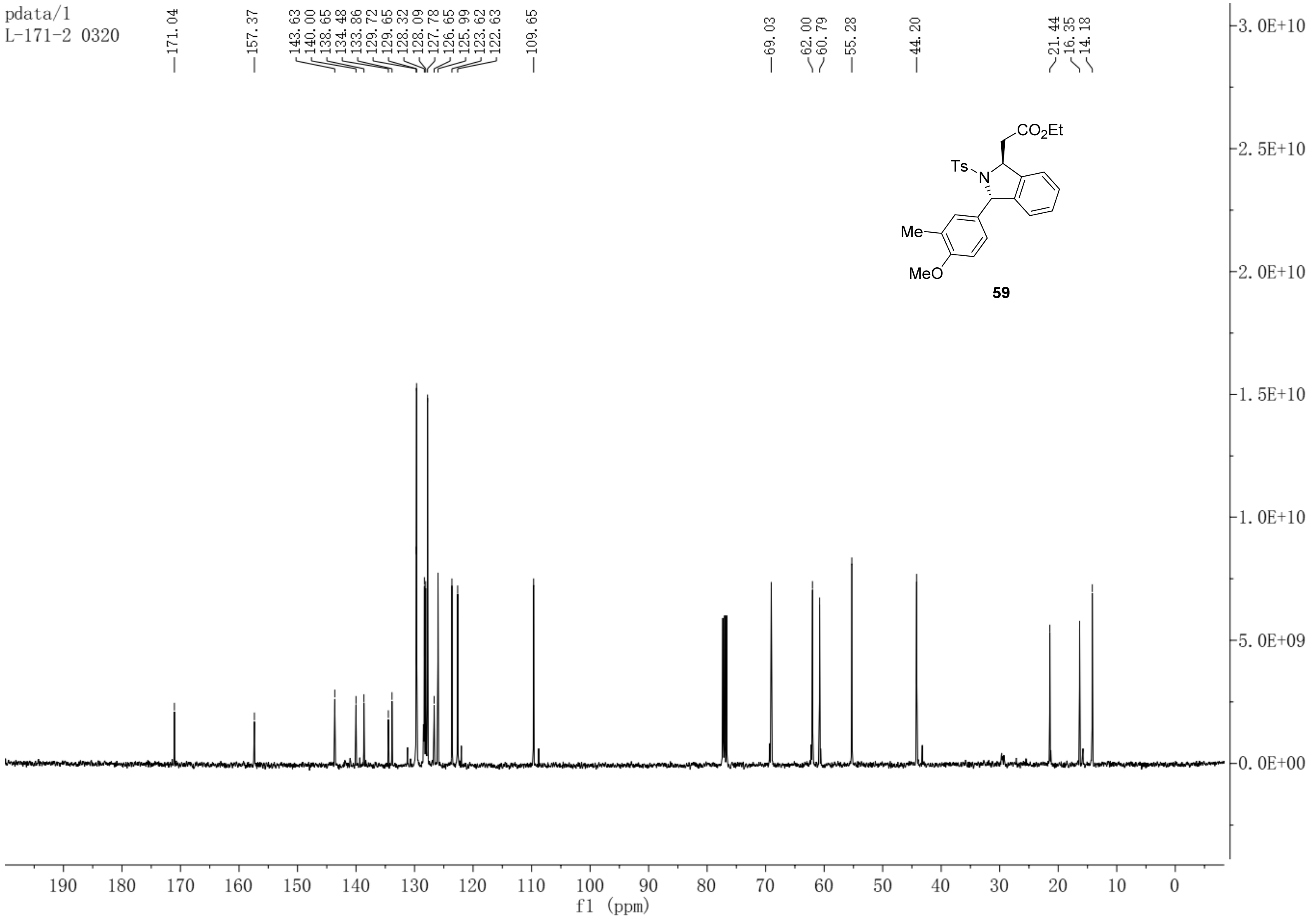

Supplementary Figure 101. ^{13}C NMR spectra of compound **59**

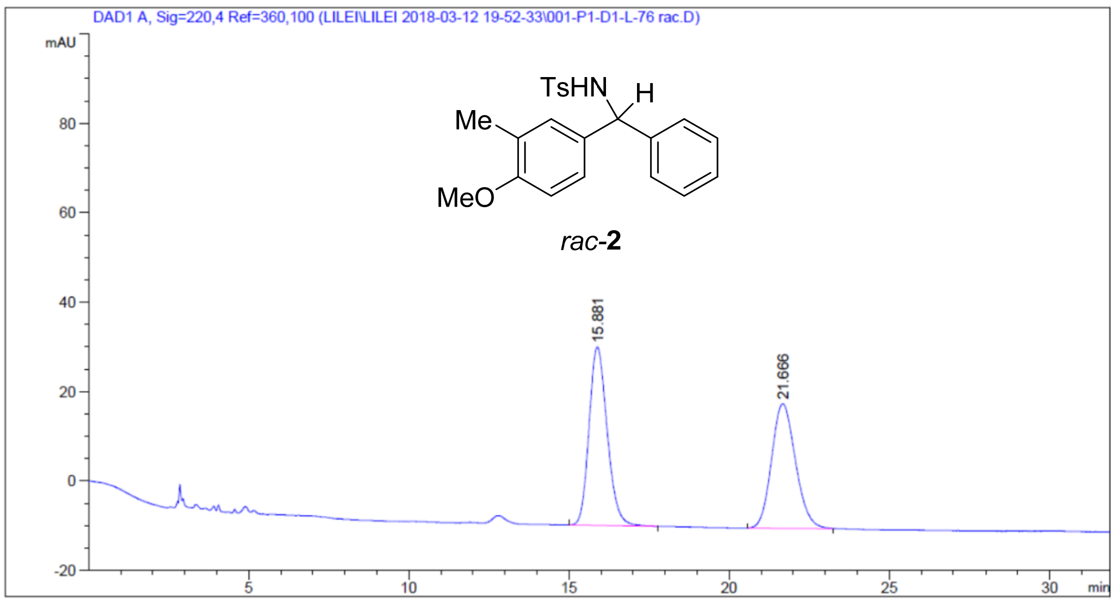

Peak #	RetTime [min]	Type	Width [min]	Area [mAU*s]	Height [mAU]	Area %
1	15.881	BB	0.5935	1573.48669	39.95135	52.1279
2	21.666	BB	0.7729	1445.02295	27.87187	47.8721

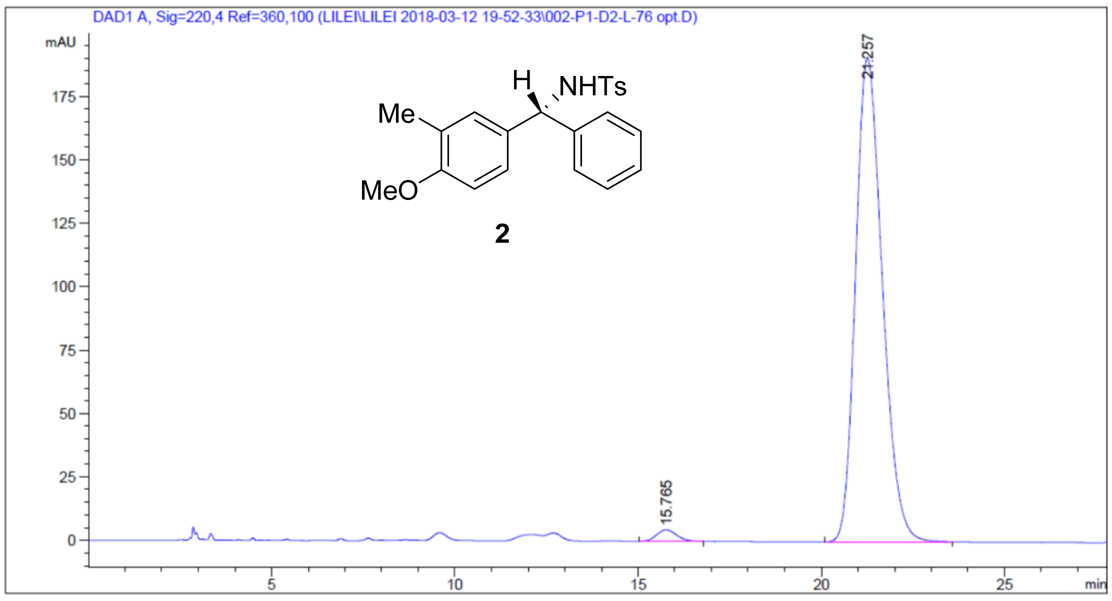

Peak #	RetTime [min]	Type	Width [min]	Area [mAU*s]	Height [mAU]	Area %
1	15.765	BB	0.4685	176.55936	4.59319	1.7879
2	21.257	BB	0.7848	9698.86133	190.88823	98.2121

Supplementary Figure 102. HPLC spectra of compound *rac*-**2** and compound **2**

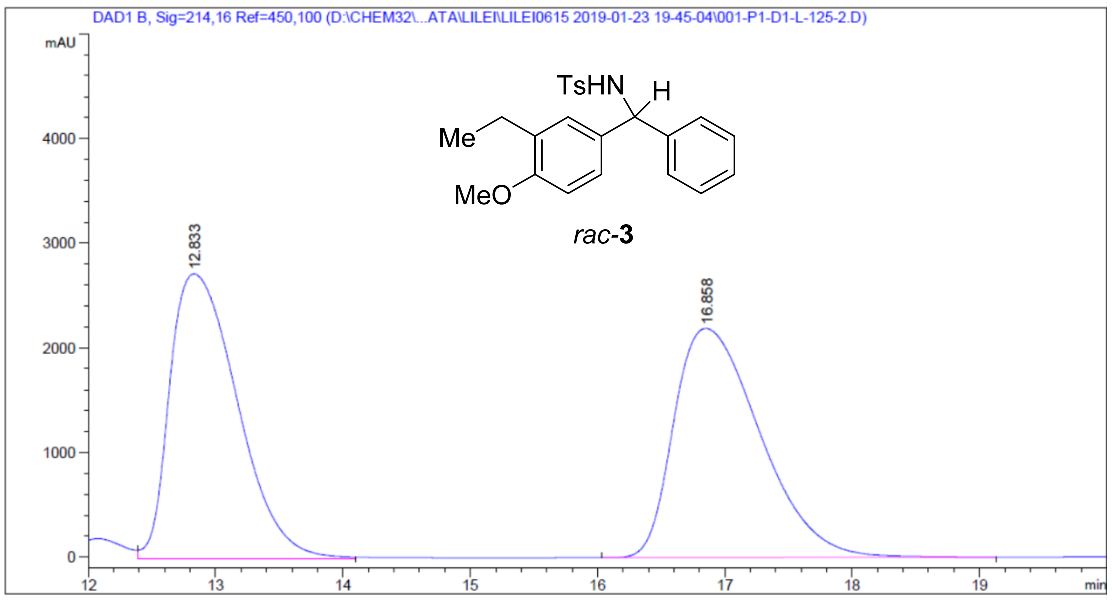

Peak #	RetTime [min]	Type	Width [min]	Area [mAU*s]	Height [mAU]	Area %
1	12.833	MM	0.6156	1.00710e5	2726.58862	49.4379
2	16.858	BV R	0.5536	1.03000e5	2192.43750	50.5621

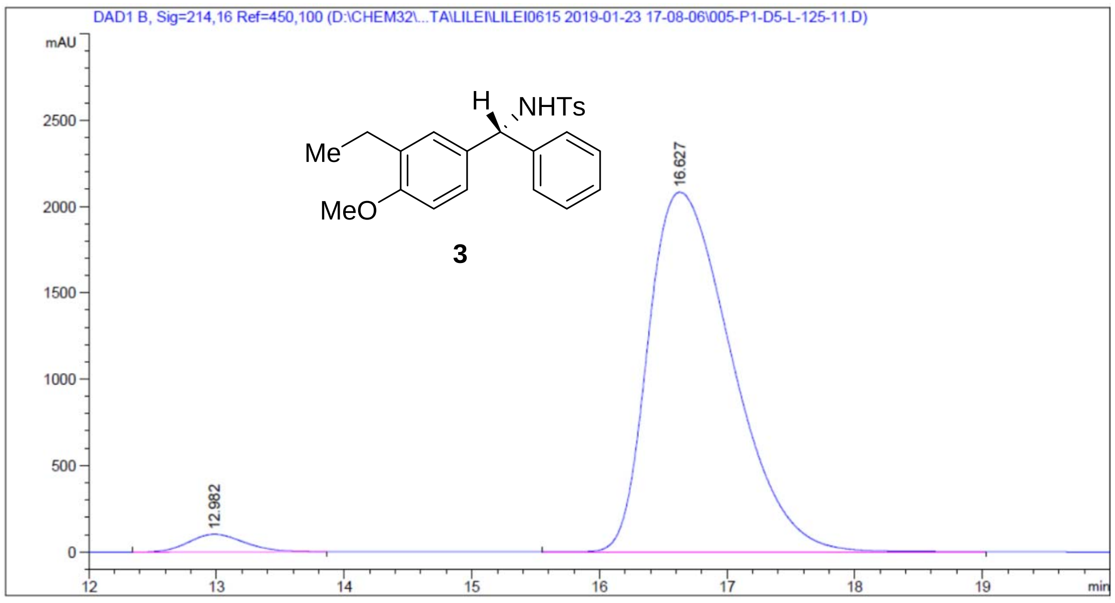

Peak #	RetTime [min]	Type	Width [min]	Area [mAU*s]	Height [mAU]	Area %
1	12.982	BB	0.3856	3128.72363	102.37308	3.2031
2	16.627	MM	0.7560	9.45487e4	2084.46899	96.7969

Supplementary Figure 103. HPLC spectra of compound *rac*-**3** and compound **3**

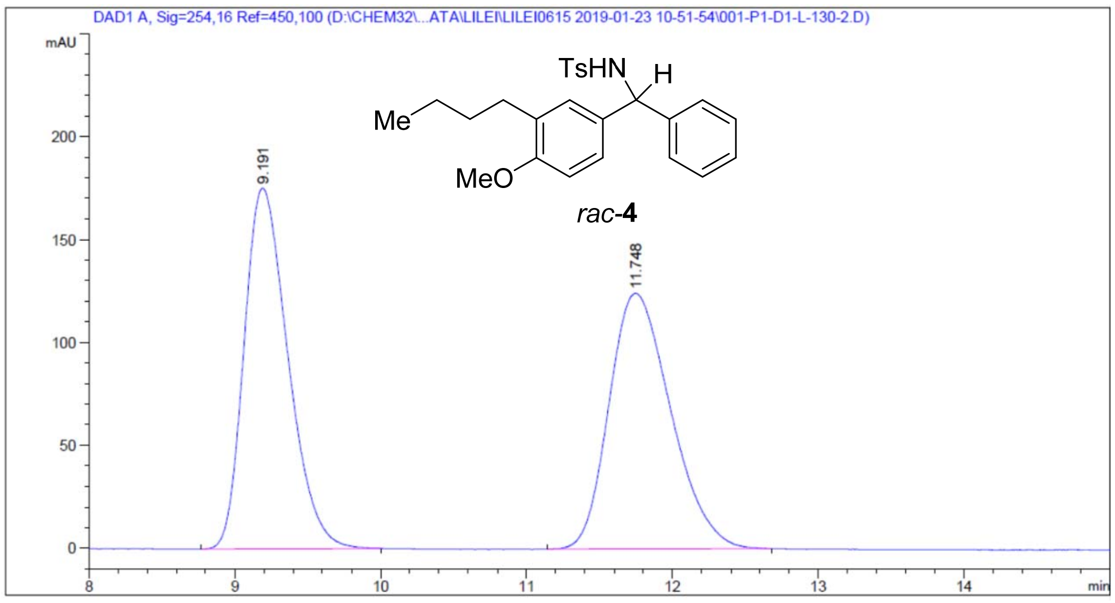

Peak #	RetTime [min]	Type	Width [min]	Area [mAU*s]	Height [mAU]	Area %
1	9.191	BB	0.3183	3666.00830	175.45500	50.0612
2	11.748	BV R	0.4168	3657.04175	124.29074	49.9388

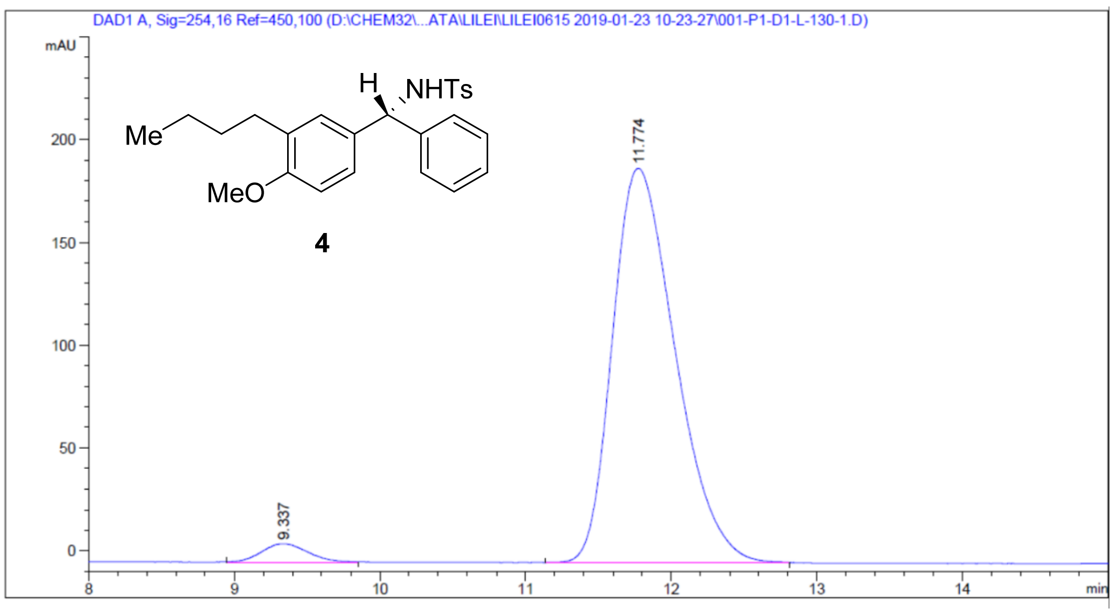

Peak #	RetTime [min]	Type	Width [min]	Area [mAU*s]	Height [mAU]	Area %
1	9.337	BV	0.2585	194.88503	8.91796	3.2878
2	11.774	BB	0.4361	5732.56299	191.84917	96.7122

Supplementary Figure 104. HPLC spectra of compound *rac*-**4** and compound **4**

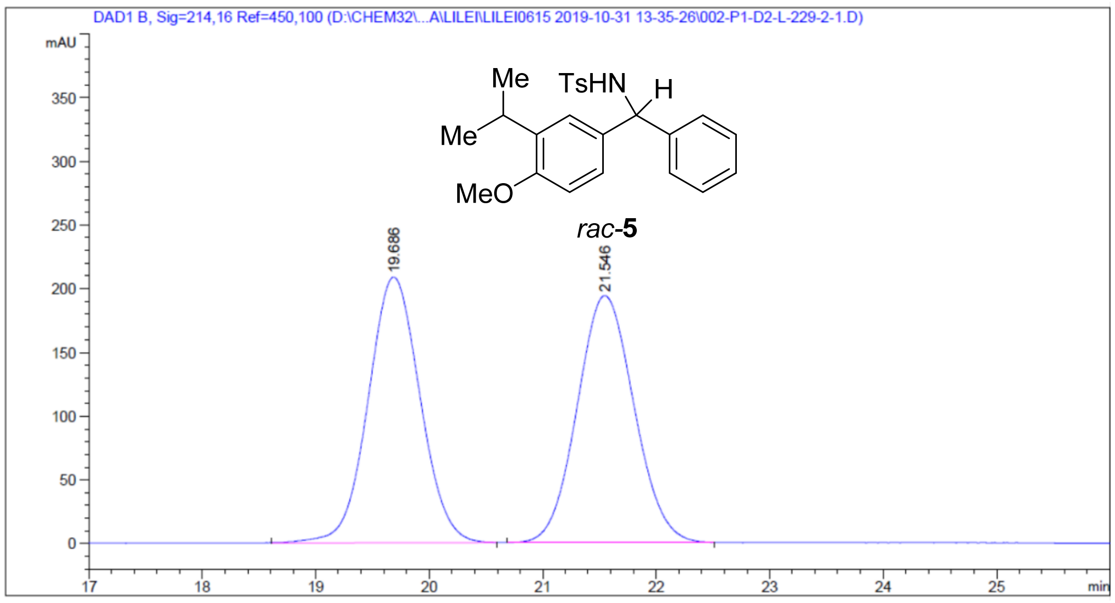

Peak #	RetTime [min]	Type	Width [min]	Area [mAU*s]	Height [mAU]	Area %
1	19.686	VV R	0.4758	6634.39746	208.46942	49.5759
2	21.546	VB R	0.4767	6747.90967	193.81253	50.4241

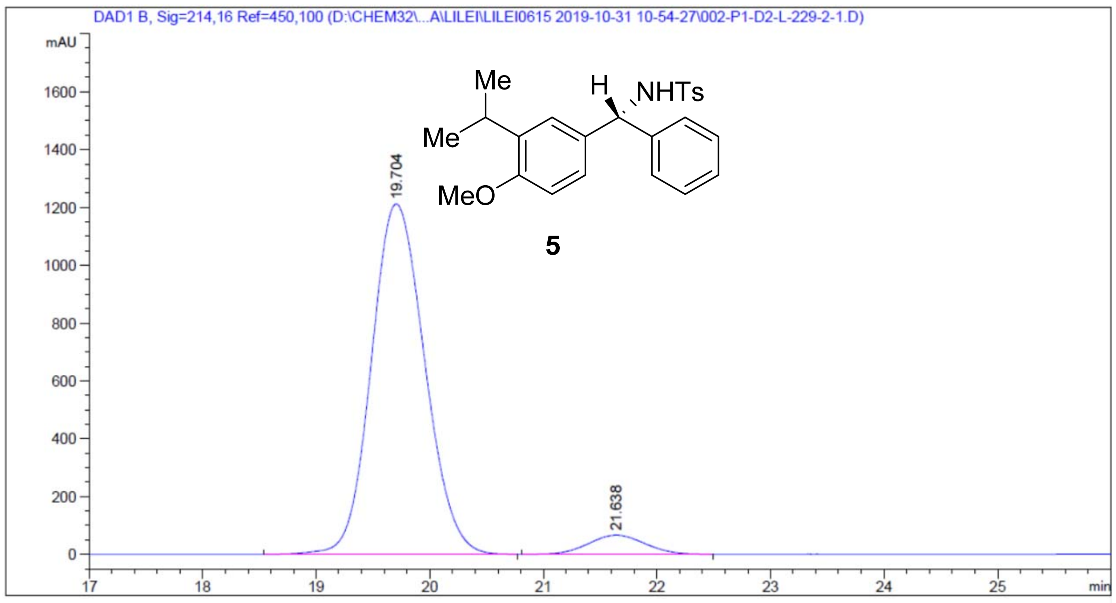

Peak #	RetTime [min]	Type	Width [min]	Area [mAU*s]	Height [mAU]	Area %
1	19.704	VV R	0.5015	3.95568e4	1211.23682	94.4442
2	21.638	VB R	0.4146	2326.99805	66.45001	5.5558

Supplementary Figure 105. HPLC spectra of compound *rac*-**5** and compound **5**

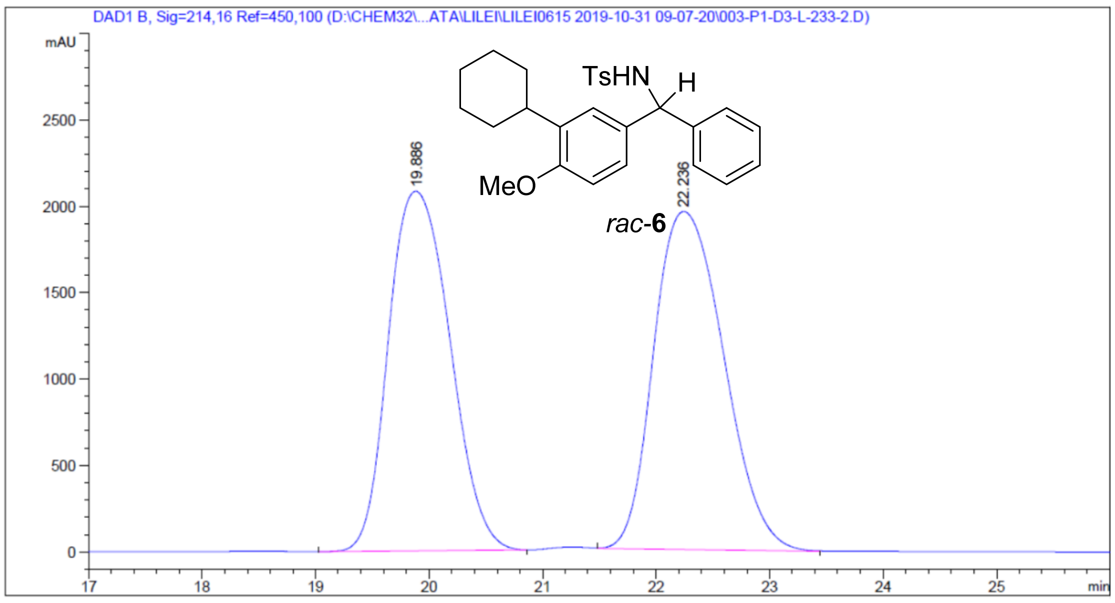

Peak #	RetTime [min]	Type	Width [min]	Area [mAU*s]	Height [mAU]	Area %
1	19.886	BB	0.4474	7.84962e4	2081.90918	48.2980
2	22.236	MM	0.7151	8.40287e4	1958.43469	51.7020

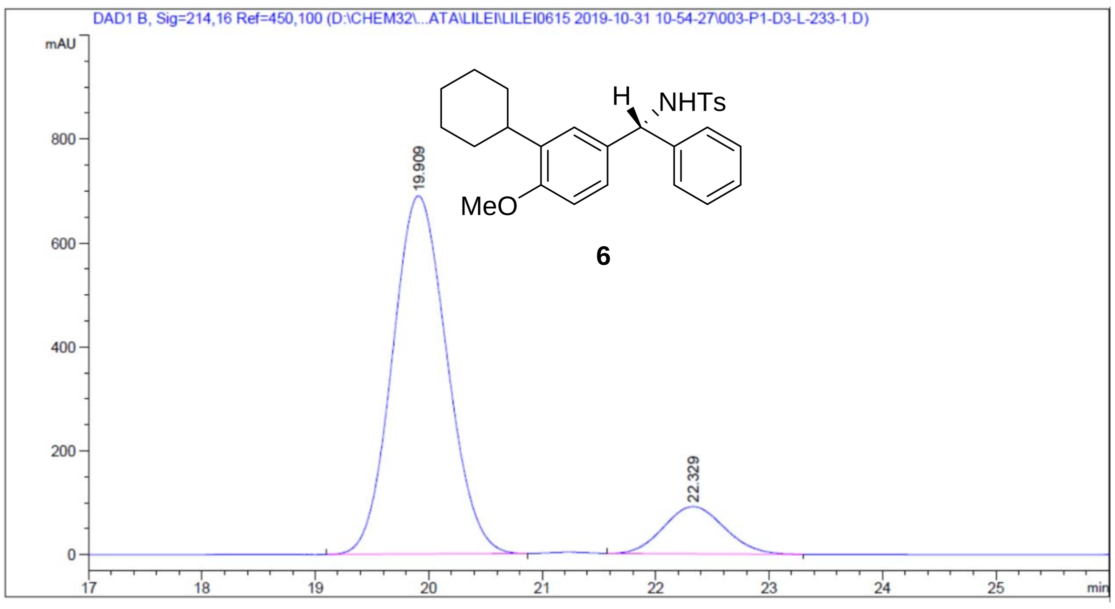

Peak #	RetTime [min]	Type	Width [min]	Area [mAU*s]	Height [mAU]	Area %
1	19.909	BB	0.5050	2.30296e4	689.76605	87.1261
2	22.329	BV R	0.4430	3402.89087	91.04781	12.8739

Supplementary Figure 106. HPLC spectra of compound *rac*-**6** and compound **6**

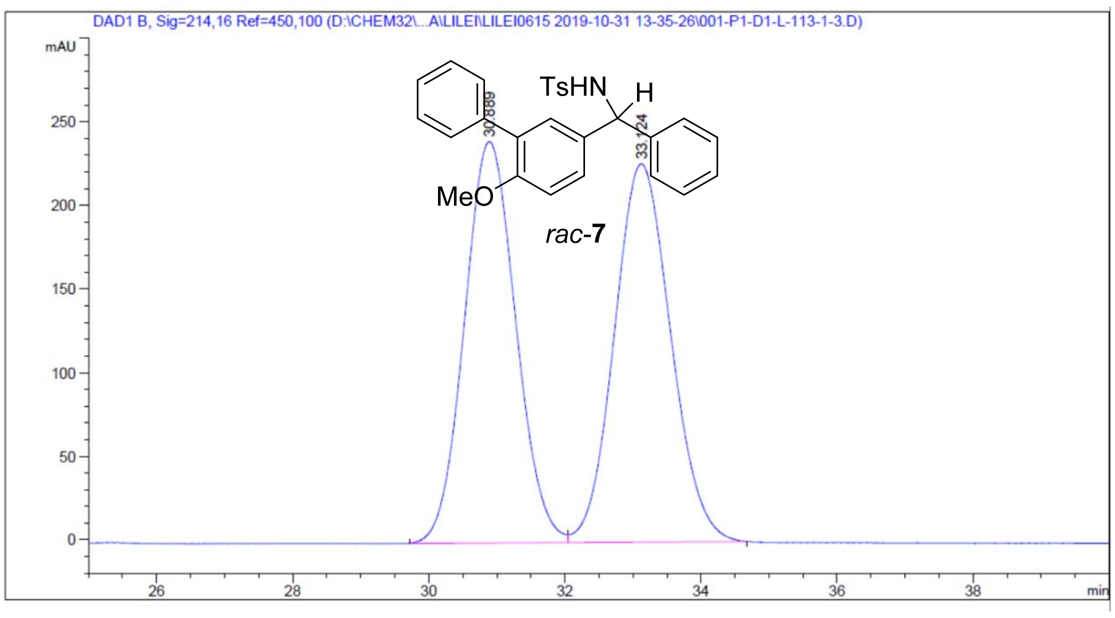

Peak #	RetTime [min]	Type	Width [min]	Area [mAU*s]	Height [mAU]	Area %
1	30.889	BV	0.6604	1.25645e4	239.96625	49.1064
2	33.124	VB	0.6935	1.30217e4	226.23187	50.8936

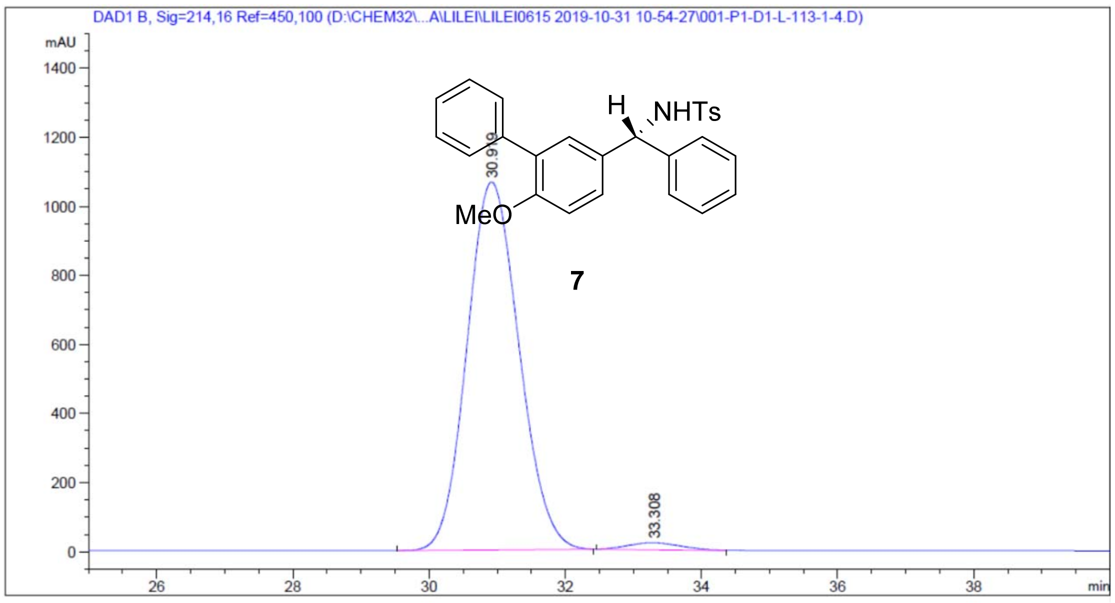

Peak #	RetTime [min]	Type	Width [min]	Area [mAU*s]	Height [mAU]	Area %
1	30.919	VV R	0.7675	5.66531e4	1065.16187	98.0878
2	33.308	MM	0.9016	1104.42407	20.41596	1.9122

Supplementary Figure 107. HPLC spectra of compound *rac*-**7** and compound **7**

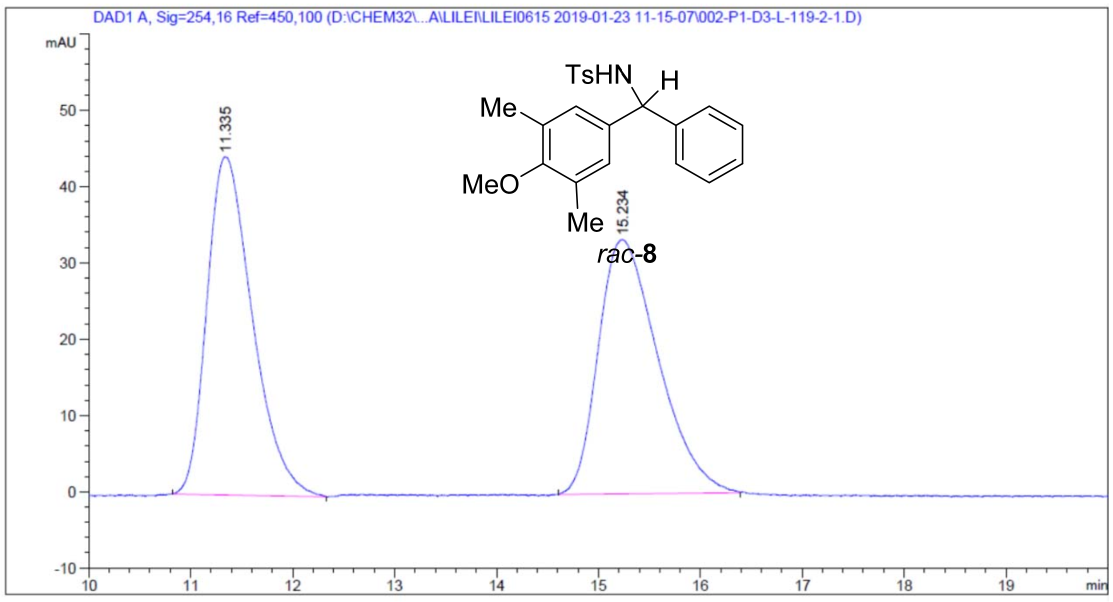

Peak #	RetTime [min]	Type	Width [min]	Area [mAU*s]	Height [mAU]	Area %
1	11.335	BB	0.3900	1377.39673	44.33589	50.3478
2	15.234	BB	0.4812	1358.36548	33.34821	49.6522

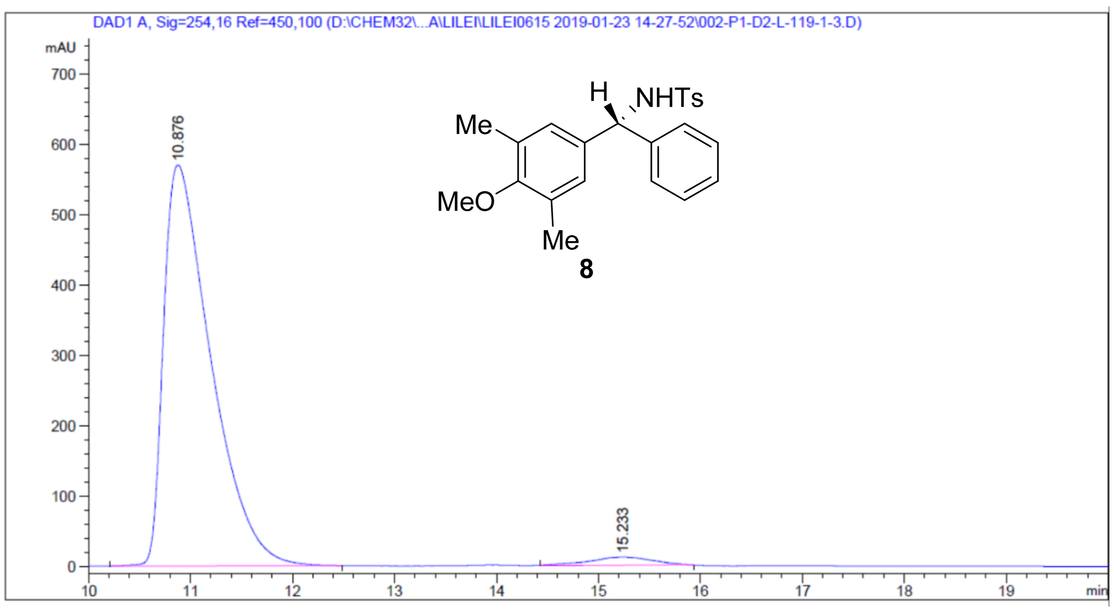

Peak #	RetTime [min]	Type	Width [min]	Area [mAU*s]	Height [mAU]	Area %
1	10.876	BV R	0.4792	1.83419e4	570.33624	97.3243
2	15.233	MM	0.7235	504.26987	11.61718	2.6757

Supplementary Figure 108. HPLC spectra of compound *rac*-**8** and compound **8**

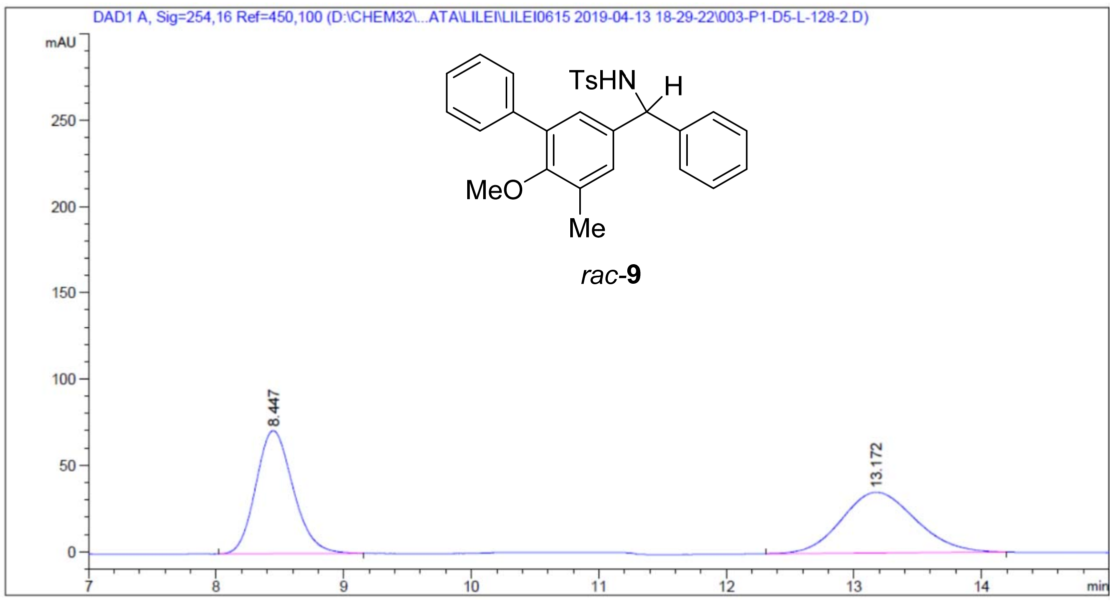

Peak #	RetTime [min]	Type	Width [min]	Area [mAU*s]	Height [mAU]	Area %
1	8.447	BB	0.3075	1452.06897	71.33645	50.4942
2	13.172	BB	0.4783	1423.64258	35.20686	49.5058

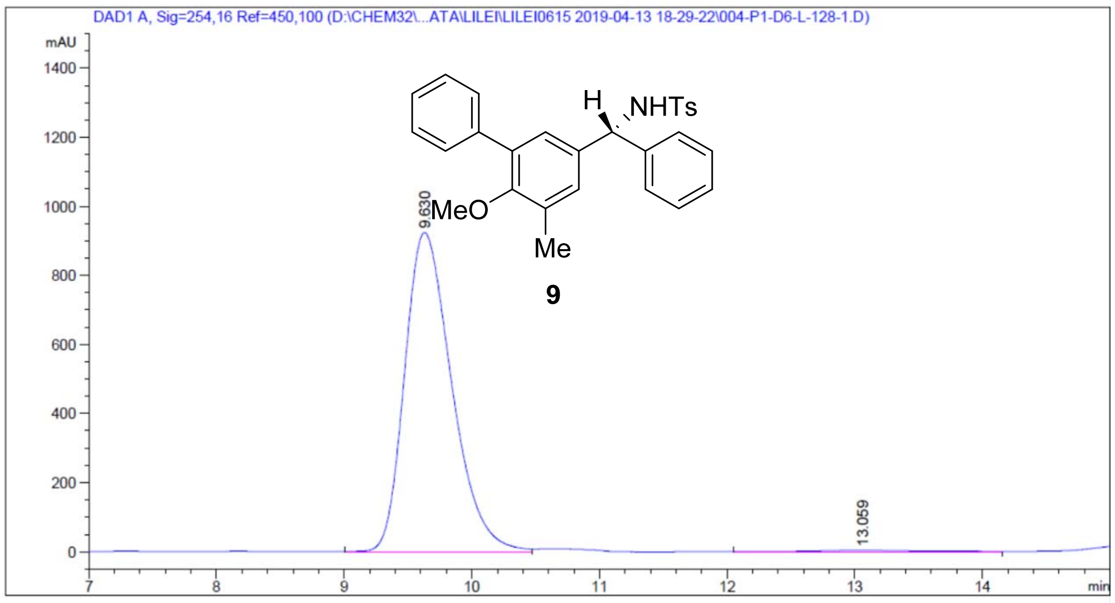

Peak #	RetTime [min]	Type	Width [min]	Area [mAU*s]	Height [mAU]	Area %
1	9.630	MM	0.4261	2.36139e4	923.66498	99.1212
2	13.059	MM	0.8545	209.36003	4.08362	0.8788

Supplementary Figure 109. HPLC spectra of compound *rac*-**9** and compound **9**

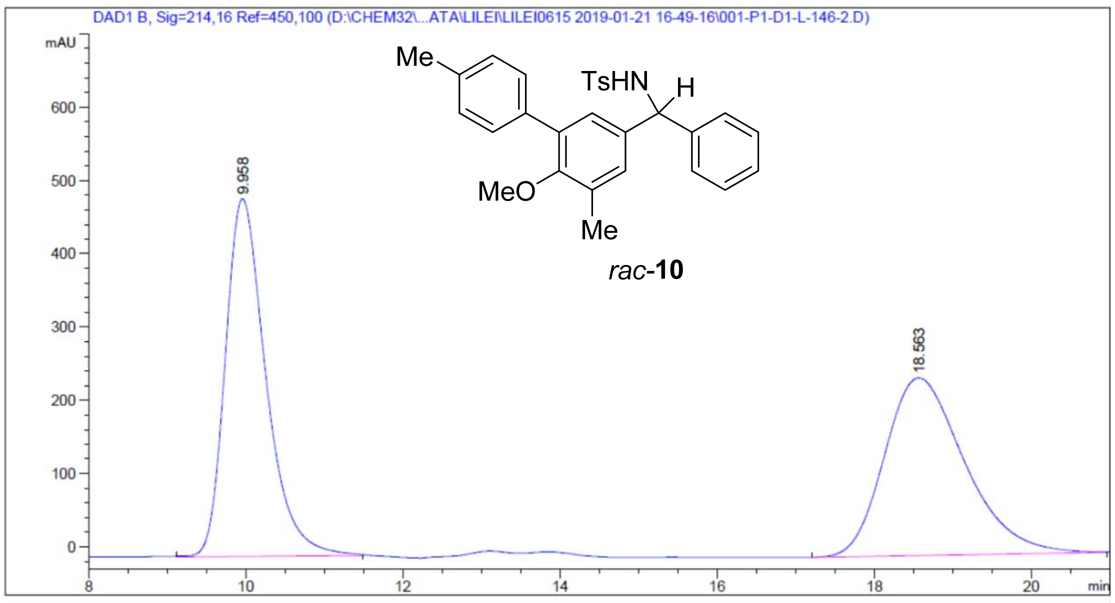

Peak #	RetTime [min]	Type	Width [min]	Area [mAU*s]	Height [mAU]	Area %
1	9.958	MM	0.5967	1.74766e4	488.18491	50.4064
2	18.563	BV R	0.8542	1.71948e4	242.65204	49.5936

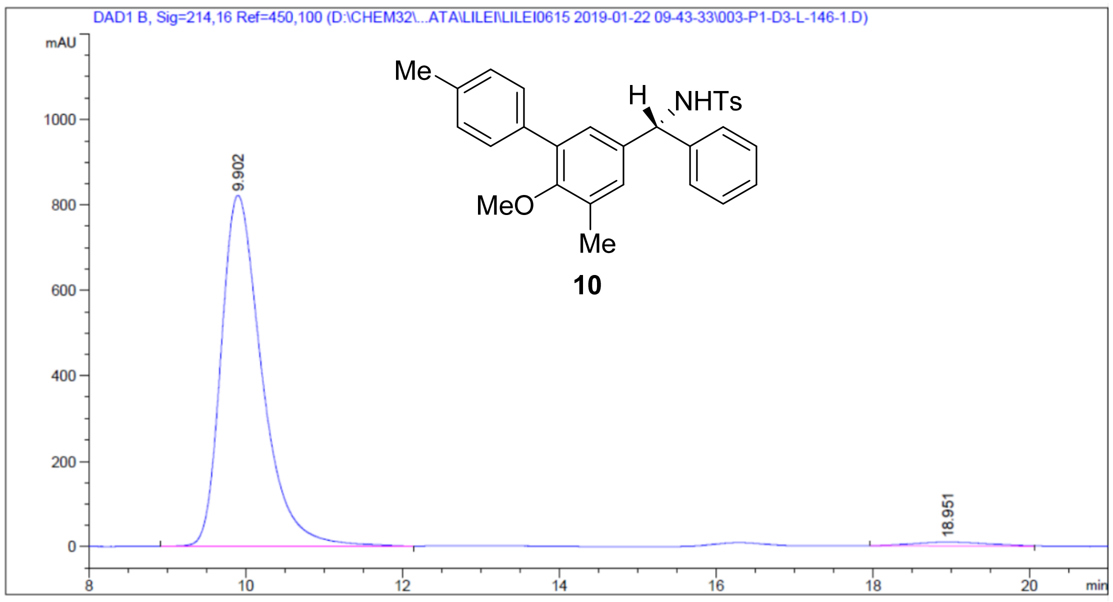

Peak #	RetTime [min]	Type	Width [min]	Area [mAU*s]	Height [mAU]	Area %
1	9.902	BB	0.5429	2.96410e4	821.65753	98.0102
2	18.951	MM	1.1025	601.76648	9.09682	1.9898

Supplementary Figure 110. HPLC spectra of compound *rac*-**10** and compound **10**

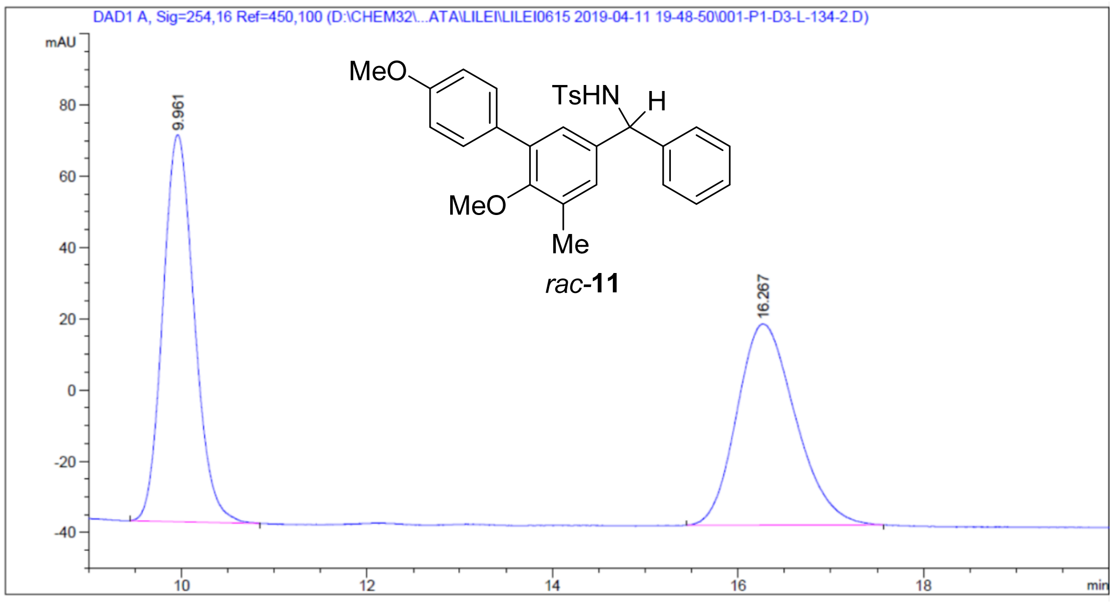

Peak #	RetTime [min]	Type	Width [min]	Area [mAU*s]	Height [mAU]	Area %
1	9.961	BB	0.3629	2558.73340	108.59732	50.8087
2	16.267	BB	0.5214	2477.28320	56.45848	49.1913

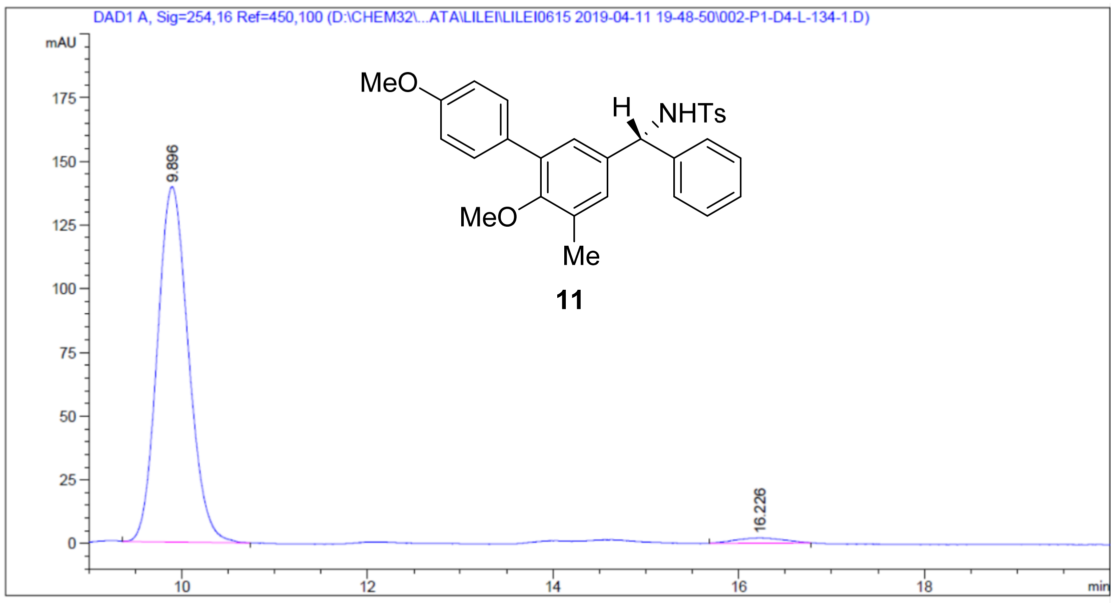

Peak #	RetTime [min]	Type	Width [min]	Area [mAU*s]	Height [mAU]	Area %
1	9.896	BB	0.3571	3240.20093	139.45175	97.8011
2	16.226	BB	0.4234	72.85006	2.02412	2.1989

Supplementary Figure 111. HPLC spectra of compound *rac*-**11** and compound **11**

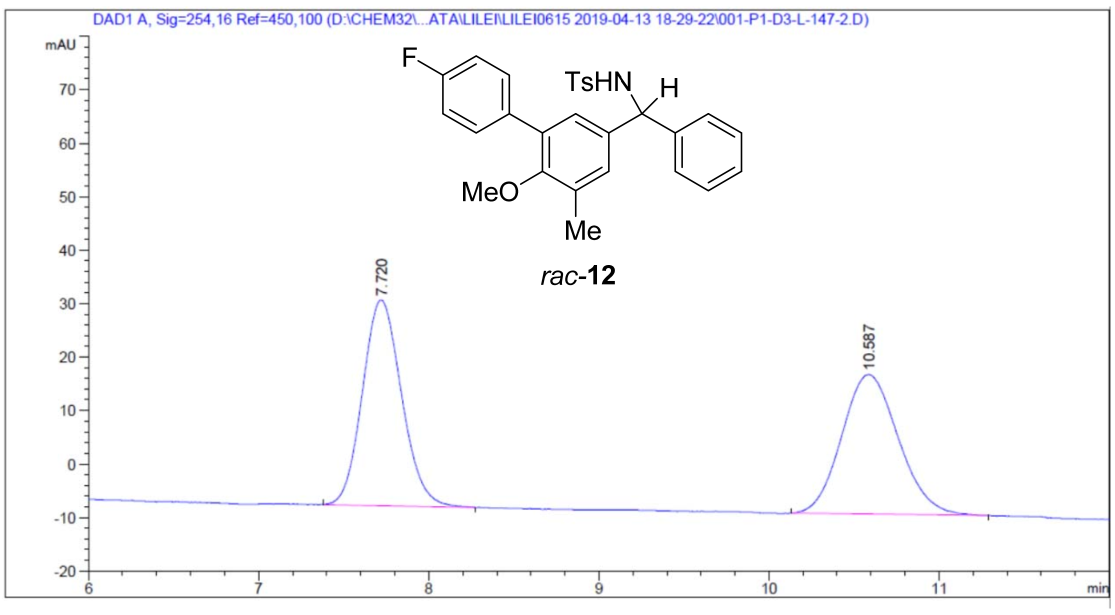

Peak #	RetTime [min]	Type	Width [min]	Area [mAU*s]	Height [mAU]	Area %
1	7.720	BB	0.2425	615.06122	38.47652	50.3914
2	10.587	BB	0.2990	605.50592	26.11326	49.6086

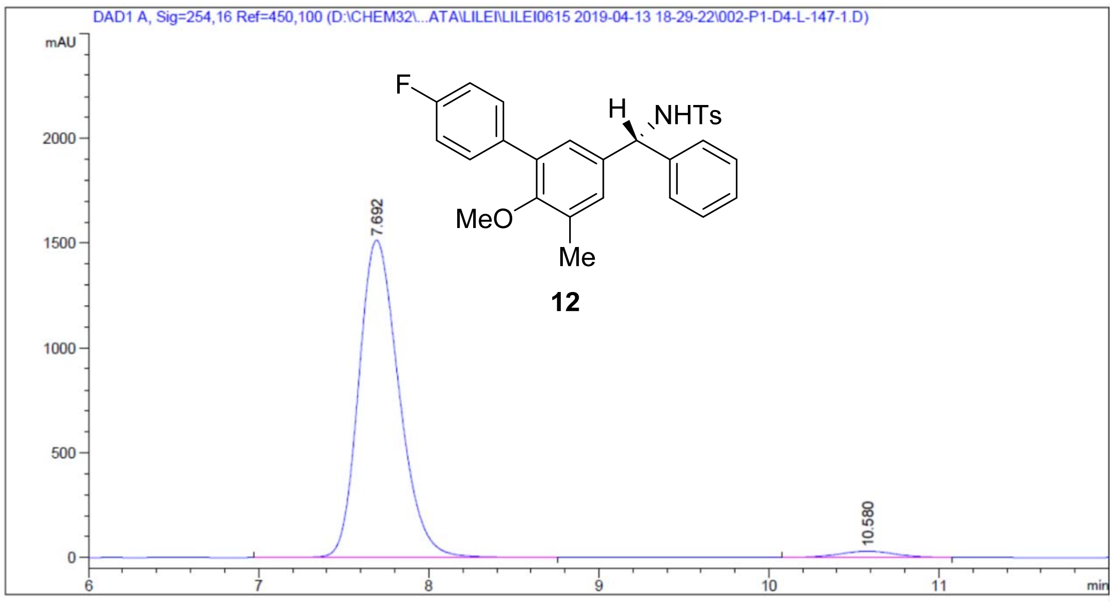

Peak #	RetTime [min]	Type	Width [min]	Area [mAU*s]	Height [mAU]	Area %
1	7.692	MM	0.2732	2.48047e4	1513.38208	97.4354
2	10.580	BB	0.3151	652.89471	29.39798	2.5646

Supplementary Figure 112. HPLC spectra of compound *rac*-**12** and compound **12**

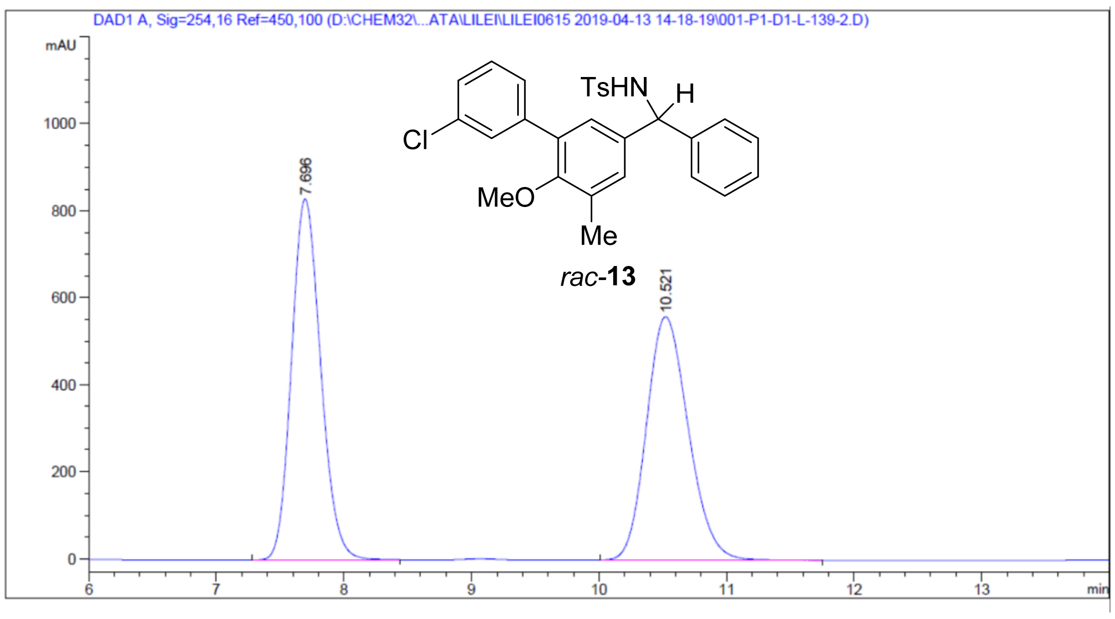

Peak #	RetTime [min]	Type	Width [min]	Area [mAU*s]	Height [mAU]	Area %
1	7.696	MM	0.2650	1.31939e4	829.83948	50.6881
2	10.521	MM	0.3818	1.28357e4	560.24402	49.3119

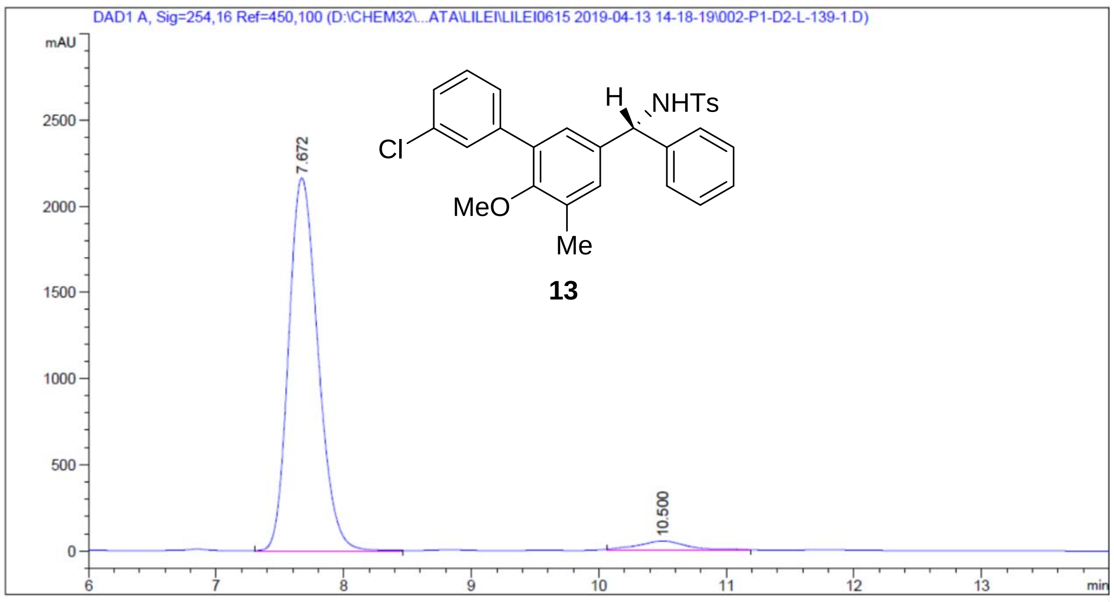

Peak #	RetTime [min]	Type	Width [min]	Area [mAU*s]	Height [mAU]	Area %
1	7.672	MM	0.2665	3.46088e4	2164.42310	96.4682
2	10.500	MM	0.4284	1267.06128	49.29372	3.5318

Supplementary Figure 113. HPLC spectra of compound *rac*-**13** and compound **13**

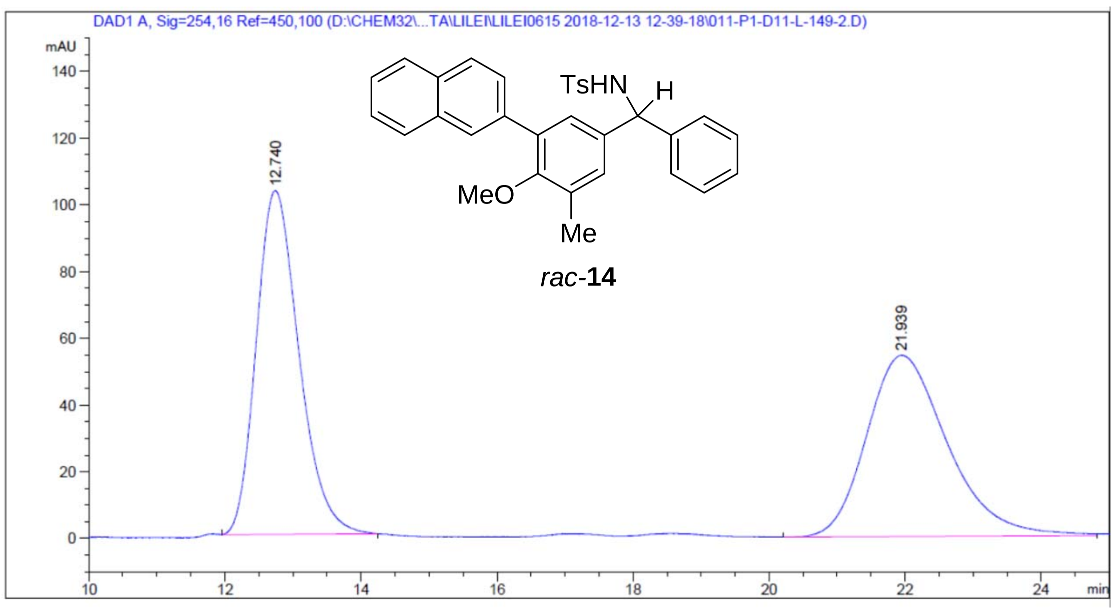

Peak #	RetTime [min]	Type	Width [min]	Area [mAU*s]	Height [mAU]	Area %
1	12.740	BB	0.5658	4494.22852	103.04344	50.1478
2	21.939	MM	1.3724	4467.72949	54.25827	49.8522

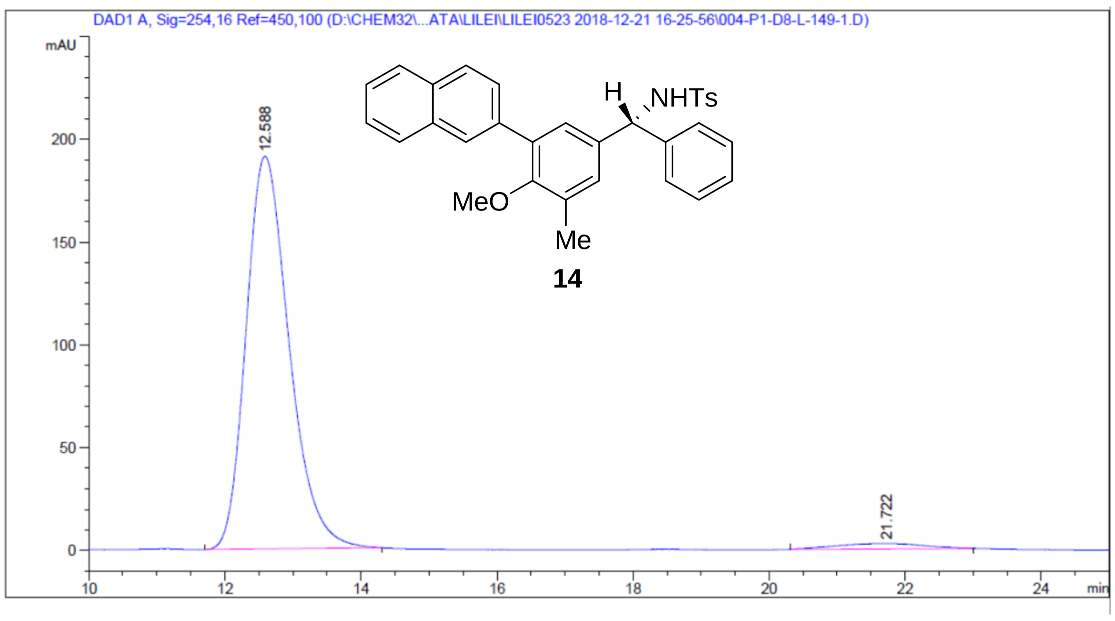

Peak #	RetTime [min]	Type	Width [min]	Area [mAU*s]	Height [mAU]	Area %
1	12.588	BV R	0.6006	8404.38672	191.08705	97.1301
2	21.722	MM	1.5065	248.32370	2.74731	2.8699

Supplementary Figure 114. HPLC spectra of compound *rac*-**14** and compound **14**

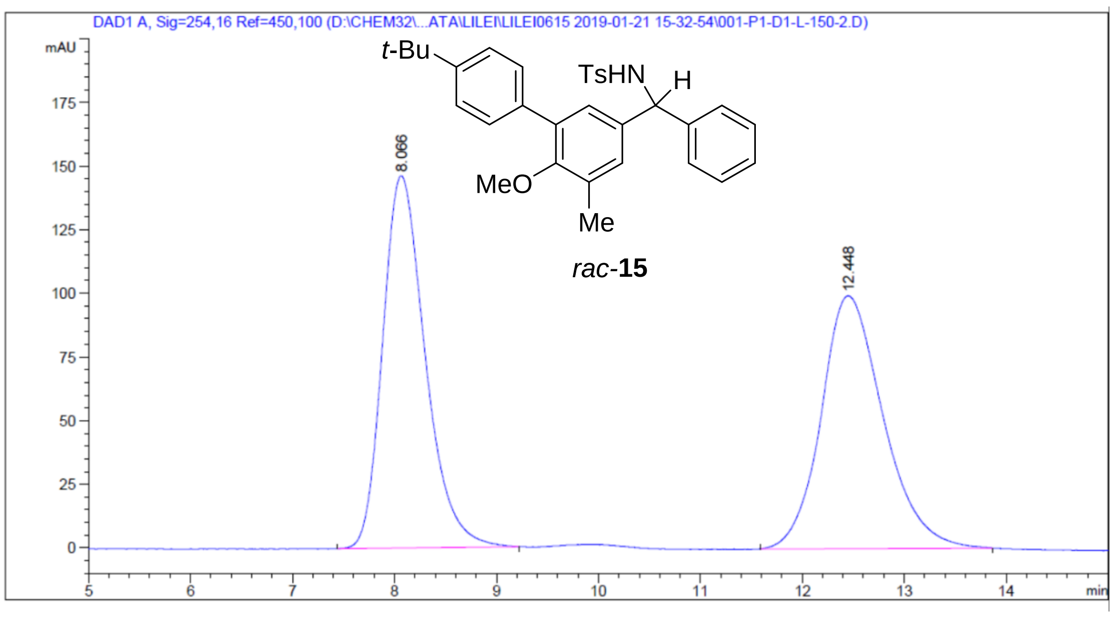

Peak #	RetTime [min]	Type	Width [min]	Area [mAU*s]	Height [mAU]	Area %
1	8.066	BB	0.4359	4236.43848	146.26231	50.2283
2	12.448	BB	0.5213	4197.93066	99.29034	49.7717

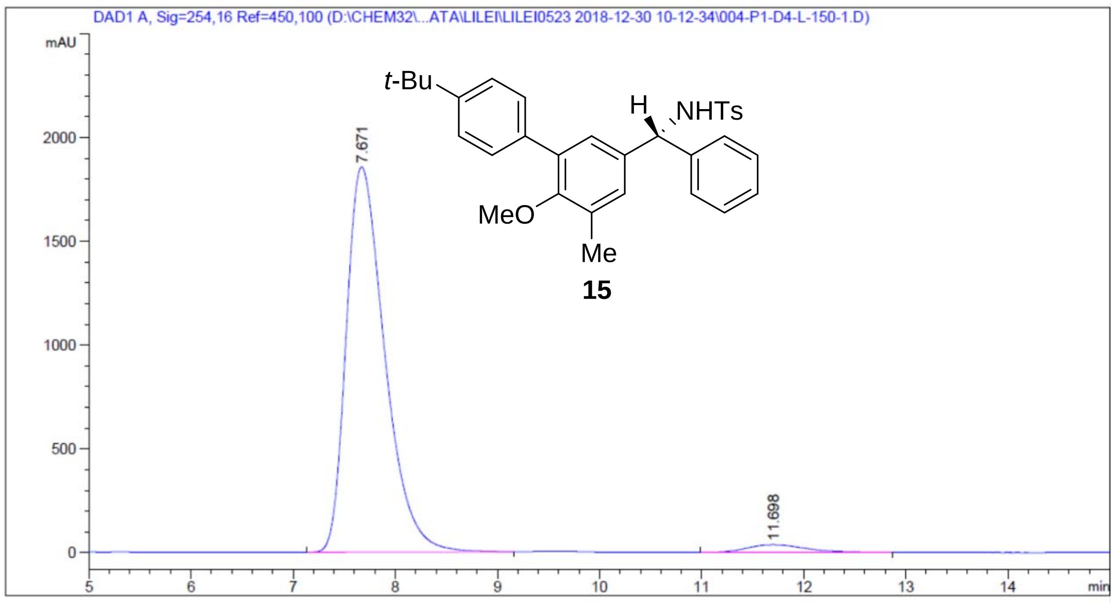

Peak #	RetTime [min]	Type	Width [min]	Area [mAU*s]	Height [mAU]	Area %
1	7.671	BB	0.4003	4.86644e4	1856.57703	97.0594
2	11.698	BB	0.4877	1474.40356	37.41671	2.9406

Supplementary Figure 115. HPLC spectra of compound *rac*-**15** and compound **15**

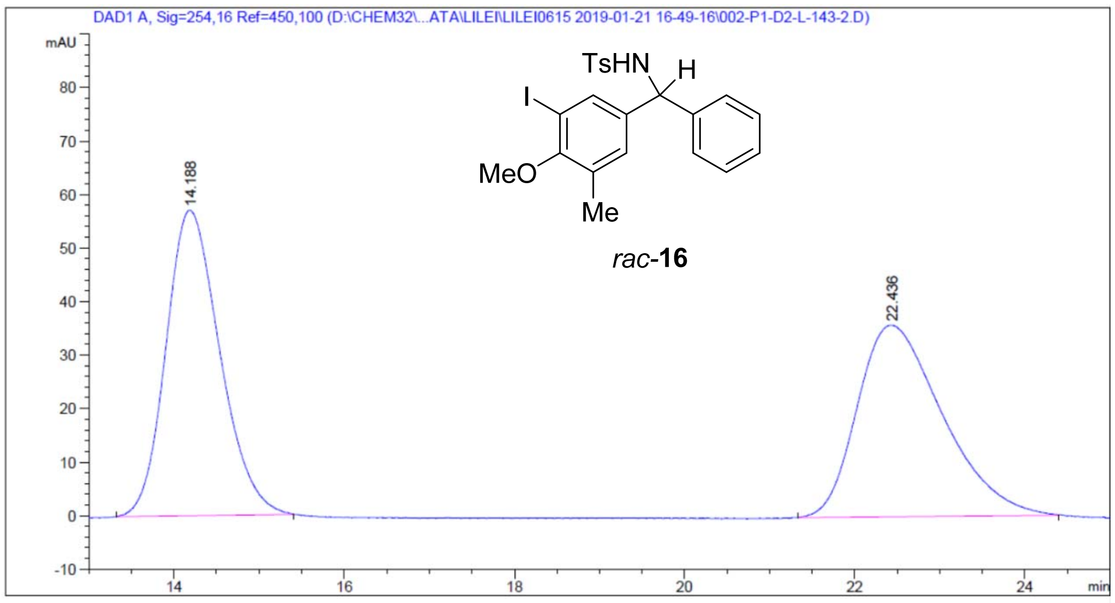

Peak #	RetTime [min]	Type	Width [min]	Area [mAU*s]	Height [mAU]	Area %
1	14.188	BB	0.5214	2509.41260	57.08345	50.1664
2	22.436	BB	0.8195	2492.76636	35.81755	49.8336

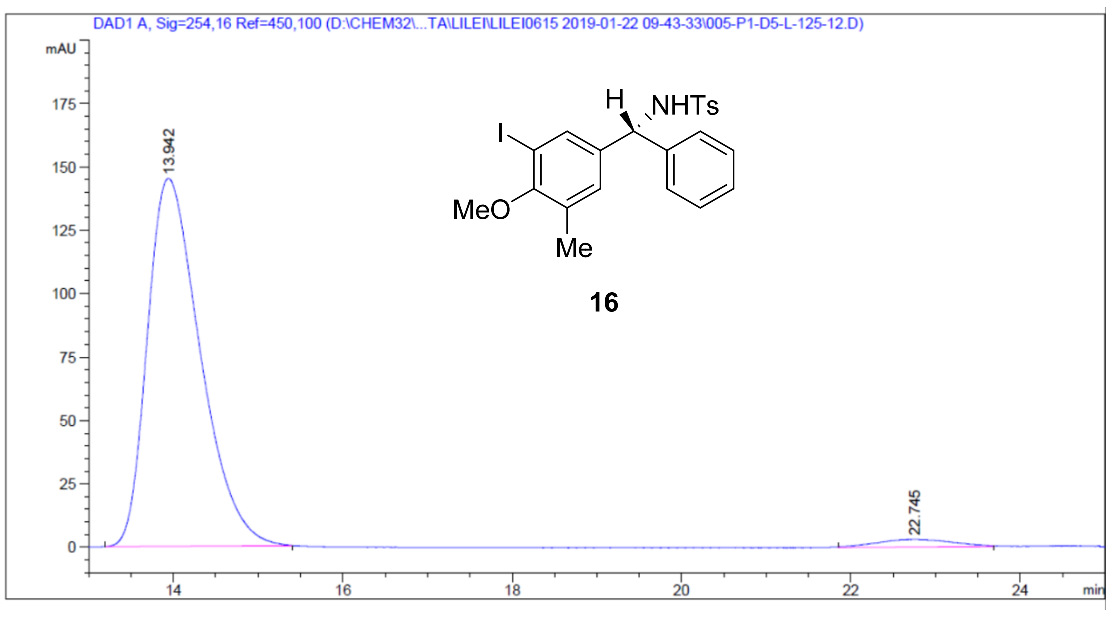

Peak #	RetTime [min]	Type	Width [min]	Area [mAU*s]	Height [mAU]	Area %
1	13.942	BB	0.5922	6283.70947	145.11275	97.1229
2	22.745	MM	1.0344	186.14610	2.99939	2.8771

Supplementary Figure 116. HPLC spectra of compound *rac*-**16** and compound **16**

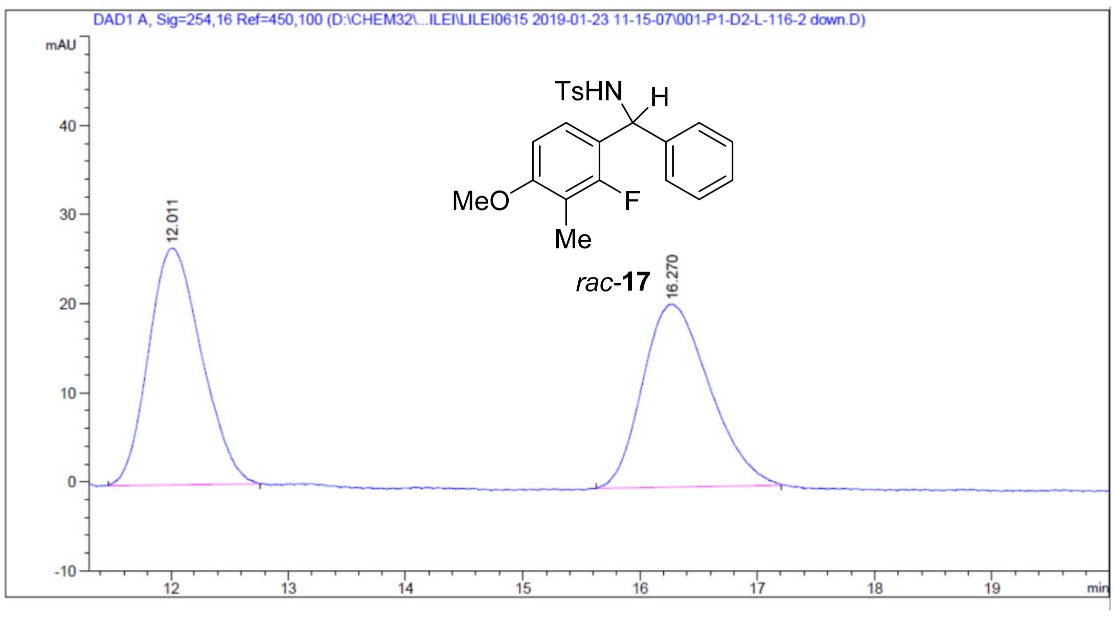

Peak #	RetTime [min]	Type	Width [min]	Area [mAU*s]	Height [mAU]	Area %
1	12.011	BB	0.3710	835.13715	26.62425	50.5823
2	16.270	BB	0.4679	815.90814	20.56261	49.4177

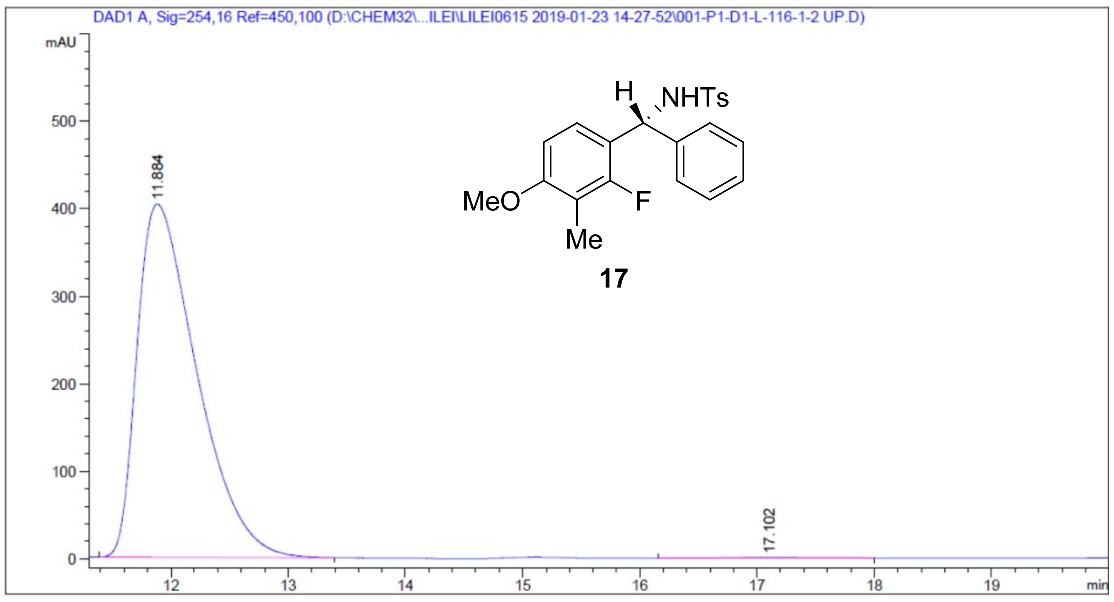

Peak #	RetTime [min]	Type	Width [min]	Area [mAU*s]	Height [mAU]	Area %
1	11.884	BB	0.5074	1.42594e4	403.35883	99.5154
2	17.102	MM	0.8553	69.43400	1.35295	0.4846

Supplementary Figure 117. HPLC spectra of compound *rac*-**17** and compound **17**

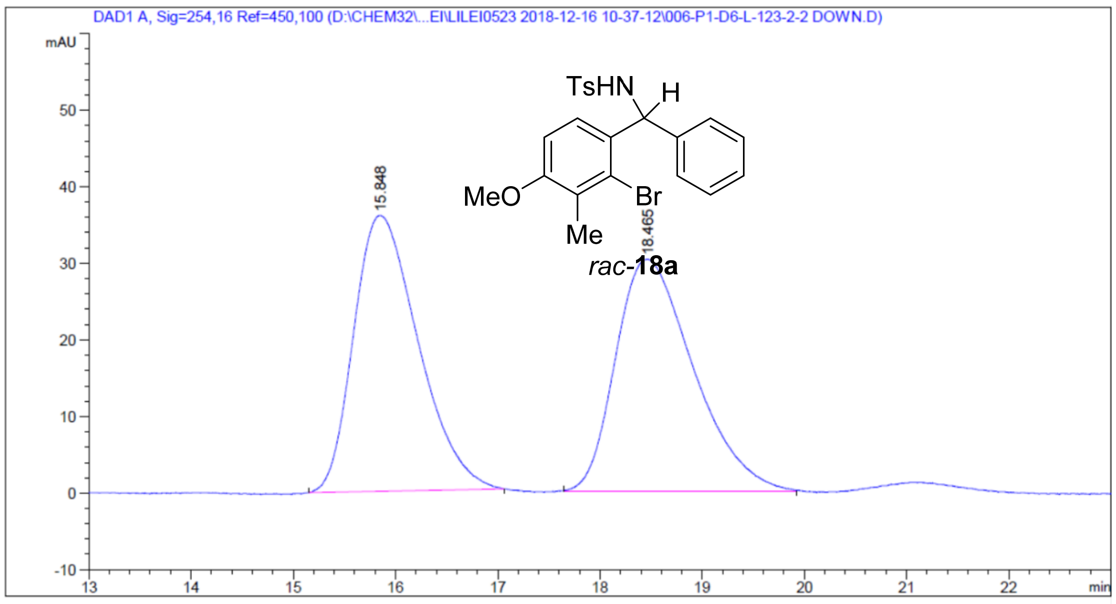

Peak #	RetTime [min]	Type	Width [min]	Area [mAU*s]	Height [mAU]	Area %
1	15.848	BB	0.5189	1582.75146	36.03997	49.8840
2	18.465	BB	0.6227	1590.11108	30.25485	50.1160

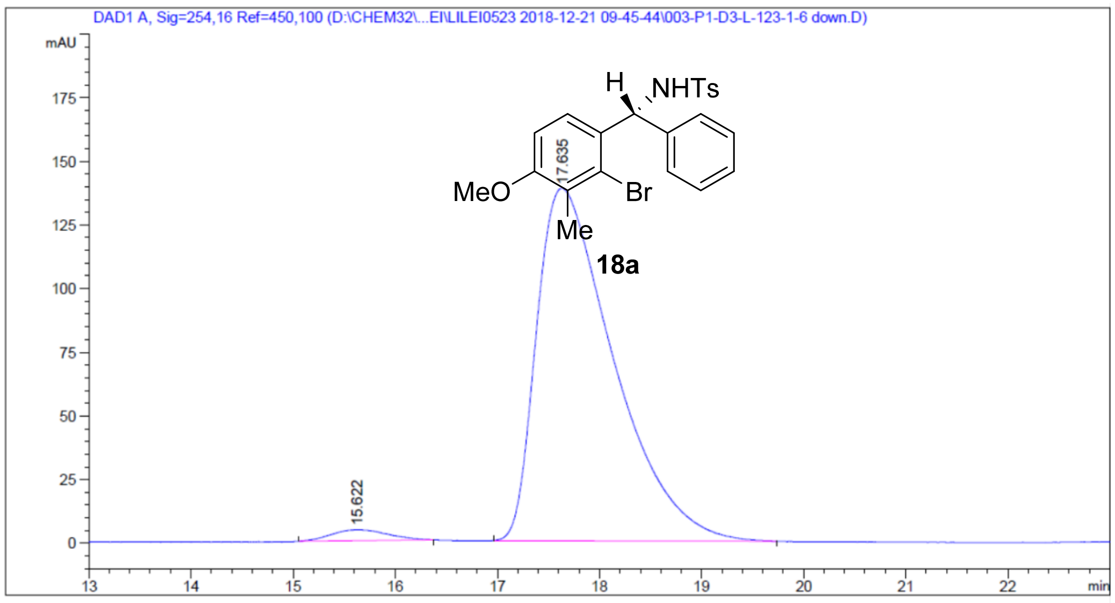

Peak #	RetTime [min]	Type	Width [min]	Area [mAU*s]	Height [mAU]	Area %
1	15.622	BB	0.4483	166.56355	4.36839	2.2418
2	17.635	BB	0.6990	7263.18018	138.45119	97.7582

Supplementary Figure 118. HPLC spectra of compound *rac*-**18a** and compound **18a**

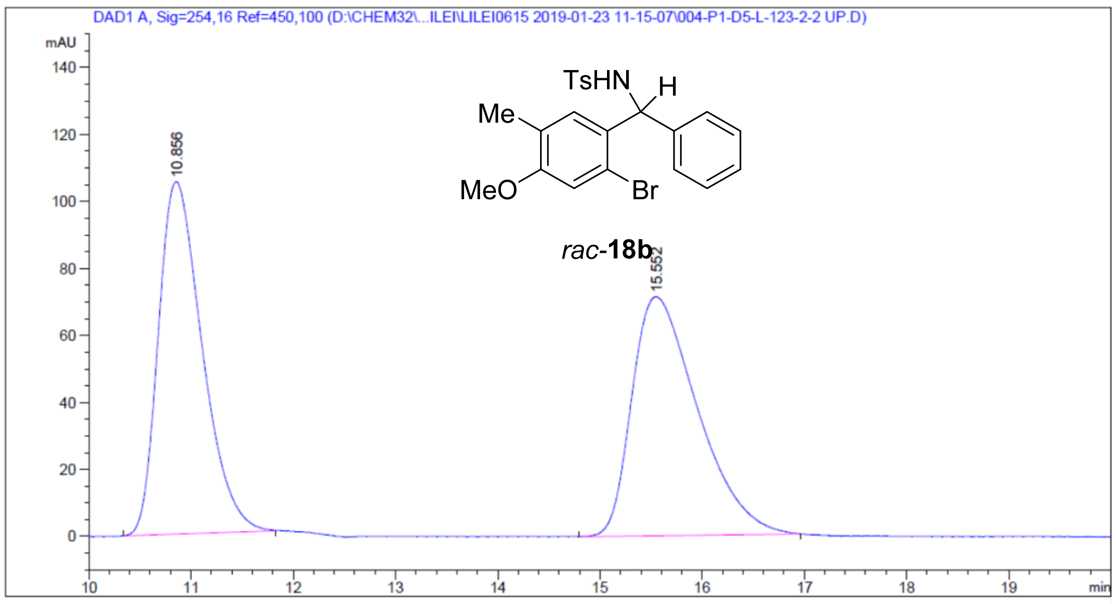

Peak #	RetTime [min]	Type	Width [min]	Area [mAU*s]	Height [mAU]	Area %
1	10.856	BB	0.4048	3128.36206	105.25044	49.5002
2	15.552	VB R	0.5494	3191.53613	71.33803	50.4998

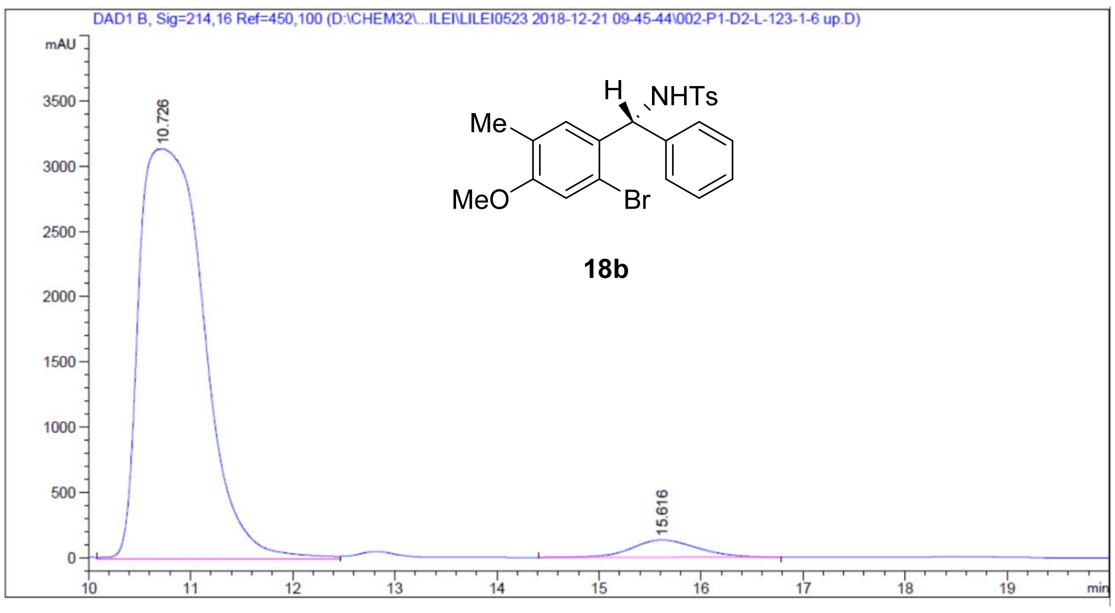

Peak #	RetTime [min]	Type	Width [min]	Area [mAU*s]	Height [mAU]	Area %
1	10.726	MM	0.7377	1.39258e5	3146.20093	95.9232
2	15.616	VB R	0.5508	5918.55713	132.20477	4.0768

Supplementary Figure 119. HPLC spectra of compound *rac*-**18b** and compound **18b**

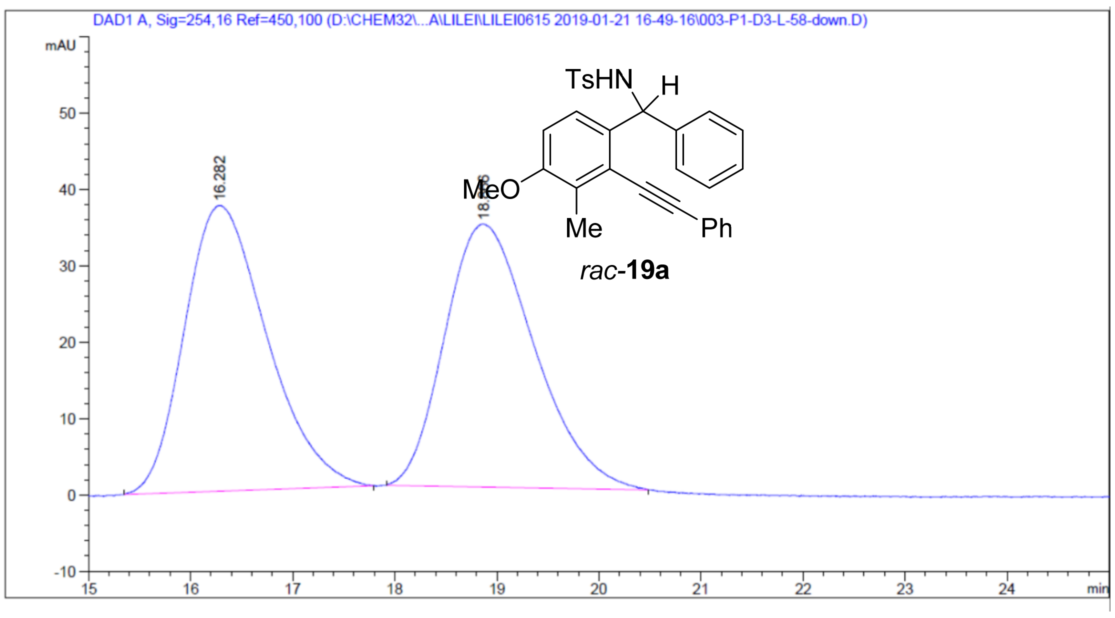

Peak #	RetTime [min]	Type	Width [min]	Area [mAU*s]	Height [mAU]	Area %
1	16.282	BB	0.6619	2101.54272	37.40760	50.0484
2	18.866	BB	0.7150	2097.47778	34.43993	49.9516

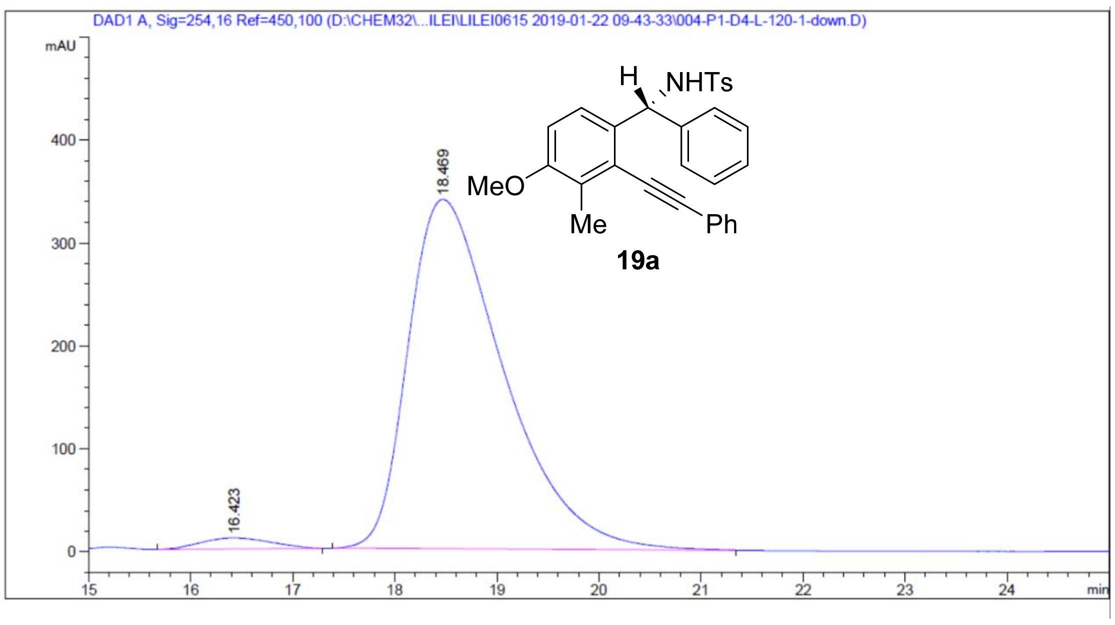

Peak #	RetTime [min]	Type	Width [min]	Area [mAU*s]	Height [mAU]	Area %
1	16.423	BB	0.5619	510.78409	10.70057	2.2643
2	18.469	BB	0.8456	2.20474e4	339.53979	97.7357

Supplementary Figure 120. HPLC spectra of compound *rac*-**19a** and compound **19a**

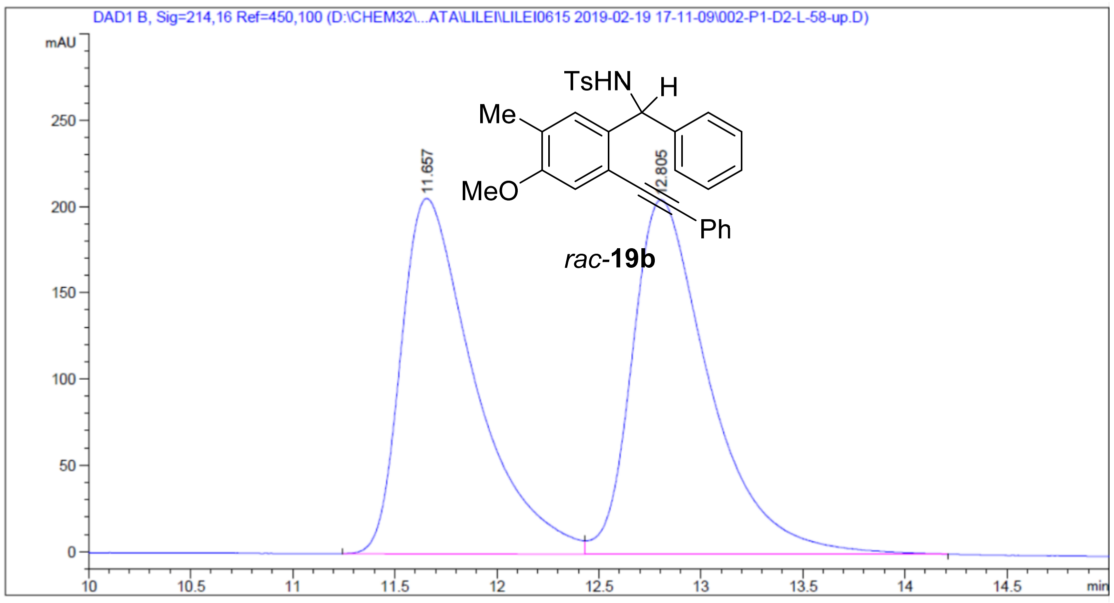

Peak #	RetTime [min]	Type	Width [min]	Area [mAU*s]	Height [mAU]	Area %
1	11.657	BV	0.3668	5180.46533	206.03651	48.8544
2	12.805	VV R	0.3850	5423.43066	205.41502	51.1456

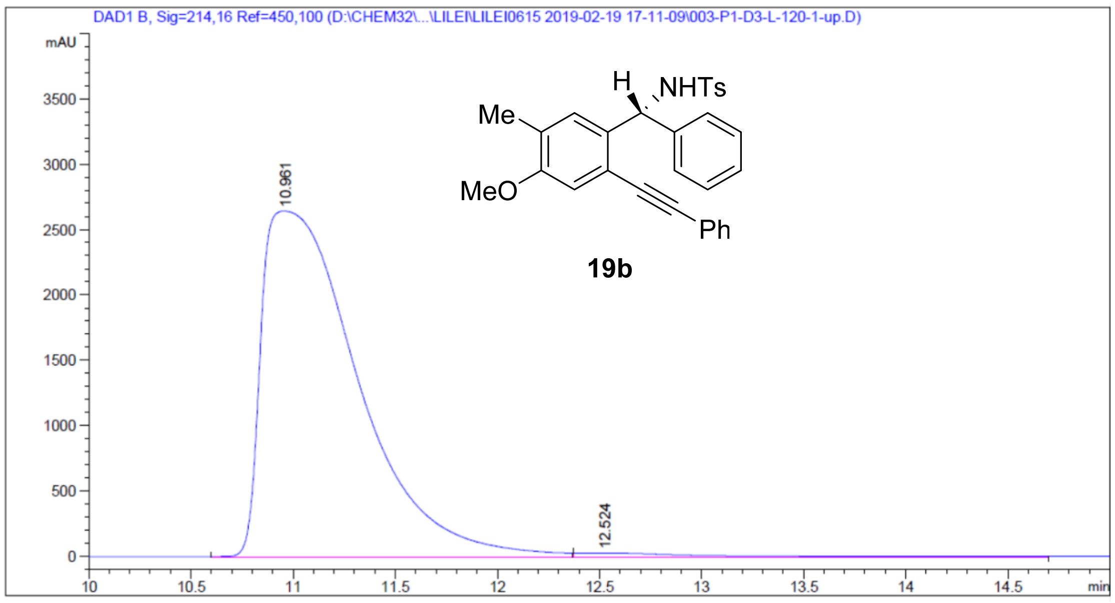

Peak #	RetTime [min]	Type	Width [min]	Area [mAU*s]	Height [mAU]	Area %
1	10.961	MM	0.5473	8.69229e4	2646.84668	98.6552
2	12.524	MM	0.6942	1184.85596	28.44611	1.3448

Supplementary Figure 121. HPLC spectra of compound *rac*-**19b** and compound **19b**

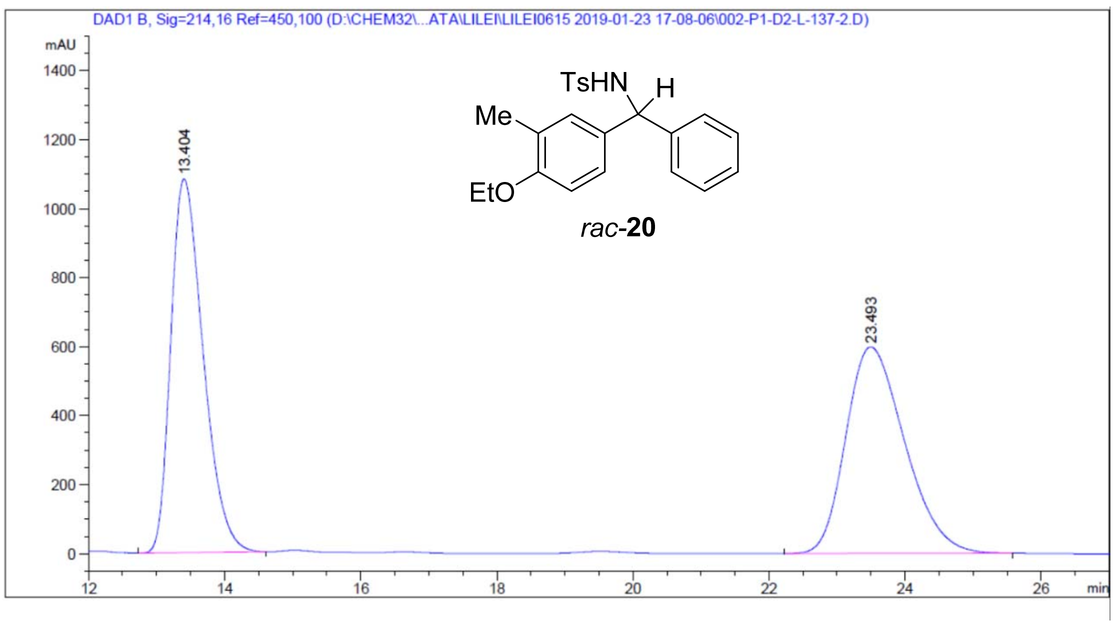

Peak #	RetTime [min]	Type	Width [min]	Area [mAU*s]	Height [mAU]	Area %
1	13.404	BB	0.5025	3.58044e4	1083.66882	49.8463
2	23.493	BV R	0.7609	3.60252e4	598.80939	50.1537

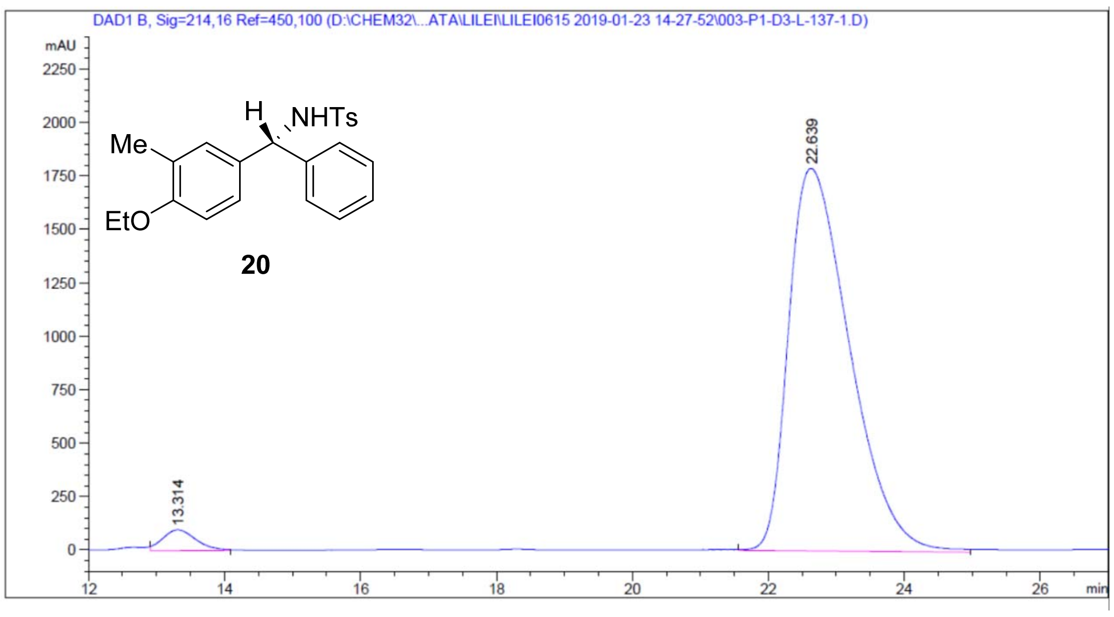

Peak #	RetTime [min]	Type	Width [min]	Area [mAU*s]	Height [mAU]	Area %
1	13.314	MM	0.5626	3274.87891	97.01439	2.8485
2	22.639	MM	1.0397	1.11692e5	1790.39282	97.1515

Supplementary Figure 122. HPLC spectra of compound *rac*-**20** and compound **20**

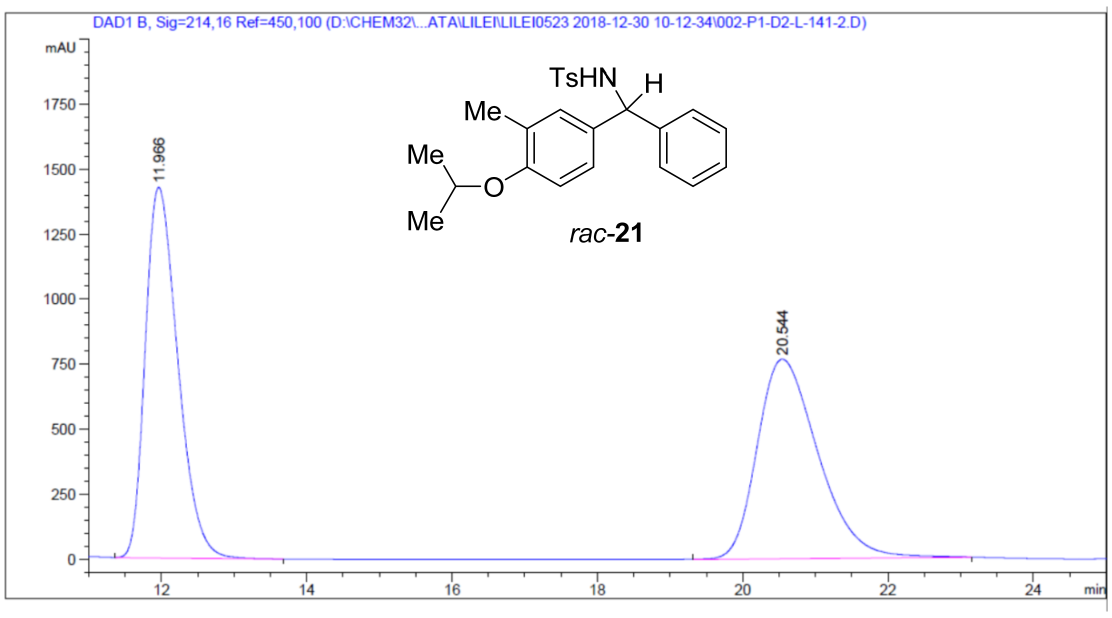

Peak #	RetTime [min]	Type	Width [min]	Area [mAU*s]	Height [mAU]	Area %
1	11.966	BB	0.4564	4.43211e4	1424.89697	49.7466
2	20.544	MM	0.9724	4.47727e4	767.37244	50.2534

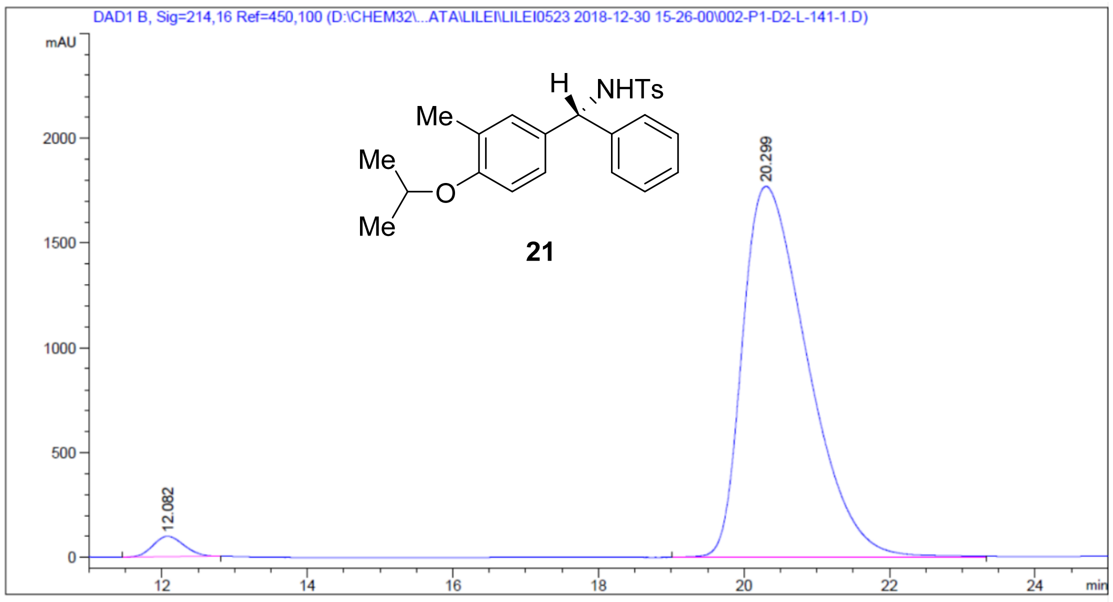

Peak #	RetTime [min]	Type	Width [min]	Area [mAU*s]	Height [mAU]	Area %
1	12.082	BB	0.3950	2896.60693	97.68357	2.5691
2	20.299	MM	1.0344	1.09850e5	1769.96301	97.4309

Supplementary Figure 123. HPLC spectra of compound *rac*-**21** and compound **21**

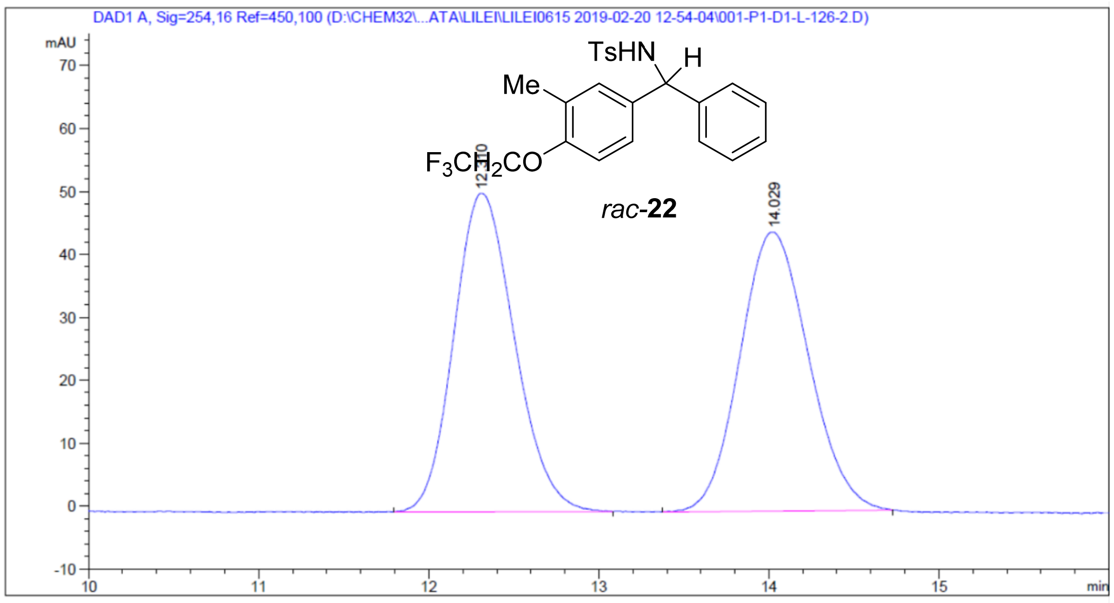

Peak #	RetTime [min]	Type	Width [min]	Area [mAU*s]	Height [mAU]	Area %
1	12.310	BV R	0.3062	1254.80713	50.62564	50.1600
2	14.029	BB	0.3434	1246.80420	44.30030	49.8400

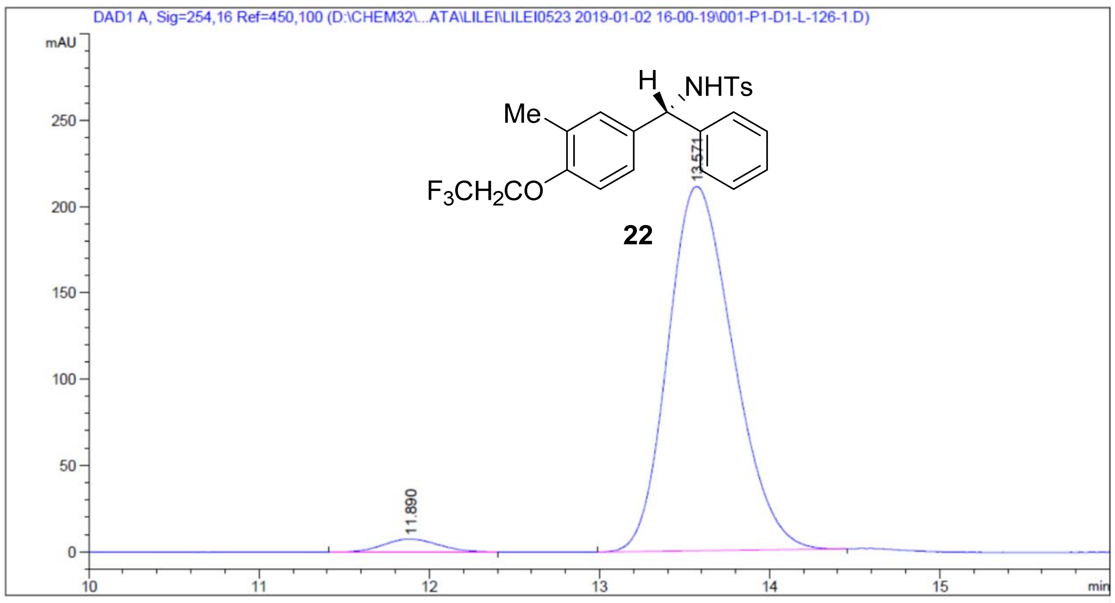

Peak #	RetTime [min]	Type	Width [min]	Area [mAU*s]	Height [mAU]	Area %
1	11.890	VV R	0.2697	172.83395	7.61901	2.9787
2	13.571	BV R	0.4122	5629.52783	211.06787	97.0213

Supplementary Figure 124. HPLC spectra of compound *rac*-**22** and compound **22**

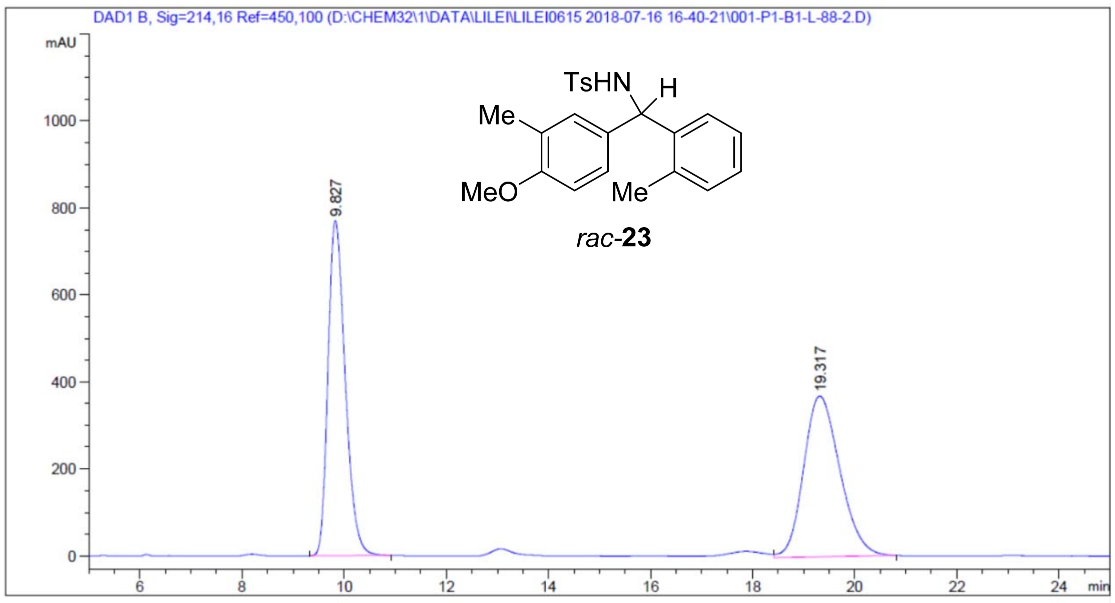

Peak #	RetTime [min]	Type	Width [min]	Area [mAU*s]	Height [mAU]	Area %
1	9.827	BB	0.3590	1.80009e4	770.77020	49.7162
2	19.317	MM	0.8209	1.82064e4	369.65085	50.2838

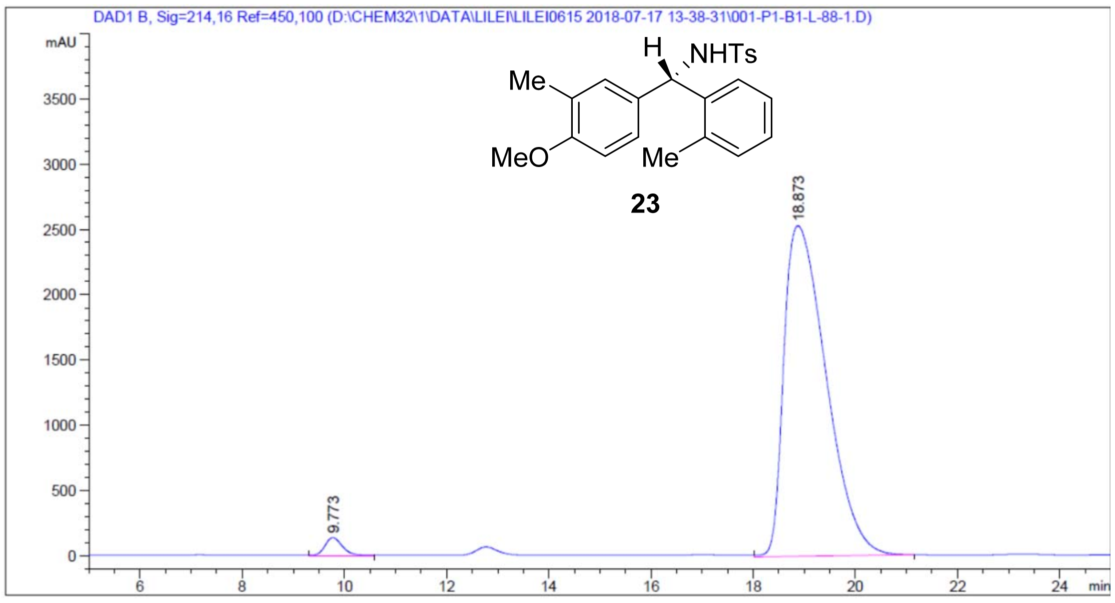

Peak #	RetTime [min]	Type	Width [min]	Area [mAU*s]	Height [mAU]	Area %
1	9.773	MM	0.3932	3235.79272	137.16701	2.1763
2	18.873	MM	0.9565	1.45447e5	2534.32397	97.8237

Supplementary Figure 125. HPLC spectra of compound *rac*-**23** and compound **23**

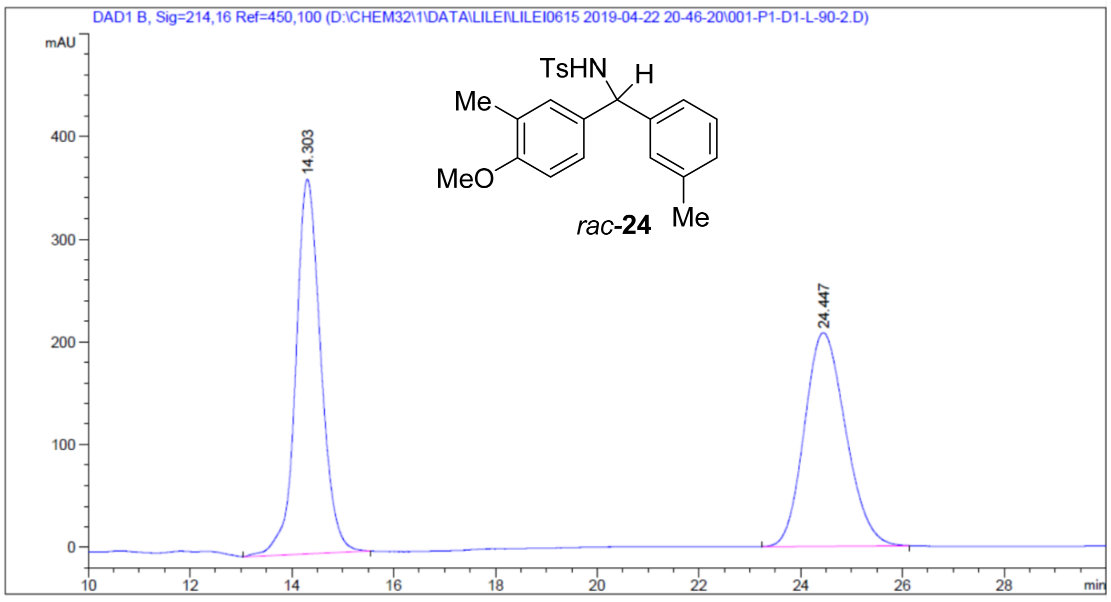

Peak #	RetTime [min]	Type	Width [min]	Area [mAU*s]	Height [mAU]	Area %
1	14.303	BV R	0.5133	1.24880e4	364.87027	51.5783
2	24.447	BV R	0.6799	1.17237e4	208.05458	48.4217

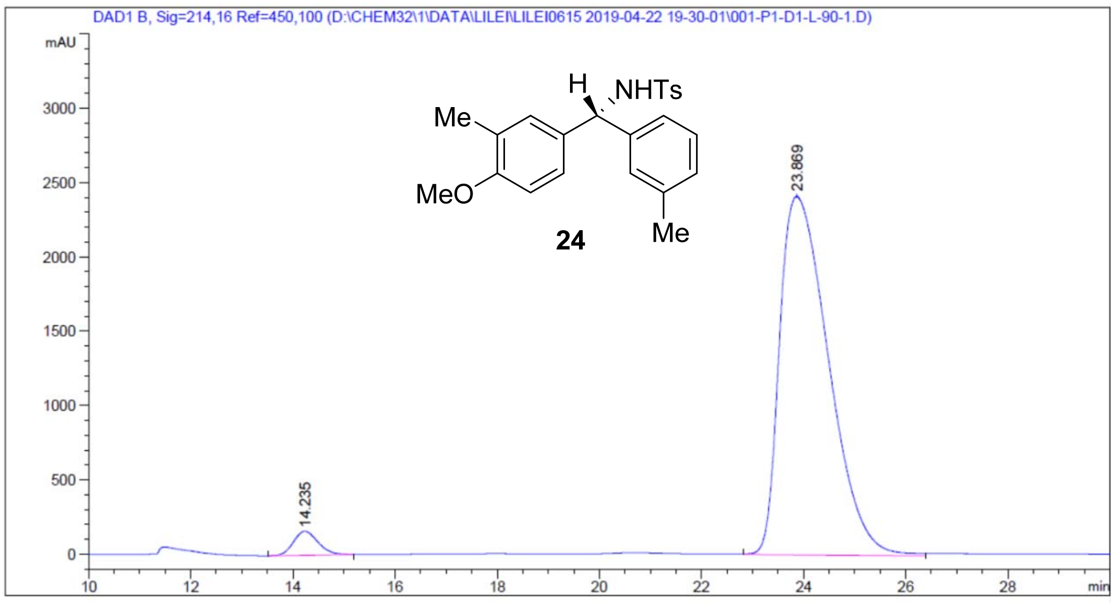

Peak #	RetTime [min]	Type	Width [min]	Area [mAU*s]	Height [mAU]	Area %
1	14.235	BB	0.4879	5345.71484	160.15608	3.2353
2	23.869	MM	1.1008	1.59885e5	2420.70020	96.7647

Supplementary Figure 126. HPLC spectra of compound *rac*-**24** and compound **24**

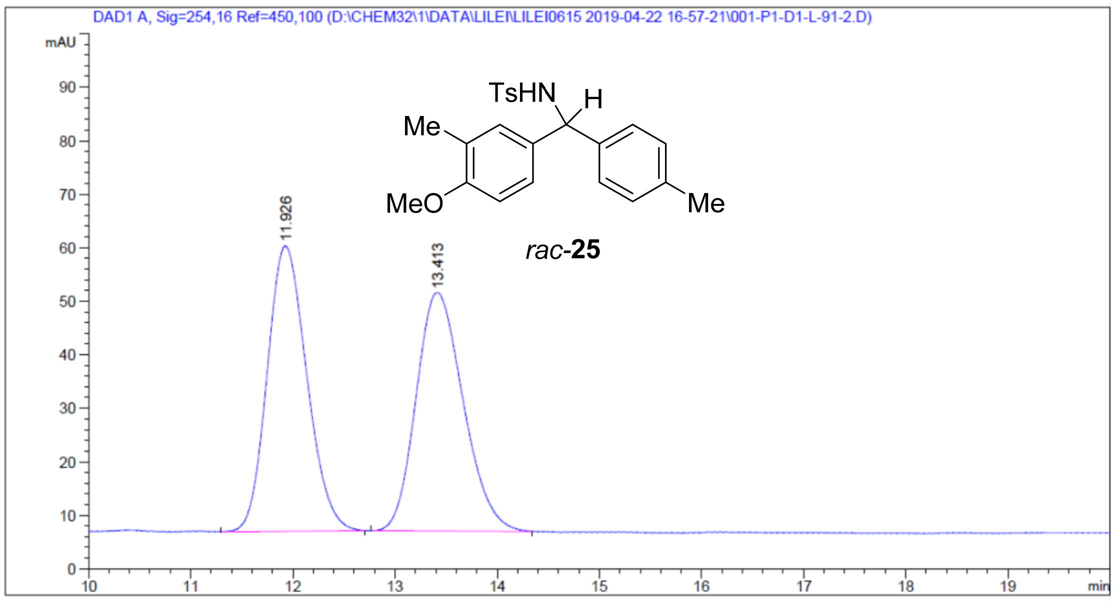

Peak #	RetTime [min]	Type	Width [min]	Area [mAU*s]	Height [mAU]	Area %
1	11.926	VB R	0.3715	1437.47070	53.32854	50.1163
2	13.413	BB	0.3897	1430.79919	44.60509	49.8837

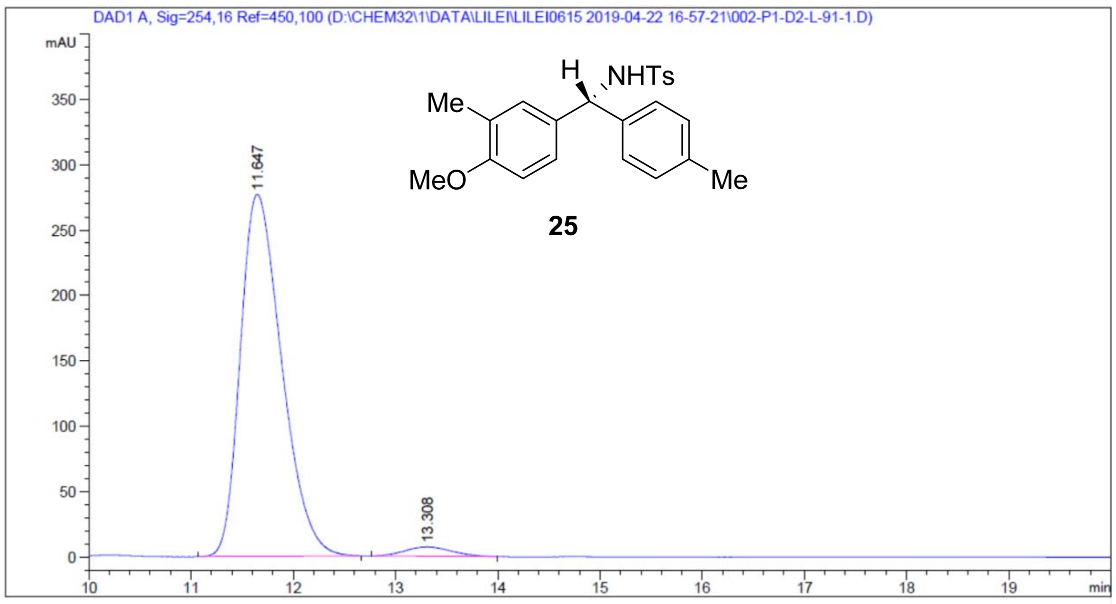

Peak #	RetTime [min]	Type	Width [min]	Area [mAU*s]	Height [mAU]	Area %
1	11.647	BB	0.4264	7768.91357	276.81674	97.1673
2	13.308	BB	0.3757	226.48726	7.11790	2.8327

Supplementary Figure 127. HPLC spectra of compound *rac*-**25** and compound **25**

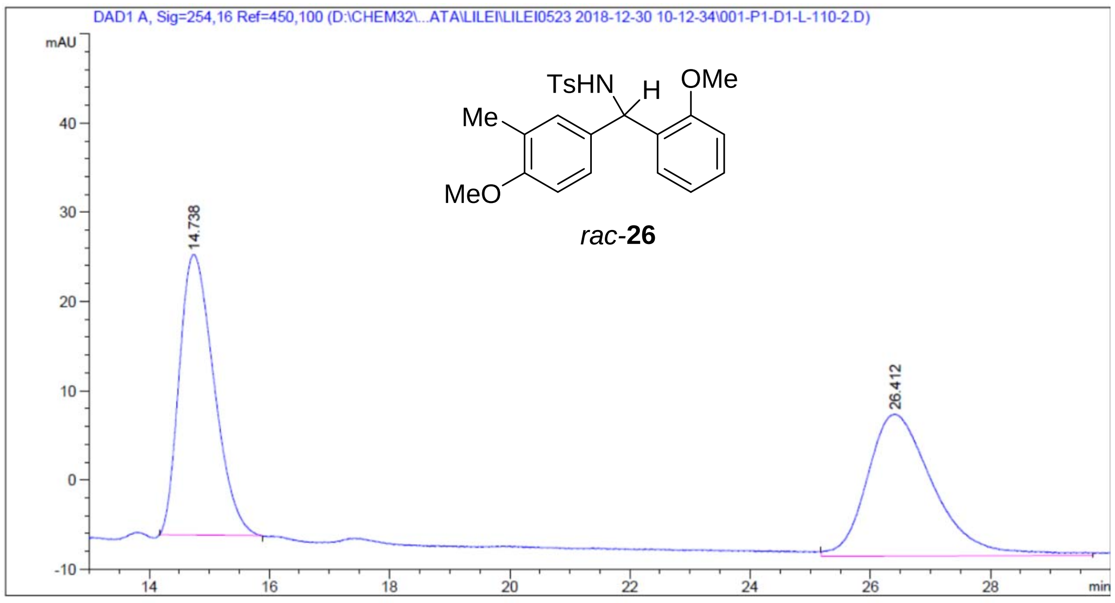

Peak #	RetTime [min]	Type	Width [min]	Area [mAU*s]	Height [mAU]	Area %
1	14.738	MM	0.6676	1259.78162	31.45198	50.8558
2	26.412	MM	1.2756	1217.38293	15.90597	49.1442

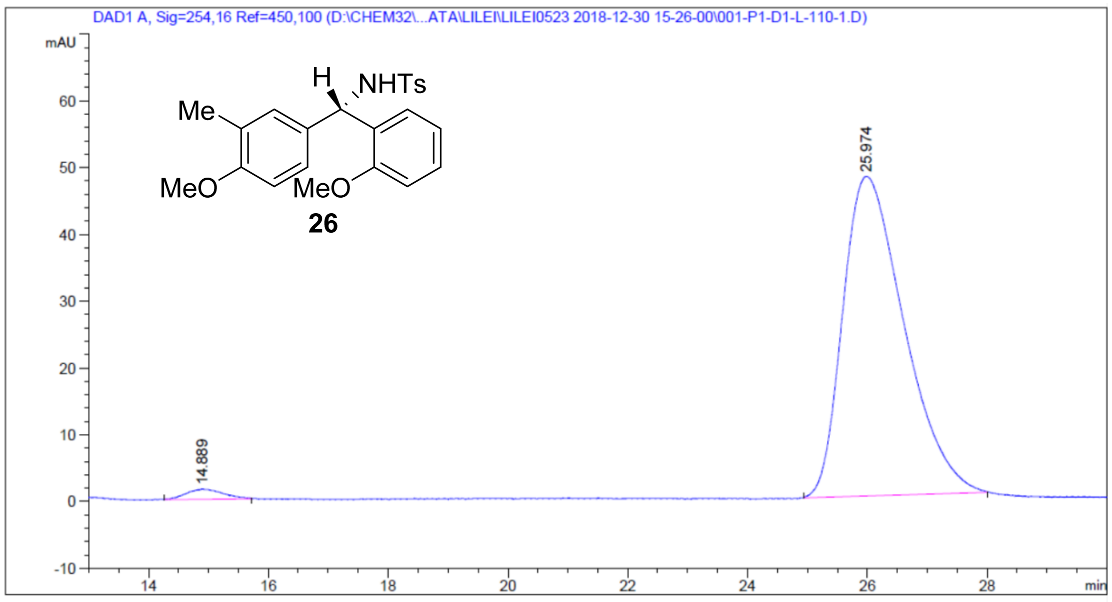

Peak #	RetTime [min]	Type	Width [min]	Area [mAU*s]	Height [mAU]	Area %
1	14.889	MM	0.7313	66.47557	1.51494	1.9215
2	25.974	BB	0.8312	3393.10376	47.82643	98.0785

Supplementary Figure 128. HPLC spectra of compound *rac*-**26** and compound **26**

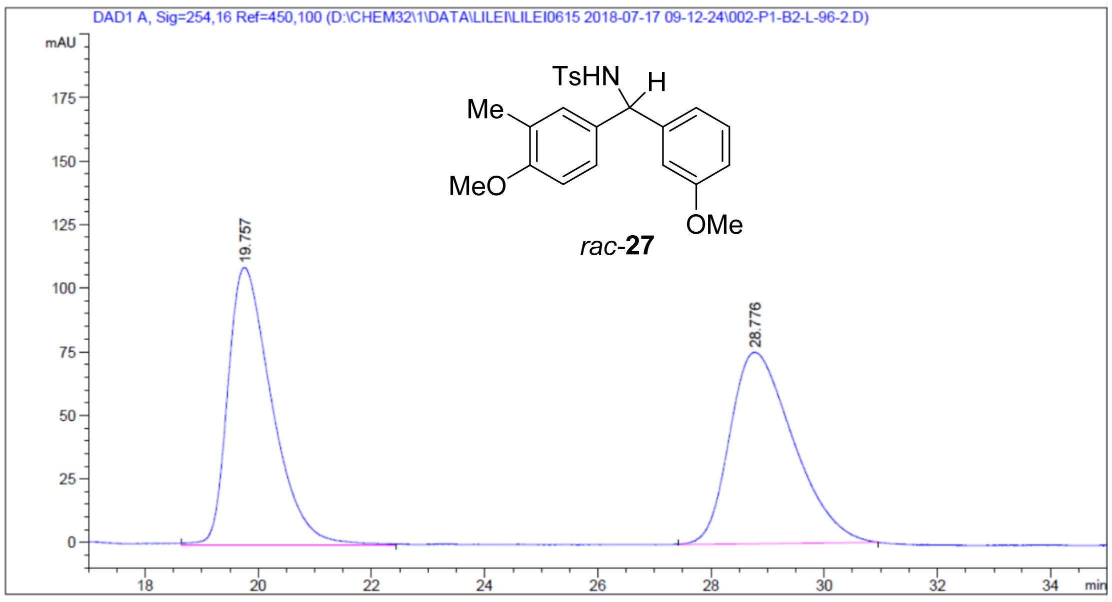

Peak #	RetTime [min]	Type	Width [min]	Area [mAU*s]	Height [mAU]	Area %
1	19.757	MM	0.9009	5902.12256	109.18343	50.1102
2	28.776	BB	0.9160	5876.15430	75.29079	49.8898

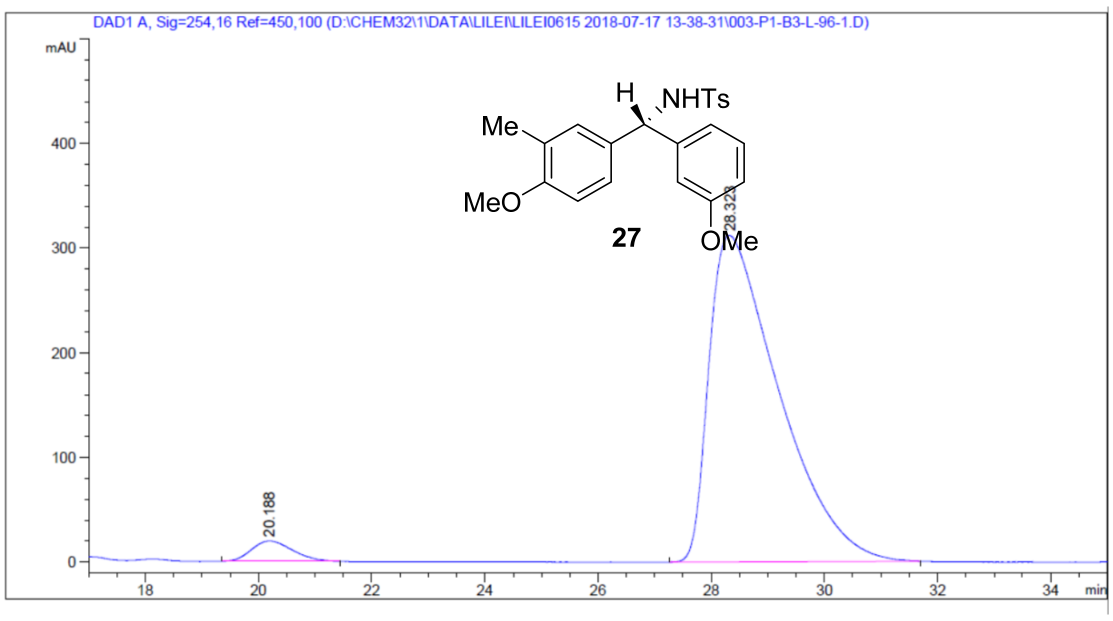

Peak #	RetTime [min]	Type	Width [min]	Area [mAU*s]	Height [mAU]	Area %
1	20.188	BB	0.5962	989.96973	19.48591	3.5602
2	28.323	BB	1.0106	2.68163e4	311.89618	96.4398

Supplementary Figure 129. HPLC spectra of compound *rac*-**27** and compound **27**

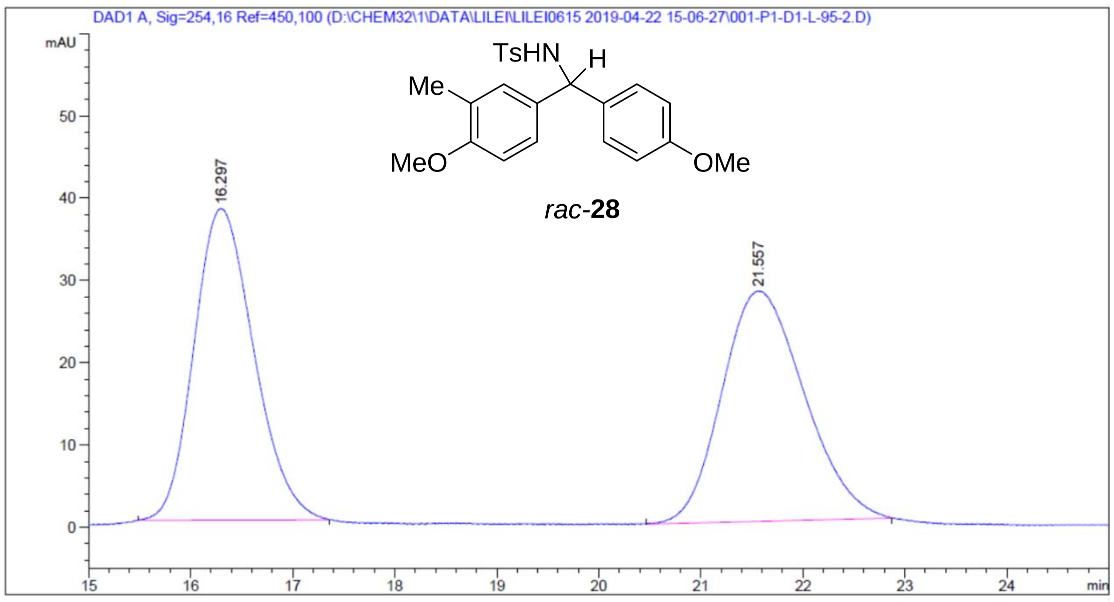

Peak #	RetTime [min]	Type	Width [min]	Area [mAU*s]	Height [mAU]	Area %
1	16.297	BB	0.4795	1548.00354	37.90434	49.6173
2	21.557	VB R	0.6608	1571.88513	28.04792	50.3827

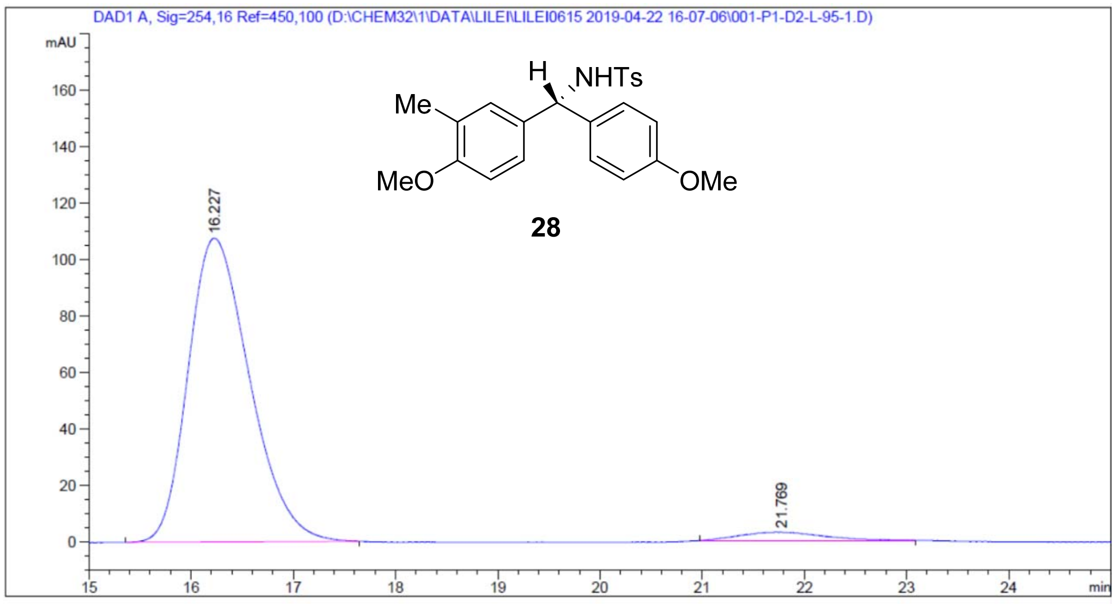

Peak #	RetTime [min]	Type	Width [min]	Area [mAU*s]	Height [mAU]	Area %
1	16.227	VV R	0.5048	4507.67773	107.69107	96.4791
2	21.769	MM	0.9400	164.50443	2.91683	3.5209

Supplementary Figure 130. HPLC spectra of compound *rac*-**28** and compound **28**

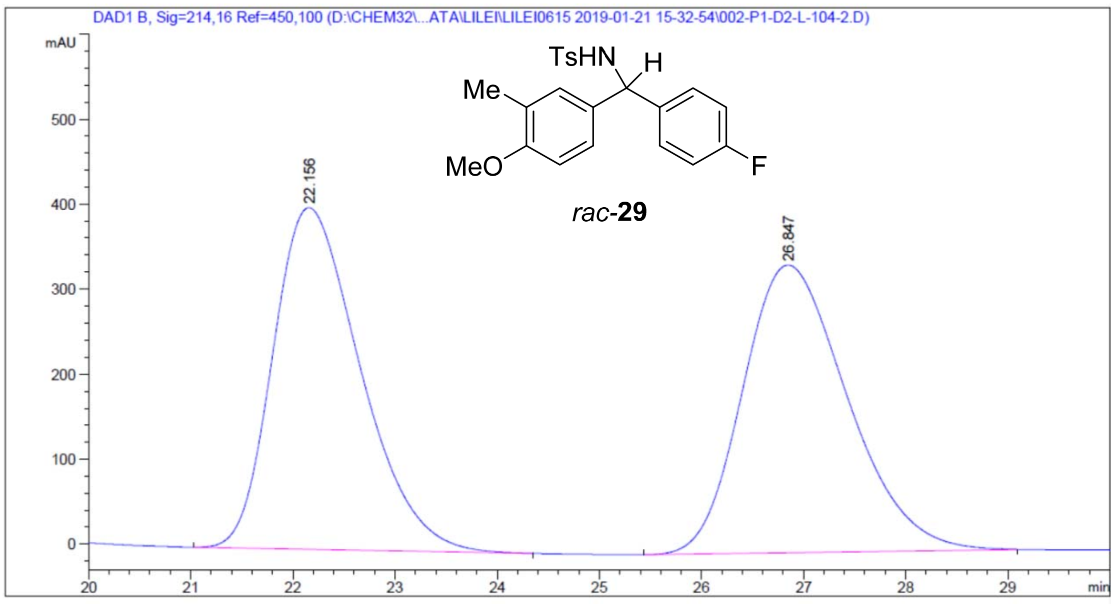

Peak #	RetTime [min]	Type	Width [min]	Area [mAU*s]	Height [mAU]	Area %
1	22.156	BB	0.8238	2.39097e4	401.76285	49.8236
2	26.847	BB	0.8572	2.40790e4	338.38989	50.1764

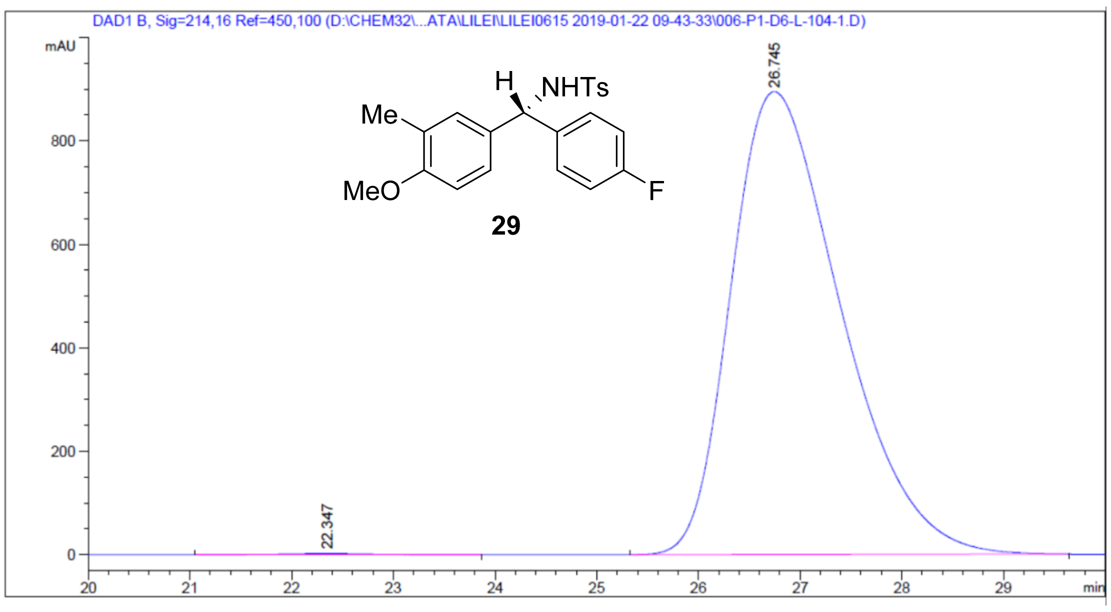

Peak #	RetTime [min]	Type	Width [min]	Area [mAU*s]	Height [mAU]	Area %
1	22.347	MM	1.0572	163.27710	2.57396	0.2425
2	26.745	VV R	0.8812	6.71587e4	894.77472	99.7575

Supplementary Figure 131. HPLC spectra of compound *rac*-**29** and compound **29**

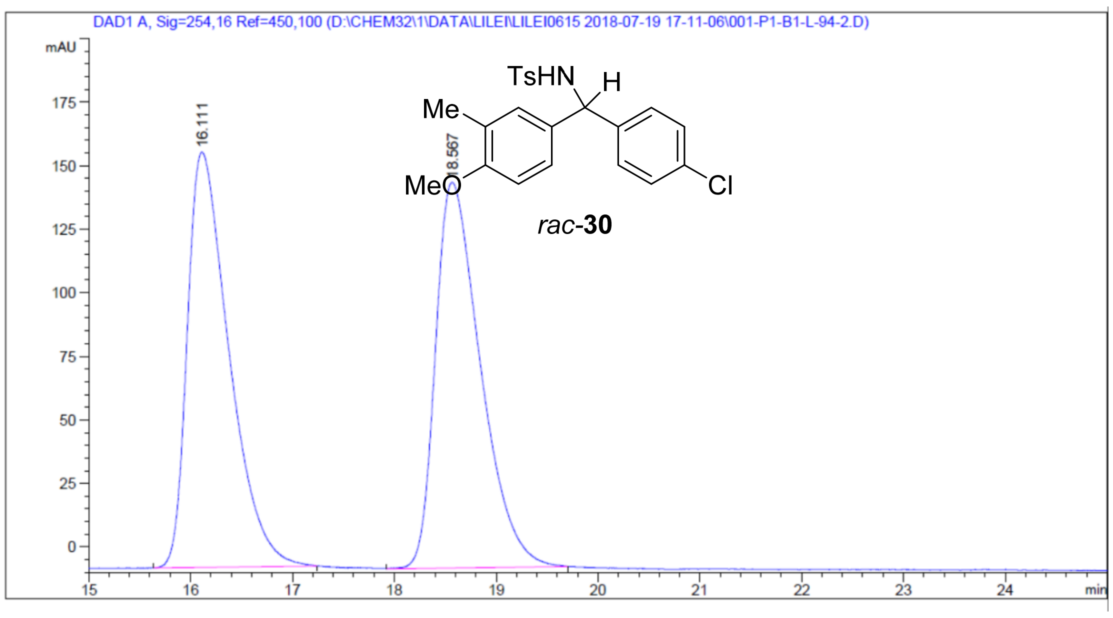

Peak #	RetTime [min]	Type	Width [min]	Area [mAU*s]	Height [mAU]	Area %
1	16.111	BB	0.4126	4644.79053	163.42624	49.9365
2	18.567	VB R	0.4150	4656.61182	151.73285	50.0635

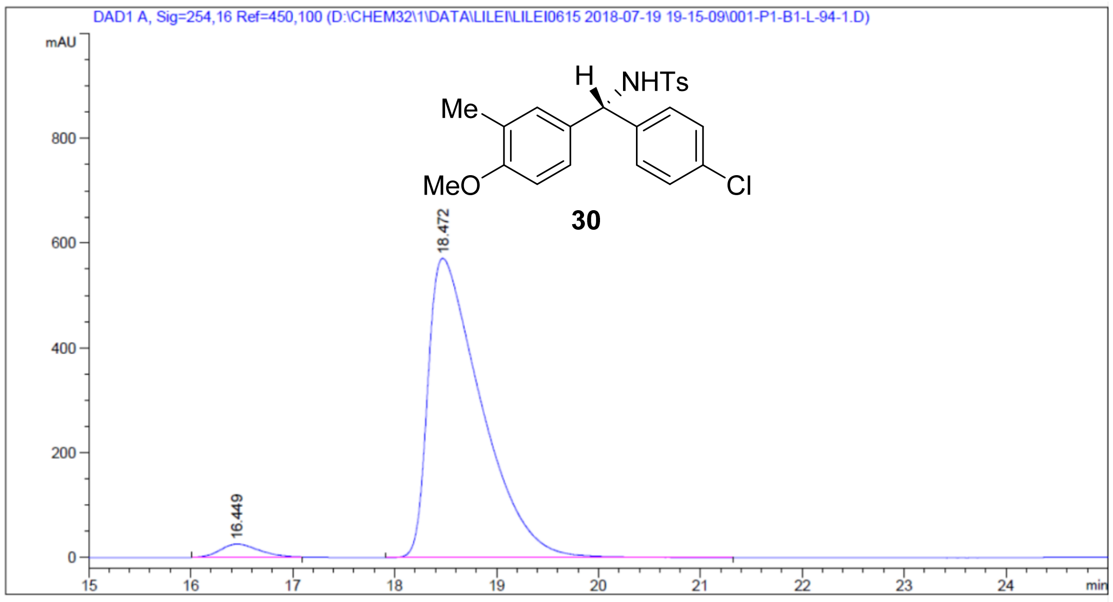

Peak #	RetTime [min]	Type	Width [min]	Area [mAU*s]	Height [mAU]	Area %
1	16.449	MM	0.4407	658.99823	24.92173	3.0795
2	18.472	MM	0.6052	2.07406e4	571.20917	96.9205

Supplementary Figure 132. HPLC spectra of compound *rac*-**30** and compound **30**

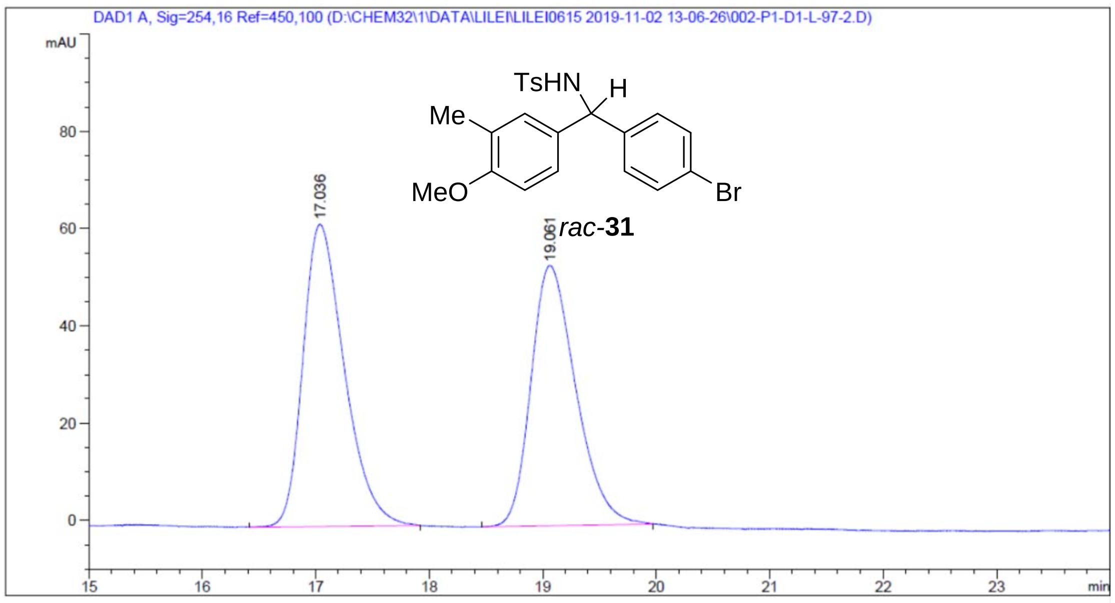

Peak #	RetTime [min]	Type	Width [min]	Area [mAU*s]	Height [mAU]	Area %
1	17.036	VB R	0.3594	1575.17993	62.10548	51.7726
2	19.061	BB	0.3677	1467.31641	53.45673	48.2274

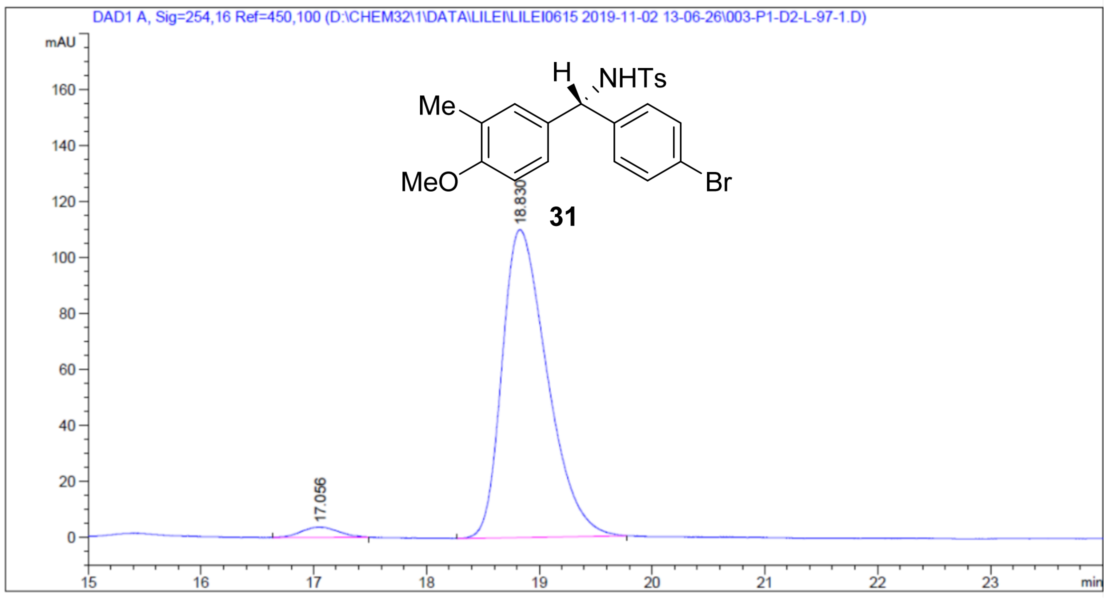

Peak #	RetTime [min]	Type	Width [min]	Area [mAU*s]	Height [mAU]	Area %
1	17.056	BB	0.2679	82.36713	3.64098	2.6200
2	18.830	VB R	0.3745	3061.37744	110.06537	97.3800

Supplementary Figure 133. HPLC spectra of compound *rac*-**31** and compound **31**

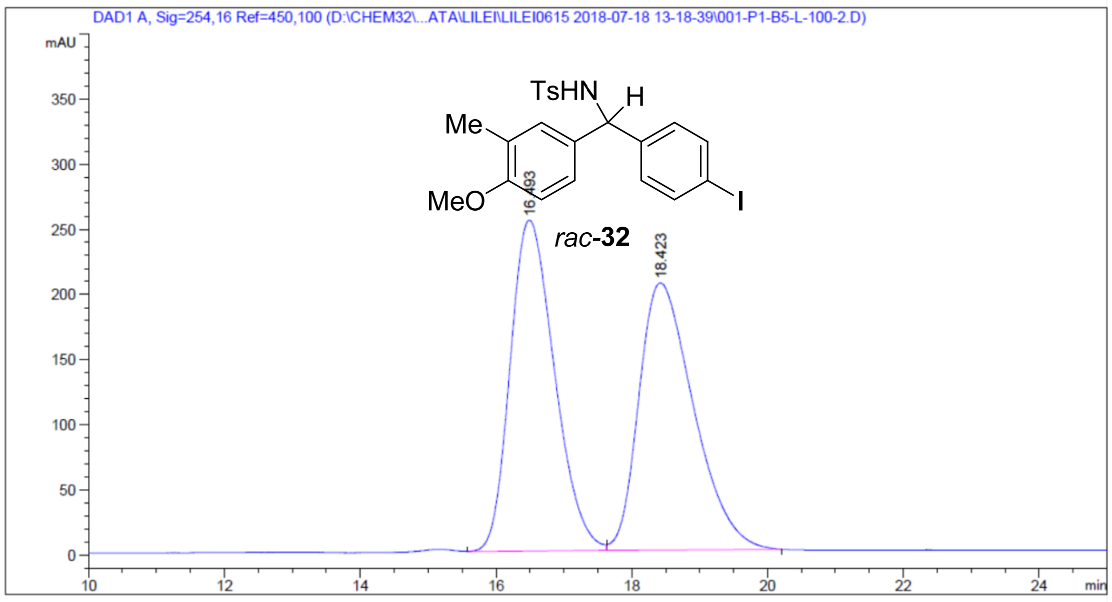

Peak #	RetTime [min]	Type	Width [min]	Area [mAU*s]	Height [mAU]	Area %
1	16.493	BV	0.6140	1.15014e4	254.12802	50.0238
2	18.423	VB	0.6683	1.14904e4	205.20375	49.9762

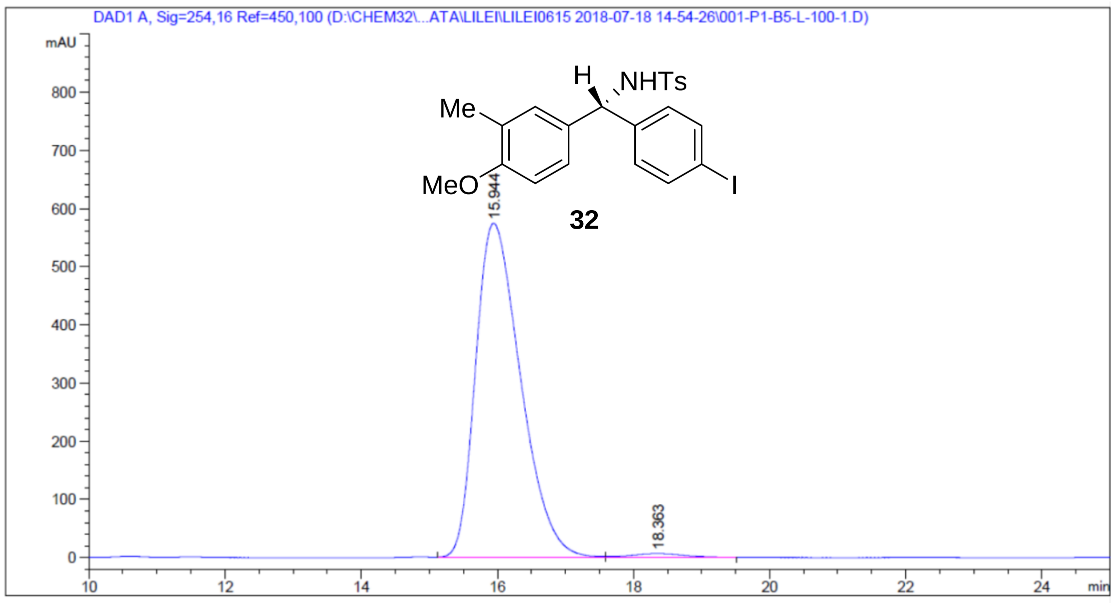

Peak #	RetTime [min]	Type	Width [min]	Area [mAU*s]	Height [mAU]	Area %
1	15.944	MM	0.7666	2.64220e4	574.43573	98.6687
2	18.363	MM	0.9333	356.49509	6.36653	1.3313

Supplementary Figure 134. HPLC spectra of compound *rac*-**32** and compound **32**

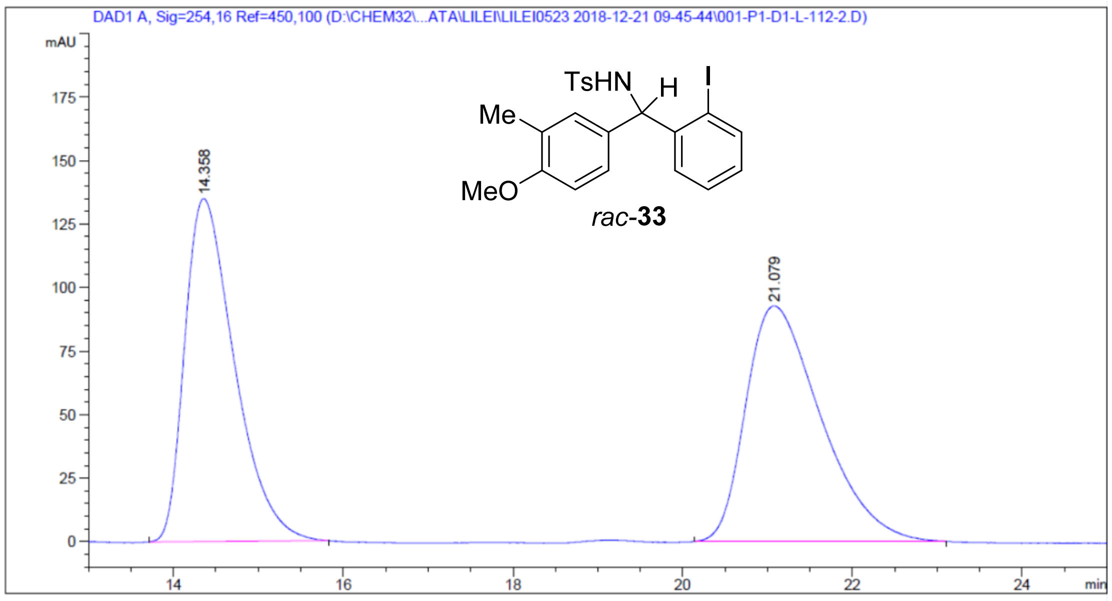

Peak #	RetTime [min]	Type	Width [min]	Area [mAU*s]	Height [mAU]	Area %
1	14.358	BB	0.5479	5498.99512	134.94783	49.0746
2	21.079	BB	0.7235	5706.37500	92.78667	50.9254

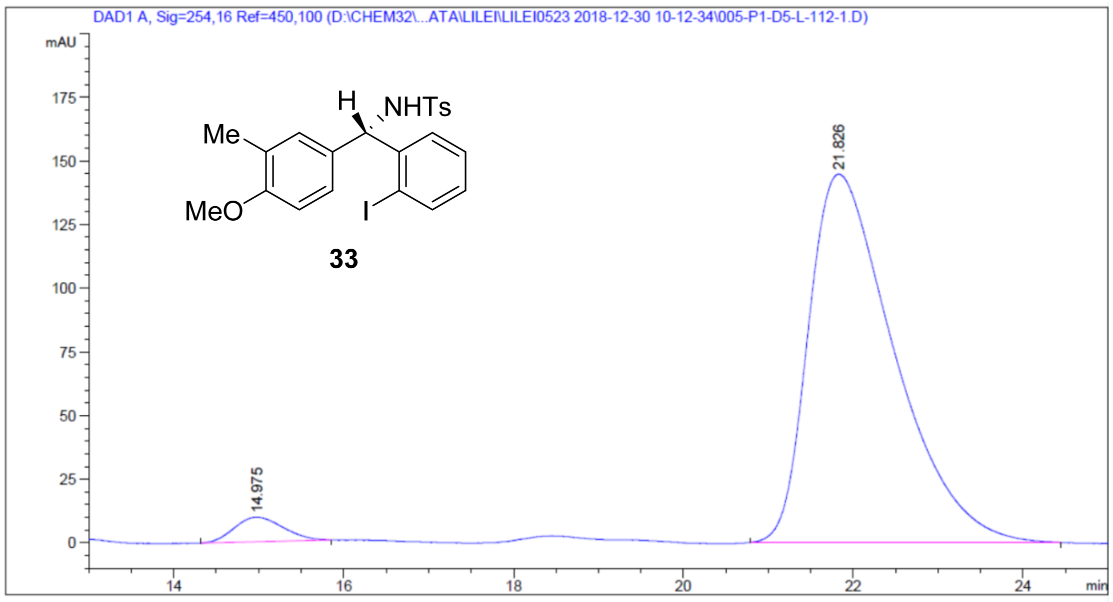

Peak #	RetTime [min]	Type	Width [min]	Area [mAU*s]	Height [mAU]	Area %
1	14.975	BB	0.4715	385.00226	9.63877	3.6550
2	21.826	BB	0.8346	1.01485e4	144.72569	96.3450

Supplementary Figure 135. HPLC spectra of compound *rac*-**33** and compound **33**

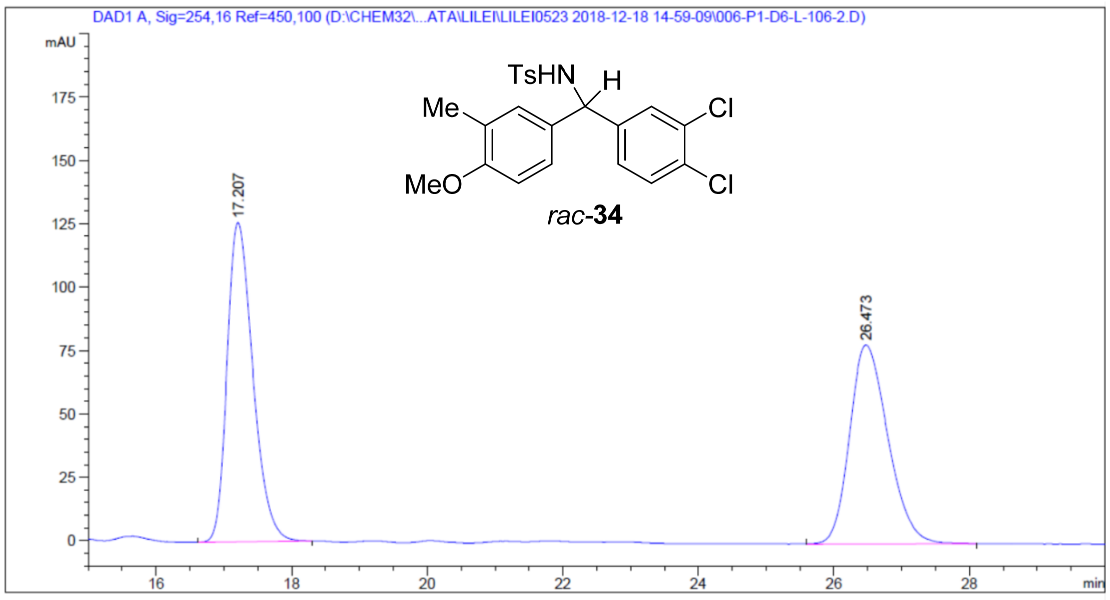

Peak #	RetTime [min]	Type	Width [min]	Area [mAU*s]	Height [mAU]	Area %
1	17.207	BB	0.4064	3328.70605	125.90872	51.9150
2	26.473	BB	0.6086	3083.13843	78.44267	48.0850

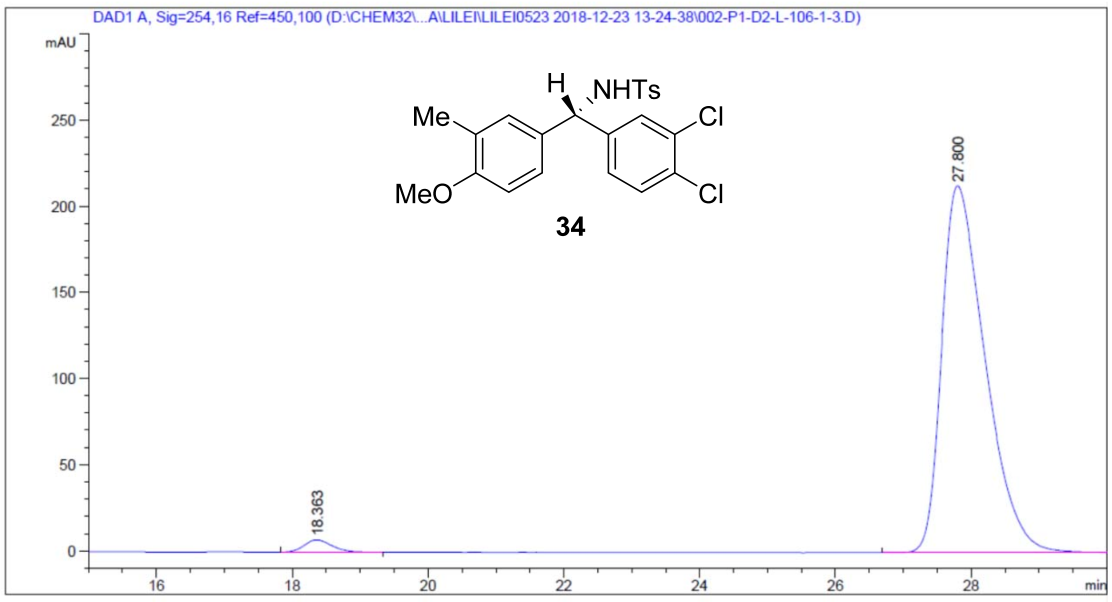

Peak #	RetTime [min]	Type	Width [min]	Area [mAU*s]	Height [mAU]	Area %
1	18.363	BB	0.4198	202.20833	7.10911	2.0996
2	27.800	BBA	0.6627	9428.70215	212.88376	97.9004

Supplementary Figure 136. HPLC spectra of compound *rac*-**34** and compound **34**

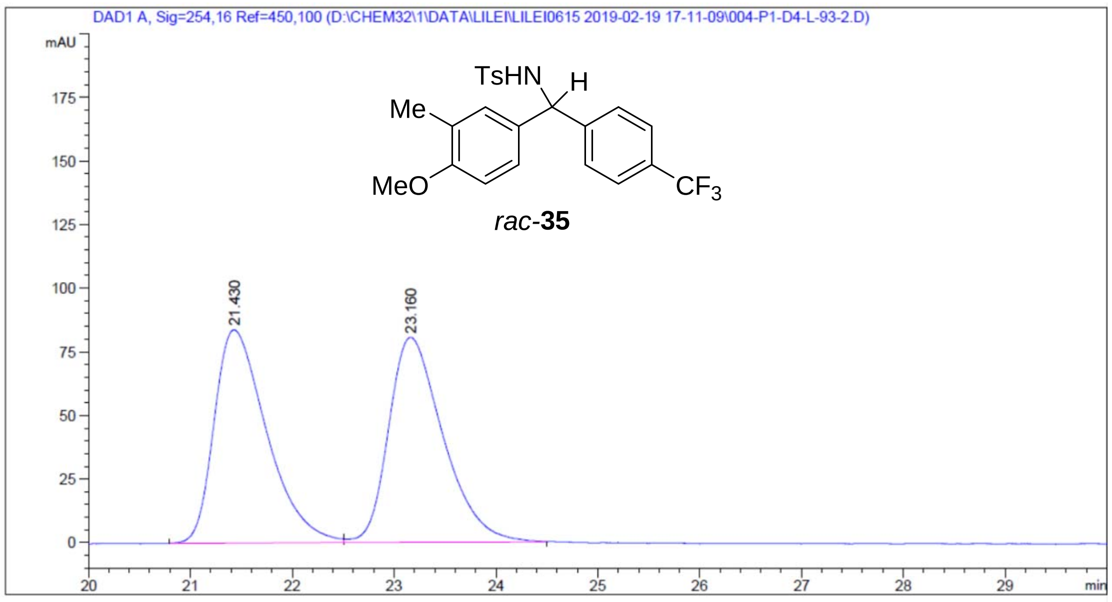

Peak #	RetTime [min]	Type	Width [min]	Area [mAU*s]	Height [mAU]	Area %
1	21.430	BV	0.4976	3002.51001	83.78690	50.0916
2	23.160	VB	0.5117	2991.52368	80.56073	49.9084

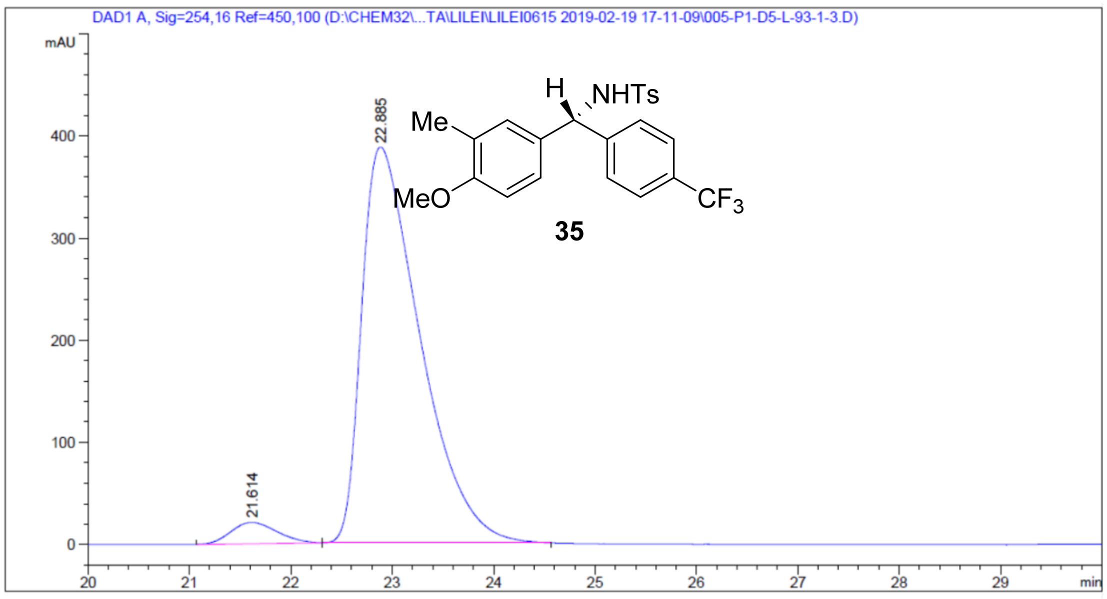

Peak #	RetTime [min]	Type	Width [min]	Area [mAU*s]	Height [mAU]	Area %
1	21.614	BB	0.3742	660.12079	20.94738	4.0860
2	22.885	BB	0.5799	1.54955e4	387.57205	95.9140

Supplementary Figure 137. HPLC spectra of compound *rac*-**35** and compound **35**

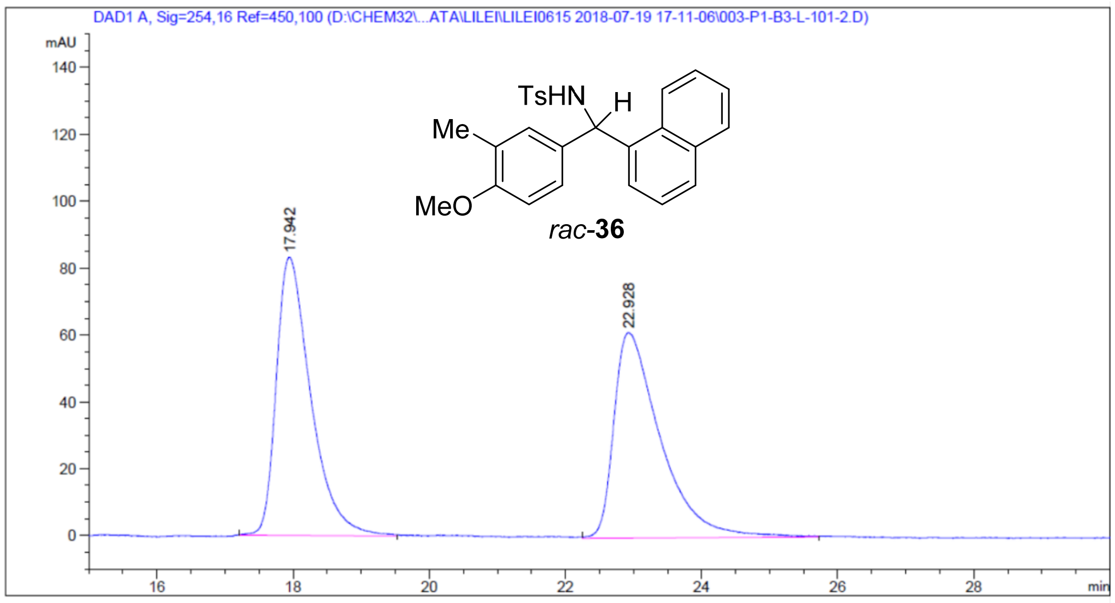

Peak #	RetTime [min]	Type	Width [min]	Area [mAU*s]	Height [mAU]	Area %
1	17.942	MM	0.5882	2935.49243	83.17239	50.0600
2	22.928	MM	0.7945	2928.45288	61.42985	49.9400

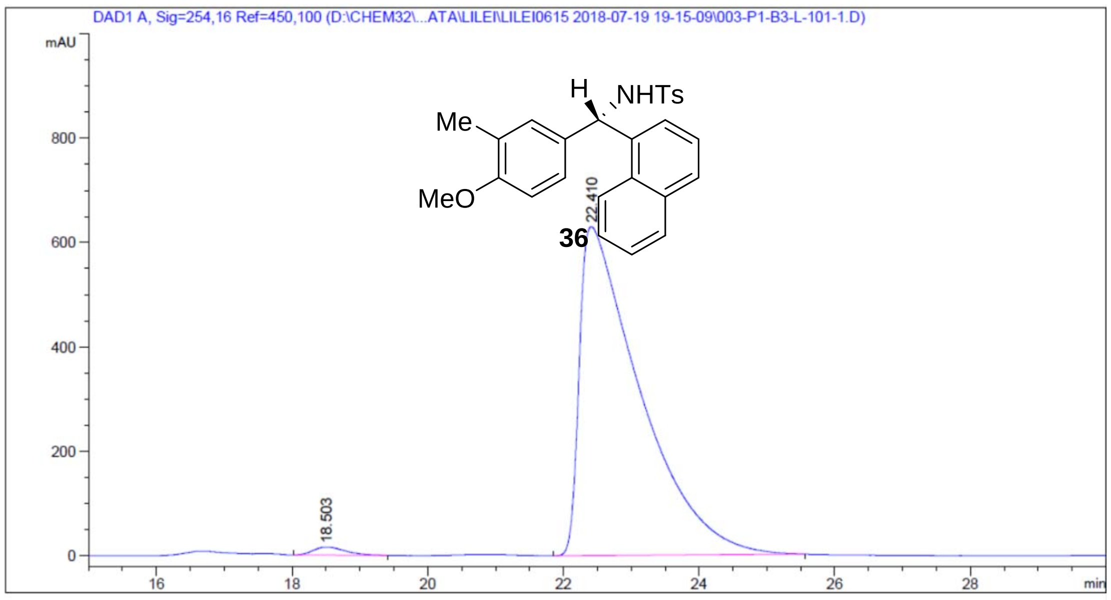

Peak #	RetTime [min]	Type	Width [min]	Area [mAU*s]	Height [mAU]	Area %
1	18.503	BV R	0.3878	512.61694	15.66279	1.2948
2	22.410	BV R	0.7957	3.90785e4	630.00037	98.7052

Supplementary Figure 138. HPLC spectra of compound *rac*-**36** and compound **36**

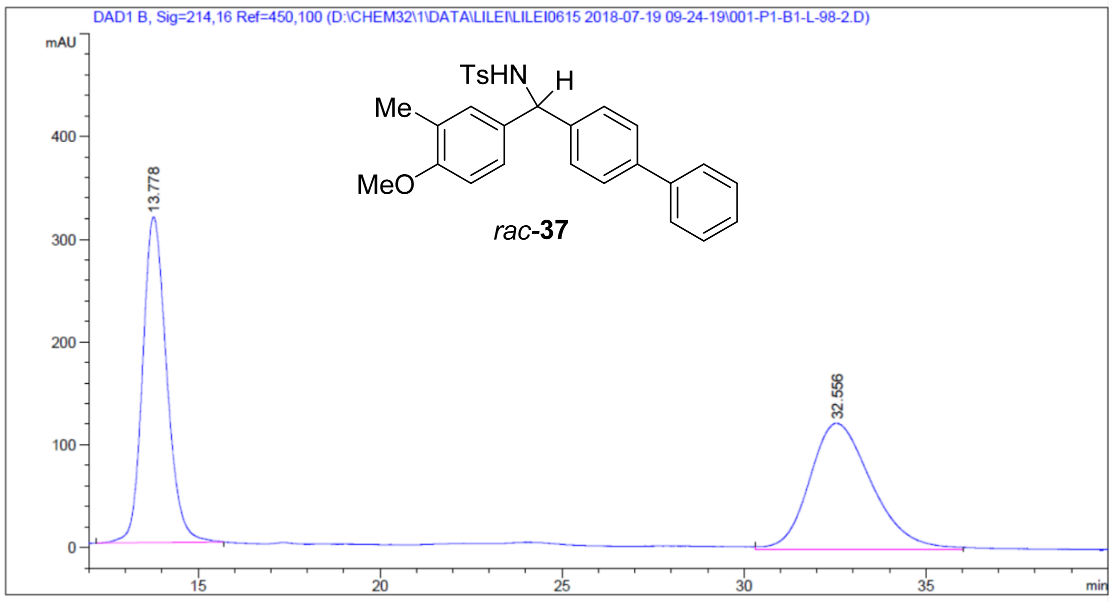

Peak #	RetTime [min]	Type	Width [min]	Area [mAU*s]	Height [mAU]	Area %
1	13.778	VV R	0.6390	1.45623e4	316.94467	49.9671
2	32.556	MM	1.9812	1.45815e4	122.66776	50.0329

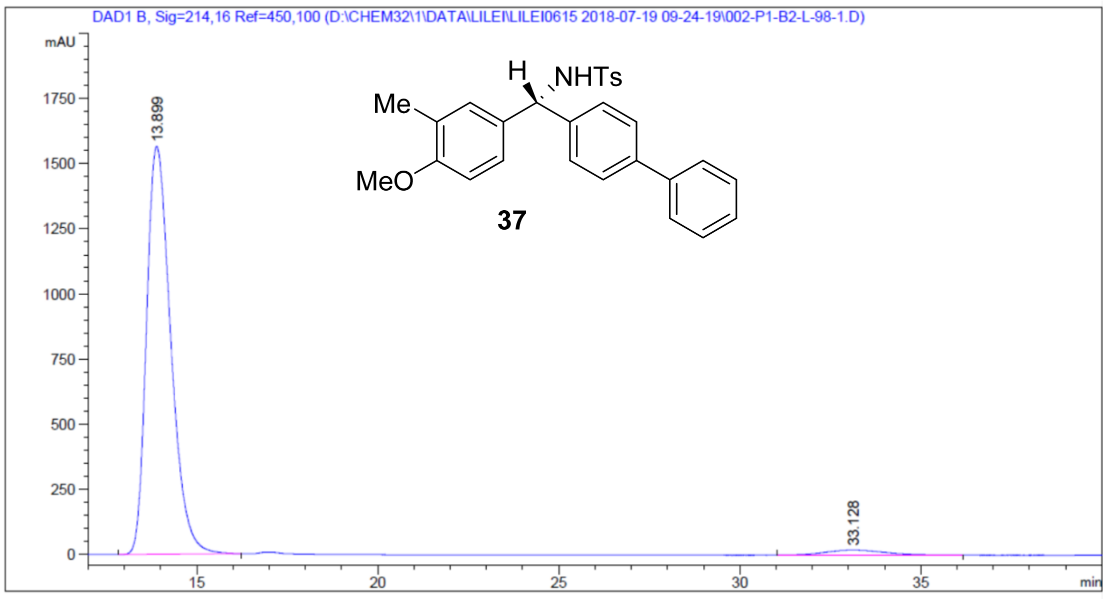

Peak #	RetTime [min]	Type	Width [min]	Area [mAU*s]	Height [mAU]	Area %
1	13.899	VV R	0.5444	7.28181e4	1566.17285	96.8842
2	33.128	MM	1.9618	2341.84570	19.89547	3.1158

Supplementary Figure 139. HPLC spectra of compound *rac*-**37** and compound **37**

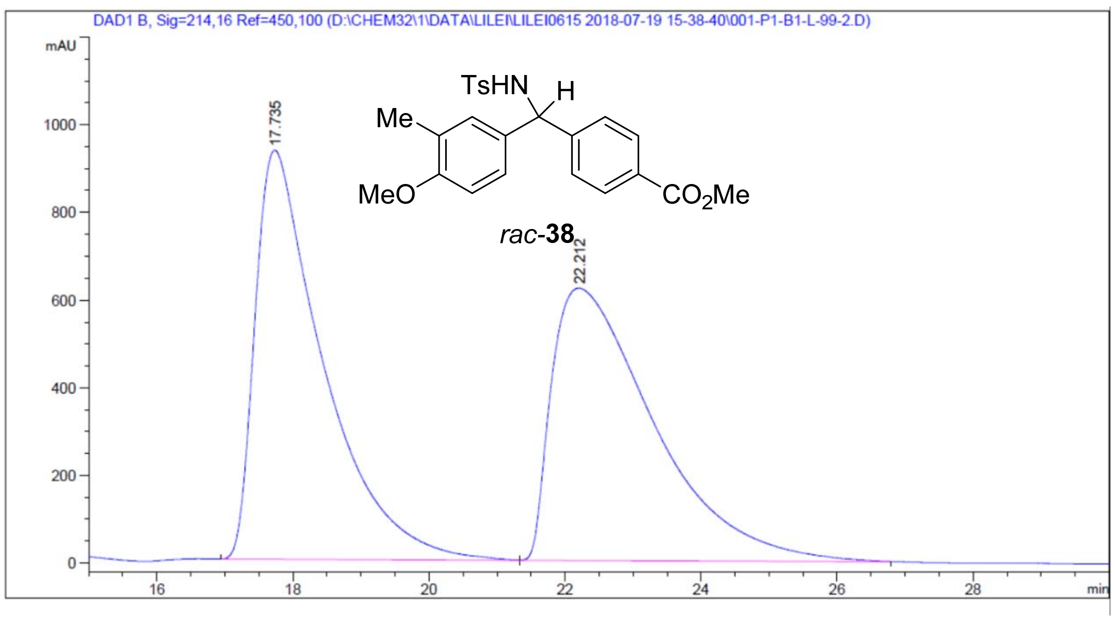

Peak #	RetTime [min]	Type	Width [min]	Area [mAU*s]	Height [mAU]	Area %
1	17.735	BV R	0.9060	6.50940e4	934.07520	50.1370
2	22.212	BV R	1.2207	6.47382e4	621.25299	49.8630

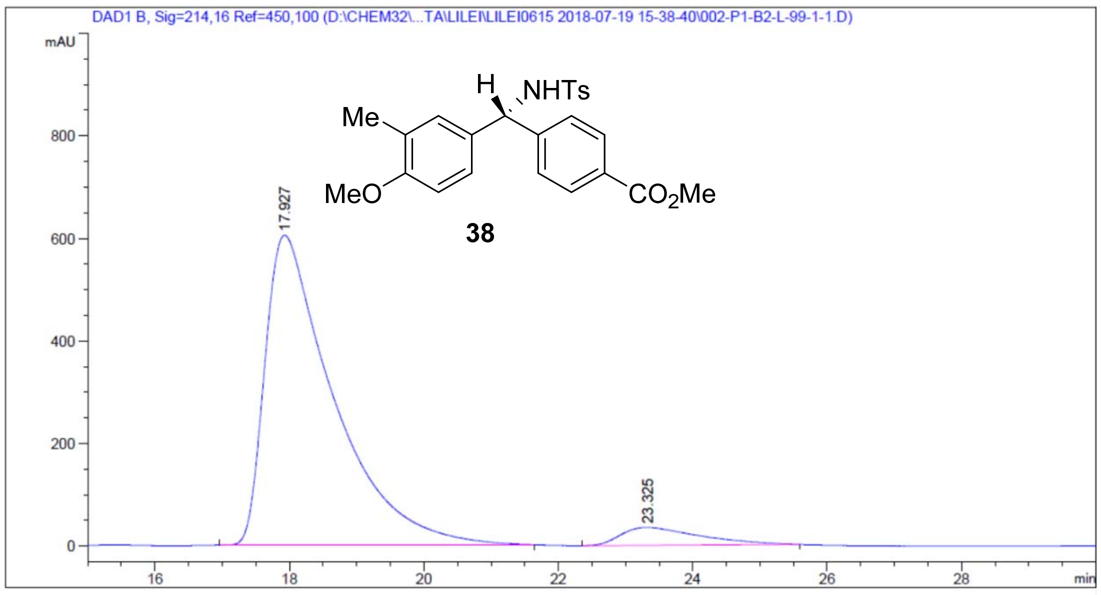

Peak #	RetTime [min]	Type	Width [min]	Area [mAU*s]	Height [mAU]	Area %
1	17.927	VV R	0.9264	4.28551e4	604.26129	93.7791
2	23.325	BV R	0.9554	2842.81104	34.87894	6.2209

Supplementary Figure 140. HPLC spectra of compound *rac*-**38** and compound **38**

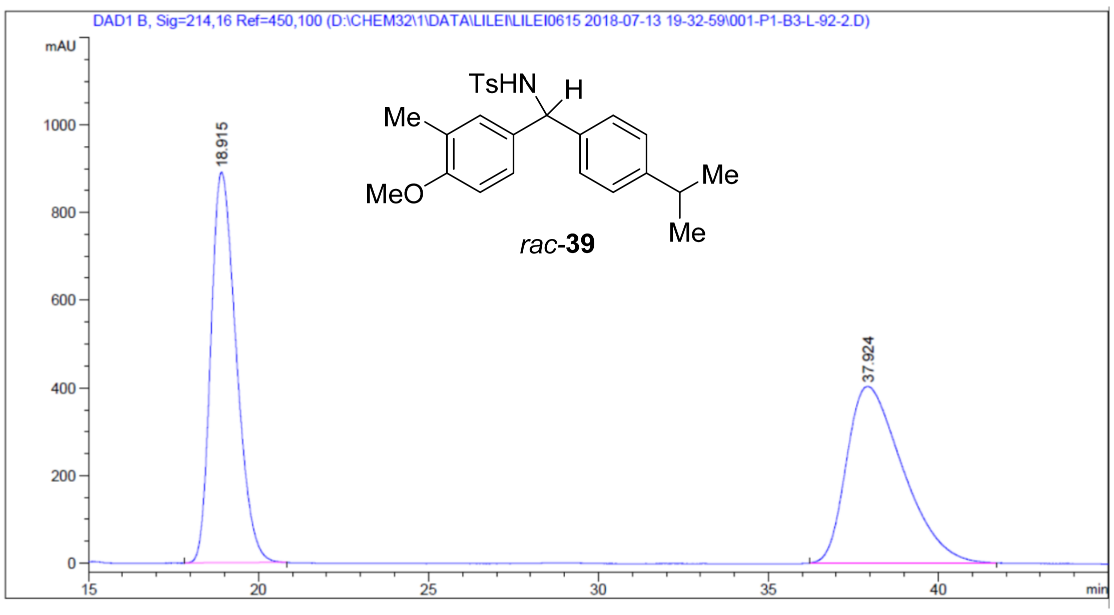

Peak #	RetTime [min]	Type	Width [min]	Area [mAU*s]	Height [mAU]	Area %
1	18.915	BV R	0.7474	4.63564e4	891.84796	49.9820
2	37.924	BV R	1.3586	4.63898e4	403.25381	50.0180

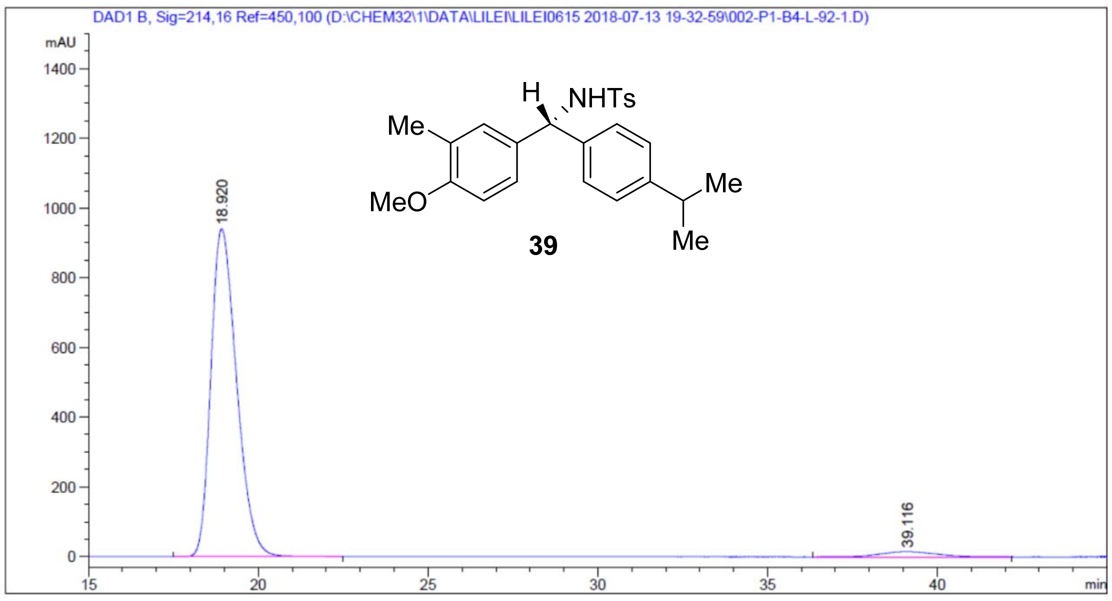

Peak #	RetTime [min]	Type	Width [min]	Area [mAU*s]	Height [mAU]	Area %
1	18.920	MM	0.8784	4.95814e4	940.71167	96.5618
2	39.116	MM	1.9557	1765.39600	15.04459	3.4382

Supplementary Figure 141. HPLC spectra of compound *rac*-**39** and compound **39**

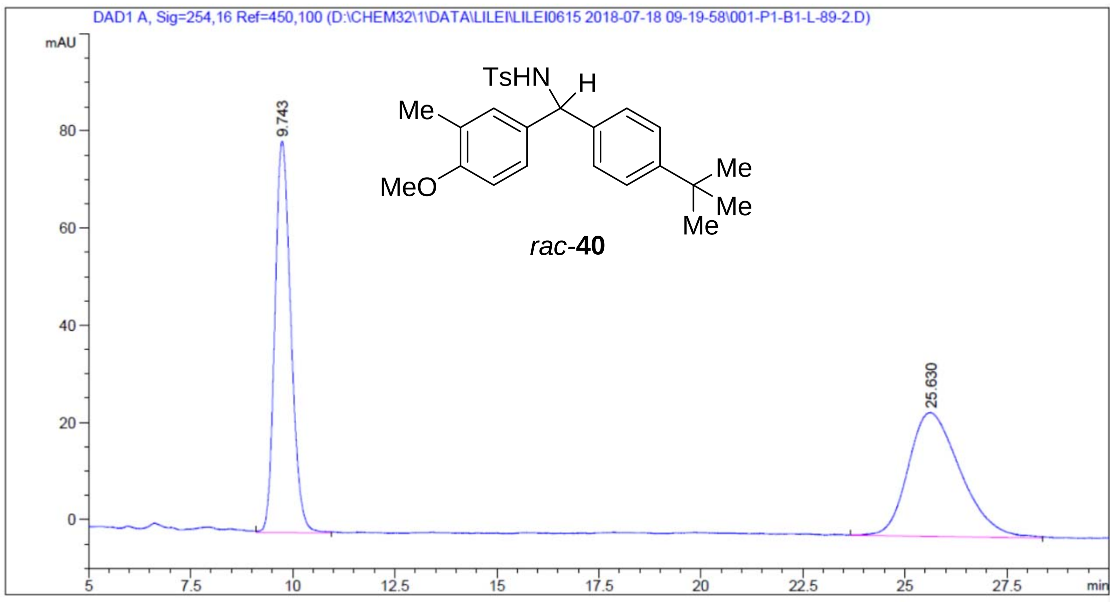

Peak #	RetTime [min]	Type	Width [min]	Area [mAU*s]	Height [mAU]	Area %
1	9.743	MM	0.4558	2202.70313	80.54964	49.2666
2	25.630	MM	1.4798	2268.28784	25.54752	50.7334

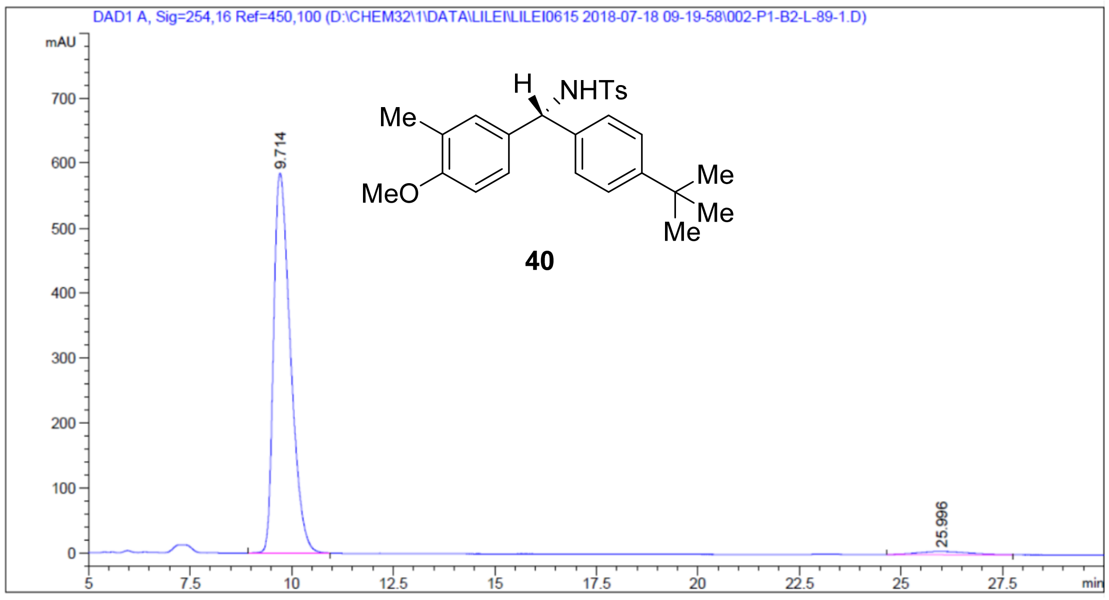

Peak #	RetTime [min]	Type	Width [min]	Area [mAU*s]	Height [mAU]	Area %
1	9.714	VB R	0.4385	1.69646e4	585.51563	97.5842
2	25.996	MM	1.4117	419.97629	4.95811	2.4158

Supplementary Figure 142. HPLC spectra of compound *rac*-**40** and compound **40**

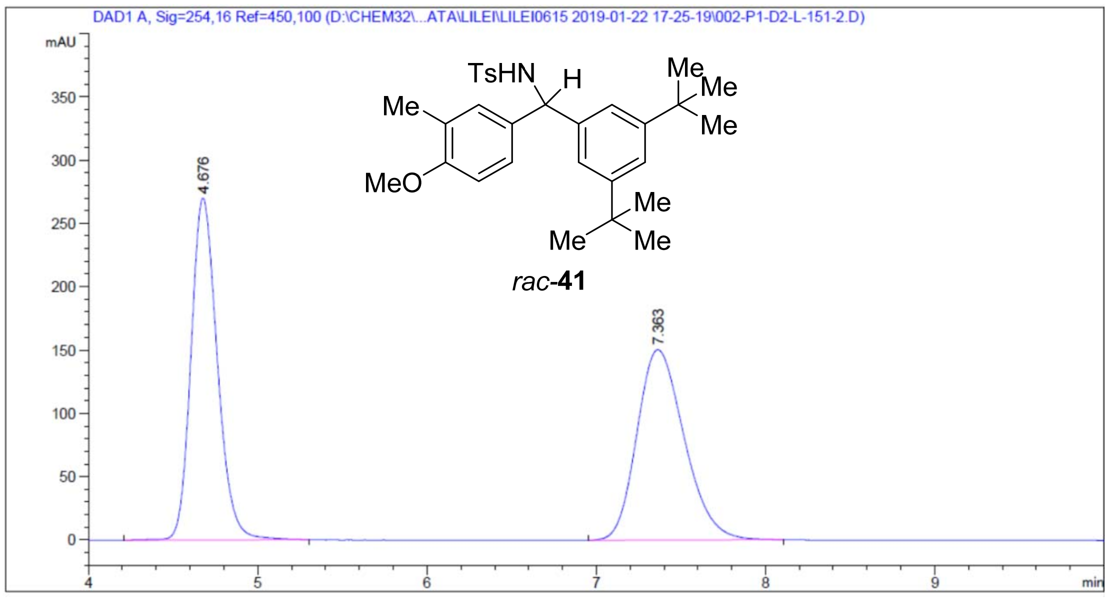

Peak #	RetTime [min]	Type	Width [min]	Area [mAU*s]	Height [mAU]	Area %
1	4.676	VB R	0.1662	2923.09619	270.17911	50.0319
2	7.363	BV R	0.2966	2919.37354	150.31570	49.9681

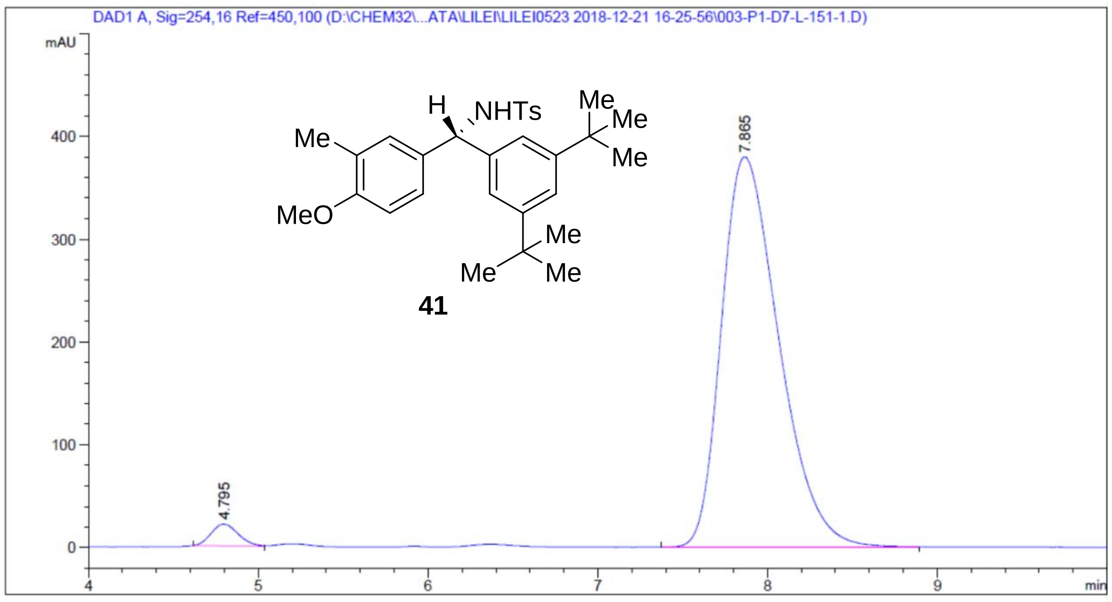

Peak #	RetTime [min]	Type	Width [min]	Area [mAU*s]	Height [mAU]	Area %
1	4.795	MM	0.1830	232.21107	21.14409	2.5348
2	7.865	BB	0.3573	8928.88379	379.82013	97.4652

Supplementary Figure 143. HPLC spectra of compound *rac*-**41** and compound **41**

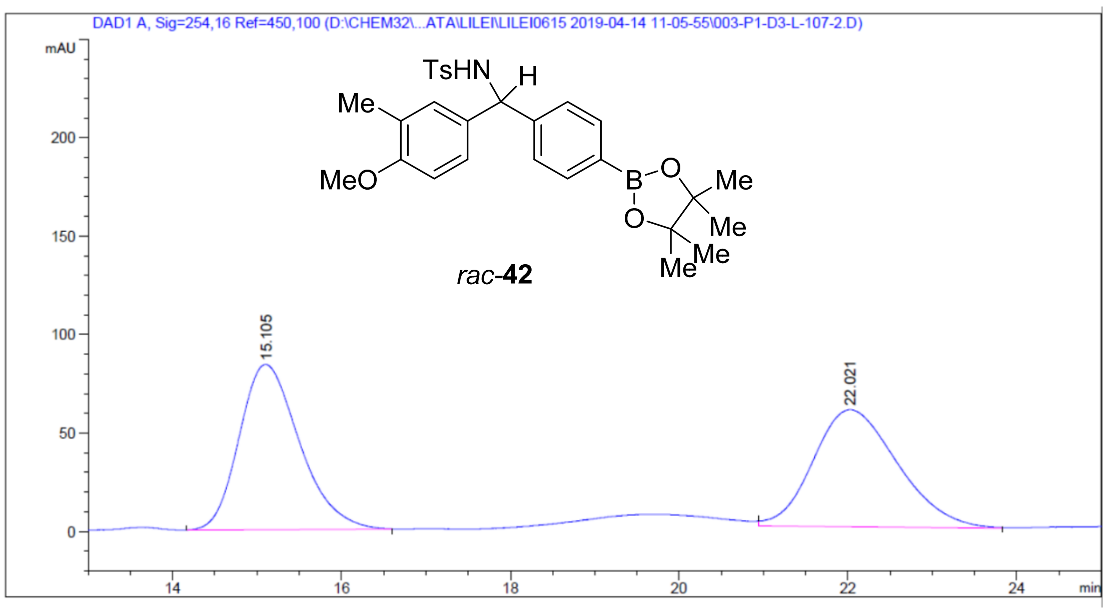

Peak #	RetTime [min]	Type	Width [min]	Area [mAU*s]	Height [mAU]	Area %
1	15.105	BV R	0.5941	4161.64014	83.99287	50.1603
2	22.021	MM	1.1588	4135.03857	59.47221	49.8397

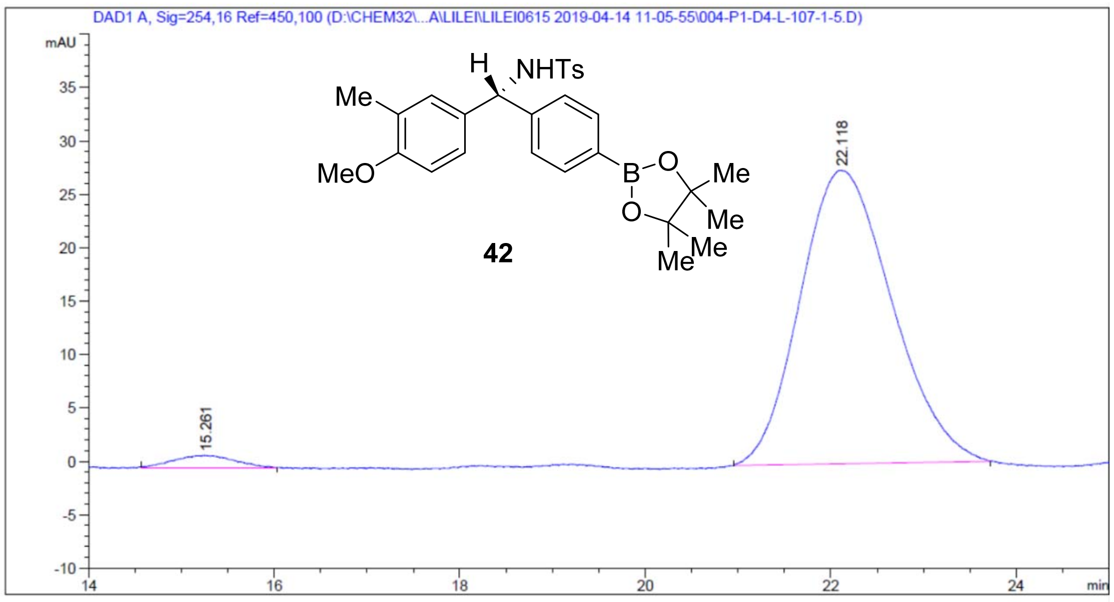

Peak #	RetTime [min]	Type	Width [min]	Area [mAU*s]	Height [mAU]	Area %
1	15.261	MM	0.7408	50.58488	1.13804	2.6091
2	22.118	BB	0.8068	1888.20459	27.49273	97.3909

Supplementary Figure 144. HPLC spectra of compound *rac*-**42** and compound **42**

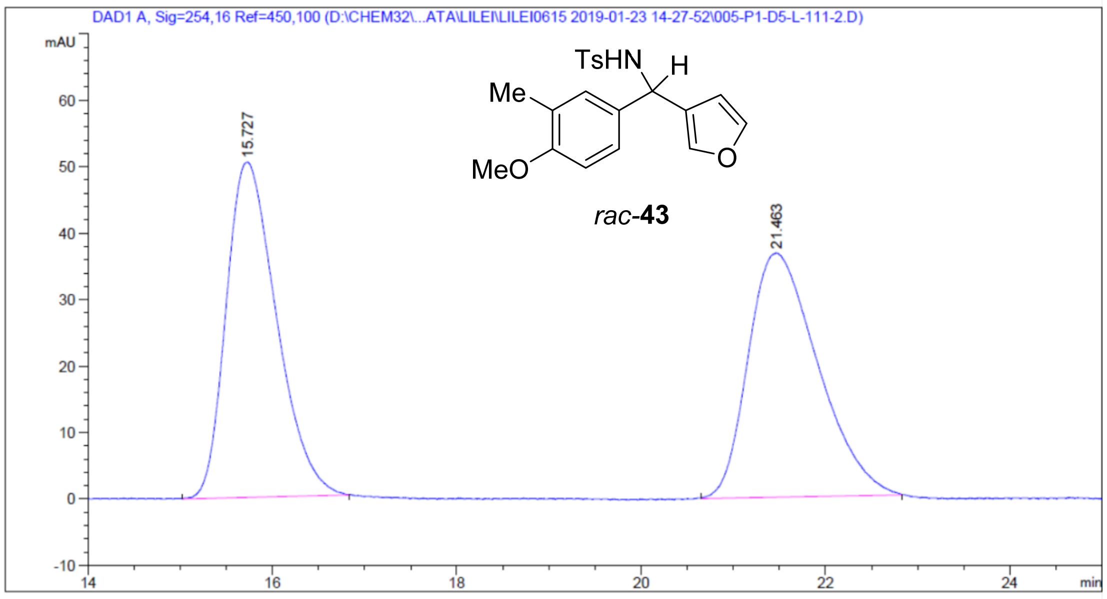

Peak #	RetTime [min]	Type	Width [min]	Area [mAU*s]	Height [mAU]	Area %
1	15.727	BV R	0.4491	1912.56445	50.46796	49.8320
2	21.463	BV R	0.6172	1925.45691	36.72396	50.1680

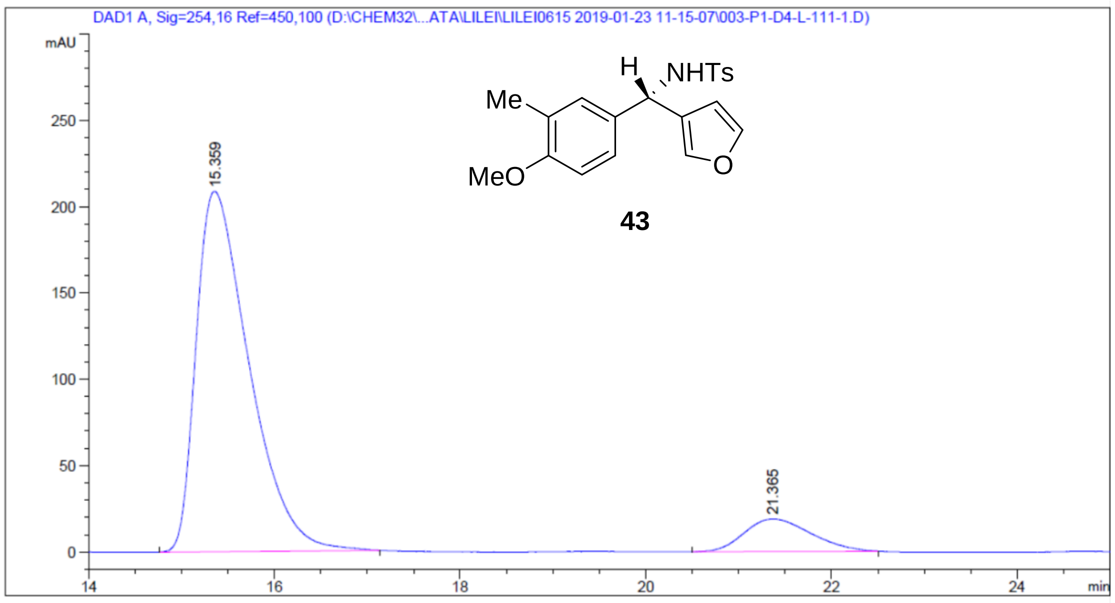

Peak #	RetTime [min]	Type	Width [min]	Area [mAU*s]	Height [mAU]	Area %
1	15.359	BV R	0.5496	8279.95117	208.83035	89.8096
2	21.365	VV R	0.5872	939.49994	18.84194	10.1904

Supplementary Figure 145. HPLC spectra of compound *rac*-**43** and compound **43**

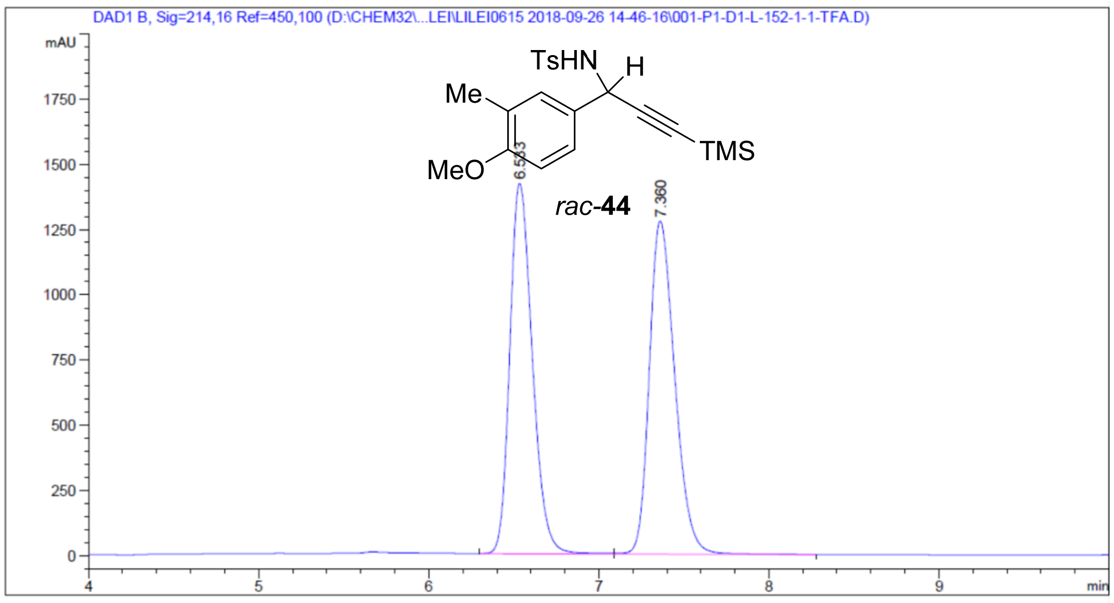

Peak #	RetTime [min]	Type	Width [min]	Area [mAU*s]	Height [mAU]	Area %
1	6.533	BV R	0.1428	1.30629e4	1419.74792	49.5760
2	7.360	VB	0.1629	1.32864e4	1276.61206	50.4240

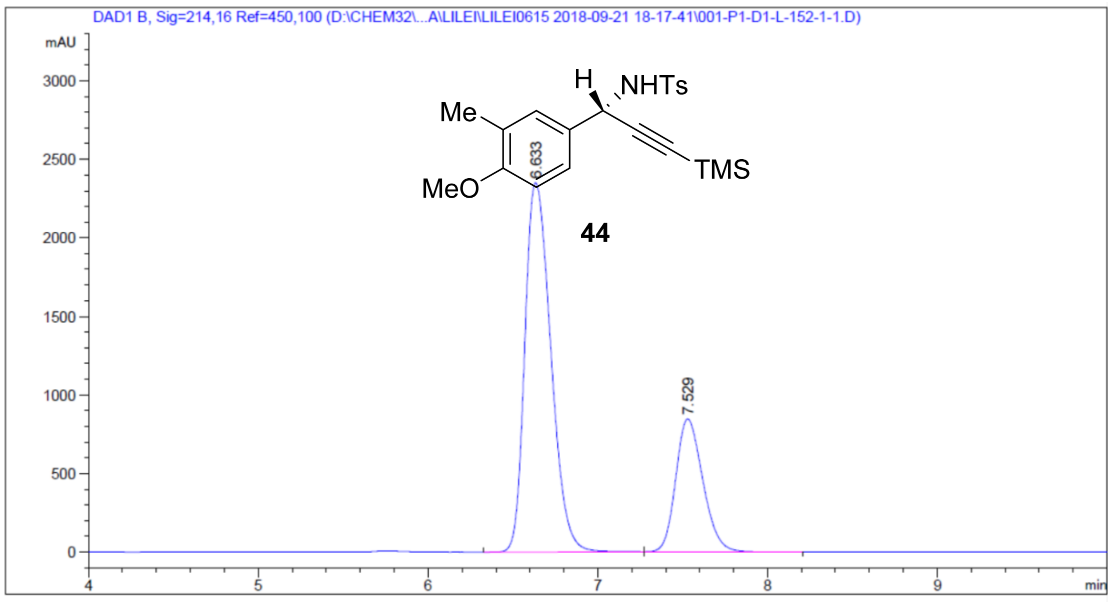

Peak #	RetTime [min]	Type	Width [min]	Area [mAU*s]	Height [mAU]	Area %
1	6.633	BV	0.1682	2.50257e4	2351.24634	72.5929
2	7.529	VB	0.1726	9448.32031	847.08667	27.4071

Supplementary Figure 146. HPLC spectra of compound *rac*-**44** and compound **44**

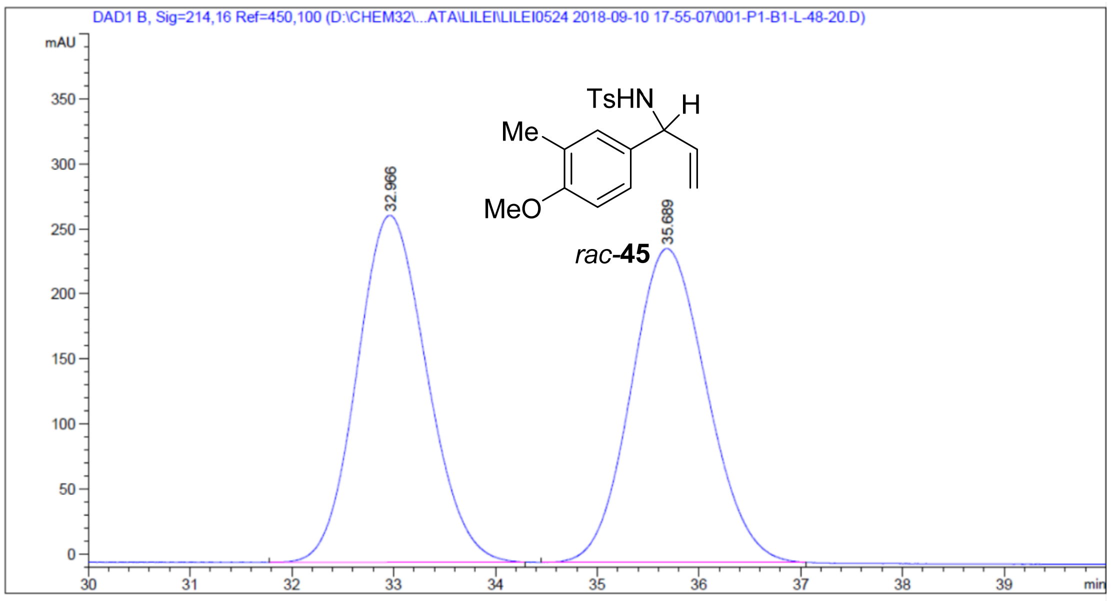

Peak #	RetTime [min]	Type	Width [min]	Area [mAU*s]	Height [mAU]	Area %
1	32.966	VV R	0.5850	1.27021e4	266.77682	50.4288
2	35.689	VV R	0.6182	1.24862e4	241.28525	49.5712

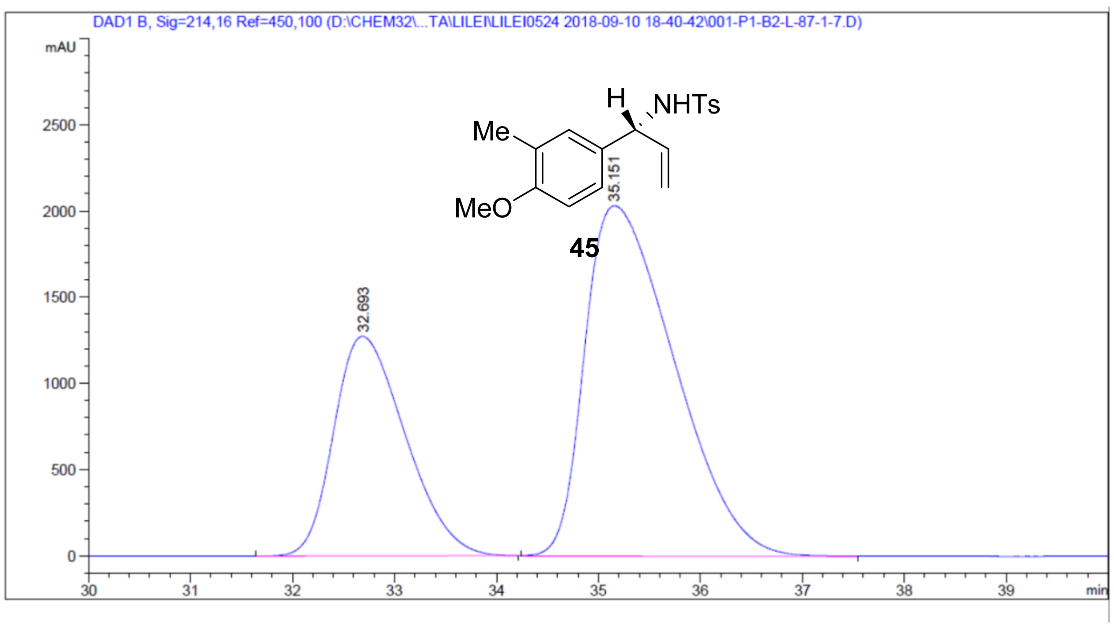

Peak #	RetTime [min]	Type	Width [min]	Area [mAU*s]	Height [mAU]	Area %
1	32.693	BB	0.5731	6.22103e4	1274.28064	33.3895
2	35.151	MM	1.0168	1.24107e5	2034.29370	66.6105

Supplementary Figure 147. HPLC spectra of compound *rac*-**45** and compound **45**

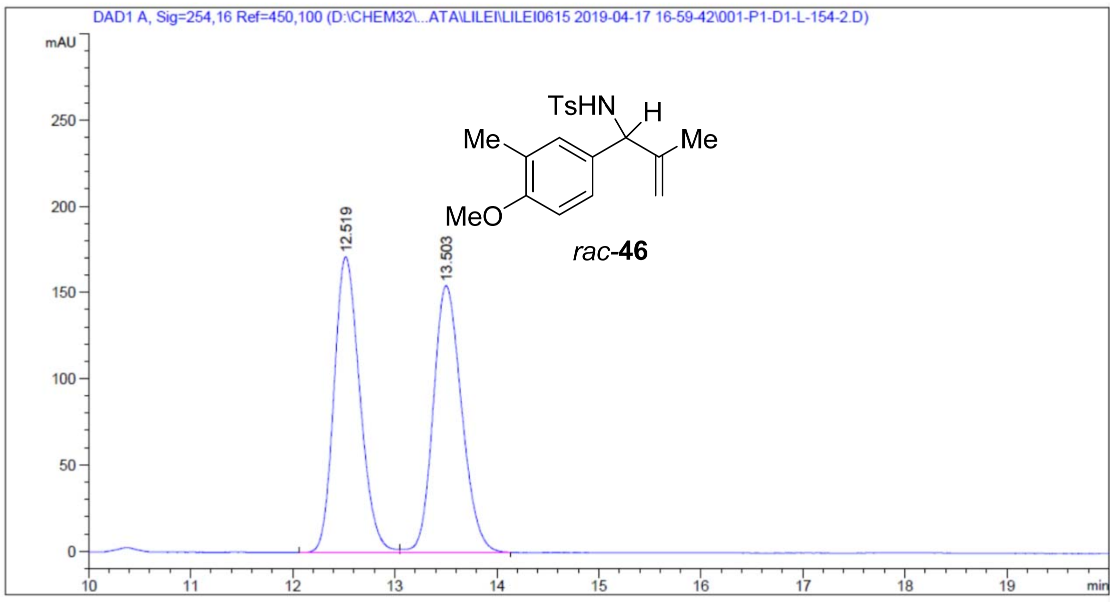

Peak #	RetTime [min]	Type	Width [min]	Area [mAU*s]	Height [mAU]	Area %
1	12.519	BV	0.2740	3021.25244	171.53821	50.1635
2	13.503	VB	0.2967	3001.56201	154.79518	49.8365

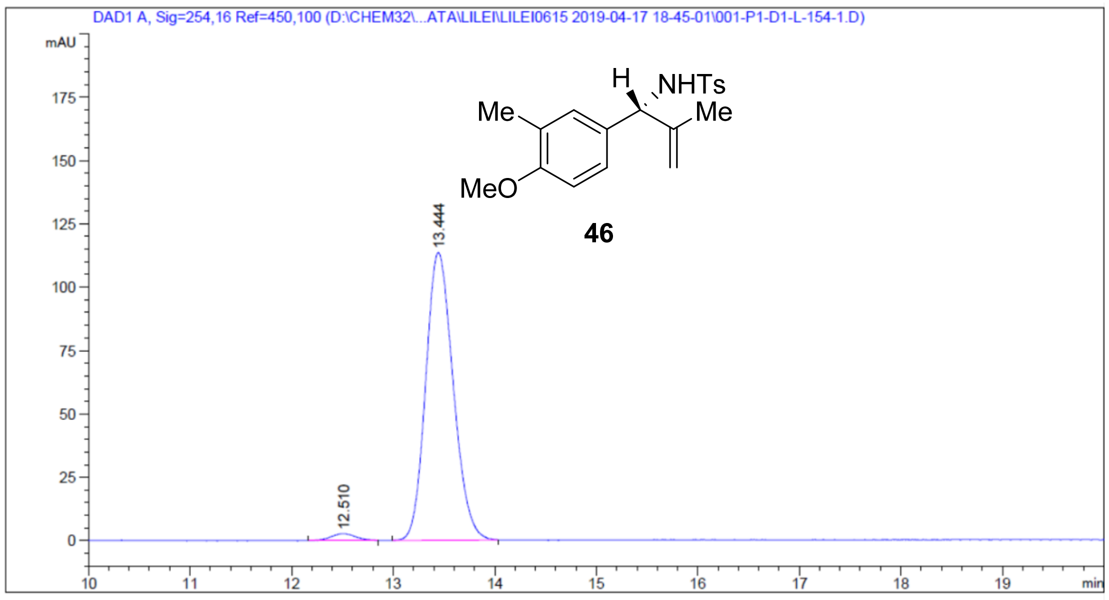

Peak #	RetTime [min]	Type	Width [min]	Area [mAU*s]	Height [mAU]	Area %
1	12.510	VB R	0.2027	47.22183	2.78722	2.1644
2	13.444	BB	0.2769	2134.52783	113.48177	97.8356

Supplementary Figure 148. HPLC spectra of compound *rac*-**46** and compound **46**

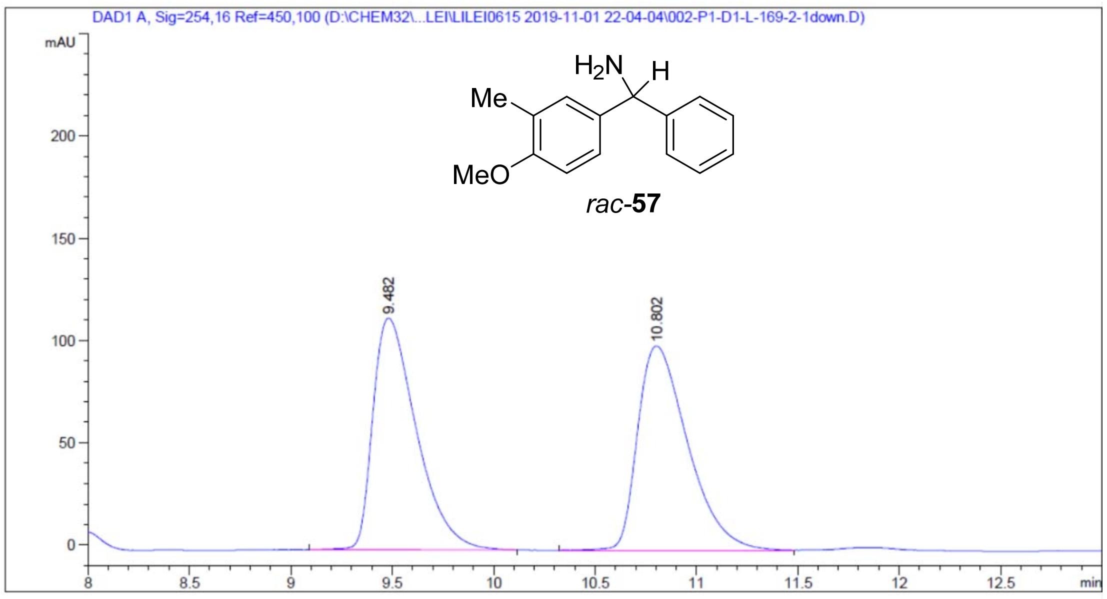

Peak #	RetTime [min]	Type	Width [min]	Area [mAU*s]	Height [mAU]	Area %
1	9.482	BB	0.2261	1687.57690	113.39314	49.5492
2	10.802	VB R	0.2625	1718.28748	99.98280	50.4508

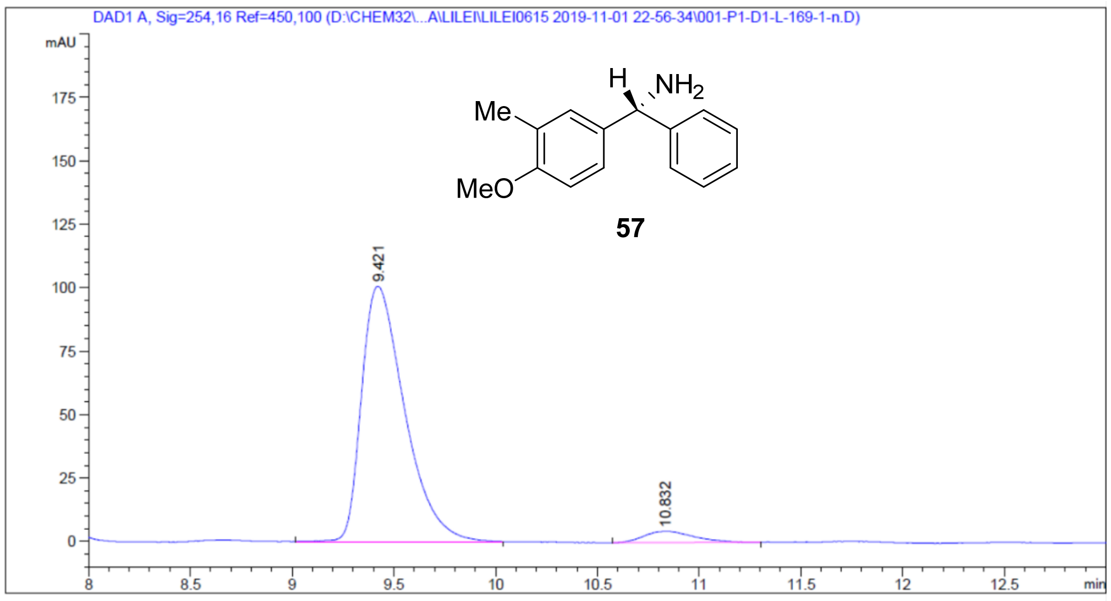

Peak #	RetTime [min]	Type	Width [min]	Area [mAU*s]	Height [mAU]	Area %
1	9.421	BB	0.2218	1481.61804	100.59053	95.1572
2	10.832	BV R	0.1998	75.40411	4.49204	4.8428

Supplementary Figure 149. HPLC spectra of compound *rac*-**57** and compound **57**

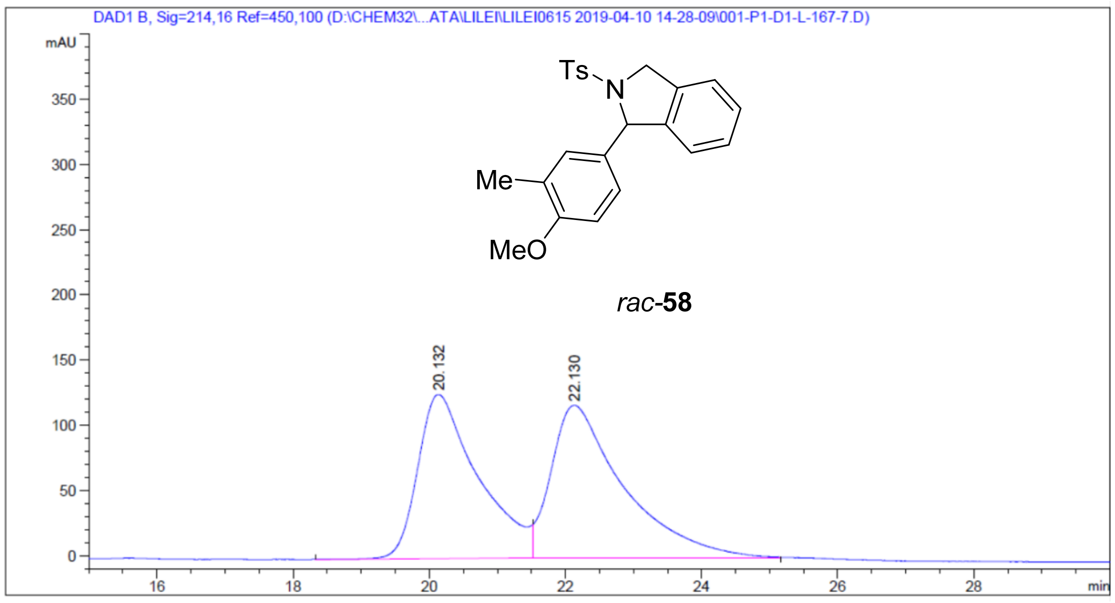

Peak #	RetTime [min]	Type	Width [min]	Area [mAU*s]	Height [mAU]	Area %
1	20.132	MM	1.0285	7760.27783	125.75498	47.4026
2	22.130	MM	1.2278	8610.73242	116.88146	52.5974

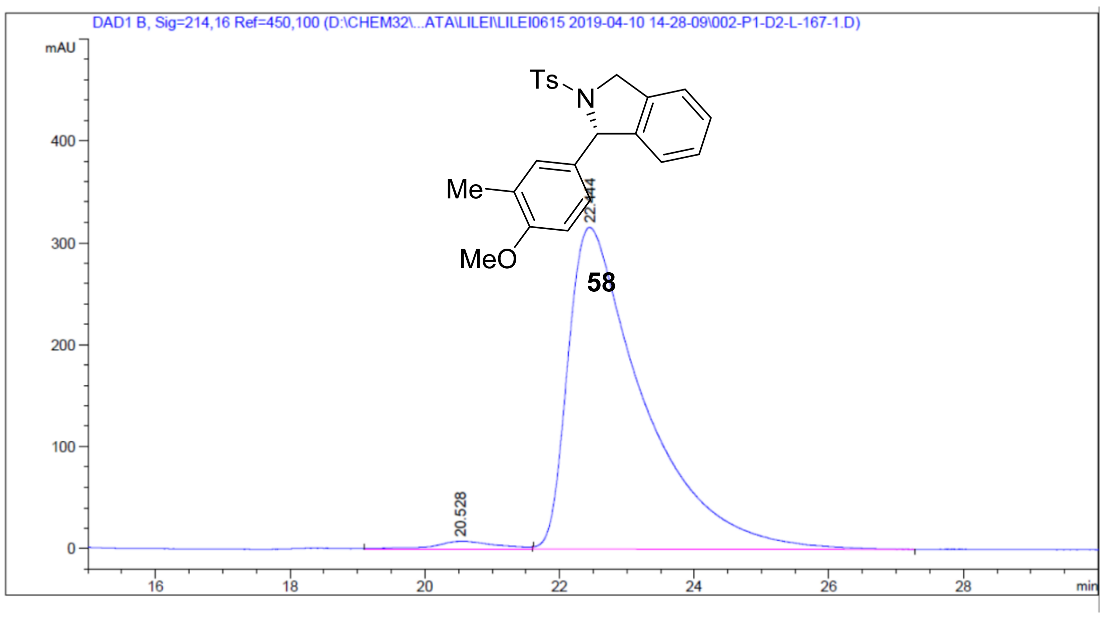

Peak #	RetTime [min]	Type	Width [min]	Area [mAU*s]	Height [mAU]	Area %
1	20.528	MM	1.0519	483.14725	7.65545	1.9206
2	22.444	MM	1.3019	2.46723e4	315.84033	98.0794

Supplementary Figure 150. HPLC spectra of compound *rac*-**58** and compound **58**

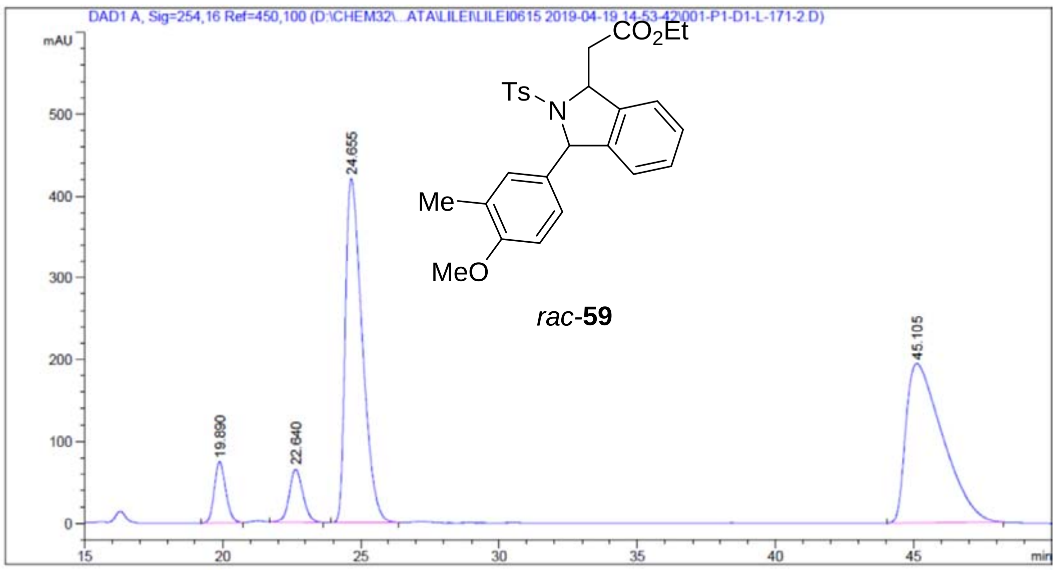

Peak #	RetTime [min]	Type	Width [min]	Area [mAU*s]	Height [mAU]	Area %
1	19.890	BB	0.4106	2137.79077	74.54519	5.2998
2	22.640	BB	0.4104	2247.76270	65.16913	5.5725
3	24.655	BB	0.6237	1.78599e4	420.73657	44.2769
4	45.105	BB	1.0942	1.80914e4	194.75676	44.8508

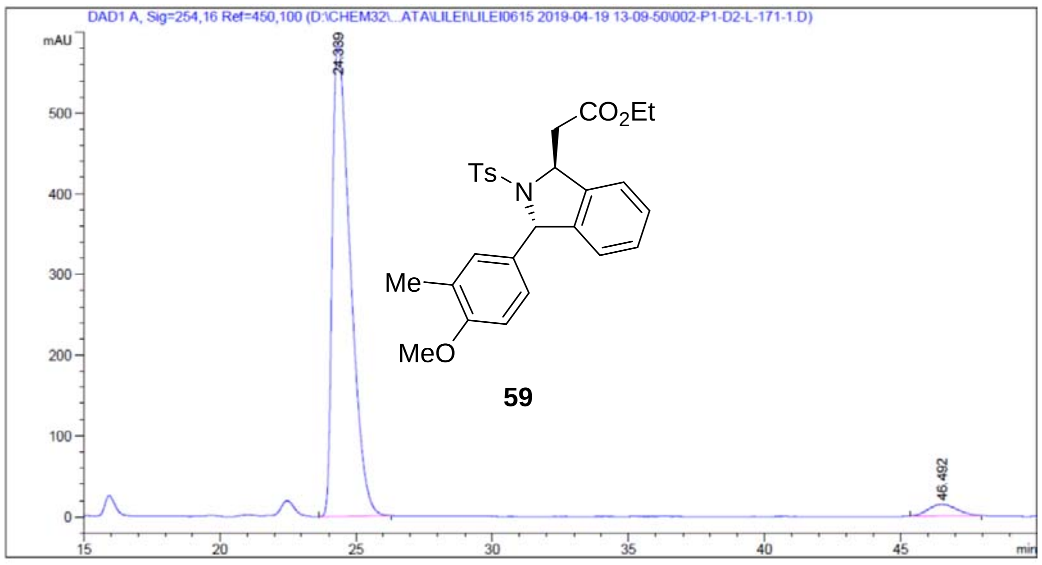

Peak #	RetTime [min]	Type	Width [min]	Area [mAU*s]	Height [mAU]	Area %
1	24.339	BB	0.6710	2.69138e4	587.40125	96.3575
2	46.492	BB	0.8458	1017.39319	14.09255	3.6425

Supplementary Figure 151. HPLC spectra of compound *rac*-**59** and compound **59**

Supplementary References

1. Illa, O. et al. Practical and Highly Selective Sulfur Ylide-Mediated Asymmetric Epoxidations and Aziridinations Using a Cheap and Readily Available Chiral Sulfide: Extensive Studies to Map Out Scope, Limitations, and Rationalization of Diastereo- and Enantioselectivities. *J. Am. Chem. Soc.* **135**, 11951−11966 (2013).
2. Sébastien, C. et al. Asymmetric Sulfur-Ylide-Mediated Formal [4+1]-Annulation Reaction: Scope and Mechanism. *Chem. Eur. J.* **24**, 11417-11425 (2018).
3. Beisel, T. & Manolikakes, G. Palladium-Catalyzed Enantioselective Three-Component Synthesis of α-Substituted Amines. *Org. Lett.* **17**, 3162-3165 (2015).
4. Lindsay, V. N. G., Viart, H. M.-F. & Sarpong, R. Stereodivergent Intramolecular C(sp^3)-H Functionalization of Azavinyl Carbenes: Synthesis of Saturated Heterocycles and Fused N-Heterotricycles. *J. Am. Chem. Soc.* **137**, 8368−8371 (2015).
5. Zhou, Y., Zhou, M., Chen, M., Su, J., Du, J. & Song, Q. β-Ketophosphonates formation via deesterification or deamidation of cinnamyl /alkynyl carboxylates or amides with H-phosphonates. *RSC Adv.* **5**, 103977-103981 (2015).
